# From pattern to process? Dual travelling waves, with contrasting propagation speeds, best describe a self‐organised spatio‐temporal pattern in population growth of a cyclic rodent

**DOI:** 10.1111/ele.14074

**Published:** 2022-07-31

**Authors:** Deon Roos, Constantino Caminero‐Saldaña, David Elston, François Mougeot, María Carmen García‐Ariza, Beatriz Arroyo, Juan José Luque‐Larena, Francisco Javier Rojo Revilla, Xavier Lambin

**Affiliations:** ^1^ School of Biological Sciences University of Aberdeen Aberdeen UK; ^2^ Área de Plagas, Instituto Tecnológico Agrario de Castilla‐y‐León (ITACyL) Valladolid Spain; ^3^ Biomathematics & Statistics Scotland Aberdeen UK; ^4^ Instituto de Investigación en Recursos Cinegéticos IREC (CSIC‐UCLM‐JCCM) Ciudad Real Spain; ^5^ Dpto. Ciencias Agroforestales, ETSIIAA Universidad de Valladolid Palencia Spain; ^6^ Instituto Universitario de Investigación en Gestión Forestal Sostenible Palencia Spain

**Keywords:** patterns, population cycles, population growth rate, spatio‐temporal, synchrony

## Abstract

The dynamics of cyclic populations distributed in space result from the relative strength of synchronising influences and the limited dispersal of destabilising factors (activators and inhibitors), known to cause multi‐annual population cycles. However, while each of these have been well studied in isolation, there is limited empirical evidence of how the processes of synchronisation and activation–inhibition act together, largely owing to the scarcity of datasets with sufficient spatial and temporal scale and resolution. We assessed a variety of models that could be underlying the spatio‐temporal pattern, designed to capture both theoretical and empirical understandings of travelling waves using large‐scale (>35,000 km^2^), multi‐year (2011–2017) field monitoring data on abundances of common vole (*Microtus arvalis*), a cyclic agricultural rodent pest. We found most support for a pattern formed from the summation of two radial travelling waves with contrasting speeds that together describe population growth rates across the region.

## Graphical abstract

The dynamics of cyclic populations distributed in space result from the relative strength of synchronising influences and the limited dispersal of destabilising factors (activators and inhibitors), known to cause multi‐annual population cycles. However, while each of these have been well studied in isolation, there is limited empirical evidence of how the processes of synchronisation and activation‐inhibition act together, largely owing to the scarcity of datasets with sufficient spatial and temporal scale and resolution. In this work, we found most support for a pattern formed from the summation of two radial travelling waves with contrasting speeds that together describe population growth rates across the region.

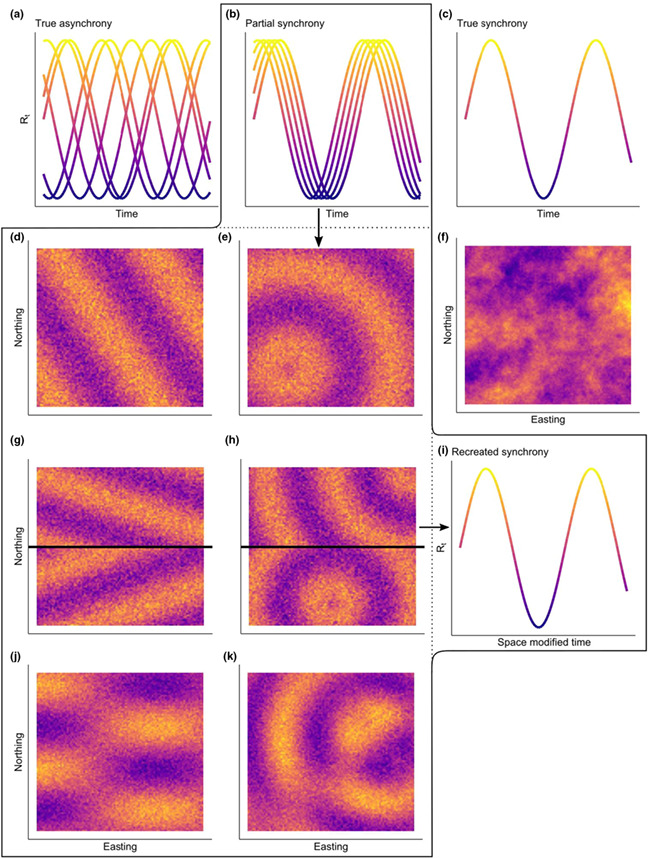

## INTRODUCTION

Classic ecological theory assumes that population dynamics result from interacting organisms in time but in a non‐spatial context (e.g. Lotka–Volterra model). However, these predictions are modified when accounting for restricted species movement by including space and dispersal (Levin, 1974). When interactions between pairs of species, broadly fitting the definition of activator‐inhibitor (e.g. predator–prey, parasite–host, etc.), result in local cycles, incorporating space and accounting for restricted dispersal can give rise to spatio‐temporal patterns (Bjørnstad et al., 2002; De Roos et al., 1991; Johnson et al., 2006; Sherratt, 2001). These dynamic spatial patterns can take various forms, ranging from chaos (Li et al., 2005) to perfect synchrony (Blasius et al., 1999) and much in between.

Causes of synchrony have been attributed to climate conditions (the Moran effect, e.g. Bogdziewicz et al., 2021), dispersal of individuals and trophic interactions. While the Moran effect is often suggested as the cause of synchrony (e.g. Fay et al., 2020), microcosm experiments have strongly implicated an interaction between the dispersal of organisms and their trophic interactions, through the differential depletion of more dense populations, as a potent cause of synchrony (Fox et al., 2013; Vasseur & Fox, 2009).

**FIGURE 1:**
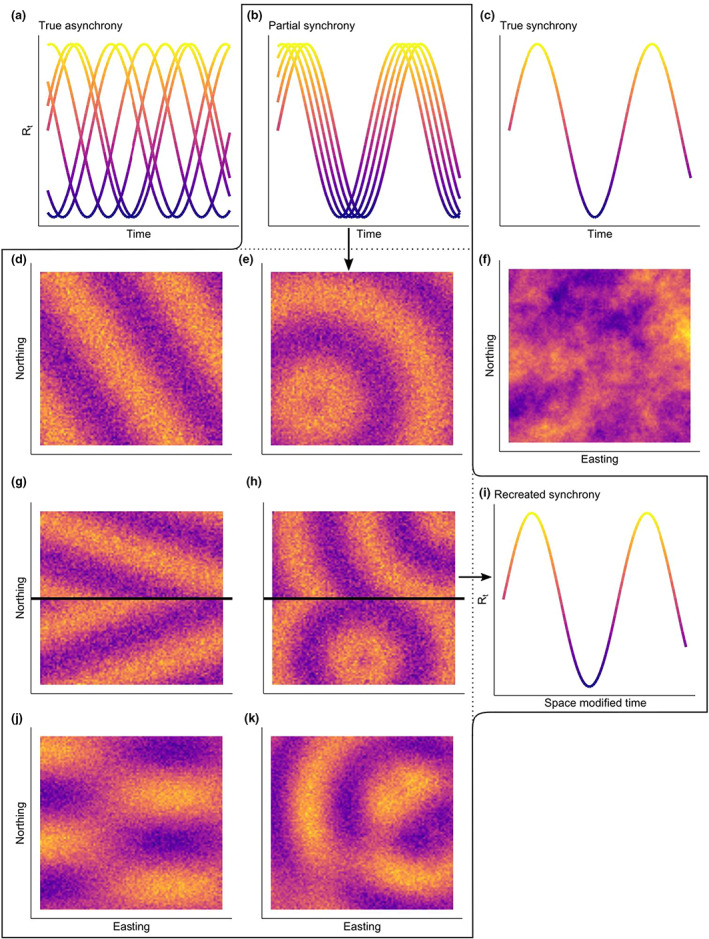
Visual representation of the various hypotheses (via simulated data), where yellow indicates high growth rates and blue low growth rates. (a) Truly asynchronous population cycles, where each population (line) cycles independently of its neighbours. (b) Partial synchrony where the neighbouring populations cycle almost simultaneously, though they are not perfectly synchronised (decomposed into subsequent models). (c) Perfectly synchronised populations where all populations cycle at precisely the same time (where $r_{t,i}$ should best be represented as varying with time only, model N_2_). (d, e, g, h, j, k) All represent the spatial patterns on a given day for the various parameterisations of fitted travelling wave models, described in Table 1. (f) A purely spatial pattern (where any perceived spatio‐temporal pattern is merely spatial, model N_3_, as Sherratt & Smith, 2008 suggested may be the case for the apparent snowshoe hare travelling wave). (d) A single planar wave at a snapshot in time (Moss et al., 2000; Lambin et al., 1998, Bjørnstad et al., 2002, Berthier et al. 2014, model P). (e) Either an expanding or contracting single radial travelling wave (radially expanding from a central location as suggested by Johnson et al., 2006 [model RE] or contracting as suggested by Sherratt & Smith, 2008, [model RC]). (g) Two isolated planar waves separated by a physical feature, the Duero river (inferred from Sherratt & Smith, 2008, model PF). (h) Two radial waves separated by the same physical feature but may be either contracting or expanding (models RFE and RFC). (j) Dual overlapping planar waves, which additively form a single overall pattern (model PD). (k) Either dual overlapping contracting or expanding radial waves, additively forming an overall pattern (models RDE and RDC). (i) The modelling approach of an underlying cycle manifesting itself in the form of partial asynchrony in the data. The graphical pathway of analysis for the selected model (RDE) would be b → k → i, where k → i is carried out according to model RDE in Table 1.

Synchrony itself exists on a spectrum. Of note are periodic travelling waves (termed *partial synchrony* in Figure 1b), whereby the oscillations of population cycles seemingly travel across space over time, either in a single constant direction (i.e. anisotropic, henceforth termed *planar wave*, e.g. Berthier et al., 2014; Bjørnstad et al., 2002; Lambin et al., 1998) or in all directions (i.e. isotropic; henceforth termed *radial wave*, e.g. Johnson et al., 2004), at a given speed. For population cycles linked via a travelling wave, all populations experience the same cycle but do so at different times. As the distance from the wave source increases, their cycles will become increasingly asynchronous for such populations. Conversely, having perfectly synchronised cycles (termed *true synchrony* in Figure 1c) is merely where the wave speed is practically infinite (Jepsen et al., 2016; Sherratt, 2001). In cycles with true synchrony, all populations in a landscape exhibit the same phase of each cycle simultaneously with no spatio‐temporal lag. The opposing end of the synchrony spectrum would be populations that exhibit completely independent cycles (termed *true asynchrony* in Figure 1a).

While travelling waves appear to be routinely detected when datasets are sufficient, there remains much uncertainty. Namely, what form a travelling wave will take when spreading across a natural landscape, what features determine the source location(s) of the wave(s), and whether activator‐inhibitor dynamics play a role in the underlying mechanisms?

Theoretical simulations of travelling waves unfolding in homogenous landscapes suggest the spread should be radial. However, real‐world landscapes include habitat heterogeneity (but see Johnson et al., 2006). Intriguingly, spatial inhomogeneity can lead to the formation of both radial and planar waves via either variation in productivity, connectivity or dispersal. However, theoretical work explicitly investigating the role that heterogeneous landscapes have on travelling waves, by including physical features (e.g. lakes), suggests that waves may originate from these structures with an imparted directionality (Sherratt et al., 2002; Sherratt et al., 2003). If the feature preventing isotropic dispersal is itself linear, then the resulting form of the wave would be expected to be planar. Because heterogeneities are ubiquitous in natural landscapes and affect dispersal and productivity, theory offers no prediction on what pattern should unfold in any real‐world landscape and arguments on any match between empirical and theoretical patterns have been post hoc.

Empirical research projects, which by their nature occur in heterogenous environments, have often used planar wave parameterisations to describe the observed travelling waves in cyclic populations (Berthier et al., 2014; Lambin et al., 1998). Such a mismatch between the predicted (i.e. radial) and observed (i.e. planar) patterns may have two interpretations. The first is that the apparent planar waves are simply a feature of observing a radial wave at too small a spatial scale (feasible given the substantial data requirements [Koenig, 1999]). Alternatively, observed planar waves may reflect real‐world conditions that some simulations fail to account for (e.g. heterogeneous landscapes regarding the distribution of habitats and organisms). Thus, true planar waves may arise due to approximately linear physical features in the landscape. Building on Sherratt and Smith's (2008) work, which suggested physical features may generate travelling waves, Berthier et al. (2014) invoked quasi‐linear physical features in their landscape as potentially responsible for planar waves in cyclic water vole populations. However, because of the necessary theoretical assumptions for how physical features interact with organisms (resulting in boundary conditions that are hard to quantify empirically), Berthier et al. (2014) could not ascertain which plausible features were responsible. This reflects the challenge of translating theoretical assumptions into real‐world characteristics and vice‐versa.

An alternative to physical features generating travelling waves is the suggestion that they are generated at foci with particular features. Such features may include: areas with high densities (Bugrim et al., 1996); areas where predators were introduced (Gurney et al., 1998; Sherratt, 2001; Sherratt, 2016; Sherratt et al., 1997; Sherratt et al., 2000) and areas of high population connectivity or habitat quality (Johnson et al., 2006). The epicentre hypothesis posits that travelling waves recurrently form at epicentres. These epicentres reflect regions in space with defined characteristics (e.g. highly connected populations in high‐quality habitats) that give rise to waves. Johnson et al. (2006) invoked the epicentre hypothesis to explain travelling waves in cyclic larch bud moths. They proposed that waves emanate from regions with high‐quality, well‐connected populations, which spread to more distant populations, resulting in partially synchronous cycles.

Related to uncertainties with what generates a wave is the ambiguity of theory on the resulting direction of travel relative to the source. For the larch budmoth, it has been suggested that waves travel outwards from epicentres (Johnson et al., 2006), resulting in expanding radial travelling waves. Conversely, alternative studies have suggested the opposite may occur, whereby waves begin at hostile environment boundaries (i.e. where individuals die if entered) and contract inwards towards a central location (Sherratt, 2003; Sherratt & Smith, 2008). There has been no empirical research with an analytical approach that explicitly tested for such expanding or contracting waves.

As a wave spreads via dispersal and trophic interaction (Vasseur & Fox, 2009), the cycle spreads across a landscape from one population to the next. Each population experiences the same cyclical successions of activation or inhibition of growth rates. Such changes to a population's growth rate are, in part, dependent on neighbouring populations. For instance inhibition may represent the spread of agents such as pathogens or predators from one population to the next, resulting in suppressed local populations as the respective wave passes. Theoretical expectations of travelling waves have been supported by empirical evidence from a variety of fields, all of which can be considered to have such activator‐inhibitor relationships; for example herbivore–plant, predator–prey, parasite–host (Berthier et al., 2014; Bierman et al., 2006; Johnson et al., 2004; Lambin et al., 1998; Mackinnon et al., 2001; Moss et al., 2000), susceptible‐recovered, (Cummings et al., 2004; Grenfell et al., 2001), death and regeneration (Sprugel, 1976) and cellular biochemistry (Bailles et al., 2019; Müller et al., 1998). Within such systems, the cumulative impact of both activator and inhibitor gives rise to the overall cyclic pattern.

The conceptualisation of population cycles as activation–inhibition, as well as the wealth of theoretical literature considering the role of such activation and inhibition accompanied by restricted dispersal in spatial patterns (Bjørnstad et al., 2002; De Roos et al., 1991; Johnson et al., 2006; Levin, 1974; Sherratt, 2001; Sherratt et al., 2000), implies that statistical representation of empirical data might decompose the overall pattern in growth and retrieve evidence of two contributing travelling waves, promoting and inhibiting growth, as found in non‐ecological travelling waves (Kapustina et al., 2013; Martinet et al., 2017). Additionally, the interplay between activator and inhibitor dispersal abilities has been suggested as a component that leads to the formation of waves (Johnson et al., 2006).

Building on exceptional data, this study evaluates a suite of hypotheses, which are flexible phenomenological descriptions of travelling waves, representing theoretical or empirical work and their logical extensions. Given the richness of our dataset, we can lessen the requirements for simplified caricatures and consider more complex forms. Our approach considers an initial demarcation between radial and planar waves, including whether the radial waves contract or expand. These hypotheses are further divided to represent either a single or multiple travelling waves (as simulated in Johnson et al., 2006), each split into whether multiple waves are isolated by physical features or coalesce into a single pattern reflecting activator‐inhibitor dynamics. We used abundance indices of a rodent crop pest from a study site spanning >35,000 km^2^ over nearly 6 years. We find evidence of a single cumulative spatio‐temporal pattern consisting of two expanding radial travelling waves, which we propose may arise due to activator‐inhibitor dynamics.

## MATERIALS AND METHODS

### Study species

The common vole (*Microtus arvalis*) is a small rodent inhabiting natural grasslands and agricultural ecosystems in Europe. It is prey for both specialist and generalist predators alike (Mougeot et al., 2019) and is host to multiple direct and vector‐transmitted pathogens (Rodríguez‐Pastor et al., 2018). Common voles are frequent farmland pests causing crop damage and disease spillovers during population outbreaks that occur every 3–4 years (Jacob & Tkadlec, 2010; Mougeot et al., 2019; Rodríguez‐Pastor et al., 2018). Common voles have been extensively monitored for pest management across our study site (>35,000 km^2^) since 2011 (see Mougeot et al., 2019, Jacob et al., 2020 and Herrero‐Cófreces et al., 2021 for complementary temporal trends).

### Study site

**FIGURE 2:**
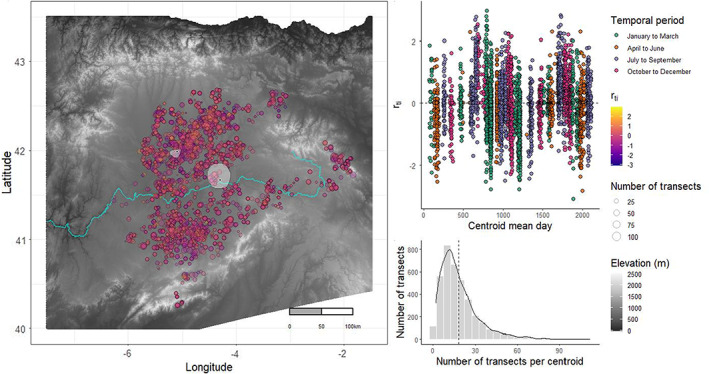
Left: Map of the Duero basin, coloured (grey scale) according to elevation (m) with mountains in the north, east and south visible as white regions. The Bay of Biscay is visible in the north. Points represent the centroid locations and are coloured according to the population growth rate and sized according to the number of transects in each centroid. Estimated epicentre locations are noted with the black dots with white edges with their respective 95% CI profiles shown with the white polygons (the epicentre to the southeast is the activator, while inhibitor is in the north west). The Duero River is visible as the turquoise line running east to west. Elevation data were downloaded from copernicus[dot]EU (EU‐DEM v1.1) and waterway data from Ea[dot]europa[dot]eu. Top right: Time series of population growth rates for each centroid, is similarly coloured according to temporal periods. The horizontal dashed line shows a growth rate of zero (i.e. no growth). Bottom right: A histogram showing the number of transects contained in each centroid. The vertical dashed line shows the mean of 18.5.

We (ITACyL, Instituto Tecnológico Agrario de Castilla y León) collected data on vole abundances in Castilla‐y‐León (CyL), NW Spain. CyL is a large (94,226 km^2^), relatively flat, semi‐arid agro‐steppe plateau encircled by mountains and bisected east to west by the ca. 25–150 m wide Duero River (Figure 2). As a result of land‐use changes (ca. the 1970s), common voles colonised the plateau from the adjacent mountain ranges in the north, east and south (Jareño et al., 2015; Luque‐Larena et al., 2013). Within the wider region, common voles are believed to occur at higher densities within the plateau than in the surrounding mountains, likely due to the region's agricultural practices (Roos et al., 2019). A particular area in the centre of CyL *(Tierra de Campos)* is known to practitioners as problematic due to early, large or persistent outbreaks.

While not a perfectly homogenous landscape, the plateau likely presents a ‘best real‐world match’ for conditions used in most theoretical research, which do not account for landscape features (but see Sherratt & Smith, 2008). However, there are two complicating physical features: the Duero river and surrounding mountain ranges. If physical features are related to the form of a wave, we may expect either planar waves that travel north and south due to the river or a contracting radial wave resulting from the encircling mountains.

### Data collection

We used a widely employed calibrated abundance index method, based on vole presence, to monitor vole abundance at large spatial scales (Jareño et al., 2014; Roos et al., 2019). Transects, up to 99 m in length (dependent on the field's length), were surveyed in stable linear landscape features (field, track or ditch margins) to estimate vole abundance from November 2011 until September 2017 (n=42,973). Margins are known to be reservoir habitats for voles, from which they colonise adjacent fields during outbreak periods (Rodríguez‐Pastor et al., 2016). Each transect was divided into 3 m sections (33 in total), and the presence or absence of one or more signs of vole activity (i.e. latrines by burrows, fresh vegetation clippings and recent burrow excavations) in each section was noted. The proportion of sections with signs of vole presence per transect was then used as the abundance index. The number of surveys carried out at any time varied adaptively with the perceived risk of an outbreak (according to changes in estimated abundance in previous monitoring surveys).

Analyses of travelling waves typically use measures that can detrend from long‐term temporal trends and autocorrelation, such as phase angle or log difference growth rates (Liebhold et al., 2004; Vindstad et al., 2019). As such, the response variable typically used in all models is proportional growth rate, $r_{t,i}=\ln\left(N_{t+1,i}\right)–\ln\left(N_{t,i}\right)$, where $N_{t,i}$ is the abundance index for site i at time t (Berryman, 2002; Royama, 1992). A benefit of using $r_{t,i}$, rather than $\ln\left(N_{t,i}\right)$, is that any multiplicative effects of site quality are cancelled out, provided they are constant over time. To calculate $r_{t,i}$, vole abundance indices are required at the same location in successive periods (i.e. t and t+1). Given that exact transect locations were rarely reused in successive months, and surveys took place throughout the year rather than discrete seasons, the data had to be aggregated to consistent locations and times to calculate the growth rate. As such, transects were temporally aggregated into a respective yearly quarter (e.g. January to March 2014). Spatial aggregation could have been achieved by allocating samples to a grid of a given size. However, doing so can result in points near adjacent to each other becoming separated by the grid and assigned with ‘less synchronous’ samples. Given that points in close spatial proximity would be expected to act near synchronously, assigning samples to a grid would reduce the information about spatio‐temporal patterns in the data. Instead, transects were spatially aggregated by sequentially selecting an unassigned transect as a reference point for the $i^{\mathrm{th}}$ centroid and assigning all unassigned transects within a 5 km radius to that centroid, and repeating until all transects had been allocated (see Figure 2). Once complete, the mean Julian day, *X* and *Y* UTM (Universal Transverse Mercator) and index were calculated for all transects assigned to centroid $i^{\prime }$ and period $t^{\prime }$. Where a centroid had successive values of $N_{t\prime ,i\prime }$ and $N_{t\prime +1,i\prime }$ available, the corresponding proportional growth rate ($r_{t\prime ,i\prime }$) was calculated.

Spatial aggregations based on 5, 10 and 15 km radii were considered, as were 1‐, 2‐ and 3‐month periods. Final selection was determined by selecting the combination (5 km radius and 3‐month periods), which gave the largest sample size, favouring spatial resolution when applicable. A constant of 3.03 was added to N to avoid zero entries (3.03 was the lowest non‐zero value of *N* observed). The final dataset consisted of 3751 observations of $r_{t,i}$ (Figure S1).

### Analysis

Bespoke models were constructed for all considered parameterisations of travelling waves (summarised in Figure 1; Table 1) based on previous modelling approaches (Berthier et al., 2014; Lambin et al., 1998; Moss et al., 2000). All the travelling wave models contained at least three components. The first component estimated distance from either an estimated planar direction (θ) or an estimated epicentre location (γ and ψ, *Distance equation*, Table 1). The next component used these distances and converted them to a space‐modified time variable (*Space‐modified time equation*, Table 1), whereby the speed of the wave was estimated (ζ, Table 1). The space‐modified time variable accounts for the potential that while two locations may be sampled on the same day, their growth rates may be at different cycle phases depending on their distance and the form of the wave under consideration. The resulting space‐modified time variable(s) was then included as an explanatory variable(s) in a GAM (generalised additive models) to explain growth rates (*Growth equation*, Table 1). These models reflect various ways to modify space and time so that one or two underlying waves can explain the dynamics at each location (see Figure 1). The parameters defining the space‐modified time variables were estimated using a stochastic annealing (SANN) optimiser (Bolker, 2008), using 15,000 iterations for each model. SANN initial values were determined using a direct search method. Conditional on the values of the space‐modified time variables, the underlying waves were fitted using GAMs as described below.

**TABLE 1:** Summary of analysis including model label, hypothesis and equations used to estimate distance, space‐modified time and growth rate. Where $r_{t,i}$ is the log difference growth rate of centroid *i* at time *t*, $\alpha_{1}$ is the intercept term, ϵ is the Normal distributed residual error, T is day since start of study, f is used to represent thin‐plate smoothing splines with a maximum of 12 bases ($f_{1}$, $f_{2}$, $f_{\text{North}}$ and $f_{\text{South}}$), $f_{p}$ is a thin‐plate tensor product with a maximum of 10 bases in each dimension, X is the mean centred easting coordinate (UTM), Y is the mean centred northing coordinate (UTM), D is the distance of a centroid from either a planar angle or radial epicentre, θ is the angle of a planar wave (radian), ρ is the space‐modified time variable, ζ is the constant speed of the wave, γ and ψ are the easting and northing coordinates of a radial wave epicentre (mean centred UTM), N and S denote north and south of the Duero river. The number of additional travelling wave parameters for each model are included. ΔAIC (adjusted for additional wave parameters) values are included.

	Hypothesis	Distance equation	Space‐modified time equation	Growth rate equation	Number of additional parameters	ΔAIC
*N*_*1*_	Null	NA	NA	$r_{t,i}=\alpha_{1}+\epsilon_{t,i}$	NA	1089.97
*N*_*2*_	Phase‐locked	NA	NA	$r_{t,i}=\alpha_{1}+f\left(T_{t,i}\right)+\epsilon_{t,i}$	NA	207.49
*N*_*3*_	Static spatial pattern	NA	NA	$r_{t,i}=\alpha_{1}+f_{p}\left(X_{t,i}Y_{t,i}\right)+\epsilon_{t,i}$	NA	1086.83
*RE*	Single expanding radial wave	$D_{i}=-\sqrt{{\left(\gamma -X_{i}\right)}^{2}+{\left(\psi -Y_{i}\right)}^{2}}$	$\rho_{t,i}=T_{t,i}+\left(\frac{1}{\zeta }\right)D_{i}$	$r_{t,i}=\alpha_{1}+f\left(\rho_{t,i}\right)+\epsilon_{t,i}$	3	183.28
*RC*	Single contracting radial wave	$D_{i}=\sqrt{{\left(\gamma -X_{i}\right)}^{2}+{\left(\psi -Y_{i}\right)}^{2}}$	$\rho_{t,i}=T_{t,i}+\left(\frac{1}{\zeta }\right)D_{i}$	$r_{t,i}=\alpha_{1}+f\left(\rho_{t,i}\right)+\epsilon_{t,i}$	3	183.36
*P*	Single planar wave	$D_{i}=\sin\left(\theta \right)X_{i}+\cos\left(\theta \right)Y_{i}$	$\rho_{t,i}=T_{t,i}+\left(\frac{1}{\zeta }\right)D_{i}$	$r_{t,i}=\alpha_{1}+f\left(\rho_{t,i}\right)+\epsilon_{t,i}$	2	181.34
*RFE*	Two expanding radial waves separated by river	$D_{N,i}=-\sqrt{{\left(\gamma_{N}-X_{i}\right)}^{2}+{\left(\psi_{N}-Y_{i}\right)}^{2}},\text{if}\;Y_{i}\geq 5,068\;m$	$\rho_{N,t,i}=T_{t,i}+\left(\frac{1}{\zeta_{N}}\right)D_{N,i}$	$r_{t,i}=\alpha_{1}+f_{\text{North}}\left(\rho_{N,t,i}\right)+f_{\text{South}}\left(\rho_{S,t,i}\right)+\epsilon_{t,i}$	6	60.78
		$D_{S,i}=-\sqrt{{\left(\gamma_{S}-X_{i}\right)}^{2}+{\left(\psi_{S}-Y_{i}\right)}^{2}},\text{if}\;Y_{i}<5,068\;m$	$\rho_{S,t,i}=T_{t,i}+\left(\frac{1}{\zeta_{S}}\right)D_{S,i}$			
*RDE*	Dual overlapping expanding radial waves	$D_{1,i}=-\sqrt{{\left(\gamma_{1}-X_{i}\right)}^{2}+{\left(\psi_{1}-Y_{i}\right)}^{2}}$	$\rho_{1,t,i}=T_{t,i}+\left(\frac{1}{\zeta_{1}}\right)D_{1,i}$	$r_{t,i}=\alpha_{1}+f_{1}\left(\rho_{1,t,i}\right)+f_{2}\left(\rho_{2,i,t}\right)+\epsilon_{i,t}$	6	0
		$D_{2,i}=-\sqrt{{\left(\gamma_{2}-X_{i}\right)}^{2}+{\left(\psi_{2}-Y_{i}\right)}^{2}}$	$\rho_{2,t,i}=T_{t,i}+\left(\frac{1}{\zeta_{2}}\right)D_{2,i}$			
*RFC*	Two contracting radial waves separated by river	$D_{N,i}=\sqrt{{\left(\gamma_{N}-X_{i}\right)}^{2}+{\left(\psi_{N}-Y_{i}\right)}^{2}},\text{if}\;Y_{i}\geq 5068\;m$	$\rho_{N,t,i}=T_{t,i}+\left(\frac{1}{\zeta_{N}}\right)D_{N,i}$	$r_{t,i}=\alpha_{1}+f_{\text{North}}\left(\rho_{N,t,i}\right)+f_{\text{South}}\left(\rho_{S,t,i}\right)+\epsilon_{t,i}$	6	109.17
		$D_{S,i}=\sqrt{{\left(\gamma_{S}-X_{i}\right)}^{2}+{\left(\psi_{S}-Y_{i}\right)}^{2}},\text{if}\;Y_{i}<5,068\;m$	$\rho_{S,t,i}=T_{t,i}+\left(\frac{1}{\zeta_{S}}\right)D_{S,i}$			
*RDC*	Dual overlapping contracting radial waves	$D_{1,i}=\sqrt{{\left(\gamma_{1}-X_{i}\right)}^{2}+{\left(\psi_{1}-Y_{i}\right)}^{2}}$	$\rho_{1,t,i}=T_{t,i}+\left(\frac{1}{\zeta_{1}}\right)D_{1,i}$	$r_{t,i}=\alpha_{1}+f_{1}\left(\rho_{1,t,i}\right)+f_{2}\left(\rho_{2,i,t}\right)+\epsilon_{i,t}$	6	53.16
		$D_{2,i}=\sqrt{{\left(\gamma_{2}-X_{i}\right)}^{2}+{\left(\psi_{2}-Y_{i}\right)}^{2}}$	$\rho_{2,t,i}=T_{t,i}+\left(\frac{1}{\zeta_{2}}\right)D_{2,i}$			
*PF*	Two planar waves separated by river	$D_{N,i}=\sin\left(\theta_{N}\right)X_{i}+\cos\left(\theta_{N}\right)Y_{i},\text{if}\;Y_{i}\geq 5068\;m$	$\rho_{N,t,i}=T_{t,i}+\left(\frac{1}{\zeta_{N}}\right)D_{N,i}$	$r_{t,i}=\alpha_{1}+f_{\text{North}}\left(\rho_{N,t,i}\right)+f_{\text{South}}\left(\rho_{S,t,i}\right)+\epsilon_{t,i}$	4	82.62
		$D_{S,i}=\sin\left(\theta_{S}\right)X_{i}+\cos\left(\theta_{S}\right)Y_{i},\text{if}\;Y_{i}<5068\;m$	$\rho_{S,t,i}=T_{t,i}+\left(\frac{1}{\zeta_{S}}\right)D_{S,i}$			
*PD*	Dual overlapping planar waves	$D_{1,i}=\sin\left(\theta_{1}\right)X_{i}+\cos\left(\theta_{1}\right)Y_{i}$	$\rho_{1,t,i}=T_{t,i}+\left(\frac{1}{\zeta_{1}}\right)D_{1,i}$	$r_{t,i}=\alpha_{1}+f_{1}\left(\rho_{1,t,i}\right)+f_{2}\left(\rho_{2,t,i}\right)+\epsilon_{i,t}$	4	90.61
		$D_{2,i}=\sin\left(\theta_{2}\right)X_{i}+\cos\left(\theta_{2}\right)Y_{i}$	$\rho_{2,t,i}=T_{t,i}+\left(\frac{1}{\zeta_{2}}\right)D_{2,i}$			

Three versions of a ‘null’ model (i.e. no travelling wave pattern) were included in the analysis and fitted using GAMs alone. These included a true null model (N_1_), a model which assumed true synchrony (N_2_) and a final model which proposed growth rates were explained by space alone (N_3_) (Table 1).

All GAM components (*Growth equation*, Table 1) assumed a Normal distribution for random errors and included a weighting term. The weighting term was $w_{t,i}=\sqrt{\frac{n_{t,i}\times n_{t-1,i}}{n_{t,i}+n_{t-1,i}}}$, where $w_{t,i}$ is the weights for centroid i at time t, and $n_{t,i}$ is the number of surveys at centroid i in time t or t−1. The term sought to account for observation variance being proportional to $\frac{1}{n}$ caused by the adaptive vole monitoring intensity. The number of transects per centroid varied over time from 2 to 111, with a mean of 18.5. The appropriateness of the weight term was checked by plotting model residuals against the weighting term (Figure S3).

All bespoke models reflected either radial or planar wave(s) parameterisations. Models P, RE and RC were the simplest and included either a single planar (P), expanding radial (RE), or contracting radial (RC) travelling wave. A further suite of models assumed the presence of two spatially isolated, that is non‐interfering, waves separated by the Duero river, with the waves being either planar (PF), expanding radial (RFE), or contracting radial (RFC). The potential for a single pattern informed by dual additive, overlapping waves was captured by allowing models to have two waves, either planar (PD), expanding radial (RDE) or contracting radial (RDC) waves. These models assumed that both waves influenced all populations in the landscape. This suite of models represents various predicted forms of travelling waves and some logical extensions to ensure a broad set of plausible candidate models were considered. Given the richness of our data, the panel of models considered extends previous research that has generally used a single form or descriptive methods that could not rule out competing hypotheses. Using this approach, we can assess which description of the spatio‐temporal patterns is most supported by our data. Parameterisations of each travelling wave model are included in Table 1.

The final model was chosen using ΔAIC (Table 1) where AIC, as reported by the final GAM, was adjusted to incorporate the additional number of wave parameters as;

AdjustedAIC=AIC+2K,

where *K* is the number of wave parameters (Table 1).

Confidence profiles for each parameter were determined using profiling as described in Bolker (2008). All analyses and visualisations were carried out in R version 4.0.2 (R Core Team, 2020) using the *mgcv* (Wood, 2011), *emdbook* (Bolker, 2020), *ggplot2* (Wickham, 2016) and *patchwork* (Pedersen, 2020) packages. The code used for the analysis is embedded in supplementary material 1.

Given that our analyses use novel models, we include in Supplementary Material 2 the analyses of two simulated datasets of a travelling wave to demonstrate that the statistical method can reliably estimate known parameter values. The first simulation is of a single radial expanding travelling wave which is analysed with the corresponding model (Table 1, model RE). The second uses the same core travelling wave but includes samples gathered via an adaptive monitoring design, whereby a core area is monitored intensively, with non‐core areas only marginally likely to be surveyed during periods of exceptionally high growth. The first simulation demonstrates the ability of our modelling to recover known parameters. In addition, the analysis of the second simulated dataset investigates if the presence of an adaptive monitoring design necessarily disrupts epicentre estimation. Model assumption checks, residual plots and summaries of each model are included in Supplementary Material 3.

A public data repository is available at https://doi.org/10.5281/zenodo.6460815.

## RESULTS

The null models (N_1_, N_2_ and N_3_) were discarded through model selection (see supplementary material 1), indicating that it is unlikely that there was true synchrony (N_2_) or that the observed growth rates are related to static environmental conditions (N_3_). The relative lack of support for N_2_ (true synchrony, ΔAIC=207.49) suggests that the population dynamics are not fully synchronised, though, given the speeds estimated for the waves (Table 2), it appears that the regional dynamics are closer to true synchrony than true asynchrony.

**TABLE 2:** Summary of RDE wave parameter estimates. Labels are as noted in Table 1

	Parameter	Parameter label	Estimate	Lower 95% CI	Upper 95% CI	Units
1st wave	Centroid (N)	$\gamma_{1}$	−41,723	−51,645	−33,626	UTM (mean centred)
	Centroid (E)	$\psi_{1}$	28,414	18,556	29,897	UTM (mean centred)
	Speed	$\zeta_{1}$	405	316	528	m per day
2nd wave	Centroid (N)	$\gamma_{2}$	23,675	5161	42,607	UTM (mean centred)
	Centroid (E)	$\psi_{2}$	−8675	−21,536	11,351	UTM (mean centred)
	Speed	$\zeta_{2}$	2287	1783	2941	m per day

Of the models which assumed the presence of travelling waves, RDE (dual expanding radial travelling waves) was unambiguously selected, with the following model (dual contracting radial, RDC) having a ΔAIC=53.2. The final model had epicentres estimated 75.2 km apart (Figure 2; Table 2). The first is in a well‐known problematic area with higher‐than‐average vole abundances, with recurrent and severe outbreaks (*Tierra de Campos*). In contrast, the second was positioned further southeast, in an area that experiences lower than average abundances (see Figure S2).

**FIGURE 3:**
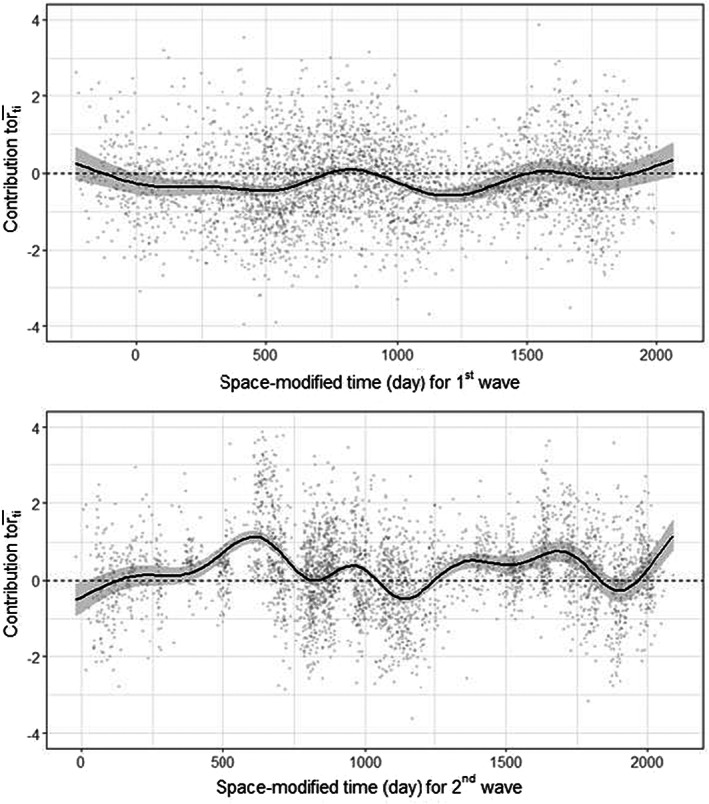
Conditional predictions, showing the contribution of the 1st (slow) and 2nd (fast) waves, including the intercept, to mean growth rate ($\bar{r_{\mathrm{it}}}$, *y*‐axis) over space‐modified time (*x*‐axis) represented by the solid black lines, with 95% confidence intervals represented by the grey ribbons. Horizontal black dashed line indicates a growth rate of 0. The light grey points represent the partial residuals for the respective smoothing spline.

Additionally, when plotting the predicted growth rates with the space‐modified time variables (Figure 3), the possibility that the overall pattern can be decomposed into possible activator and inhibitor influences (themselves, phenomenological statistical descriptions) on vole population growth is suggested; the first, slow‐wave predominantly inhibited growth and was estimated to travel radially at 148 km per year, while the second, faster wave was estimated to travel radially at 835 km per year and generally promoted growth. When the effects of both of these waves are visualised over the region, the cumulative spatio‐temporal pattern becomes apparent (Video S1) with a speed of approximately 0.9 km per day (or 329 km per year, calculated by extracting the furthest south predictions where $r_{t}>0.5$ [i.e. the wave front] at two arbitrarily chosen times, then calculating the distance between those and dividing by the difference in time).

In Supplementary Material 2, we demonstrate that the statistical methods employed herein can reliably recover known parameter values of the simulated travelling wave, regardless of the employment of an adaptive monitoring design. Epicentre coordinates and speeds were estimated to be within 0.2 to 1.1 km and 5 to 10 m per day of the true values, indicating that our statistical methods can reliably recover the underlying spatial pattern when the correct model is used.

## DISCUSSION

We find clear evidence of a self‐organising spatio‐temporal pattern in the population growth rates of common voles in Castilla‐y‐León, resulting from two travelling waves spreading radially at contrasting speeds. Furthermore, in line with Johnson et al. (2004), we find that the pattern is best approximated as two expanding radial travelling waves. However, the waves detected here are not independent, as in Johnson et al. (2004), instead acting additively as activator and inhibitor, suggesting they may be more than phenomenological descriptions of an overall pattern. The dual expanding, fast and slow radial waves are of a form not previously observed in the empirical literature. Still, they align with the fundamental interactions of activators (e.g. host) and inhibitors (e.g. pathogens) in population cycles. Such activation and inhibition and their spatial diffusion are similarly believed to be the processes generating synchrony (Vasseur & Fox, 2009). Further, activator and inhibitor dynamics are intrinsic to formal travelling wave simulations. As such, we find convergence between understandings of synchrony, travelling waves and population cycles.

### True synchrony or partial synchrony?

While we refer to the population cycles of common voles in CyL as partially synchronous, various studies have apparently demonstrated that cyclic populations, both of common voles and other cyclic species, occur synchronously. To understand this apparent contradiction, it is important to note that synchrony occurs not as a dichotomous state but as a spectrum (Bjørnstad et al. 1999; Koenig, 1999, see Figure 1). Nevertheless, the dichotomous representation of synchrony has led to an approach whereby evidence of synchrony (notably synchrony which decays with distance) can be perceived as evidence, or lack‐there‐of, of true synchrony (Andersson & Jonasson, 1986; Erlinge et al., 1999; Fay et al., 2020; Huitu et al., 2003; Huitu et al., 2008; Lambin et al., 2006; Sundell et al., 2004). The terms ‘synchrony’ and ‘asynchrony’, which imply a dichotomous state, may lead to the view that there are no nuanced forms of synchrony.

If travelling waves are ubiquitous in cyclic populations, a crucial component for detecting such nuanced forms of synchrony, overcome in the present study, is the requirement for a vast amount of data to distinguish between more subtle forms (Koenig, 1999). Early descriptions of synchrony in population cycles were largely limited to qualitative assessments, where populations were deemed synchronous if they peaked sometime in the same year (e.g. Andersson & Jonasson, 1986). While such qualitative assessments of synchrony may reflect genuine true synchrony, they likely suffer from temporal aggregations, that is population synchrony is deemed to have occurred because the same phase is experienced within the same broad period. While research on synchrony has become more quantitative, some subsequent attempts to characterise synchrony have suffered from similar issues: a lack of spatial and temporal resolution (Koenig, 1999).

Perhaps due to the long history of time series use in population cycle literature, many datasets that test for synchrony generally last for an extended period (e.g. 21 years in Huitu et al., 2003). However, even in long‐term datasets, the temporal resolution can be severely limited. For instance Sundell et al. (2004) used the annual breeding output of raptors in 50 km x 50 km areas across Finland as a vole abundance index to characterise synchrony across the country. While such datasets are likely able to determine if true asynchrony or true synchrony is better supported (e.g. peaks occur in the same year), they seem ill‐suited for detecting more subtle forms of synchrony as any signal of a within year spatio‐temporal delay in synchrony (e.g. a travelling wave) would be obscured.

While such issues surrounding temporal resolution and aggregation may mask more subtle forms of synchrony, such as travelling waves, a lack of spatial resolution is perhaps equally detrimental. Indeed, in many instances, population synchrony has been characterised using far fewer spatial replicates than those used in this analysis (Huitu et al., 2003; Huitu et al., 2008; Lambin et al., 2006). In such cases of comparatively low spatial resolution, as with studies with a low temporal resolution, the result may be an ability to distinguish between the two extremes of synchrony but an inability to explore where a metapopulation exists on the spectrum of synchrony.

Indeed, whenever spatio‐temporal datasets have been rich in both spatial and temporal resolution, the outcome appears to be the detection of travelling waves, irrespective of the method used (Berthier et al., 2014; Cummings et al., 2004; Grenfell et al., 2013; Johnson et al., 2004; Lambin et al., 1998). Such datasets tend to exist only for species with public health or economic interests, such as pest species (Bjørnstad, 2000), which may partly explain the relatively few examples of travelling waves in the literature compared to detections of apparent true synchrony. However, if the waves captured here do represent activator‐inhibitor dynamics and their dispersal, it is logical to assume that all cyclic systems are synchronised via travelling waves, which are only subsequently modified by the Moran effect (Hugueny, 2006).

### Activator‐inhibitor waves

Given the long history of using activator‐inhibitor systems to model population cycles (e.g. Levin, 1974), as well as the finding that trophic interactions and dispersal promote synchrony, our findings, which suggest the presence of activator and inhibitor travelling waves, provide some measure of consistency between understandings of synchrony and population cycle theory (Bjørnstad, 2000; Bierman et al., 2006). Such activator‐inhibitor travelling waves have previously been detected in cellular biology (Kapustina et al., 2013; Martinet et al., 2017) but not in ecology.

Our results are, to our knowledge, the first instance where a single spatio‐temporal pattern of population cycles has been decomposed into constituent parts, which we propose to represent the influences of activator and inhibitor on vole population growth. Microcosm experiments investigating the effects of dispersal and trophic interactions (and the Moran effect) found that the synchronising effect of dispersal in the presence of predation led to greater synchrony in population cycles of protists (Vasseur & Fox, 2009), suggesting that the two waves here may partly represent the synchronising effects of dispersal of voles, dispersal of inhibitors (possibly pathogens or predators) and the interactions between them. Indeed, a potential candidate agent for an inhibitor, pathogens, are known to spread via travelling waves (Cummings et al., 2004; Grenfell et al., 2001).

The presence of two epicentres is in line with previous research on travelling waves (Johnson et al., 2004), though the finding that the final cumulative pattern is dependent on both epicentres, with apparently distinct roles (i.e. activation and inhibition of growth rates, Figure 3) is new to ecology. The positioning of the epicentres, estimated as distinct locations (Figure 2), may support the interpretation of activator and inhibitor dynamics. The estimated location of the inhibitor epicentre is in an area with higher‐than‐average abundances of voles (*Tierra de Campos*, see Figure S2). This region is known locally to practitioners for recurrently experiencing severe outbreaks, which may be related to farming practices (Roos et al., 2019). Such a location would present an area consistent with understandings of where travelling waves of diseases initiate, as pathogen waves have been found to originate in areas of high density (Cummings et al., 2004; Grenfell et al., 2001). If so, this epicentre may represent the starting location for the outward spread of pathogens because of infected dispersing individuals, which inhibit the growth rates of voles. A testable hypothesis would be that this region experiences a higher proportion of infected individuals than a regional average. Indeed, two pathogens, Tularemia (*Francisella tularensis*) and *Bartonella* sp. are known to occur in a density‐dependent relationship with vole densities in Tierra de Campos (Rodríguez‐Pastor et al., 2017). As a consequence of being reliant on dispersers for the propagation of the disease, in combination with various delaying processes (e.g. latency to infection), we would expect the speed of the inhibitor wave to be slower than the activator, which we observe (Table 2).

Conversely, the activator epicentre was located in a lower‐than‐average abundance region (see Figure S2). We propose that this may be explained by a slight adjustment to the epicentre hypothesis described in Johnson et al. (2006). The epicentre hypothesis posits that emigration between suitable close habitats causes travelling waves. We consider that our epicentre meets these requirements in all but ‘suitable habitat’ (i.e. lower‐than‐average densities). However, given the high reproductive capacity of common voles, we would assume they can produce as many offspring in a ‘less‐suitable habitat’ as elsewhere in the region, but most of these offspring become emigrants. In this light, the core understanding of the epicentre hypothesis is maintained, where an epicentre is a location producing many emigrants but altering it to consider the reproductive ability of common voles for which there is no evidence of any spatial variation in CyL. Evidence of this would come from finding a higher‐than‐average proportion of dispersers at this location.

The speed of the inhibitor wave was estimated at 147 km per year, while the activator was estimated at 835 km per year, which appear to be middle‐ground speed estimates amongst empirical travelling wave literature (which vary from a minimum of 7–8 km per year [Berthier et al., 2014] to a maximal 1776 km per year [Cummings et al., 2004]). Differences in speed offer some confirmation with simulations (Johnson et al., 2006), where differences in activator‐inhibitor dispersal abilities resulted in radial travelling waves. We propose that pathogen (i.e. possible inhibitors) diffusion would be dependent on host dispersal, mode of transmission, latency to infection, and so forth, all possible means to impart a delay in the spread to adjoining populations. Conversely, we posit that the fast speed of the activator wave may reflect the relative ease at which voles can disperse (i.e. habitat connectivity, where field margins criss‐cross CyL) or the effectiveness of a dispersal event (related to density).

Our modelling has demonstrated evidence for both activator and inhibitor influences on population growth rates in voles. Further work is required to establish the processes underlying these influences and collect sufficient large‐scale data on other ecological systems to establish whether these too are underpinned by activator and inhibitor influences.

## AUTHOR CONTRIBUTIONS

**Deon Roos**: Conceptualisation, Methodology, Software, Validation, Formal analysis, Data Curation, Writing ‐ Original Draft, Writing ‐ Review & Editing, Visualisation **Constantino Caminero‐Saldaña**: Conceptualisation, Methodology, Investigation, Resources, Data Curation, Writing ‐ Review & Editing, Supervision, Project administration, Funding acquisition **David Elston**: Conceptualisation, Methodology, Validation, Formal analysis, Writing ‐ Review & Editing, Supervision **François Mougeot**: Conceptualisation, Writing ‐ Review & Editing, Supervision **María Carmen García‐Ariza**: Methodology, Investigation, Data Curation **Beatriz Arroyo**: Conceptualisation, Writing ‐ Review & Editing, Supervision **Juan José Luque‐Larena**: Conceptualisation, Resources, Writing ‐ Review & Editing, Supervision **Francisco Javier Rojo Revilla**: Methodology, Investigation, Data Curation **Xavier Lambin**: Conceptualisation, Validation, Resources, Writing ‐ Review & Editing, Supervision, Project administration, Funding acquisition.

### PEER REVIEW

The peer review history for this article is available at https://publons.com/publon/10.1111/ele.14074.

## Supporting information


Figure S1

Supinfo S1

Supinfo S2

Supinfo S3

Video S1

## Data Availability

A publicly available data repository is available at https://doi.org/10.5281/zenodo.6460815, which contains the data used in this analysis.
