# Supplementary material for: From pattern to process? Dual travelling waves, with contrasting propagation speeds, best describe a self‐organised spatio‐temporal pattern in population growth of a cyclic rodent
Source: Ecol Lett. 2022 Jul 31;25(9):1986–98. doi: 10.1111/ele.14074 (PMC9543711; doi:10.1111/ele.14074)
Supplement: Supplementary file 1 — Figure S1 [file ELE-25-1986-s003.docx]

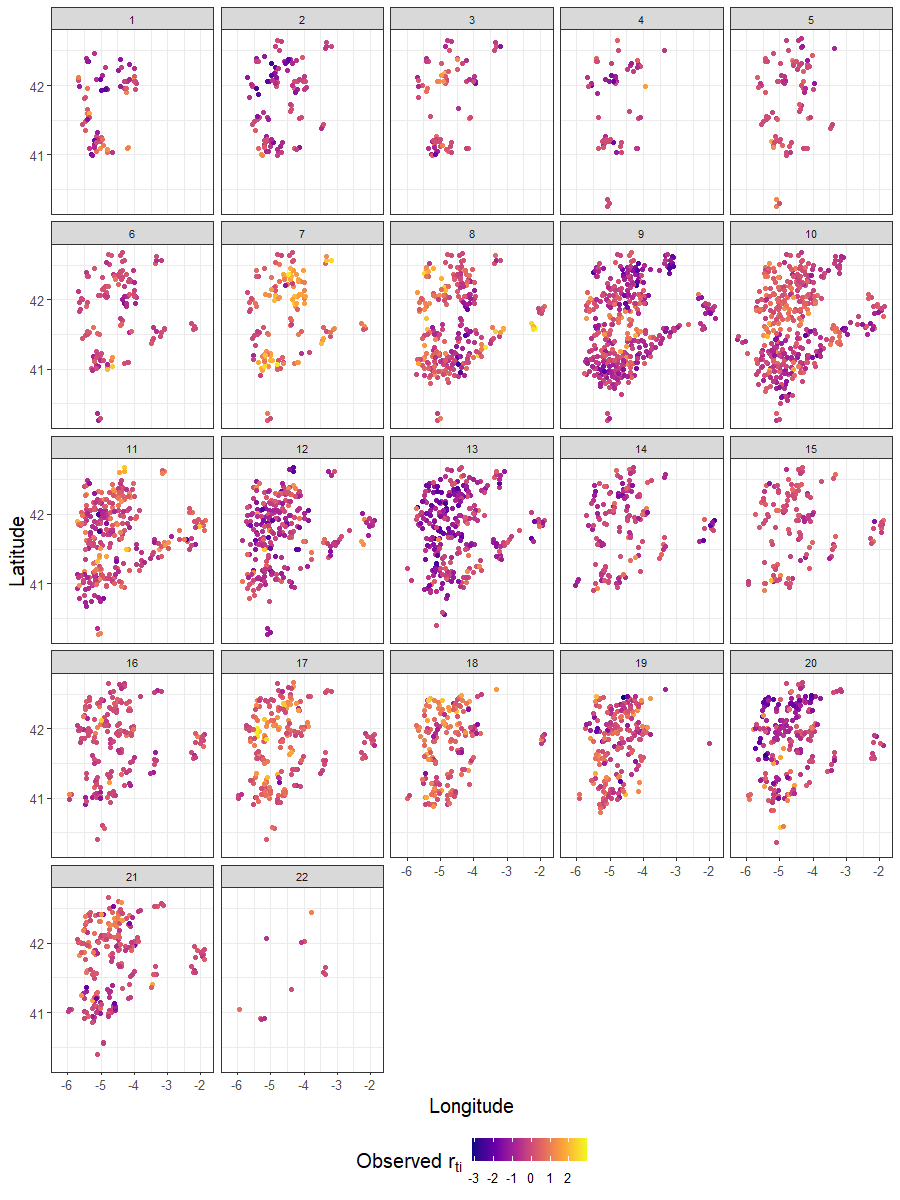


**SFigure 1:** Observed growth rates of centroids following space-time aggregation. Longitude and latitude are on the x and y-axes, respectively, with points coloured according to observed $r_{t}$. Each facet is a yearly quarter.


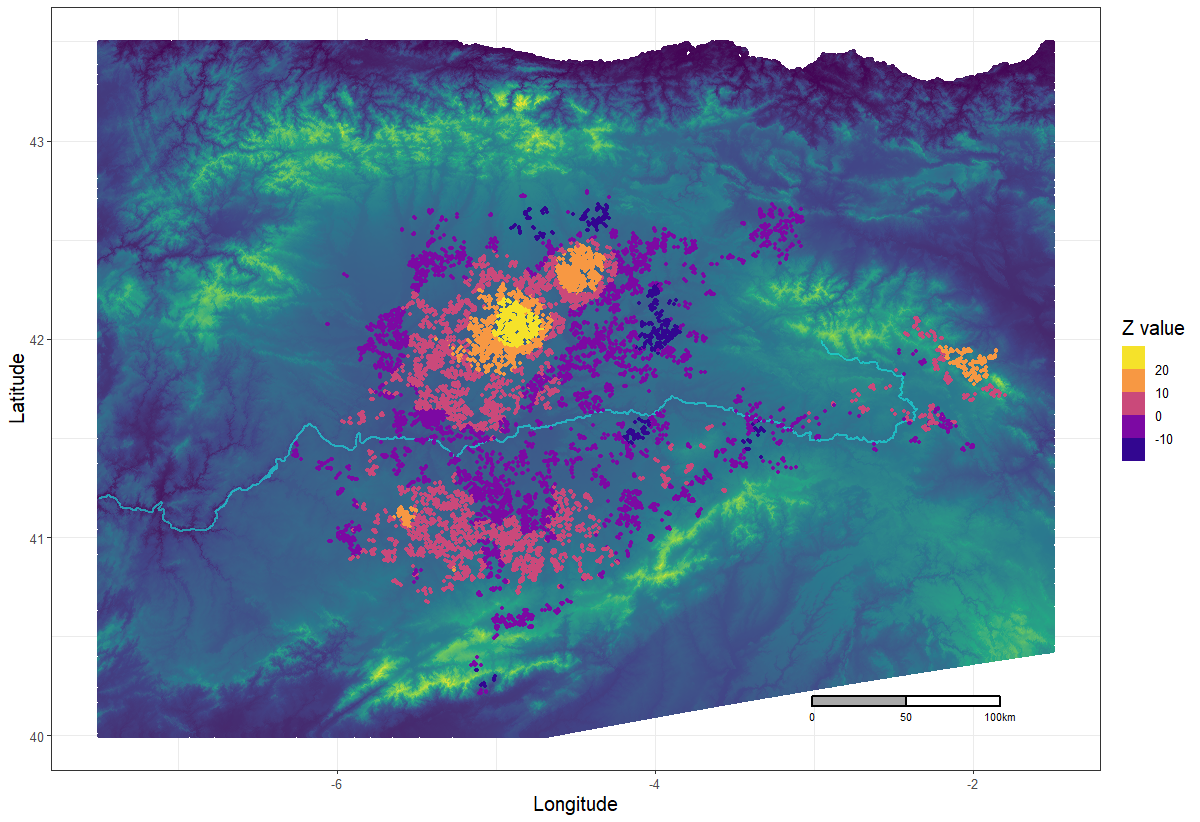


**SFigure 2:** Results from a local G_i_^*^ descriptive analysis of abundance indices from field margins, using a neighbour distance of 15 km. Elevation coloured as per Fig. 4, with points coloured according to the G_i_^*^ Z value, with positive values indicating areas with higher than (global) average abundance (i.e. hot spots), and negative values indicating areas with lower than (global) average abundance (i.e. cold spots). The G_i_^*^ analysis used all transects across time ($n = 42,973$).


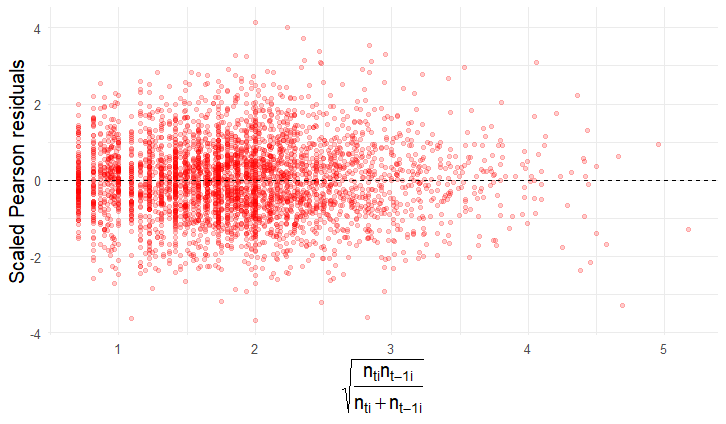


**SFigure 3:** Scaled Pearson residuals against the weighting term ($\mathcal{w}_{t,\mathcal{i}}$) with little evidence of heteroscedasticity across different values of $\sqrt{\frac{n_{t,i}\times n_{t-1,i}}{n_{t,i}+ n_{t-1,i}}}$.
