# Supplementary material for: From pattern to process? Dual travelling waves, with contrasting propagation speeds, best describe a self‐organised spatio‐temporal pattern in population growth of a cyclic rodent
Source: Ecol Lett. 2022 Jul 31;25(9):1986–98. doi: 10.1111/ele.14074 (PMC9543711; doi:10.1111/ele.14074)
Supplement: Supplementary file 2 — Supinfo S1 [file ELE-25-1986-s002.pdf]

Roos et al., Travelling wave supplementary material

Deon Roos et al.

Last run of 2021-03-29

Contents

Dataset and libraries **3**

Number of iterations **3**

Null models **4**

N1 . . . 4
N2 . . . 4
N3 . . . 4

Functions for each wave **4**

RE: Single expanding radial wave . . . 4
RC: Single contracting radial wave . . . 5
P: Single planar wave . . . 5
RFE: Two isolated expanding radial waves . . . 5
RDE: Two interacting activator-inhibitor radial waves . . . 6
RFC: Two isolated contracting radial waves . . . 7
RDC: Two interacting activator-inhibitor contracting radial waves . . . 7
PF: Two isolated planar waves . . . 8
PD: Two interacting activator-inhibitor planar waves . . . 8

Optimisations **9**

RE . . . 9
RC . . . 9
P . . . 9
RFE . . . 9
RDE . . . 10
RFC . . . 10
RDC . . . 10
PF . . . 11
PD . . . 11

Wave estimates & CI 11
RE . . . 11
RC . . . 13
P . . . 14
RFE . . . 16
RDE . . . 19
RFC . . . 22
RDC . . . 25
PF . . . 28
PD . . . 31

Figures 33
Optimisation figures . . . 33
RE . . . 33
RC . . . 35
P . . . 37
RFE . . . 39
RDE . . . 42
RFC . . . 45
RDC . . . 48
PF . . . 51
PD . . . 55
Predictions . . . 59
N2 . . . 59
N3 . . . 60
P . . . 61
RE . . . 63
RC . . . 66
RFE . . . 68
RDE . . . 71
RFC . . . 73
RDC . . . 76
PF . . . 79
PD . . . 81

AIC 84

Total run time 85

Saving environment **85**

System info **85**

Below are runs of optimisers for each of the travelling wave models. Included is:

1. The functions (i.e. the full models)
2. How the optimisers were run
3. Estimates and uncertainty of test runs
4. Figures to show optimisation process for SANN
5. Temporal and spatio-temporal predictions based on best fits from test runs
6. AIC of the best fit models

Dataset and libraries

Data from ITACyL:

```
space <- read.csv("C:\\Users\\r01dr16\\Desktop\\Travelling wave\\vole_TW_publication_data_roos_et_al.cs
space$sqrt_diff_survey <- sqrt(space$differential.survey)
```

Packages used:

```
library(mgcv)      # for gams
library(beepr)     # beeps when optimisation is done
library(emdbook)   # Bolker's package for stochastic annealing optimiser method
library(ggplot2)   # For visualisation
library(gganimate) # For animations
library(scales)    # For comma scales
library(patchwork) # For plotting side-by-side
library(dplyr)     # For sample_n
```

Setting seed:

```
set.seed(2020)
```

Number of iterations

Set for all simulated annealing optimisers.

10,000 used for full fit.

```
N <- 15000
```

Null models

N1

```
N1 <- gam(r.growth ~ 1,
          weights = sqrt_diff_survey,
          method = "ML",
          family = "gaussian",
          data = space)
```

N2

```
N2 <- gam(r.growth ~ s(julian.mean.trans, k = 12, bs = "tp"),
          weights = sqrt_diff_survey,
          method = "ML",
          family = "gaussian",
          data = space)
```

N3

```
N3 <- gam(r.growth ~ te(cen.x, cen.y, k = c(10, 10), bs = "tp"),
          weights = sqrt_diff_survey,
          method = "ML",
          family = "gaussian",
          data = space)
```

Functions for each wave

All functions include the arguments, `dataset = space`, specifying the dataset to use in the `mgcv::gam` function, as well as a vector `par = par_list_x` specifying parameters to be estimated.

Wave models all use stochastic/simulated annealing (SANN) optimiser.

RE: Single expanding radial wave

```
RE_TW_fun <- function(par, data = space) {
  space$D <- -sqrt((par[1] - space$cen.x)^2 + (par[2] - space$cen.y)^2)
  space$rho <- space$julian.mean.trans + (1 / par[3]) * space$D

  -logLik.gam(gam(r.growth ~ s(rho, k = 12, bs = "tp"),
                  weights = sqrt_diff_survey,
                  method = "ML",
                  data = space,
                  family = "gaussian")
  )[1]
}
```

With initial values:

```
par_list_RE <- c(4508903.843, -5258566.702, 4772.709)
```

RC: Single contracting radial wave

```
RC_TW_fun <- function(par, data = space) {
  space$D <- sqrt((par[1] - space$cen.x)^2 + (par[2] - space$cen.y)^2)
  space$rho <- space$julian.mean.trans + (1 / par[3]) * space$D

  -logLik.gam(gam(r.growth ~ s(rho, k = 12, bs = "tp"),
                  weights = sqrt_diff_survey,
                  method = "ML",
                  data = space,
                  family = "gaussian")
  )[1]
}
```

With initial values:

```
par_list_RC <- c(699241.424265928, -665819.57, 4910.105)
```

P: Single planar wave

```
single_planar <- function(par, dataset = space) {

  space$D <- sin(par[1]) * space$cen.x + cos(par[1]) * space$cen.y
  space$rho <- space$julian.mean.trans + (1 / par[2]) * space$D

  -logLik.gam(gam(r.growth ~ s(rho, k = 12, bs = "tp"),
                  weights = sqrt_diff_survey,
                  method = "ML",
                  family = "gaussian",
                  data = space)
  )[1]
}
```

With initial values:

```
par_list_P_TW <- c(5.6, 4678)
```

RFE: Two isolated expanding radial waves

North south dummy variables

```
space$north <- NA
space$south <- NA

space$north <- ifelse(space$north.south == "north", 1, ifelse(space$north.south == "south", 0, NA))
space$south <- ifelse(space$north.south == "south", 1, ifelse(space$north.south == "north", 0, NA))
```

Function:

```
RFE_fun <- function(par, data = space) {
  # Loop with if to calculate euclidean distance from north/south "epicentres" & convert to time
  for(i in 1:nrow(space)){
    if (space$north.south[i] == "north") {
      space$D1[i] <- -sqrt((par[1] - space$cen.x[i])^2 + (par[2] - space$cen.y[i])^2)
      space$rho1[i] <- space$julian.mean.trans[i] + (1 / par[3]) * space$D1[i]
    } else {
      space$D2[i] <- -sqrt((par[4] - space$cen.x[i])^2 + (par[5] - space$cen.y[i])^2)
      space$rho2[i] <- space$julian.mean.trans[i] + (1 / par[6]) * space$D2[i]
    }
  }

  -logLik.gam(gam(r.growth ~ s(rho1, by = north, k = 12, bs = "tp") + s(rho2, by = south, k = 12, bs =
                  weights = sqrt_diff_survey,
                  method = "ML",
                  data = space,
                  family = "gaussian")
  )[1]
}
```

With initial values:

```
par_list_RFE <- c(-188786.998, 130207.775, 12883.387,
                  -1922.858, 37086.704, 1030.109)
```

RDE: Two interacting activator-inhibitor radial waves

```
RDE_fun <- function(par, data = space) {
  space$D1 <- -sqrt((par[1] - space$cen.x)^2 + (par[2] - space$cen.y)^2)
  space$rho1 <- space$julian.mean.trans + (1 / par[3]) * space$D1

  space$D2 <- -sqrt((par[4] - space$cen.x)^2 + (par[5] - space$cen.y)^2)
  space$rho2 <- space$julian.mean.trans + (1 / par[6]) * space$D2

  -logLik.gam(gam(r.growth ~ s(rho1, k = 12, bs = "tp") + s(rho2, k = 12, bs = "tp"),
                  weights = sqrt_diff_survey,
                  method = "ML",
                  data = space,
                  family = "gaussian")
  )[1]
}
```

With initial values;

```
par_list_RDE <- c(-32406.318, 5611.767, 2261.920,
                  29532.097, -59547.747, 3169.842)
```

RFC: Two isolated contracting radial waves

North south dummy variables

```
space$north <- NA
space$south <- NA

space$north <- ifelse(space$north.south == "north", 1, ifelse(space$north.south == "south", 0, NA))
space$south <- ifelse(space$north.south == "south", 1, ifelse(space$north.south == "north", 0, NA))
```

Function:

```
RFC_fun <- function(par, data = space) {
  # Loop with if to calculate euclidean distance from north/south "epicentres" & convert to time
  for(i in 1:nrow(space)){
    if (space$north.south[i] == "north") {
      space$D1[i] <- sqrt((par[1] - space$cen.x[i])^2 + (par[2] - space$cen.y[i])^2)
      space$rho1[i] <- space$julian.mean.trans[i] + (1 / par[3]) * space$D1[i]
    } else {
      space$D2[i] <- sqrt((par[4] - space$cen.x[i])^2 + (par[5] - space$cen.y[i])^2)
      space$rho2[i] <- space$julian.mean.trans[i] + (1 / par[6]) * space$D2[i]
    }
  }

  -logLik.gam(gam(r.growth ~ s(rho1, by = north, k = 12, bs = "tp") + s(rho2, by = south, k = 12, bs =
                  weights = sqrt_diff_survey,
                  method = "ML",
                  data = space,
                  family = "gaussian")
  )[1]
}
```

With initial values:

```
par_list_RFC <- c(158385.3, 90449.47, 4500,
                  139750.6,  -474535.87, 4500)
```

RDC: Two interacting activator-inhibitor contracting radial waves

```
RDC_fun <- function(par, data = space) {
  space$D1 <- sqrt((par[1] - space$cen.x)^2 + (par[2] - space$cen.y)^2)
  space$rho1 <- space$julian.mean.trans + (1 / par[3]) * space$D1

  space$D2 <- sqrt((par[4] - space$cen.x)^2 + (par[5] - space$cen.y)^2)
  space$rho2 <- space$julian.mean.trans + (1 / par[6]) * space$D2
```

```
  -logLik.gam(gam(r.growth ~ s(rho1, k = 12, bs = "tp") + s(rho2, k = 12, bs = "tp"),
                  weights = sqrt_diff_survey,
                  method = "ML",
                  data = space,
                  family = "gaussian")
  )[1]
}
```

With initial values;

```
par_list_RDC <- c(-30730.33, 5165.24, 14286.43,
                  -55296.03, -91968.48, 42857.29)
```

PF: Two isolated planar waves

```
PF_fun <- function(par, data = space) {
  # Loop with if to calculate distance from north/south planar waves & convert to time
  for(i in 1:nrow(space)){
    if (space$north.south[i] == "north") {
      space$D1[i] <- sin(par[1]) * space$cen.x[i] + cos(par[1]) * space$cen.y[i]
      space$rho1[i] <- space$julian.mean.trans[i] + (1 / par[2]) * space$D1[i]
    } else {
      space$D2[i] <- sin(par[3]) * space$cen.x[i] + cos(par[3]) * space$cen.y[i]
      space$rho2[i] <- space$julian.mean.trans[i] + (1 / par[4]) * space$D2[i]
    }
  }

  -logLik.gam(gam(r.growth ~ s(rho1, by = north, k = 12, bs = "tp") + s(rho2, by = south, k = 12, bs =
                  weights = sqrt_diff_survey,
                  method = "ML",
                  data = space,
                  family = "gaussian")
  )[1]
}
```

With initial values;

```
par_list_PF <- c(3.707079, 5000,
                 5.843362, 2250.95)
```

PD: Two interacting activator-inhibitor planar waves

```
PD_fun <- function(par, data = space) {
  space$D1 <- sin(par[1]) * space$cen.x + cos(par[1]) * space$cen.y
  space$rho1 <- space$julian.mean.trans + (1 / par[2]) * space$D1

  space$D2 <- sin(par[3]) * space$cen.x + cos(par[3]) * space$cen.y
  space$rho2 <- space$julian.mean.trans + (1 / par[4]) * space$D2
```

```
  -logLik.gam(gam(r.growth ~ s(rho1, k = 12, bs = "tp") + s(rho2, k = 12, bs = "tp"),
                  weights = sqrt_diff_survey,
                  method = "ML",
                  data = space,
                  family = "gaussian")
  )[1]
}
```

With initial values:

```
par_list_PD <- c(3.6, 3725,
                 6.0, 4657)
```

Optimisations

RE

```
RE_out <- metropSB(fn = RE_TW_fun,
                   start = par_list_RE,
                   nmax = N,
                   retvals = TRUE,
                   retfreq = 1,
                   verbose = FALSE)
```

RC

```
RC_out <- metropSB(fn = RC_TW_fun,
                   start = par_list_RC,
                   nmax = N,
                   retvals = TRUE,
                   retfreq = 1,
                   verbose = FALSE)
```

P

```
P_out <- metropSB(fn = single_planar,
                  start = par_list_P_TW,
                  nmax = N,
                  retvals = TRUE,
                  retfreq = 1,
                  verbose = FALSE)
```

RFE

```r
# "Empty" vectors to allow gam to run - unused due to dummy variables
space$D1 <- 1
space$rho1 <- 1
space$D2 <- 1
space$rho2 <- 1

RFE_out <- metropSB(fn = RFE_fun,
                    start = par_list_RFE,
                    nmax = N,
                    retvals = TRUE,
                    retfreq = 1,
                    verbose = FALSE)
```

RDE

```r
RDE_out <- metropSB(fn = RDE_fun,
                    start = par_list_RDE,
                    nmax = N,
                    retvals = TRUE,
                    retfreq = 1,
                    verbose = FALSE)
```

RFC

```r
# "Empty" vectors to allow gam to run - unused due to dummy variables
space$D1 <- 1
space$rho1 <- 1
space$D2 <- 1
space$rho2 <- 1

RFC_out <- metropSB(fn = RFC_fun,
                    start = par_list_RFC,
                    nmax = N,
                    retvals = TRUE,
                    retfreq = 1,
                    verbose = FALSE)
```

RDC

```r
RDC_out <- metropSB(fn = RDC_fun,
                    start = par_list_RDC,
                    nmax = N,
                    retvals = TRUE,
                    retfreq = 1,
                    verbose = FALSE)
```

PF

```
# "Empty" vectors to allow gam to run - unused due to dummy variables
space$D1 <- 1
space$rho1 <- 1
space$D2 <- 1
space$rho2 <- 1

PF_out <- metropSB(fn = PF_fun,
                   start = par_list_PF,
                   nmax = N,
                   retvals = TRUE,
                   retfreq = 1,
                   verbose = FALSE)
```

PD

```
# "Empty" vectors to allow gam to run - unused due to dummy variables
space$D1 <- 1
space$rho1 <- 1
space$D2 <- 1
space$rho2 <- 1

PD_out <- metropSB(fn = PD_fun,
                   start = par_list_PD,
                   nmax = N,
                   retvals = TRUE,
                   retfreq = 1,
                   verbose = FALSE)
```

Wave estimates & CI

RE

```
RE_out_df <- data.frame(RE_out$retvals[,c(1:3, 10)])
RE_out_df$val <- 2*RE_out_df$val # convert to -2loglikelihood

RE_profile <- data.frame(
  p1 = NA,
  p2 = NA,
  p3 = NA,
  val = NA
)

for(i in 1:length(unique(RE_out_df$p3))) {           # For each unique value of speed
  match <- unique(RE_out_df$p3)                      # Store all unique speeds
  temp <- subset(RE_out_df, p3 == match[i])          # Subset all iterations from speed i to temporary
  temp1 <- temp[temp$val == max(temp$val),]          # Within temp df, extract value where L is maximis
```

```
  RE_profile <- rbind(RE_profile, temp1)              # Store in empty df
}

RE_profile <- RE_profile[-1,]                         # Remove NA row used to create empty df

# Find Lmin
lnLmax <- RE_profile$val[RE_profile$val == min(RE_profile$val)]

# Calc delta -2lnL
RE_profile$lnL_diff <- RE_profile$val - lnLmax

# Subset by 95% CI by chisq dist
p3ci_profile_df <- RE_profile[RE_profile$lnL_diff < qchisq(p = 0.95, df = 1),]

# Report range
p3min <- min(p3ci_profile_df$p3)
p3max <- max(p3ci_profile_df$p3)

# Centroid
RE_out_df$centroid <- paste(RE_out_df$p1, RE_out_df$p2, sep = " ")

RE_profile <- data.frame(
  p1= NA,
  p2 = NA,
  p3 = NA,
  val = NA,
  centroid = NA
)

for(i in 1:length(unique(RE_out_df$centroid))) { # For each unique centroid location
  match <- unique(RE_out_df$centroid)            # Store all unique speeds
  temp <- subset(RE_out_df, centroid == match[i])# Subset all iterations from speed i to temporary df
  temp1 <- temp[temp$val == max(temp$val),]            # Within temp df, extract value where L is maxim

  RE_profile <- rbind(RE_profile, temp1)            # Store in empty df
}

RE_profile <- RE_profile[-1,]                       # Remove NA row used to create empty df

lnLmax <- RE_profile$val[RE_profile$val == min(RE_profile$val)]
RE_profile$lnL_diff <- RE_profile$val - lnLmax

ci_profile_df <- RE_profile[RE_profile$lnL_diff < qchisq(p = 0.95, df = 2),]

# Report range for centroid location
p1min <- min(ci_profile_df$p1)
p1max <- max(ci_profile_df$p1)

p2min <- min(ci_profile_df$p2)
p2max <- max(ci_profile_df$p2)

RE_est <- data.frame(
  Parameter = c("Epicentre X", "Epicentre Y", "Speed"),
```

```r
  Units = c("mean centred UTM", "mean centred UTM", "km per day"),
  Estimate = comma(RE_out$estimate),
  Lower95CI = comma(c(p1min, p2min, p3min)),
  Upper95CI = comma(c(p1max, p2max, p3max))
)
RE_est
```

Parameter	Units	Estimate	Lower95CI	Upper95CI
Epicentre X	mean centred UTM	-16,125,486	-325,023,098	-3,914,729
Epicentre Y	mean centred UTM	18,375,362	2,264,934	417,879,520
Speed	km per day	4,628	3,458	8,029

RC

```r
RC_out_df <- data.frame(RC_out$retvals[,c(1:3, 10)])
RC_out_df$val <- 2*RC_out_df$val # convert to -2loglikelihood

RC_profile <- data.frame(
  p1 = NA,
  p2 = NA,
  p3 = NA,
  val = NA
)

for(i in 1:length(unique(RC_out_df$p3))) {           # For each unique value of speed
  match <- unique(RC_out_df$p3)                       # Store all unique speeds
  temp <- subset(RC_out_df, p3 == match[i])           # Subset all iterations from speed i to temporary
  temp1 <- temp[temp$val == max(temp$val),]           # Within temp df, extract value where L is maximis

  RC_profile <- rbind(RC_profile, temp1)              # Store in empty df
}

RC_profile <- RC_profile[-1,]                         # Remove NA row used to create empty df

# Find Lmin
lnLmax <- RC_profile$val[RC_profile$val == min(RC_profile$val)]

# Calc delta -2lnL
RC_profile$lnL_diff <- RC_profile$val - lnLmax

# Subset by 95% CI by chisq dist
p3ci_profile_df <- RC_profile[RC_profile$lnL_diff < qchisq(p = 0.95, df = 1),]

# Report range
p3min <- min(p3ci_profile_df$p3)
p3max <- max(p3ci_profile_df$p3)

# Centroid
RC_out_df$centroid <- paste(RC_out_df$p1, RC_out_df$p2, sep = " ")
```

```
RC_profile <- data.frame(
  p1= NA,
  p2 = NA,
  p3 = NA,
  val = NA,
  centroid = NA
)

for(i in 1:length(unique(RC_out_df$centroid))) { # For each unique centroid location
  match <- unique(RC_out_df$centroid)            # Store all unique speeds
  temp <- subset(RC_out_df, centroid == match[i])# Subset all iterations from speed i to temporary df
  temp1 <- temp[temp$val == max(temp$val),]            # Within temp df, extract value where L is maxim

  RC_profile <- rbind(RC_profile, temp1)           # Store in empty df
}

RC_profile <- RC_profile[-1,]                      # Remove NA row used to create empty df

lnLmax <- RC_profile$val[RC_profile$val == min(RC_profile$val)]
RC_profile$lnL_diff <- RC_profile$val - lnLmax

ci_profile_df <- RC_profile[RC_profile$lnL_diff < qchisq(p = 0.95, df = 2),]

# Report range for centroid location
p1min <- min(ci_profile_df$p1)
p1max <- max(ci_profile_df$p1)

p2min <- min(ci_profile_df$p2)
p2max <- max(ci_profile_df$p2)

RC_est <- data.frame(
  Parameter = c("Epicentre X", "Epicentre Y", "Speed"),
  Units = c("mean centred UTM", "mean centred UTM", "km per day"),
  Estimate = comma(RC_out$estimate),
  Lower95CI = comma(c(p1min, p2min, p3min)),
  Upper95CI = comma(c(p1max, p2max, p3max))
)
RC_est
```

Parameter	Units	Estimate	Lower95CI	Upper95CI
Epicentre X	mean centred UTM	41,658,297	419,095	44,203,821
Epicentre Y	mean centred UTM	-45,805,574	-49,877,682	-404,473
Speed	km per day	4,738	3,458	8,022

P

```
P_out_df <- data.frame(P_out$retvals[,c(1:2, 7)])
P_out_df$val <- 2*P_out_df$val # convert to -2loglikelihood

# Angle
```

```
P_profile <- data.frame(
  p1= NA,
  p2 = NA,
  val = NA
)

for(i in 1:length(unique(P_out_df$p1))) {           # For each unique value of angle
  match <- unique(P_out_df$p1)                      # Store all unique angle
  temp <- subset(P_out_df, p1 == match[i])          # Subset all iterations from speed i to temporary d
  temp1 <- temp[temp$val == max(temp$val),]          # Within temp df, extract value where L is maximis

  P_profile <- rbind(P_profile, temp1)             # Store in empty df
}

P_profile <- P_profile[-1,]                        # Remove NA row used to create empty df

# Find Lmin
lnLmax <- P_profile$val[P_profile$val == min(P_profile$val)]

# Calc delta -2lnL
P_profile$lnL_diff <- P_profile$val - lnLmax

# Subset by 95% CI by chisq dist
p1ci_profile_df <- P_profile[P_profile$lnL_diff < qchisq(p = 0.95, df = 1),]

# CI range
p1min <- min(p1ci_profile_df$p1)
p1max <- max(p1ci_profile_df$p1)

# Speed

P_profile <- data.frame(
  p1= NA,
  p2 = NA,
  val = NA
)

for(i in 1:length(unique(P_out_df$p2))) {           # For each unique value of angle
  match <- unique(P_out_df$p2)                      # Store all unique angle
  temp <- subset(P_out_df, p2 == match[i])          # Subset all iterations from speed i to temporary d
  temp1 <- temp[temp$val == max(temp$val),]          # Within temp df, extract value where L is maximis

  P_profile <- rbind(P_profile, temp1)             # Store in empty df
}

P_profile <- P_profile[-1,]                      # Remove NA row used to create empty df

lnLmax <- P_profile$val[P_profile$val == min(P_profile$val)]
P_profile$lnL_diff <- P_profile$val - lnLmax

p2ci_profile_df <- P_profile[P_profile$lnL_diff < qchisq(p = 0.95, df = 1),]

# Report CI range
```

```
p2min <- min(p2ci_profile_df$p2)
p2max <- max(p2ci_profile_df$p2)

P_est <- data.frame(
  Parameter = c("Angle", "Speed"),
  Units = c("radian", "km per day"),
  Estimate = comma(P_out$estimate),
  Lower95CI = comma(c(p1min, p2min)),
  Upper95CI = comma(c(p1max, p2max))
)
P_est
```

Parameter	Units	Estimate	Lower95CI	Upper95CI
Angle	radian	6	5	6
Speed	km per day	4,737	3,463	8,040

RFE

```
RFE_out_df <- data.frame(RFE_out$retvals[,c(1:6, 19)])
RFE_out_df$val <- 2*RFE_out_df$val # convert to -2loglikelihood

#North speed

RFE_profile <- data.frame(  # Empty dataframe to store loop output
  p1 = NA,
  p2 = NA,
  p3 = NA,

  p4 = NA,
  p5 = NA,
  p6 = NA,

  val = NA
)

for(i in 1:length(unique(RFE_out_df$p3))) { # For each unique value of speed
  match <- unique(RFE_out_df$p3)            # Store all unique speeds
  temp <- subset(RFE_out_df, p3 == match[i])# Subset all iterations from speed i to temporary df
  temp1 <- temp[temp$val == max(temp$val),]            # Within temp df, extract value where L is maxim

  RFE_profile <- rbind(RFE_profile, temp1)           # Store in empty df
}

RFE_profile <- RFE_profile[-1,]                      # Remove NA row used to create empty df
lnLmax <- RFE_profile$val[RFE_profile$val == min(RFE_profile$val)]
RFE_profile$lnL_diff <- RFE_profile$val - lnLmax
ci_profile_df <- RFE_profile[RFE_profile$lnL_diff < qchisq(p = 0.95, df = 1),]

p3min <- min(ci_profile_df$p3)
p3max <- max(ci_profile_df$p3)
```

```r
# North epicentre

RFE_out_df$centroid <- paste(RFE_out_df$p1, RFE_out_df$p2, sep = " ")

RFE_profile <- data.frame(  # Empty dataframe to store loop output
  p1 = NA,
  p2 = NA,
  p3 = NA,
  p4 = NA,
  p5 = NA,
  p6 = NA,

  centroid = NA,
  val = NA
)

for(i in 1:length(unique(RFE_out_df$centroid))) { # For each unique centroid location
  match <- unique(RFE_out_df$centroid)            # Store all unique speeds
  temp <- subset(RFE_out_df, centroid == match[i])# Subset all iterations from speed i to temporary df
  temp1 <- temp[temp$val == max(temp$val),]            # Within temp df, extract value where L is maxim

  RFE_profile <- rbind(RFE_profile, temp1)           # Store in empty df
}

RFE_profile <- RFE_profile[-1,]                      # Remove NA row used to create empty df

lnLmax <- RFE_profile$val[RFE_profile$val == min(RFE_profile$val)]
RFE_profile$lnL_diff <- RFE_profile$val - lnLmax
ci_profile_df <- RFE_profile[RFE_profile$lnL_diff < qchisq(p = 0.95, df = 2),]

# Report range for centroid location
p1min <- min(ci_profile_df$p1)
p1max <- max(ci_profile_df$p1)

p2min <- min(ci_profile_df$p2)
p2max <- max(ci_profile_df$p2)

# South speed

RFE_profile <- data.frame(  # Empty dataframe to store loop output
  p1 = NA,
  p2 = NA,
  p3 = NA,

  p4 = NA,
  p5 = NA,
  p6 = NA,

  centroid = NA,
  val = NA
)

for(i in 1:length(unique(RFE_out_df$p6))) { # For each unique value of speed
```

```r
  match <- unique(RFE_out_df$p6)            # Store all unique speeds
  temp <- subset(RFE_out_df, p6 == match[i])# Subset all iterations from speed i to temporary df
  temp1 <- temp[temp$val == max(temp$val),]            # Within temp df, extract value where L is maxim

  RFE_profile <- rbind(RFE_profile, temp1)           # Store in empty df
}

RFE_profile <- RFE_profile[-1,]                      # Remove NA row used to create empty df
lnLmax <- RFE_profile$val[RFE_profile$val == min(RFE_profile$val)]
RFE_profile$lnL_diff <- RFE_profile$val - lnLmax
ci_profile_df <- RFE_profile[RFE_profile$lnL_diff < qchisq(p = 0.95, df = 1),]

p6min <- min(ci_profile_df$p6)
p6max <- max(ci_profile_df$p6)

# South epicentre

RFE_out_df$centroid <- paste(RFE_out_df$p4, RFE_out_df$p5, sep = " ")

RFE_profile <- data.frame(  # Empty dataframe to store loop output
  p1 = NA,
  p2 = NA,
  p3 = NA,

  p4 = NA,
  p5 = NA,
  p6 = NA,

  centroid = NA,
  val = NA
)

for(i in 1:length(unique(RFE_out_df$centroid))) { # For each unique centroid location
  match <- unique(RFE_out_df$centroid)            # Store all unique speeds
  temp <- subset(RFE_out_df, centroid == match[i])# Subset all iterations from speed i to temporary df
  temp1 <- temp[temp$val == max(temp$val),]            # Within temp df, extract value where L is maxim

  RFE_profile <- rbind(RFE_profile, temp1)           # Store in empty df
}

RFE_profile <- RFE_profile[-1,]                      # Remove NA row used to create empty df

lnLmax <- RFE_profile$val[RFE_profile$val == min(RFE_profile$val)]
RFE_profile$lnL_diff <- RFE_profile$val - lnLmax
ci_profile_df <- RFE_profile[RFE_profile$lnL_diff < qchisq(p = 0.95, df = 2),]

# Report range for centroid location
p4min <- min(ci_profile_df$p4)
p4max <- max(ci_profile_df$p4)

p5min <- min(ci_profile_df$p5)
p5max <- max(ci_profile_df$p5)
```

```r
RFE_est <- data.frame(
  Parameter = c("North Epicentre X", "North Epicentre Y", "North Speed",
                "South Epicentre X", "South Epicentre Y", "South Speed"),
  Units = c("mean centred UTM", "mean centred UTM", "km per day",
            "mean centred UTM", "mean centred UTM", "km per day"),
  Estimate = comma(RFE_out$estimate),
  Lower95CI = comma(c(p1min, p2min, p3min, p4min, p5min, p6min)),
  Upper95CI = comma(c(p1max, p2max, p3max, p4max, p5max, p6max))
)
RFE_est
```

Parameter	Units	Estimate	Lower95CI	Upper95CI
North Epicentre X	mean centred UTM	-730,219	-1,901,806	-139,144
North Epicentre Y	mean centred UTM	-313,562	-562,143	241,670
North Speed	km per day	8,210	5,008	22,365
South Epicentre X	mean centred UTM	-9,824	-17,739	2,575
South Epicentre Y	mean centred UTM	27,766	-6,446	70,349
South Speed	km per day	1,140	890	1,501

RDE

```r
RDE_out_df <- data.frame(RDE_out$retvals[,c(1:6, 19)])
RDE_out_df$val <- 2*RDE_out_df$val # convert to -2loglikelihood

# Activator speed

RDE_profile <- data.frame(  # Empty dataframe to store loop output
  p1 = NA,
  p2 = NA,
  p3 = NA,

  p4 = NA,
  p5 = NA,
  p6 = NA,

  val = NA
)

for(i in 1:length(unique(RDE_out_df$p3))) { # For each unique value of speed
  match <- unique(RDE_out_df$p3)            # Store all unique speeds
  temp <- subset(RDE_out_df, p3 == match[i])# Subset all iterations from speed i to temporary df
  temp1 <- temp[temp$val == max(temp$val),]            # Within temp df, extract value where L is maxim

  RDE_profile <- rbind(RDE_profile, temp1)           # Store in empty df
}

RDE_profile <- RDE_profile[-1,]                       # Remove NA row used to create empty df
lnLmax <- RDE_profile$val[RDE_profile$val == min(RDE_profile$val)]
RDE_profile$lnL_diff <- RDE_profile$val - lnLmax
ci_profile_df <- RDE_profile[RDE_profile$lnL_diff < qchisq(p = 0.95, df = 1),]
```

```
p3min <- min(ci_profile_df$p3)
p3max <- max(ci_profile_df$p3)

# Activator epicentre

RDE_out_df$centroid <- paste(RDE_out_df$p1, RDE_out_df$p2, sep = " ")

RDE_profile <- data.frame(  # Empty dataframe to store loop output
  p1 = NA,
  p2 = NA,
  p3 = NA,
  p4 = NA,
  p5 = NA,
  p6 = NA,

  centroid = NA,
  val = NA
)

for(i in 1:length(unique(RDE_out_df$centroid))) { # For each unique centroid location
  match <- unique(RDE_out_df$centroid)            # Store all unique speeds
  temp <- subset(RDE_out_df, centroid == match[i])# Subset all iterations from speed i to temporary df
  temp1 <- temp[temp$val == max(temp$val),]            # Within temp df, extract value where L is maxim

  RDE_profile <- rbind(RDE_profile, temp1)           # Store in empty df
}

RDE_profile <- RDE_profile[-1,]                      # Remove NA row used to create empty df

lnLmax <- RDE_profile$val[RDE_profile$val == min(RDE_profile$val)]
RDE_profile$lnL_diff <- RDE_profile$val - lnLmax
ci_profile_df <- RDE_profile[RDE_profile$lnL_diff < qchisq(p = 0.95, df = 2),]

# Report range for centroid location
p1min <- min(ci_profile_df$p1)
p1max <- max(ci_profile_df$p1)

p2min <- min(ci_profile_df$p2)
p2max <- max(ci_profile_df$p2)

# Inhibitor speed

RDE_profile <- data.frame(  # Empty dataframe to store loop output
  p1 = NA,
  p2 = NA,
  p3 = NA,

  p4 = NA,
  p5 = NA,
  p6 = NA,

  centroid = NA,
  val = NA
```

```
)

for(i in 1:length(unique(RDE_out_df$p6))) { # For each unique value of speed
  match <- unique(RDE_out_df$p6)            # Store all unique speeds
  temp <- subset(RDE_out_df, p6 == match[i])# Subset all iterations from speed i to temporary df
  temp1 <- temp[temp$val == max(temp$val),]            # Within temp df, extract value where L is maxim

  RDE_profile <- rbind(RDE_profile, temp1)           # Store in empty df
}

RDE_profile <- RDE_profile[-1,]                      # Remove NA row used to create empty df
lnLmax <- RDE_profile$val[RDE_profile$val == min(RDE_profile$val)]
RDE_profile$lnL_diff <- RDE_profile$val - lnLmax
ci_profile_df <- RDE_profile[RDE_profile$lnL_diff < qchisq(p = 0.95, df = 1),]

p6min <- min(ci_profile_df$p6)
p6max <- max(ci_profile_df$p6)

# Inhibitor epicentre

RDE_out_df$centroid <- paste(RDE_out_df$p4, RDE_out_df$p5, sep = " ")

RDE_profile <- data.frame(  # Empty dataframe to store loop output
  p1 = NA,
  p2 = NA,
  p3 = NA,

  p4 = NA,
  p5 = NA,
  p6 = NA,

  centroid = NA,
  val = NA
)

for(i in 1:length(unique(RDE_out_df$centroid))) { # For each unique centroid location
  match <- unique(RDE_out_df$centroid)            # Store all unique speeds
  temp <- subset(RDE_out_df, centroid == match[i])# Subset all iterations from speed i to temporary df
  temp1 <- temp[temp$val == max(temp$val),]            # Within temp df, extract value where L is maxim

  RDE_profile <- rbind(RDE_profile, temp1)           # Store in empty df
}

RDE_profile <- RDE_profile[-1,]                      # Remove NA row used to create empty df

lnLmax <- RDE_profile$val[RDE_profile$val == min(RDE_profile$val)]
RDE_profile$lnL_diff <- RDE_profile$val - lnLmax
ci_profile_df <- RDE_profile[RDE_profile$lnL_diff < qchisq(p = 0.95, df = 2),]

# Report range for centroid location
p4min <- min(ci_profile_df$p4)
p4max <- max(ci_profile_df$p4)
```

```r
p5min <- min(ci_profile_df$p5)
p5max <- max(ci_profile_df$p5)

RDE_est <- data.frame(
  Parameter = c("Activator Epicentre X", "Activator Epicentre Y", "Activator Speed",
                "Inhibitor Epicentre X", "Inhibitor Epicentre Y", "Inhibitor Speed"),
  Units = c("mean centred UTM", "mean centred UTM", "km per day",
            "mean centred UTM", "mean centred UTM", "km per day"),
  Estimate = comma(RDE_out$estimate),
  Lower95CI = comma(c(p1min, p2min, p3min, p4min, p5min, p6min)),
  Upper95CI = comma(c(p1max, p2max, p3max, p4max, p5max, p6max))
)
RDE_est
```

Parameter	Units	Estimate	Lower95CI	Upper95CI
Activator Epicentre X	mean centred UTM	-41,723	-51,645	-33,626
Activator Epicentre Y	mean centred UTM	28,414	18,556	29,897
Activator Speed	km per day	405	316	528
Inhibitor Epicentre X	mean centred UTM	23,675	5,161	42,607
Inhibitor Epicentre Y	mean centred UTM	-8,675	-21,536	11,351
Inhibitor Speed	km per day	2,287	1,783	2,941

RFC

```r
RFC_out_df <- data.frame(RFC_out$retvals[,c(1:6, 19)])
RFC_out_df$val <- 2*RFC_out_df$val # convert to -2loglikelihood

#North speed

RFC_profile <- data.frame(  # Empty dataframe to store loop output
  p1 = NA,
  p2 = NA,
  p3 = NA,

  p4 = NA,
  p5 = NA,
  p6 = NA,

  val = NA
)

for(i in 1:length(unique(RFC_out_df$p3))) { # For each unique value of speed
  match <- unique(RFC_out_df$p3)            # Store all unique speeds
  temp <- subset(RFC_out_df, p3 == match[i])# Subset all iterations from speed i to temporary df
  temp1 <- temp[temp$val == max(temp$val),]            # Within temp df, extract value where L is maxim

  RFC_profile <- rbind(RFC_profile, temp1)            # Store in empty df
}

RFC_profile <- RFC_profile[-1,]                      # Remove NA row used to create empty df
```

```
lnLmax <- RFC_profile$val[RFC_profile$val == min(RFC_profile$val)]
RFC_profile$lnL_diff <- RFC_profile$val - lnLmax
ci_profile_df <- RFC_profile[RFC_profile$lnL_diff < qchisq(p = 0.95, df = 1),]

p3min <- min(ci_profile_df$p3)
p3max <- max(ci_profile_df$p3)

# North epicentre

RFC_out_df$centroid <- paste(RFC_out_df$p1, RFC_out_df$p2, sep = " ")

RFC_profile <- data.frame(  # Empty dataframe to store loop output
  p1 = NA,
  p2 = NA,
  p3 = NA,
  p4 = NA,
  p5 = NA,
  p6 = NA,

  centroid = NA,
  val = NA
)

for(i in 1:length(unique(RFC_out_df$centroid))) { # For each unique centroid location
  match <- unique(RFC_out_df$centroid)            # Store all unique speeds
  temp <- subset(RFC_out_df, centroid == match[i])# Subset all iterations from speed i to temporary df
  temp1 <- temp[temp$val == max(temp$val),]           # Within temp df, extract value where L is maxim

  RFC_profile <- rbind(RFC_profile, temp1)          # Store in empty df
}

RFC_profile <- RFC_profile[-1,]                     # Remove NA row used to create empty df

lnLmax <- RFC_profile$val[RFC_profile$val == min(RFC_profile$val)]
RFC_profile$lnL_diff <- RFC_profile$val - lnLmax
ci_profile_df <- RFC_profile[RFC_profile$lnL_diff < qchisq(p = 0.95, df = 2),]

# Report range for centroid location
p1min <- min(ci_profile_df$p1)
p1max <- max(ci_profile_df$p1)

p2min <- min(ci_profile_df$p2)
p2max <- max(ci_profile_df$p2)

# South speed

RFC_profile <- data.frame(  # Empty dataframe to store loop output
  p1 = NA,
  p2 = NA,
  p3 = NA,

  p4 = NA,
  p5 = NA,
```

```r
  p6 = NA,

  centroid = NA,
  val = NA
)

for(i in 1:length(unique(RFC_out_df$p6))) { # For each unique value of speed
  match <- unique(RFC_out_df$p6)             # Store all unique speeds
  temp <- subset(RFC_out_df, p6 == match[i])# Subset all iterations from speed i to temporary df
  temp1 <- temp[temp$val == max(temp$val),]            # Within temp df, extract value where L is maxim

  RFC_profile <- rbind(RFC_profile, temp1)           # Store in empty df
}

RFC_profile <- RFC_profile[-1,]                      # Remove NA row used to create empty df
lnLmax <- RFC_profile$val[RFC_profile$val == min(RFC_profile$val)]
RFC_profile$lnL_diff <- RFC_profile$val - lnLmax
ci_profile_df <- RFC_profile[RFC_profile$lnL_diff < qchisq(p = 0.95, df = 1),]

p6min <- min(ci_profile_df$p6)
p6max <- max(ci_profile_df$p6)

# South epicentre

RFC_out_df$centroid <- paste(RFC_out_df$p4, RFC_out_df$p5, sep = " ")

RFC_profile <- data.frame(  # Empty dataframe to store loop output
  p1 = NA,
  p2 = NA,
  p3 = NA,

  p4 = NA,
  p5 = NA,
  p6 = NA,

  centroid = NA,
  val = NA
)

for(i in 1:length(unique(RFC_out_df$centroid))) { # For each unique centroid location
  match <- unique(RFC_out_df$centroid)            # Store all unique speeds
  temp <- subset(RFC_out_df, centroid == match[i])# Subset all iterations from speed i to temporary df
  temp1 <- temp[temp$val == max(temp$val),]            # Within temp df, extract value where L is maxim

  RFC_profile <- rbind(RFC_profile, temp1)           # Store in empty df
}

RFC_profile <- RFC_profile[-1,]                      # Remove NA row used to create empty df

lnLmax <- RFC_profile$val[RFC_profile$val == min(RFC_profile$val)]
RFC_profile$lnL_diff <- RFC_profile$val - lnLmax
ci_profile_df <- RFC_profile[RFC_profile$lnL_diff < qchisq(p = 0.95, df = 2),]
```

```
# Report range for centroid location
p4min <- min(ci_profile_df$p4)
p4max <- max(ci_profile_df$p4)

p5min <- min(ci_profile_df$p5)
p5max <- max(ci_profile_df$p5)

RFC_est <- data.frame(
  Parameter = c("North Epicentre X", "North Epicentre Y", "North Speed",
                "South Epicentre X", "South Epicentre Y", "South Speed"),
  Units = c("mean centred UTM", "mean centred UTM", "km per day",
            "mean centred UTM", "mean centred UTM", "km per day"),
  Estimate = comma(RFC_out$estimate),
  Lower95CI = comma(c(p1min, p2min, p3min, p4min, p5min, p6min)),
  Upper95CI = comma(c(p1max, p2max, p3max, p4max, p5max, p6max))
)
RFC_est
```

Parameter	Units	Estimate	Lower95CI	Upper95CI
North Epicentre X	mean centred UTM	328,433	123,814	1,123,603
North Epicentre Y	mean centred UTM	561,120	183,866	1,040,756
North Speed	km per day	5,574	3,350	12,088
South Epicentre X	mean centred UTM	242,077	146,419	773,274
South Epicentre Y	mean centred UTM	-223,234	-622,770	97,933
South Speed	km per day	4,204	2,151	9,326

RDC

```
RDC_out_df <- data.frame(RDC_out$retvals[,c(1:6, 19)])
RDC_out_df$val <- 2*RDC_out_df$val # convert to -2loglikelihood

# Activator speed

RDC_profile <- data.frame(  # Empty dataframe to store loop output
  p1 = NA,
  p2 = NA,
  p3 = NA,

  p4 = NA,
  p5 = NA,
  p6 = NA,

  val = NA
)

for(i in 1:length(unique(RDC_out_df$p3))) { # For each unique value of speed
  match <- unique(RDC_out_df$p3)            # Store all unique speeds
  temp <- subset(RDC_out_df, p3 == match[i])# Subset all iterations from speed i to temporary df
  temp1 <- temp[temp$val == max(temp$val),]            # Within temp df, extract value where L is maxim
```

```r
  RDC_profile <- rbind(RDC_profile, temp1)           # Store in empty df
}

RDC_profile <- RDC_profile[-1,]                      # Remove NA row used to create empty df
lnLmax <- RDC_profile$val[RDC_profile$val == min(RDC_profile$val)]
RDC_profile$lnL_diff <- RDC_profile$val - lnLmax
ci_profile_df <- RDC_profile[RDC_profile$lnL_diff < qchisq(p = 0.95, df = 1),]

p3min <- min(ci_profile_df$p3)
p3max <- max(ci_profile_df$p3)

# Activator epicentre

RDC_out_df$centroid <- paste(RDC_out_df$p1, RDC_out_df$p2, sep = " ")

RDC_profile <- data.frame(  # Empty dataframe to store loop output
  p1 = NA,
  p2 = NA,
  p3 = NA,
  p4 = NA,
  p5 = NA,
  p6 = NA,

  centroid = NA,
  val = NA
)

for(i in 1:length(unique(RDC_out_df$centroid))) { # For each unique centroid location
  match <- unique(RDC_out_df$centroid)            # Store all unique speeds
  temp <- subset(RDC_out_df, centroid == match[i])# Subset all iterations from speed i to temporary df
  temp1 <- temp[temp$val == max(temp$val),]            # Within temp df, extract value where L is maxim

  RDC_profile <- rbind(RDC_profile, temp1)           # Store in empty df
}

RDC_profile <- RDC_profile[-1,]                      # Remove NA row used to create empty df

lnLmax <- RDC_profile$val[RDC_profile$val == min(RDC_profile$val)]
RDC_profile$lnL_diff <- RDC_profile$val - lnLmax
ci_profile_df <- RDC_profile[RDC_profile$lnL_diff < qchisq(p = 0.95, df = 2),]

# Report range for centroid location
p1min <- min(ci_profile_df$p1)
p1max <- max(ci_profile_df$p1)

p2min <- min(ci_profile_df$p2)
p2max <- max(ci_profile_df$p2)

# Inhibitor speed

RDC_profile <- data.frame(  # Empty dataframe to store loop output
  p1 = NA,
  p2 = NA,
```

```r
  p3 = NA,

  p4 = NA,
  p5 = NA,
  p6 = NA,

  centroid = NA,
  val = NA
)

for(i in 1:length(unique(RDC_out_df$p6))) { # For each unique value of speed
  match <- unique(RDC_out_df$p6)            # Store all unique speeds
  temp <- subset(RDC_out_df, p6 == match[i])# Subset all iterations from speed i to temporary df
  temp1 <- temp[temp$val == max(temp$val),]            # Within temp df, extract value where L is maxim

  RDC_profile <- rbind(RDC_profile, temp1)           # Store in empty df
}

RDC_profile <- RDC_profile[-1,]                      # Remove NA row used to create empty df
lnLmax <- RDC_profile$val[RDC_profile$val == min(RDC_profile$val)]
RDC_profile$lnL_diff <- RDC_profile$val - lnLmax
ci_profile_df <- RDC_profile[RDC_profile$lnL_diff < qchisq(p = 0.95, df = 1),]

p6min <- min(ci_profile_df$p6)
p6max <- max(ci_profile_df$p6)

# Inhibitor epicentre

RDC_out_df$centroid <- paste(RDC_out_df$p4, RDC_out_df$p5, sep = " ")

RDC_profile <- data.frame(  # Empty dataframe to store loop output
  p1 = NA,
  p2 = NA,
  p3 = NA,

  p4 = NA,
  p5 = NA,
  p6 = NA,

  centroid = NA,
  val = NA
)

for(i in 1:length(unique(RDC_out_df$centroid))) { # For each unique centroid location
  match <- unique(RDC_out_df$centroid)            # Store all unique speeds
  temp <- subset(RDC_out_df, centroid == match[i])# Subset all iterations from speed i to temporary df
  temp1 <- temp[temp$val == max(temp$val),]            # Within temp df, extract value where L is maxim

  RDC_profile <- rbind(RDC_profile, temp1)           # Store in empty df
}

RDC_profile <- RDC_profile[-1,]                      # Remove NA row used to create empty df
```

```r
lnLmax <- RDC_profile$val[RDC_profile$val == min(RDC_profile$val)]
RDC_profile$lnL_diff <- RDC_profile$val - lnLmax
ci_profile_df <- RDC_profile[RDC_profile$lnL_diff < qchisq(p = 0.95, df = 2),]

# Report range for centroid location
p4min <- min(ci_profile_df$p4)
p4max <- max(ci_profile_df$p4)

p5min <- min(ci_profile_df$p5)
p5max <- max(ci_profile_df$p5)

RDC_est <- data.frame(
  Parameter = c("Activator Epicentre X", "Activator Epicentre Y", "Activator Speed",
                "Inhibitor Epicentre X", "Inhibitor Epicentre Y", "Inhibitor Speed"),
  Units = c("mean centred UTM", "mean centred UTM", "km per day",
            "mean centred UTM", "mean centred UTM", "km per day"),
  Estimate = comma(RDC_out$estimate),
  Lower95CI = comma(c(p1min, p2min, p3min, p4min, p5min, p6min)),
  Upper95CI = comma(c(p1max, p2max, p3max, p4max, p5max, p6max))
)
RDC_est
```

Parameter	Units	Estimate	Lower95CI	Upper95CI
Activator Epicentre X	mean centred UTM	-19,593	-43,574	-7,090
Activator Epicentre Y	mean centred UTM	-4,812	-15,265	19,138
Activator Speed	km per day	29,352	14,474	47,973
Inhibitor Epicentre X	mean centred UTM	9,283	-28,099	41,320
Inhibitor Epicentre Y	mean centred UTM	-44,488	-82,853	-20,169
Inhibitor Speed	km per day	37,245	17,543	77,447

PF

```r
PF_out_df <- data.frame(PF_out$retvals[,c(1:4, 13)])
PF_out_df$val <- 2*PF_out_df$val # convert to -2loglikelihood

# North angle

PF_profile <- data.frame(  # Empty dataframe to store loop output
  p1 = NA,
  p2 = NA,
  p3 = NA,
  p4 = NA,

  val = NA
)

for(i in 1:length(unique(PF_out_df$p1))) { # For each unique value of speed
  match <- unique(PF_out_df$p1)            # Store all unique speeds
  temp <- subset(PF_out_df, p1 == match[i])# Subset all iterations from speed i to temporary df
  temp1 <- temp[temp$val == max(temp$val),]            # Within temp df, extract value where L is maxim
```

```
  PF_profile <- rbind(PF_profile, temp1)           # Store in empty df
}

PF_profile <- PF_profile[-1,]                      # Remove NA row used to create empty df
lnLmax <- PF_profile$val[PF_profile$val == min(PF_profile$val)]
PF_profile$lnL_diff <- PF_profile$val - lnLmax
ci_profile_df <- PF_profile[PF_profile$lnL_diff < qchisq(p = 0.95, df = 1),]

p1min <- min(ci_profile_df$p1)
p1max <- max(ci_profile_df$p1)

# North speed

PF_profile <- data.frame(  # Empty dataframe to store loop output
  p1 = NA,
  p2 = NA,
  p3 = NA,
  p4 = NA,

  val = NA
)

for(i in 1:length(unique(PF_out_df$p2))) { # For each unique centroid location
  match <- unique(PF_out_df$p2)            # Store all unique speeds
  temp <- subset(PF_out_df, p2 == match[i])# Subset all iterations from speed i to temporary df
  temp1 <- temp[temp$val == max(temp$val),]            # Within temp df, extract value where L is maxim

  PF_profile <- rbind(PF_profile, temp1)           # Store in empty df
}

PF_profile <- PF_profile[-1,]                      # Remove NA row used to create empty df

lnLmax <- PF_profile$val[PF_profile$val == min(PF_profile$val)]
PF_profile$lnL_diff <- PF_profile$val - lnLmax
ci_profile_df <- PF_profile[PF_profile$lnL_diff < qchisq(p = 0.95, df = 1),]

p2min <- min(ci_profile_df$p2)
p2max <- max(ci_profile_df$p2)

# South angle

PF_profile <- data.frame(  # Empty dataframe to store loop output
  p1 = NA,
  p2 = NA,
  p3 = NA,
  p4 = NA,

  val = NA
)

for(i in 1:length(unique(PF_out_df$p3))) { # For each unique value of speed
  match <- unique(PF_out_df$p3)            # Store all unique speeds
  temp <- subset(PF_out_df, p3 == match[i])# Subset all iterations from speed i to temporary df
```

```
  temp1 <- temp[temp$val == max(temp$val),]            # Within temp df, extract value where L is maxim

  PF_profile <- rbind(PF_profile, temp1)           # Store in empty df
}

PF_profile <- PF_profile[-1,]                        # Remove NA row used to create empty df
lnLmax <- PF_profile$val[PF_profile$val == min(PF_profile$val)]
PF_profile$lnL_diff <- PF_profile$val - lnLmax
ci_profile_df <- PF_profile[PF_profile$lnL_diff < qchisq(p = 0.95, df = 1),]

p3min <- min(ci_profile_df$p3)
p3max <- max(ci_profile_df$p3)

# South angle
PF_profile <- data.frame(  # Empty dataframe to store loop output
  p1 = NA,
  p2 = NA,
  p3 = NA,
  p4 = NA,

  val = NA
)

for(i in 1:length(unique(PF_out_df$p4))) { # For each unique centroid location
  match <- unique(PF_out_df$p4)            # Store all unique speeds
  temp <- subset(PF_out_df, p4 == match[i])# Subset all iterations from speed i to temporary df
  temp1 <- temp[temp$val == max(temp$val),]            # Within temp df, extract value where L is maxim

  PF_profile <- rbind(PF_profile, temp1)           # Store in empty df
}

PF_profile <- PF_profile[-1,]                        # Remove NA row used to create empty df

lnLmax <- PF_profile$val[PF_profile$val == min(PF_profile$val)]
PF_profile$lnL_diff <- PF_profile$val - lnLmax
ci_profile_df <- PF_profile[PF_profile$lnL_diff < qchisq(p = 0.95, df = 1),]

# Report range for centroid location
p4min <- min(ci_profile_df$p4)
p4max <- max(ci_profile_df$p4)

PF_est <- data.frame(
  Parameter = c("North Angle", "North Speed",
                "South Angle", "South Speed"),
  Units = c("radian", "km per day",
            "radian", "km per day"),
  Estimate = comma(PF_out$estimate),
  Lower95CI = comma(c(p1min, p2min, p3min, p4min)),
  Upper95CI = comma(c(p1max, p2max, p3max, p4max))
)
PF_est
```

Parameter	Units	Estimate	Lower95CI	Upper95CI
North Angle	radian	3.5	3.2	4.0
North Speed	km per day	3,291.0	2,120.1	7,312.9
South Angle	radian	5.8	5.6	6.0
South Speed	km per day	1,385.7	1,042.7	2,243.6

PD

```
PD_out_df <- data.frame(PD_out$retvals[,c(1:4, 13)])
PD_out_df$val <- 2*PD_out_df$val # convert to -2loglikelihood

# North angle

PD_profile <- data.frame(  # Empty dataframe to store loop output
  p1 = NA,
  p2 = NA,
  p3 = NA,
  p4 = NA,

  val = NA
)

for(i in 1:length(unique(PD_out_df$p1))) { # For each unique value of speed
  match <- unique(PD_out_df$p1)            # Store all unique speeds
  temp <- subset(PD_out_df, p1 == match[i])# Subset all iterations from speed i to temporary df
  temp1 <- temp[temp$val == max(temp$val),]            # Within temp df, extract value where L is maxim

  PD_profile <- rbind(PD_profile, temp1)           # Store in empty df
}

PD_profile <- PD_profile[-1,]                      # Remove NA row used to create empty df
lnLmax <- PD_profile$val[PD_profile$val == min(PD_profile$val)]
PD_profile$lnL_diff <- PD_profile$val - lnLmax
ci_profile_df <- PD_profile[PD_profile$lnL_diff < qchisq(p = 0.95, df = 1),]

p1min <- min(ci_profile_df$p1)
p1max <- max(ci_profile_df$p1)

# North speed

PD_profile <- data.frame(  # Empty dataframe to store loop output
  p1 = NA,
  p2 = NA,
  p3 = NA,
  p4 = NA,

  val = NA
)

for(i in 1:length(unique(PD_out_df$p2))) { # For each unique centroid location
  match <- unique(PD_out_df$p2)            # Store all unique speeds
```

```r
  temp <- subset(PD_out_df, p2 == match[i])# Subset all iterations from speed i to temporary df
  temp1 <- temp[temp$val == max(temp$val),]            # Within temp df, extract value where L is maxim

  PD_profile <- rbind(PD_profile, temp1)           # Store in empty df
}

PD_profile <- PD_profile[-1,]                      # Remove NA row used to create empty df

lnLmax <- PD_profile$val[PD_profile$val == min(PD_profile$val)]
PD_profile$lnL_diff <- PD_profile$val - lnLmax
ci_profile_df <- PD_profile[PD_profile$lnL_diff < qchisq(p = 0.95, df = 1),]

p2min <- min(ci_profile_df$p2)
p2max <- max(ci_profile_df$p2)

# South angle

PD_profile <- data.frame(  # Empty dataframe to store loop output
  p1 = NA,
  p2 = NA,
  p3 = NA,
  p4 = NA,

  val = NA
)

for(i in 1:length(unique(PD_out_df$p3))) { # For each unique value of speed
  match <- unique(PD_out_df$p3)            # Store all unique speeds
  temp <- subset(PD_out_df, p3 == match[i])# Subset all iterations from speed i to temporary df
  temp1 <- temp[temp$val == max(temp$val),]            # Within temp df, extract value where L is maxim

  PD_profile <- rbind(PD_profile, temp1)           # Store in empty df
}

PD_profile <- PD_profile[-1,]                      # Remove NA row used to create empty df
lnLmax <- PD_profile$val[PD_profile$val == min(PD_profile$val)]
PD_profile$lnL_diff <- PD_profile$val - lnLmax
ci_profile_df <- PD_profile[PD_profile$lnL_diff < qchisq(p = 0.95, df = 1),]

p3min <- min(ci_profile_df$p3)
p3max <- max(ci_profile_df$p3)

# South angle
PD_profile <- data.frame(  # Empty dataframe to store loop output
  p1 = NA,
  p2 = NA,
  p3 = NA,
  p4 = NA,

  val = NA
)

for(i in 1:length(unique(PD_out_df$p4))) { # For each unique centroid location
```

```
  match <- unique(PD_out_df$p4)           # Store all unique speeds
  temp <- subset(PD_out_df, p4 == match[i])# Subset all iterations from speed i to temporary df
  temp1 <- temp[temp$val == max(temp$val),]           # Within temp df, extract value where L is maxim

  PD_profile <- rbind(PD_profile, temp1)           # Store in empty df
}

PD_profile <- PD_profile[-1,]                    # Remove NA row used to create empty df

lnLmax <- PD_profile$val[PD_profile$val == min(PD_profile$val)]
PD_profile$lnL_diff <- PD_profile$val - lnLmax
ci_profile_df <- PD_profile[PD_profile$lnL_diff < qchisq(p = 0.95, df = 1),]

# Report range for centroid location
p4min <- min(ci_profile_df$p4)
p4max <- max(ci_profile_df$p4)

PD_est <- data.frame(
  Parameter = c("Activator Angle", "Activator Speed",
              "Inhibitor Angle", "Inhibitor Speed"),
  Units = c("radian", "km per day",
          "radian", "km per day"),
  Estimate = comma(PD_out$estimate),
  Lower95CI = comma(c(p1min, p2min, p3min, p4min)),
  Upper95CI = comma(c(p1max, p2max, p3max, p4max))
)
PD_est
```

Parameter	Units	Estimate	Lower95CI	Upper95CI
Activator Angle	radian	3.0	2.8	3.1
Activator Speed	km per day	527.0	421.1	746.9
Inhibitor Angle	radian	4.8	3.9	5.4
Inhibitor Speed	km per day	7,162.9	6,530.7	16,844.3

Figures

Optimisation figures

These figures show the optimisation process for models using SANN. Each parameter value tested is indicated with a grey dot. When the $-2ln\mathcal{L}$ is $\leq \chi^2(0.95, df)$ (where *df* is either 2, for centroid locations, or else 1), the $-2ln\mathcal{L}$ is indicated with a coloured dot according to $\Delta - 2ln\mathcal{L}$.

RE

```
RE_out_df$iter <- as.numeric(row.names(RE_out_df))
RE_out_df$L <- RE_out_df$val - min(RE_out_df$val)
```

```r
ggplot(RE_out_df) +
  geom_point(data = space, aes(x = cen.x, y = cen.y),
             colour = "black", size = 0.5, shape = 2, alpha = 0.2) +
  geom_path(aes(x = p1, y = p2),
            linetype = 2,
            colour = "grey") +
  geom_point(aes(x = p1, y = p2, group = iter),
             size = 2, colour = "grey") +
  geom_point(data = RE_out_df[RE_out_df$L <= qchisq(0.95, 2),],
             aes(x = p1, y = p2, colour = val, group = iter),
             size = 1.5) +
  scale_colour_viridis_c(option = "C") +
  theme_bw() +
  theme(text = element_text(size = 15)) +
  labs(x = "X UTM (centered)",
       y = "Y UTM (centered)",
       title = "RE Centroid location",
       colour = expression(paste(Delta, "-2lnL <= ", chi^{2}, "(0.95, 2)")))
```

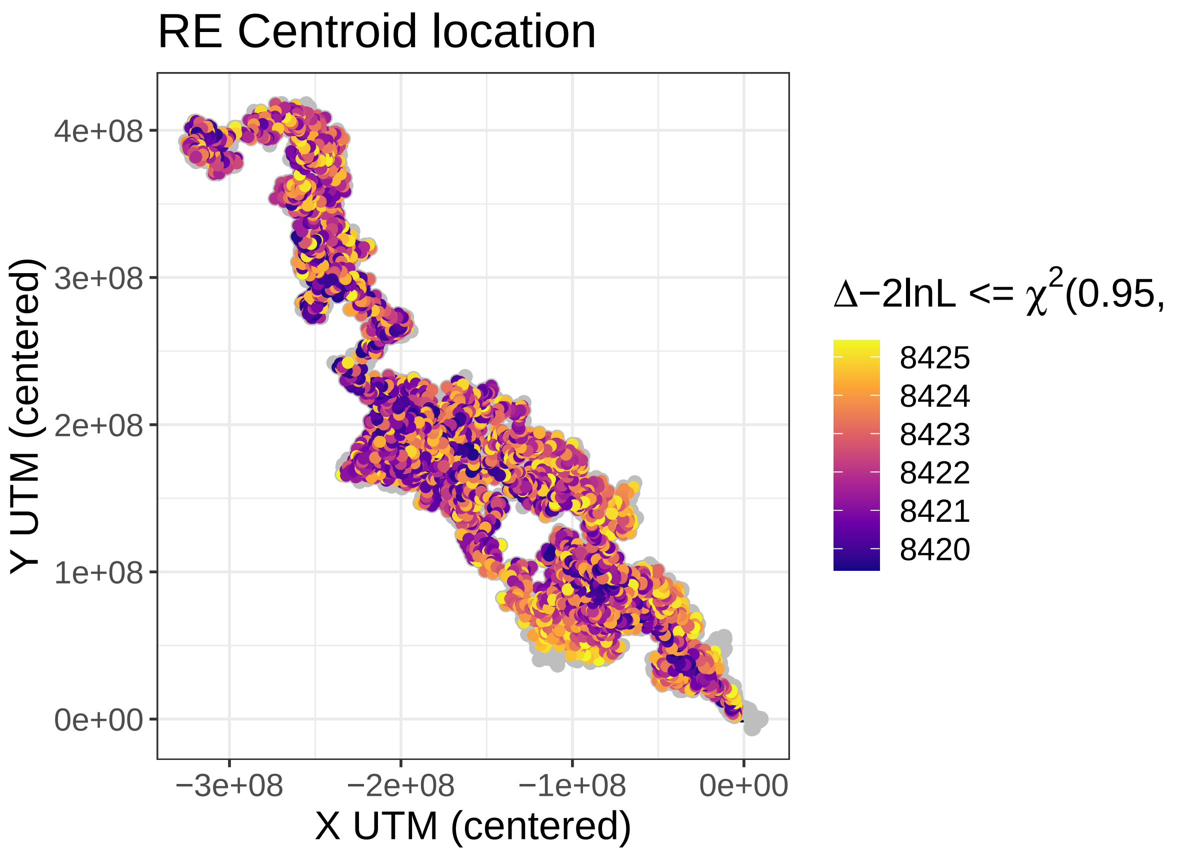

Centroid location

```r
ggplot(RE_out_df) +
  geom_point(aes(x = p3, y = val, group = iter),
             size = 2, colour = "grey") +
```

```
  geom_point(data = RE_out_df[RE_out_df$L <= qchisq(0.95, 1),],
             aes(x = p3, y = val, colour = val, group = iter),
             size = 1.5) +
  scale_colour_viridis_c(option = "C") +
  theme_bw() +
  theme(text = element_text(size = 15)) +
  labs(x = "Speed (m per day)",
       y = "-2lnL",
       title = "RE Speed",
       colour = expression(paste(Delta, "-2lnL <= ", chi^{2}, "(0.95, 1)")))
```

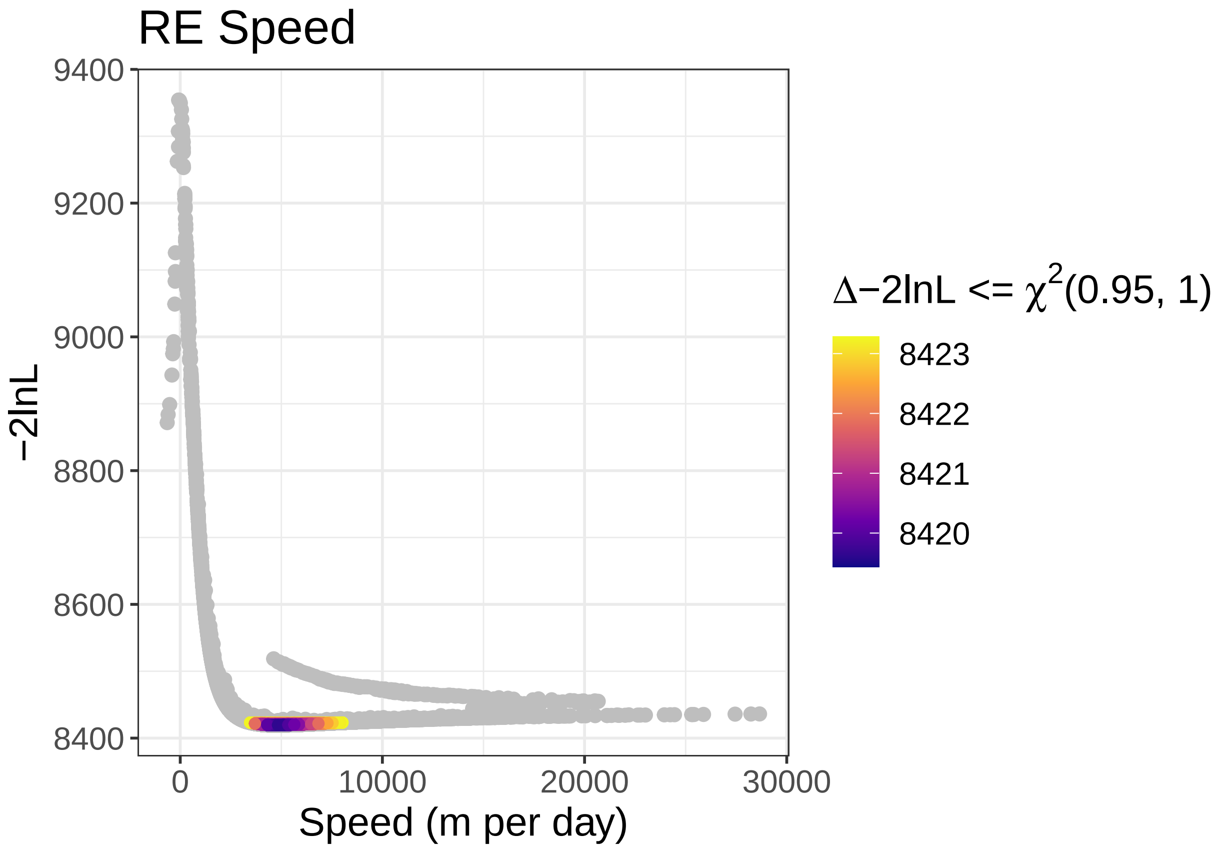

Speed

RC

```
RC_out_df$iter <- as.numeric(row.names(RC_out_df))
RC_out_df$L <- RC_out_df$val - min(RC_out_df$val)

ggplot(RC_out_df) +
  geom_point(data = space, aes(x = cen.x, y = cen.y),
             colour = "black", size = 0.5, shape = 2, alpha = 0.2) +
  geom_point(aes(x = p1, y = p2, group = iter),
             size = 2, colour = "grey") +
  geom_point(data = RC_out_df[RC_out_df$L <= qchisq(0.95, 2),],
```

```
             aes(x = p1, y = p2, colour = val, group = iter),
             size = 1.5) +
  scale_colour_viridis_c(option = "C") +
  theme_bw() +
  theme(text = element_text(size = 15)) +
  labs(x = "X UTM (centered)",
       y = "Y UTM (centered)",
       title = "RC Centroid location",
       colour = expression(paste(Delta, "-2lnL <= ", chi^{2}, "(0.95, 2)")))
```

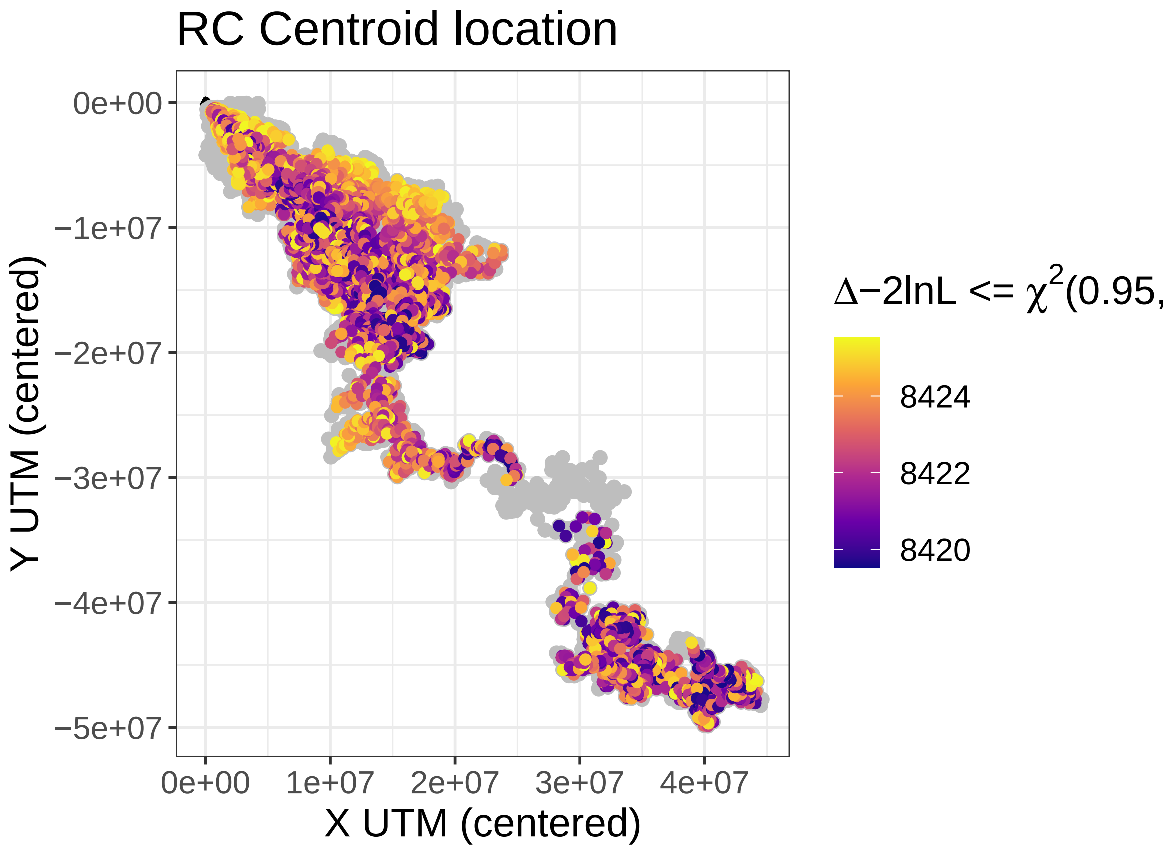

Centroid location

```
ggplot(RC_out_df) +
  geom_point(aes(x = p3, y = val, group = iter),
             size = 2, colour = "grey") +
  geom_point(data = RC_out_df[RC_out_df$L <= qchisq(0.95, 1),],
             aes(x = p3, y = val, colour = val, group = iter),
             size = 1.5) +
  scale_colour_viridis_c(option = "C") +
  theme_bw() +
  theme(text = element_text(size = 15)) +
  labs(x = "Speed (m per day)",
       y = "-2lnL",
       title = "RC Speed",
       colour = expression(paste(Delta, "-2lnL <= ", chi^{2}, "(0.95, 1)")))
```

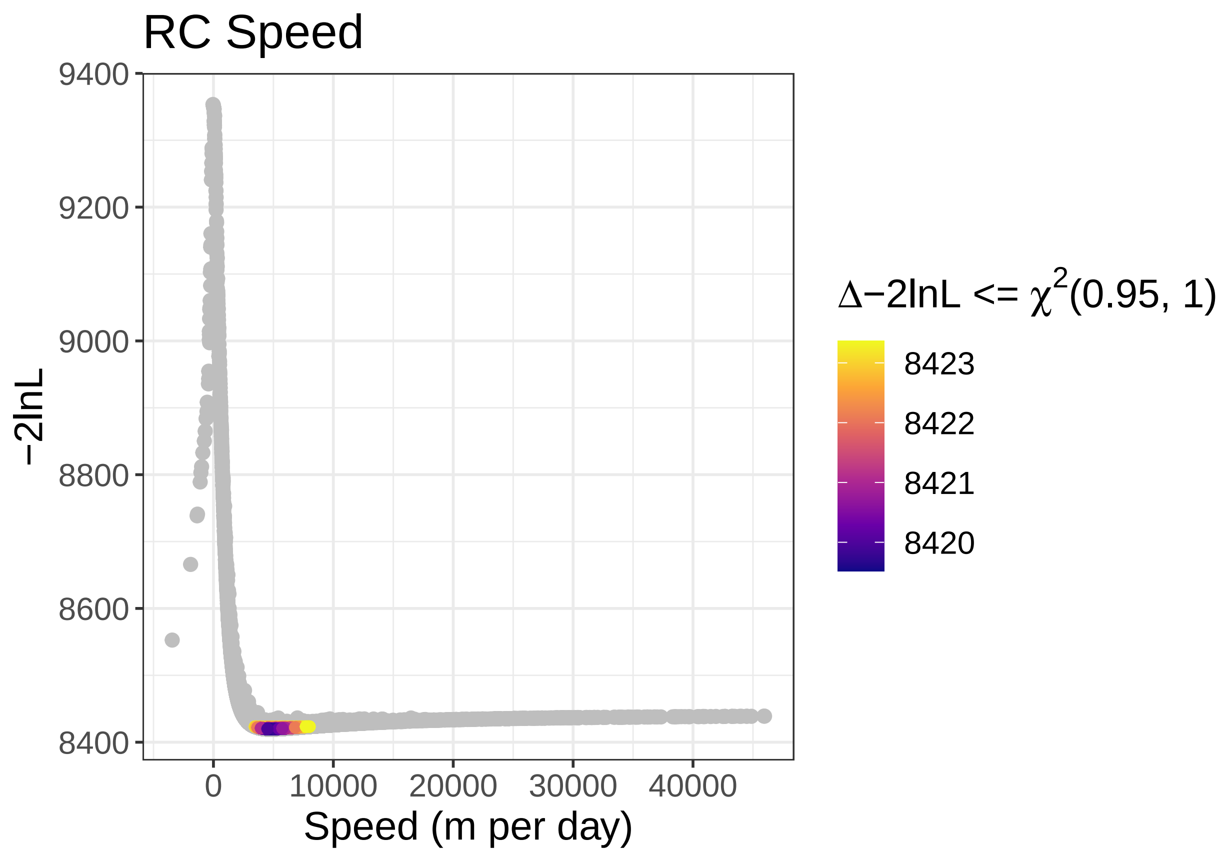

Speed

P

```
P_out_df$iter <- as.numeric(row.names(P_out_df))
P_out_df$L <- P_out_df$val - min(P_out_df$val)

ggplot(P_out_df) +
  geom_point(aes(x = p1, y = val, group = iter),
             size = 2, colour = "grey") +
  geom_point(data = P_out_df[P_out_df$L <= qchisq(0.95, 1),],
             aes(x = p1, y = val, colour = val, group = iter),
             size = 1.5) +
  scale_colour_viridis_c(option = "C") +
  theme_bw() +
  theme(text = element_text(size = 15)) +
  labs(x = "Angle (radian)",
       y = "-2lnL",
       title = "P Angle",
       colour = expression(paste(Delta, "-2lnL <= ", chi^{2}, "(0.95, 1)")))
```

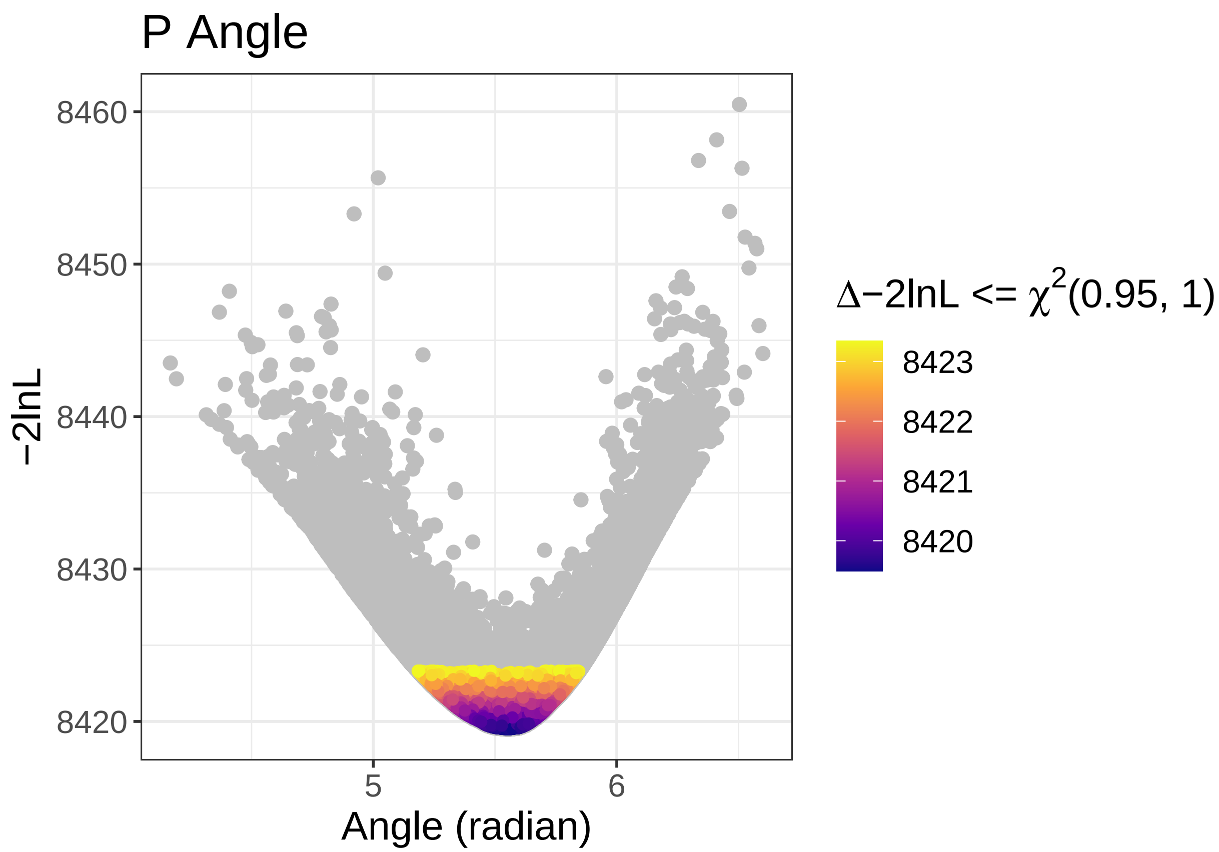

Angle

```
ggplot(P_out_df) +
  geom_point(aes(x = p2, y = val, group = iter),
             size = 2, colour = "grey") +
  geom_point(data = P_out_df[P_out_df$L <= qchisq(0.95, 1),],
             aes(x = p2, y = val, colour = val, group = iter),
             size = 1.5) +
  scale_colour_viridis_c(option = "C") +
  theme_bw() +
  theme(text = element_text(size = 15)) +
  labs(x = "Speed (km per day)",
       y = "-2lnL",
       title = "P Speed",
       colour = expression(paste(Delta, "-2lnL <= ", chi^{2}, "(0.95, 1)")))
```

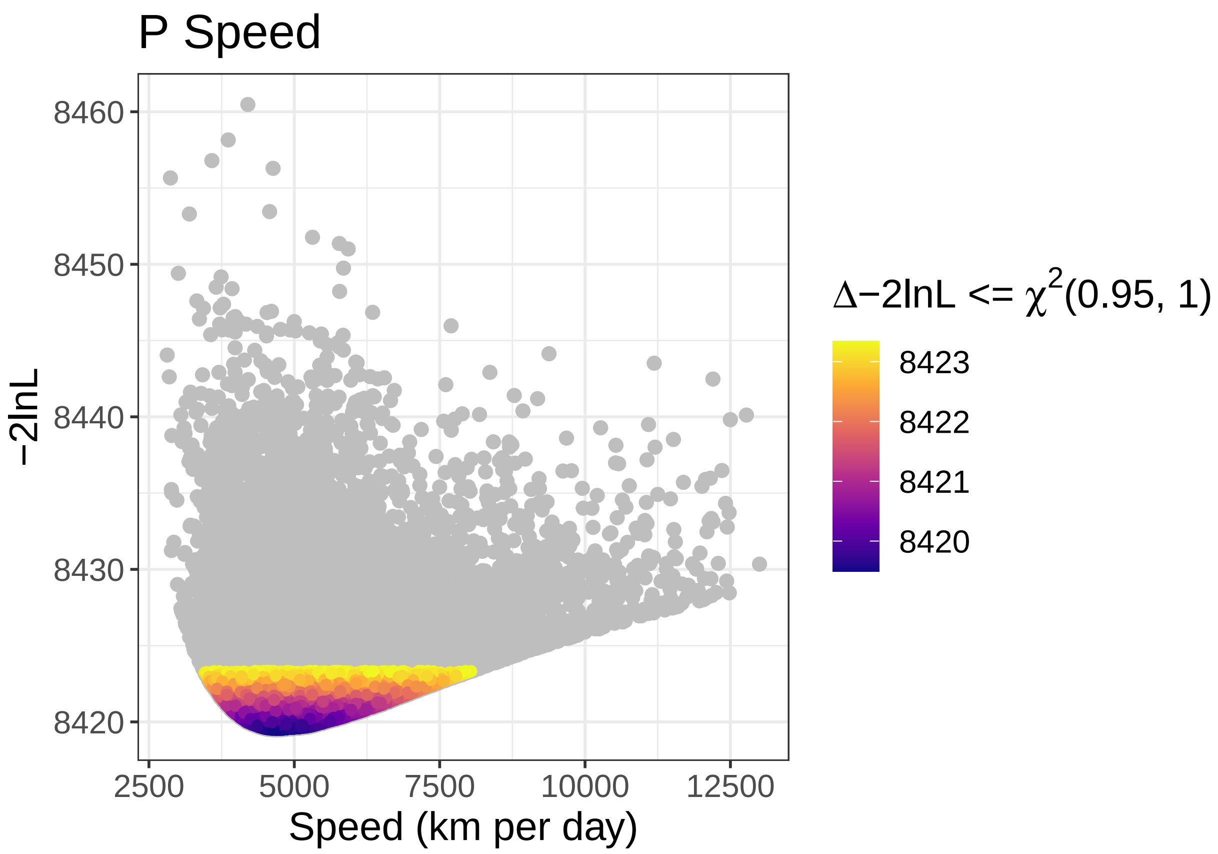

Speed

RFE

```
RFE_out_df$iter <- as.numeric(row.names(RFE_out_df))
RFE_out_df$L <- RFE_out_df$val - min(RFE_out_df$val)

ggplot(RFE_out_df) +
  geom_point(data = space, aes(x = cen.x, y = cen.y),
             colour = "black", size = 0.5, shape = 2, alpha = 0.2) +
  geom_point(aes(x = p1, y = p2, group = iter),
             size = 2, colour = "grey") +
  geom_point(data = RFE_out_df[RFE_out_df$L <= qchisq(0.95, 2),],
             aes(x = p1, y = p2, colour = val, group = iter),
             size = 1.5) +
  geom_point(aes(x = p4, y = p5, group = iter),
             size = 2, colour = "grey") +
  geom_point(data = RFE_out_df[RFE_out_df$L <= qchisq(0.95, 2),],
             aes(x = p4, y = p5, colour = val, group = iter),
             size = 1.5) +
  scale_colour_viridis_c(option = "C") +
  theme_bw() +
  theme(text = element_text(size = 15)) +
  labs(x = "X UTM (centered)",
       y = "Y UTM (centered)",
```

```
       title = "RFE Centroid location",
       colour = expression(paste(Delta, "-2lnL <= ", chi^{2}, "(0.95, 2)")))
```

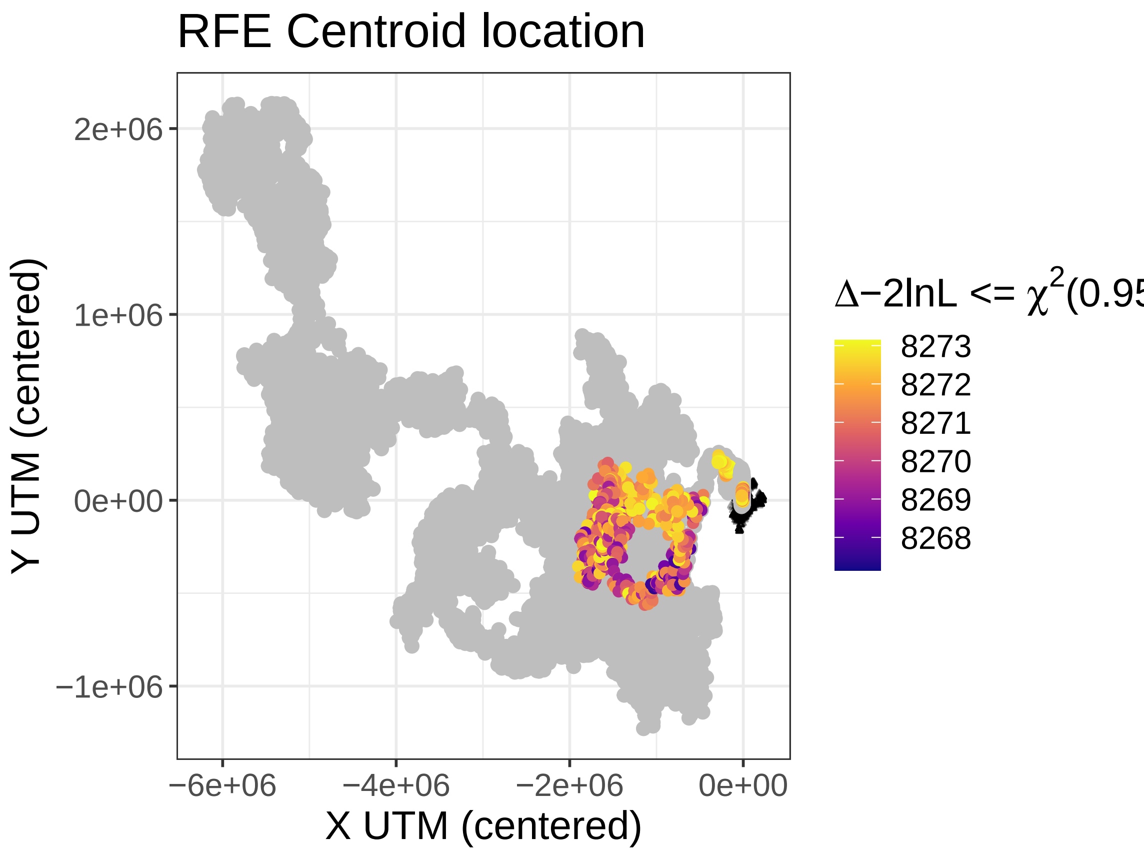

Both wave centroids

```
ggplot(RFE_out_df) +
  geom_point(aes(x = p3, y = val, group = iter),
             size = 2, colour = "grey") +
  geom_point(data = RFE_out_df[RFE_out_df$L <= qchisq(0.95, 1),],
             aes(x = p3, y = val, colour = val, group = iter),
             size = 1.5) +
  scale_colour_viridis_c(option = "C") +
  theme_bw() +
  theme(text = element_text(size = 15)) +
  labs(x = "Speed (m per day)",
       y = "-2lnL",
       title = "RFE North speed",
       colour = expression(paste(Delta, "-2lnL <= ", chi^{2}, "(0.95, 1)")))
```

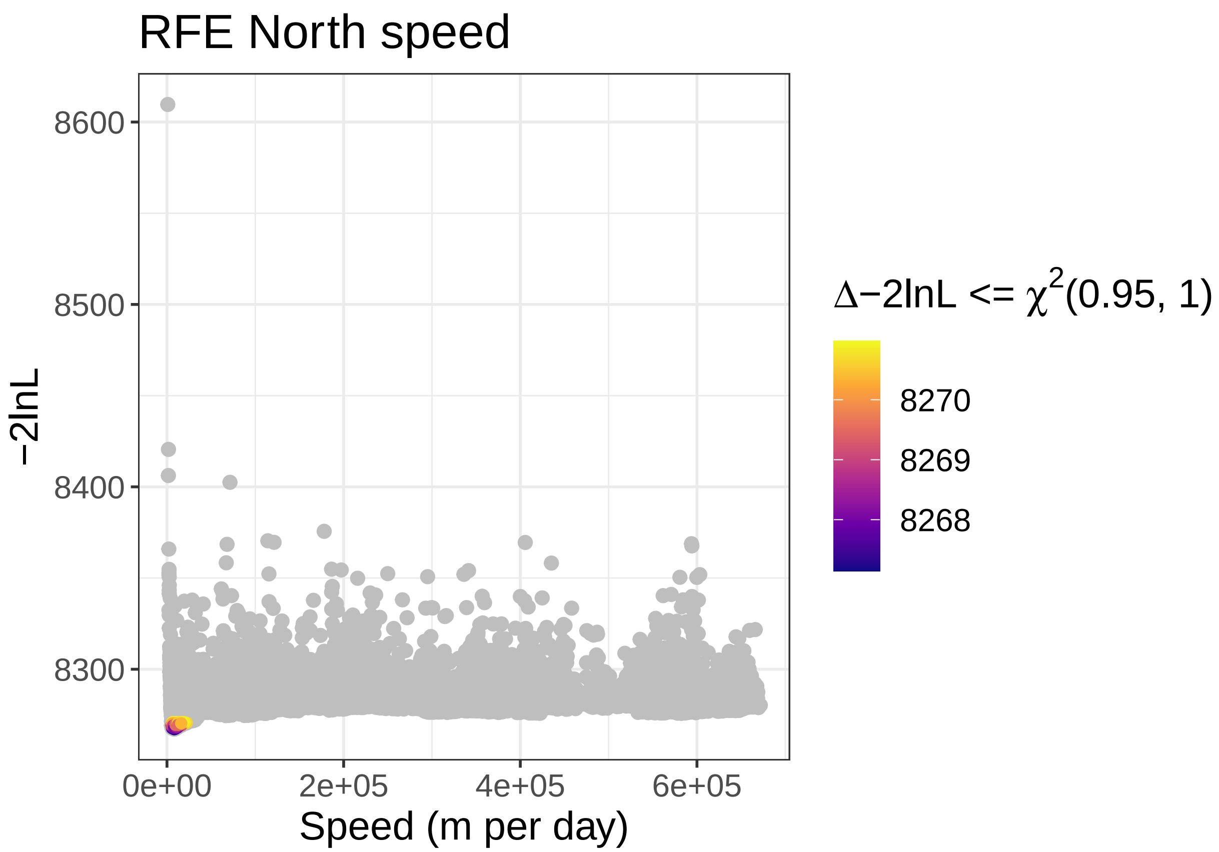

North speed

```
ggplot(RFE_out_df) +
  geom_point(aes(x = p6, y = val, group = iter),
             size = 2, colour = "grey") +
  geom_point(data = RFE_out_df[RFE_out_df$L <= qchisq(0.95, 1),],
             aes(x = p6, y = val, colour = val, group = iter),
             size = 1.5) +
  scale_colour_viridis_c(option = "C") +
  theme_bw() +
  theme(text = element_text(size = 15)) +
  labs(x = "Speed (m per day)",
       y = "-2lnL",
       title = "RFE South speed",
       colour = expression(paste(Delta, "-2lnL <= ", chi^{2}, "(0.95, 1)")))
```

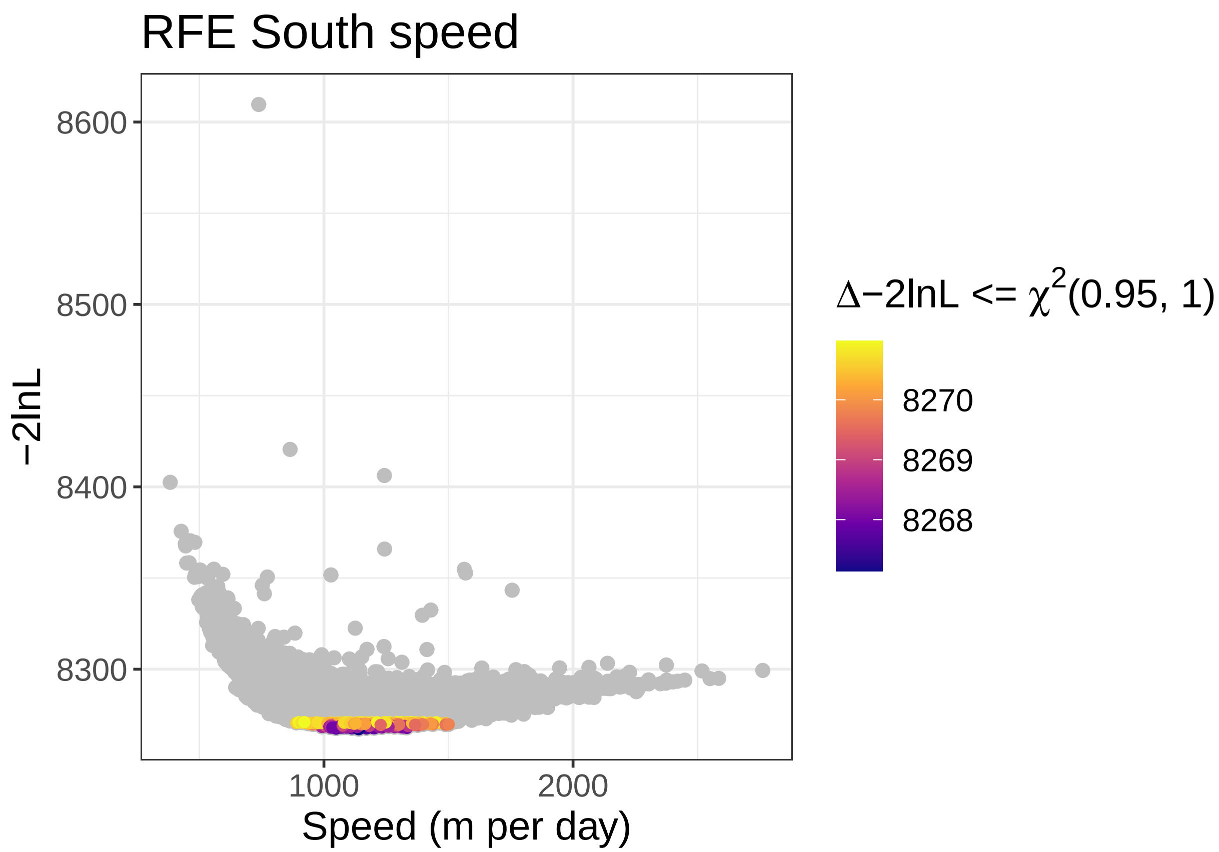

South speed

RDE

```
RDE_out_df$iter <- as.numeric(row.names(RDE_out_df))
RDE_out_df$L <- RDE_out_df$val - min(RDE_out_df$val)

ggplot(RDE_out_df) +
  geom_point(data = space, aes(x = cen.x, y = cen.y),
             colour = "black", size = 0.5, shape = 2, alpha = 0.2) +
  geom_point(aes(x = p1, y = p2, group = iter),
             size = 2, colour = "grey") +
  geom_point(data = RDE_out_df[RDE_out_df$L <= qchisq(0.95, 2),],
             aes(x = p1, y = p2, colour = val, group = iter),
             size = 1.5) +
  geom_point(aes(x = p4, y = p5, group = iter),
             size = 2, colour = "grey") +
  geom_point(data = RDE_out_df[RDE_out_df$L <= qchisq(0.95, 2),],
             aes(x = p4, y = p5, colour = val, group = iter),
             size = 1.5) +
  scale_colour_viridis_c(option = "C") +
  theme_bw() +
  theme(text = element_text(size = 15)) +
  labs(x = "X UTM (centered)",
       y = "Y UTM (centered)",
```

```
       title = "RDE Centroid location",
       colour = expression(paste(Delta, "-2lnL <= ", chi^{2}, "(0.95, 2)")))
```

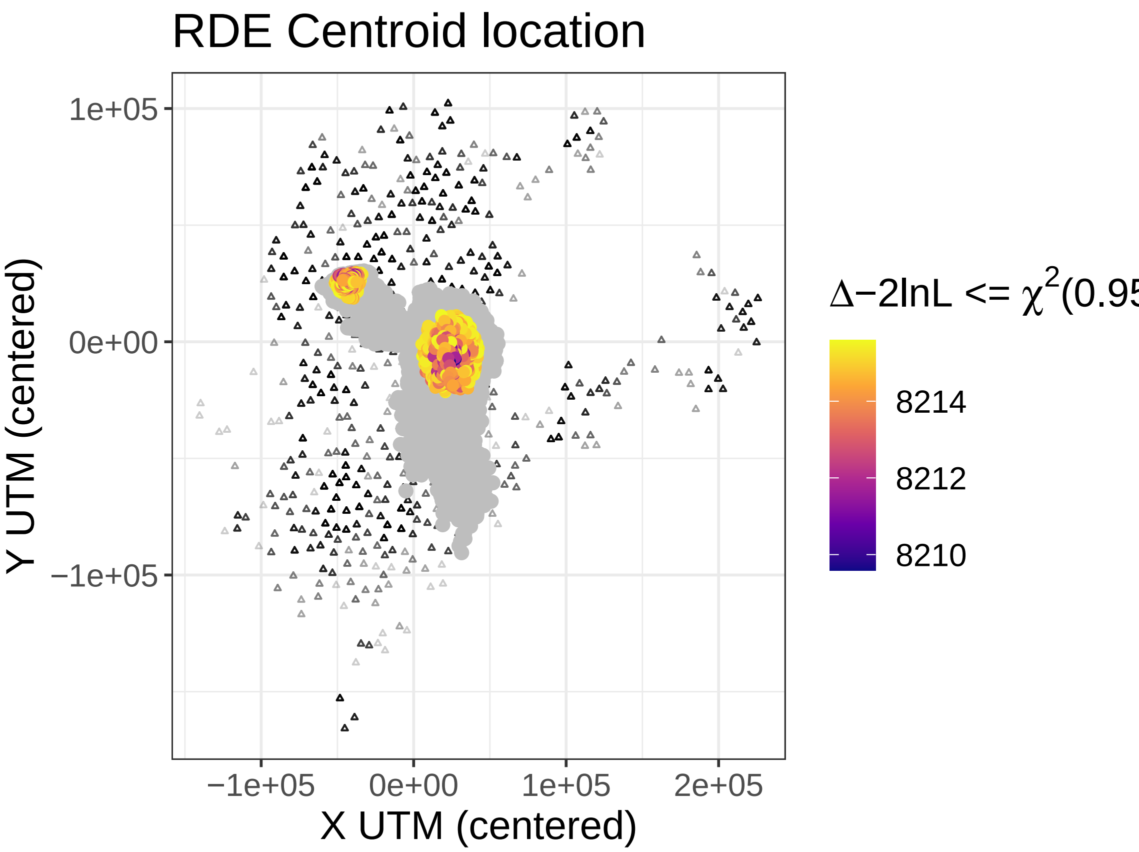

Both wave centroids

```
ggplot(RDE_out_df) +
  geom_point(aes(x = p3, y = val, group = iter),
             size = 2, colour = "grey") +
  geom_point(data = RDE_out_df[RDE_out_df$L <= qchisq(0.95, 1),],
             aes(x = p3, y = val, colour = val, group = iter),
             size = 1.5) +
  scale_colour_viridis_c(option = "C") +
  theme_bw() +
  theme(text = element_text(size = 15)) +
  labs(x = "Speed (m per day)",
       y = "-2lnL",
       title = "RDE Activator speed",
       colour = expression(paste(Delta, "-2lnL <= ", chi^{2}, "(0.95, 1)")))
```

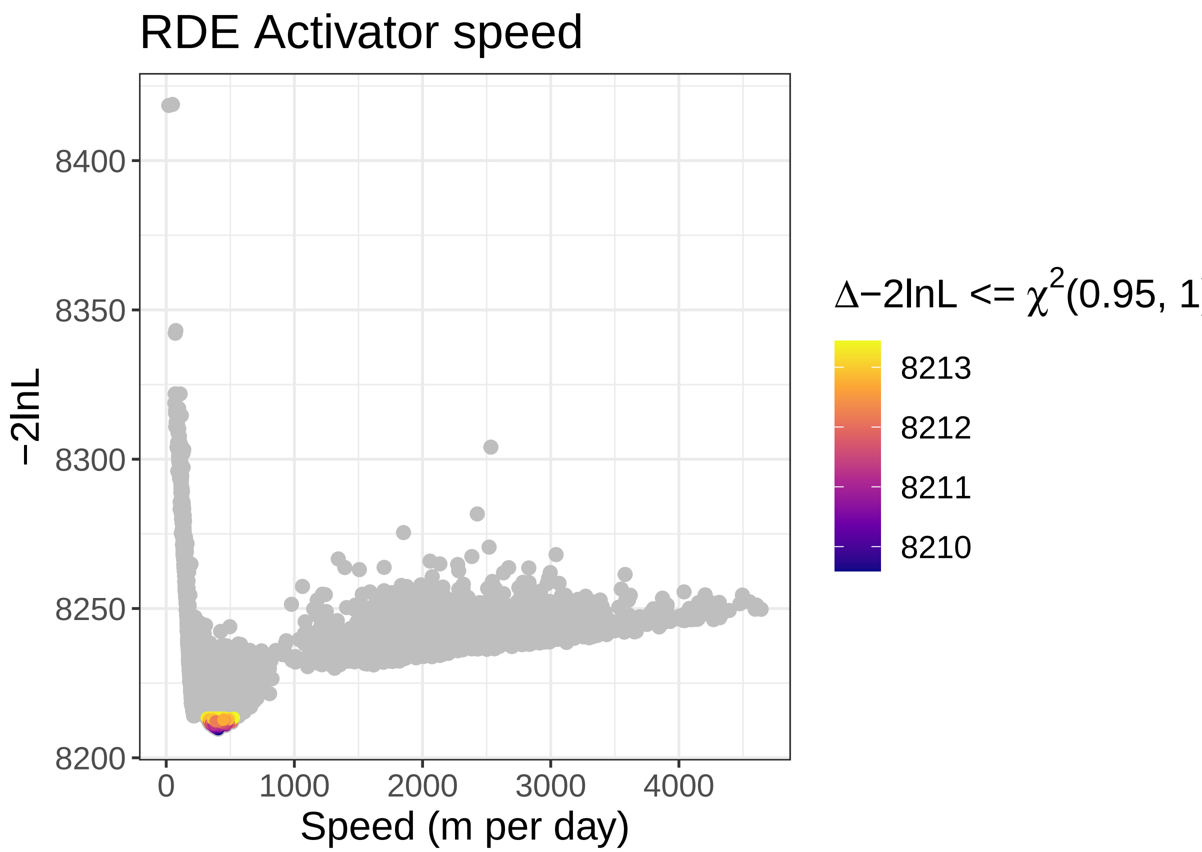

Activator speed

```
ggplot(RDE_out_df) +
  geom_point(aes(x = p6, y = val, group = iter),
             size = 2, colour = "grey") +
  geom_point(data = RDE_out_df[RDE_out_df$L <= qchisq(0.95, 1),],
             aes(x = p6, y = val, colour = val, group = iter),
             size = 1.5) +
  scale_colour_viridis_c(option = "C") +
  theme_bw() +
  theme(text = element_text(size = 15)) +
  labs(x = "Speed (m per day)",
       y = "-2lnL",
       title = "RDE Inhibitor speed",
       colour = expression(paste(Delta, "-2lnL <= ", chi^{2}, "(0.95, 1)")))
```

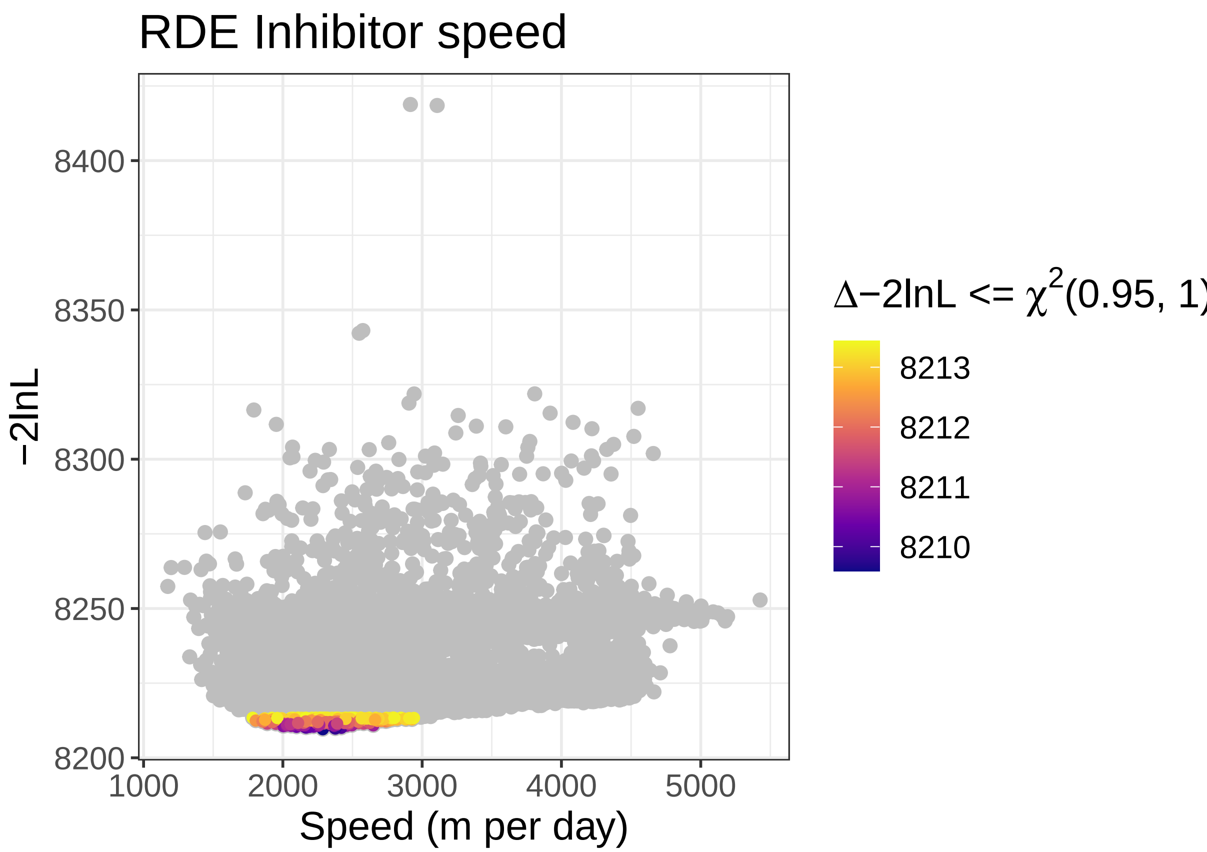

Inhibitor speed

RFC

```
RFC_out_df$iter <- as.numeric(row.names(RFC_out_df))
RFC_out_df$L <- RFC_out_df$val - min(RFC_out_df$val)

ggplot(RFC_out_df) +
  geom_point(data = space, aes(x = cen.x, y = cen.y),
             colour = "black", size = 0.5, shape = 2, alpha = 0.2) +
  geom_point(aes(x = p1, y = p2, group = iter),
             size = 2, colour = "grey") +
  geom_point(data = RFC_out_df[RFC_out_df$L <= qchisq(0.95, 2),],
             aes(x = p1, y = p2, colour = val, group = iter),
             size = 1.5) +
  geom_point(aes(x = p4, y = p5, group = iter),
             size = 2, colour = "grey") +
  geom_point(data = RFC_out_df[RFC_out_df$L <= qchisq(0.95, 2),],
             aes(x = p4, y = p5, colour = val, group = iter),
             size = 1.5) +
  scale_colour_viridis_c(option = "C") +
  theme_bw() +
  theme(text = element_text(size = 15)) +
  labs(x = "X UTM (centered)",
       y = "Y UTM (centered)",
```

```
       title = "RFC Centroid location",
       colour = expression(paste(Delta, "-2lnL <= ", chi^{2}, "(0.95, 2)")))
```

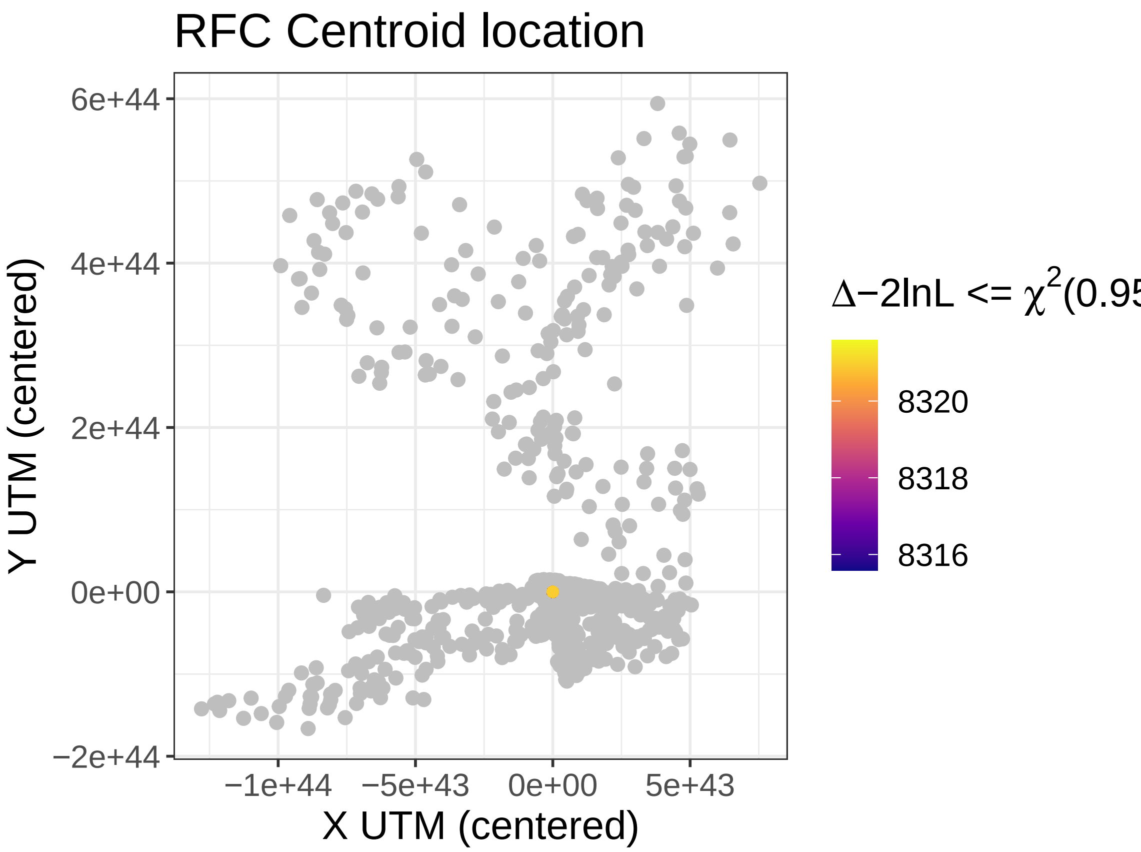

Both wave centroids

```
ggplot(RFC_out_df) +
  geom_point(aes(x = p3, y = val, group = iter),
             size = 2, colour = "grey") +
  geom_point(data = RFC_out_df[RFC_out_df$L <= qchisq(0.95, 1),],
             aes(x = p3, y = val, colour = val, group = iter),
             size = 1.5) +
  scale_colour_viridis_c(option = "C") +
  theme_bw() +
  theme(text = element_text(size = 15)) +
  labs(x = "Speed (m per day)",
       y = "-2lnL",
       title = "RFC North speed",
       colour = expression(paste(Delta, "-2lnL <= ", chi^{2}, "(0.95, 1)")))
```

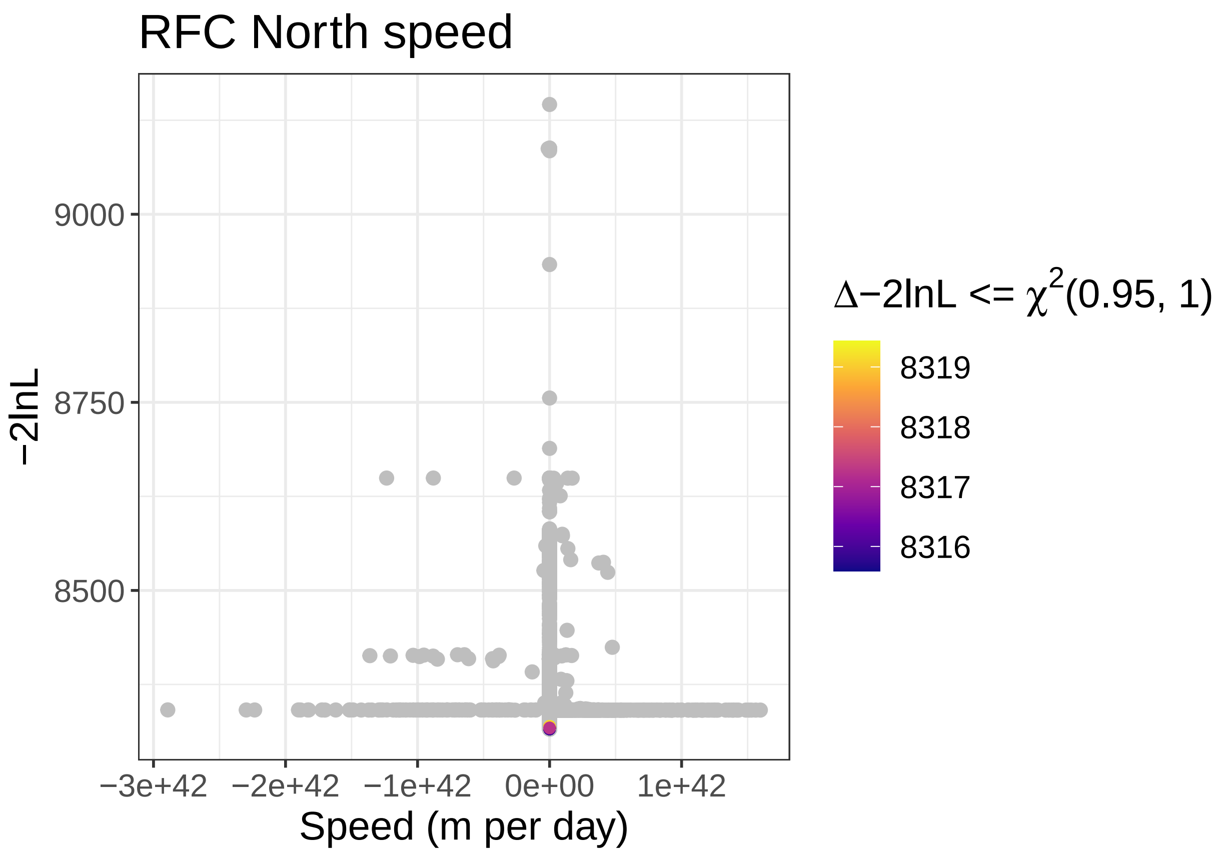

North speed

```
ggplot(RFC_out_df) +
  geom_point(aes(x = p6, y = val, group = iter),
             size = 2, colour = "grey") +
  geom_point(data = RFC_out_df[RFC_out_df$L <= qchisq(0.95, 1),],
             aes(x = p6, y = val, colour = val, group = iter),
             size = 1.5) +
  scale_colour_viridis_c(option = "C") +
  theme_bw() +
  theme(text = element_text(size = 15)) +
  labs(x = "Speed (m per day)",
       y = "-2lnL",
       title = "RFC South speed",
       colour = expression(paste(Delta, "-2lnL <= ", chi^{2}, "(0.95, 1)")))
```

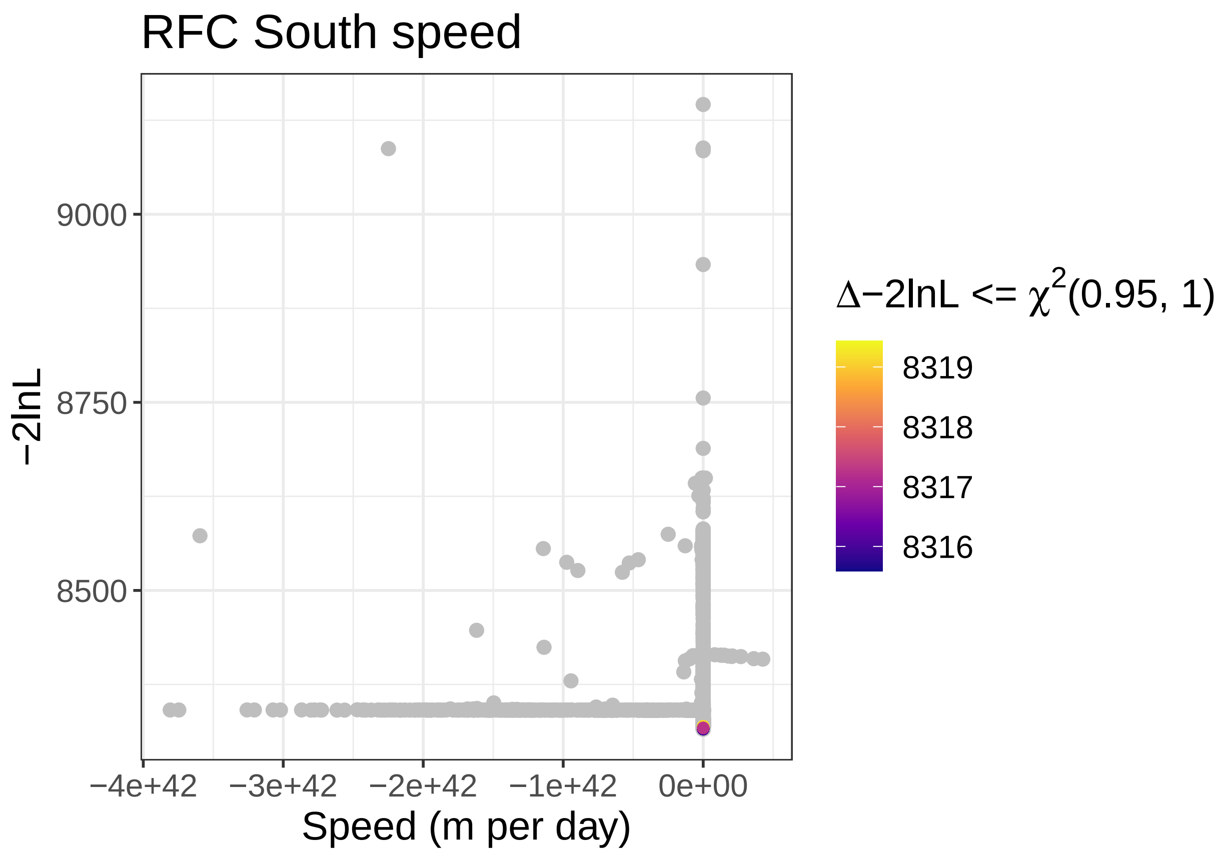

South speed

RDC

```
RDC_out_df$iter <- as.numeric(row.names(RDC_out_df))
RDC_out_df$L <- RDC_out_df$val - min(RDC_out_df$val)

ggplot(RDC_out_df) +
  geom_point(data = space, aes(x = cen.x, y = cen.y),
             colour = "black", size = 0.5, shape = 2, alpha = 0.2) +
  geom_point(aes(x = p1, y = p2, group = iter),
             size = 2, colour = "grey") +
  geom_point(data = RDC_out_df[RDC_out_df$L <= qchisq(0.95, 2),],
             aes(x = p1, y = p2, colour = val, group = iter),
             size = 1.5) +
  geom_point(aes(x = p4, y = p5, group = iter),
             size = 2, colour = "grey") +
  geom_point(data = RDC_out_df[RDC_out_df$L <= qchisq(0.95, 2),],
             aes(x = p4, y = p5, colour = val, group = iter),
             size = 1.5) +
  scale_colour_viridis_c(option = "C") +
  theme_bw() +
  theme(text = element_text(size = 15)) +
  labs(x = "X UTM (centered)",
       y = "Y UTM (centered)",
```

```
       title = "RDC Centroid location",
       colour = expression(paste(Delta, "-2lnL <= ", chi^{2}, "(0.95, 2)")))
```

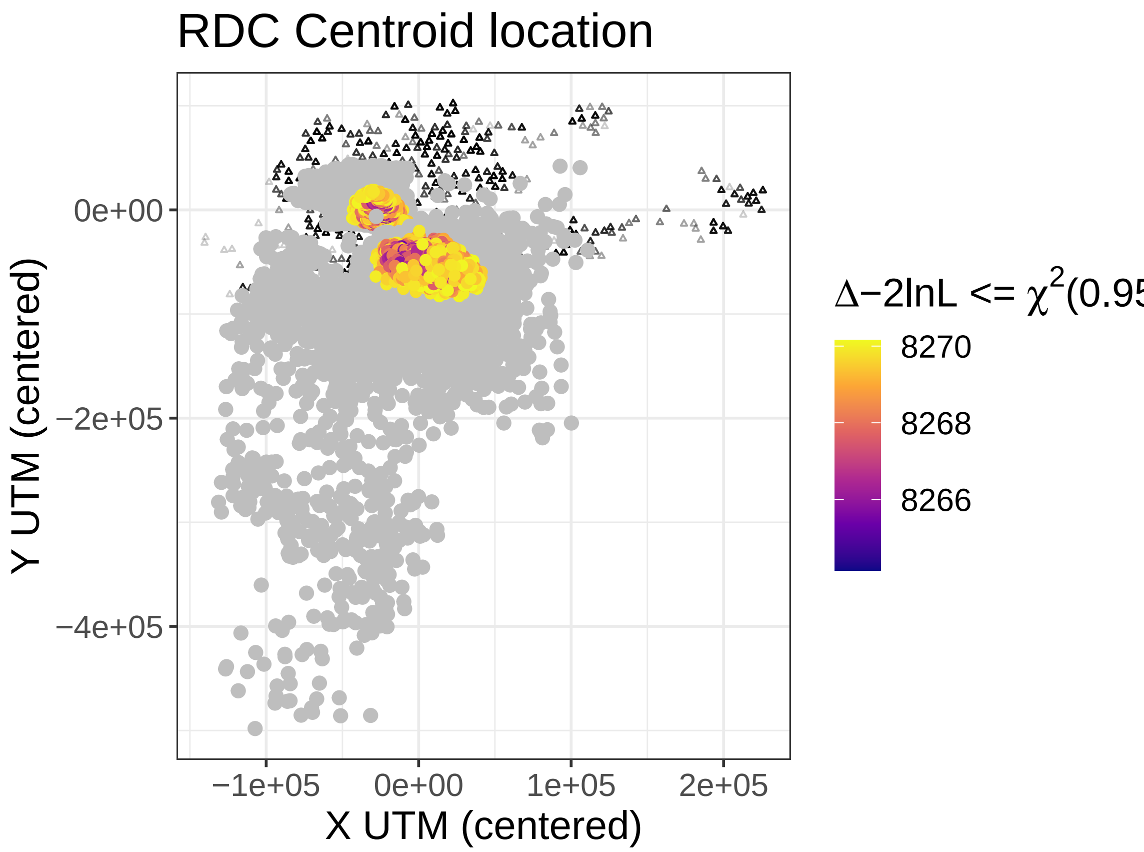

Both wave centroids

```
ggplot(RDC_out_df) +
  geom_point(aes(x = p3, y = val, group = iter),
             size = 2, colour = "grey") +
  geom_point(data = RDC_out_df[RDC_out_df$L <= qchisq(0.95, 1),],
             aes(x = p3, y = val, colour = val, group = iter),
             size = 1.5) +
  scale_colour_viridis_c(option = "C") +
  theme_bw() +
  theme(text = element_text(size = 15)) +
  labs(x = "Speed (m per day)",
       y = "-2lnL",
       title = "RDC Activator speed",
       colour = expression(paste(Delta, "-2lnL <= ", chi^{2}, "(0.95, 1)")))
```

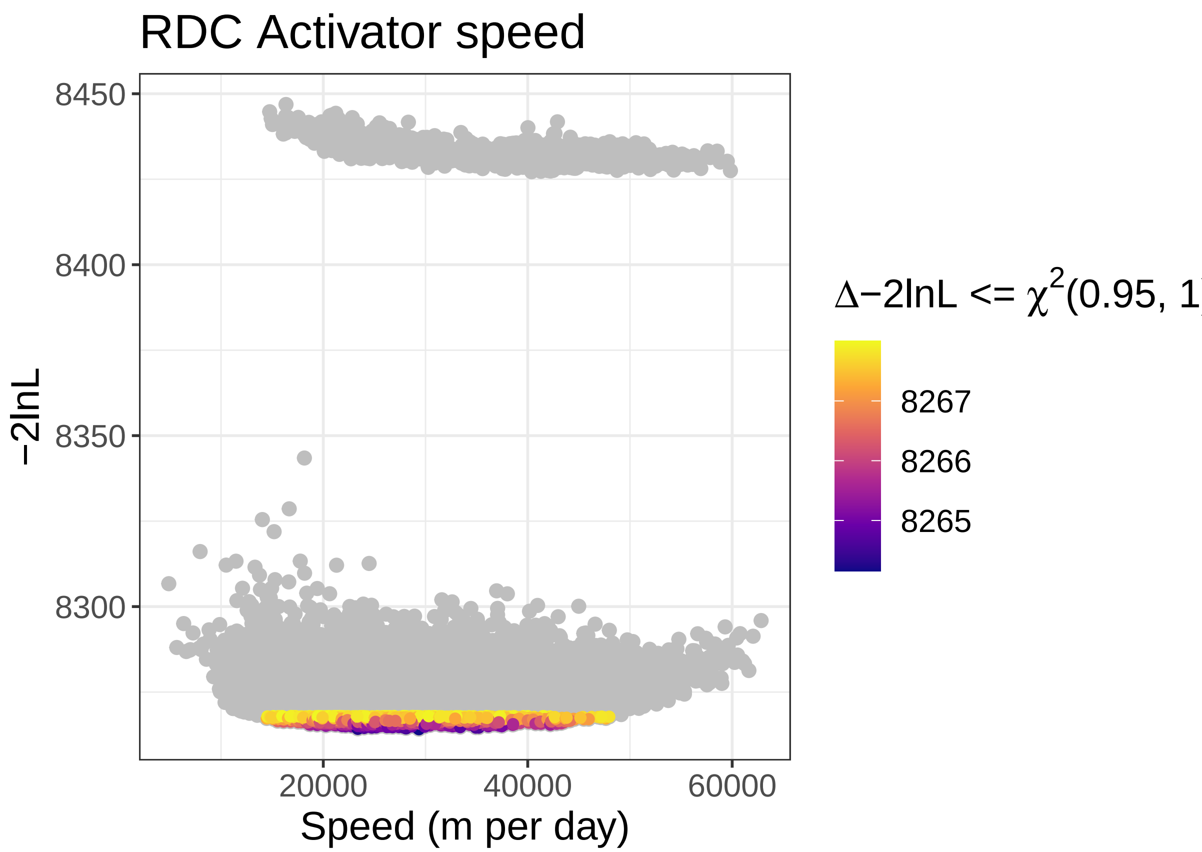

Activator speed

```
ggplot(RDC_out_df) +
  geom_point(aes(x = p6, y = val, group = iter),
             size = 2, colour = "grey") +
  geom_point(data = RDC_out_df[RDC_out_df$L <= qchisq(0.95, 1),],
             aes(x = p6, y = val, colour = val, group = iter),
             size = 1.5) +
  scale_colour_viridis_c(option = "C") +
  theme_bw() +
  theme(text = element_text(size = 15)) +
  labs(x = "Speed (m per day)",
       y = "-2lnL",
       title = "RDC Inhibitor speed",
       colour = expression(paste(Delta, "-2lnL <= ", chi^{2}, "(0.95, 1)")))
```

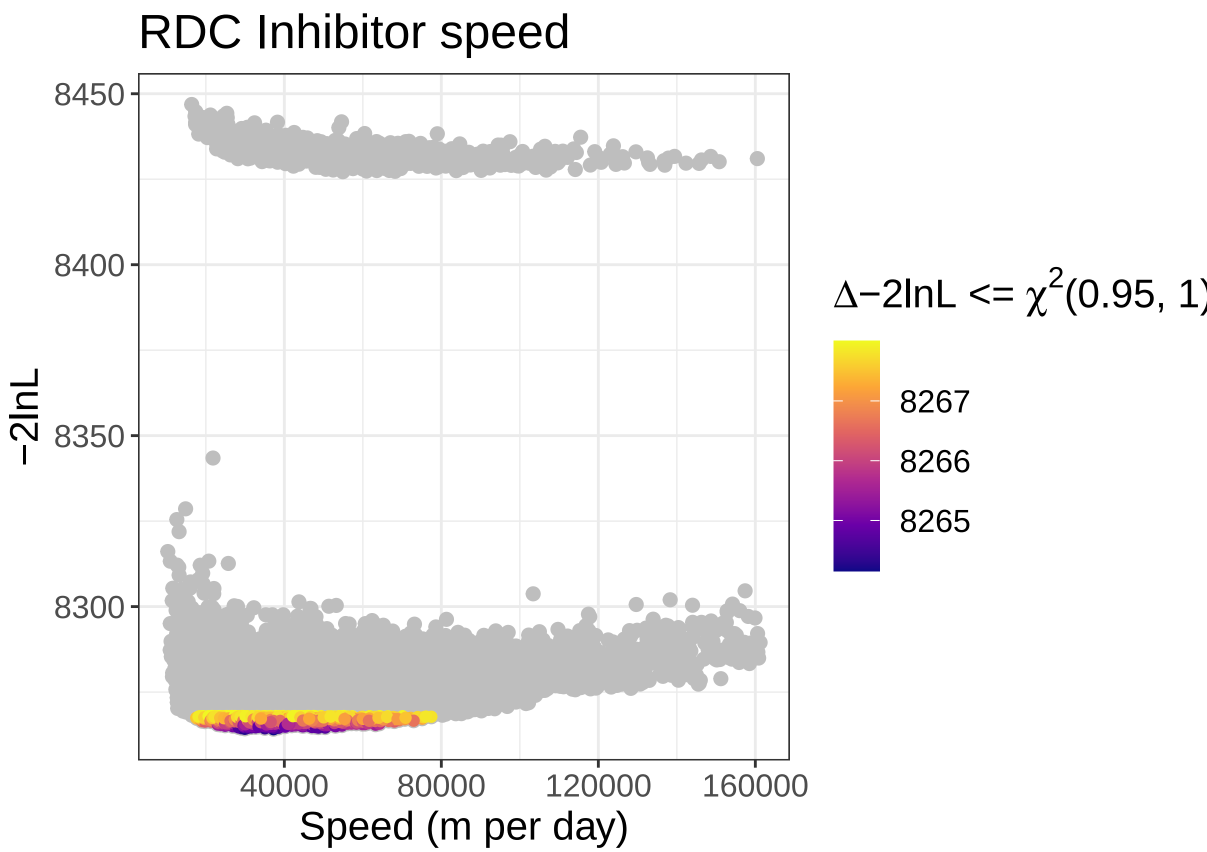

Inhibitor speed

PF

```
PF_out_df$iter <- as.numeric(row.names(PF_out_df))
PF_out_df$L <- PF_out_df$val - min(PF_out_df$val)

ggplot(PF_out_df) +
  geom_point(aes(x = p1, y = val, group = iter),
             size = 2, colour = "grey") +
  geom_point(data = PF_out_df[PF_out_df$L <= qchisq(0.95, 1),],
             aes(x = p1, y = val, colour = val, group = iter),
             size = 2) +
  scale_colour_viridis_c(option = "C") +
  theme_bw() +
  theme(text = element_text(size = 15)) +
  labs(x = "North angle (radian)",
       y = "-2lnL",
       title = "PF North angle",
       colour = expression(paste(Delta, "-2lnL <= ", chi^{2}, "(0.95, 1)")))
```

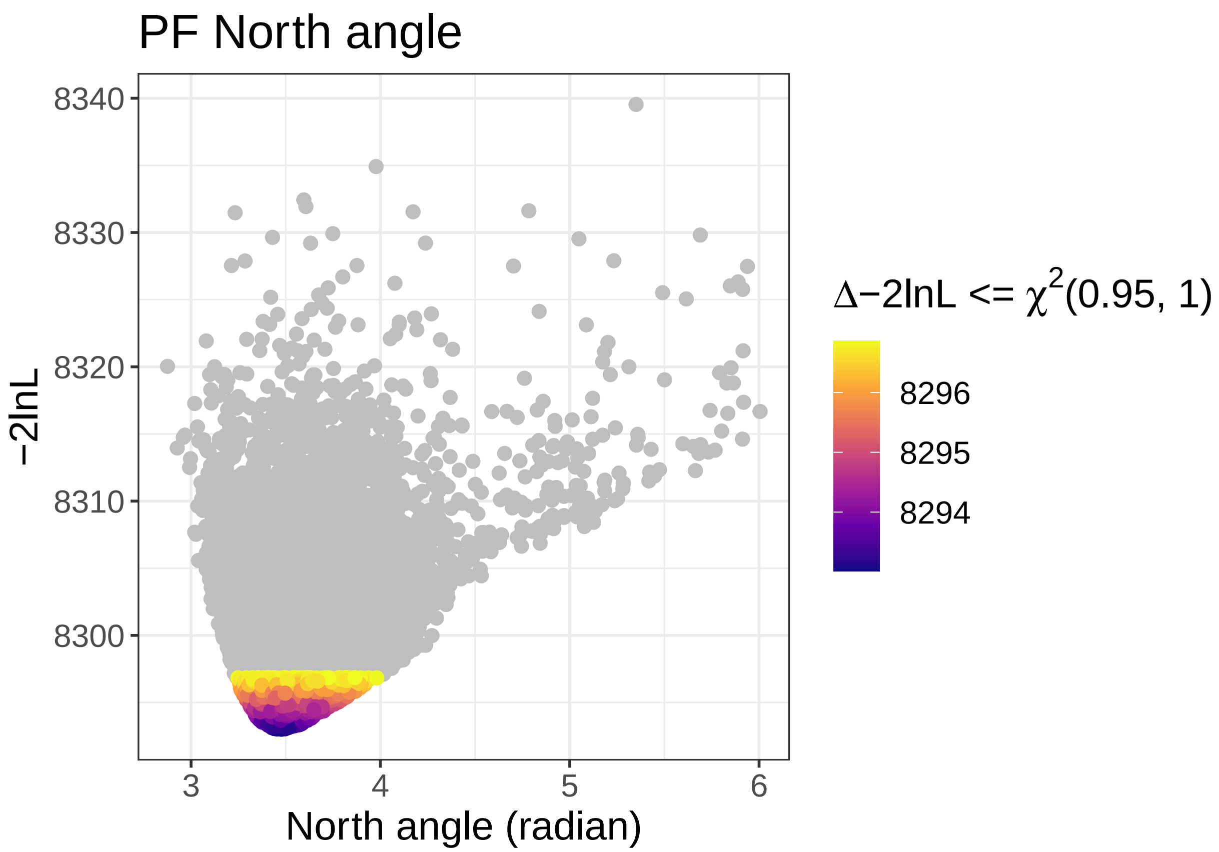

North angle

```
ggplot(PF_out_df) +
  geom_point(aes(x = p2, y = val, group = iter),
             size = 2, colour = "grey") +
  geom_point(data = PF_out_df[PF_out_df$L <= qchisq(0.95, 1),],
             aes(x = p2, y = val, colour = val, group = iter),
             size = 2) +
  scale_colour_viridis_c(option = "C") +
  theme_bw() +
  theme(text = element_text(size = 15)) +
  labs(x = "North speed (m per day)",
       y = "-2lnL",
       title = "PF North speed",
       colour = expression(paste(Delta, "-2lnL <= ", chi^{2}, "(0.95, 1)")))
```

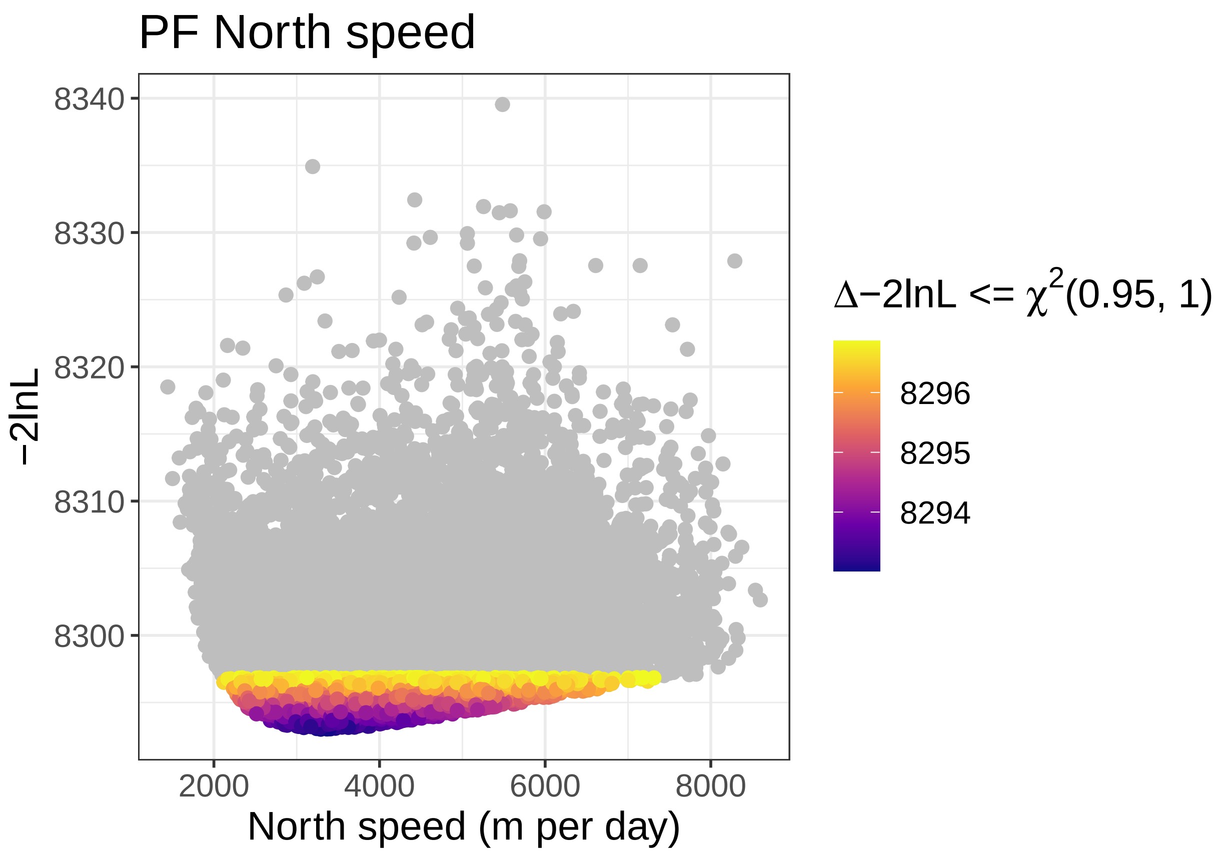

North speed

```
ggplot(PF_out_df) +
  geom_point(aes(x = p3, y = val, group = iter),
             size = 2, colour = "grey") +
  geom_point(data = PF_out_df[PF_out_df$L <= qchisq(0.95, 1),],
             aes(x = p3, y = val, colour = val, group = iter),
             size = 2) +
  scale_colour_viridis_c(option = "C") +
  theme_bw() +
  theme(text = element_text(size = 15)) +
  labs(x = "South angle (radian)",
       y = "-2lnL",
       title = "PF South angle",
       colour = expression(paste(Delta, "-2lnL <= ", chi^{2}, "(0.95, 1)")))
```

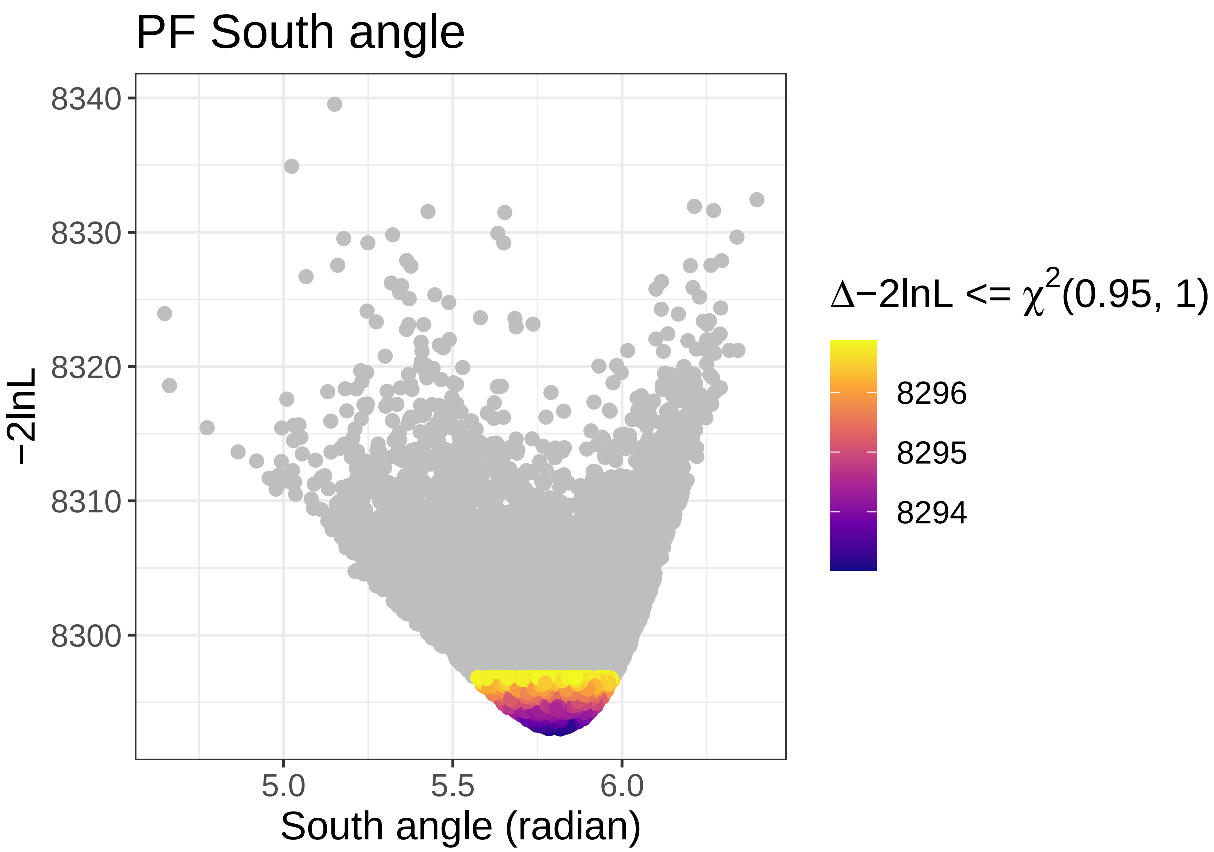

South angle

```
ggplot(PF_out_df) +
  geom_point(aes(x = p4, y = val, group = iter),
             size = 2, colour = "grey") +
  geom_point(data = PF_out_df[PF_out_df$L <= qchisq(0.95, 1),],
             aes(x = p4, y = val, colour = val, group = iter),
             size = 2) +
  scale_colour_viridis_c(option = "C") +
  theme_bw() +
  theme(text = element_text(size = 15)) +
  labs(x = "South speed (m per day)",
       y = "-2lnL",
       title = "PF South speed",
       colour = expression(paste(Delta, "-2lnL <= ", chi^{2}, "(0.95, 1)")))
```

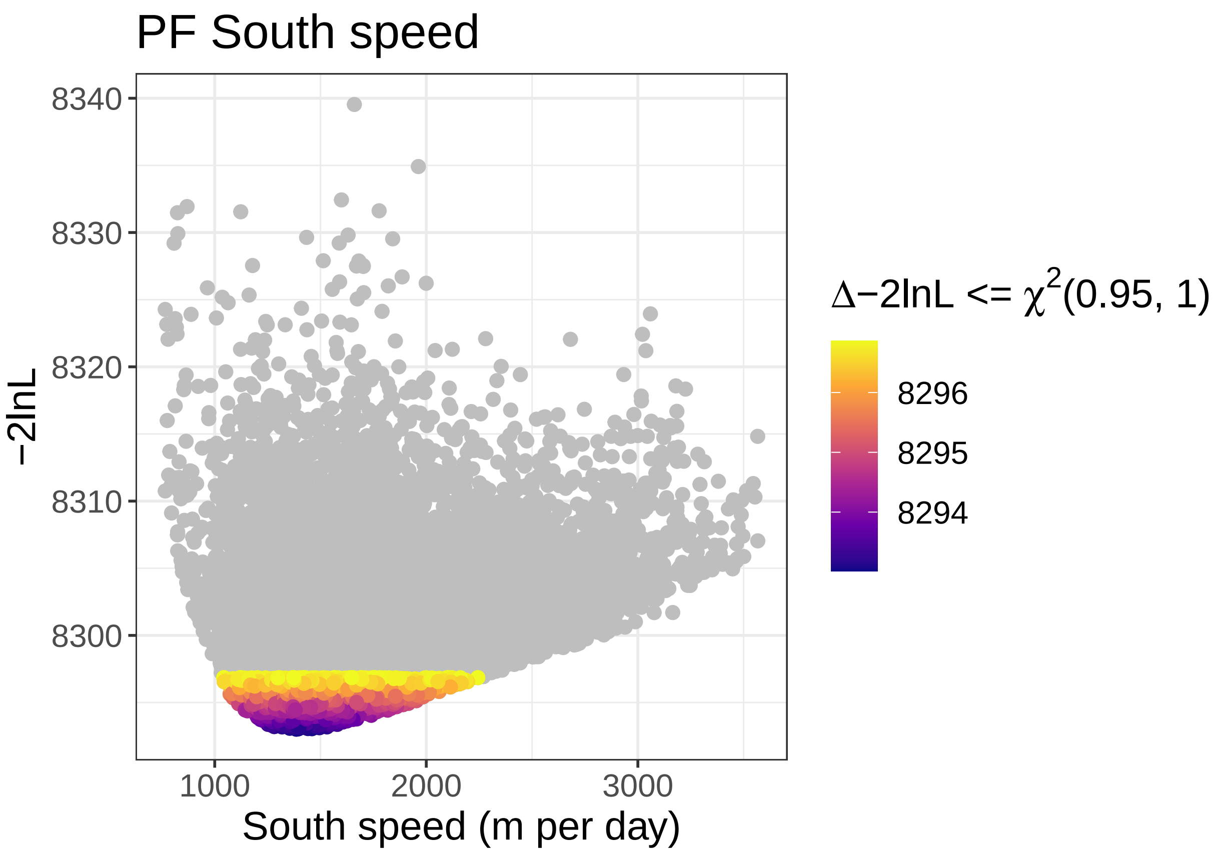

South speed

PD

```
PD_out_df$iter <- as.numeric(row.names(PD_out_df))
PD_out_df$L <- PD_out_df$val - min(PD_out_df$val)

ggplot(PD_out_df) +
  geom_point(aes(x = p1, y = val, group = iter),
             size = 2, colour = "grey") +
  geom_point(data = PD_out_df[PD_out_df$L <= qchisq(0.95, 1),],
             aes(x = p1, y = val, colour = val, group = iter),
             size = 2) +
  scale_colour_viridis_c(option = "C") +
  theme_bw() +
  theme(text = element_text(size = 15)) +
  labs(x = "Activator angle (radian)",
       y = "-2lnL",
       title = "PD Activator angle",
       colour = expression(paste(Delta, "-2lnL <= ", chi^{2}, "(0.95, 1)")))
```

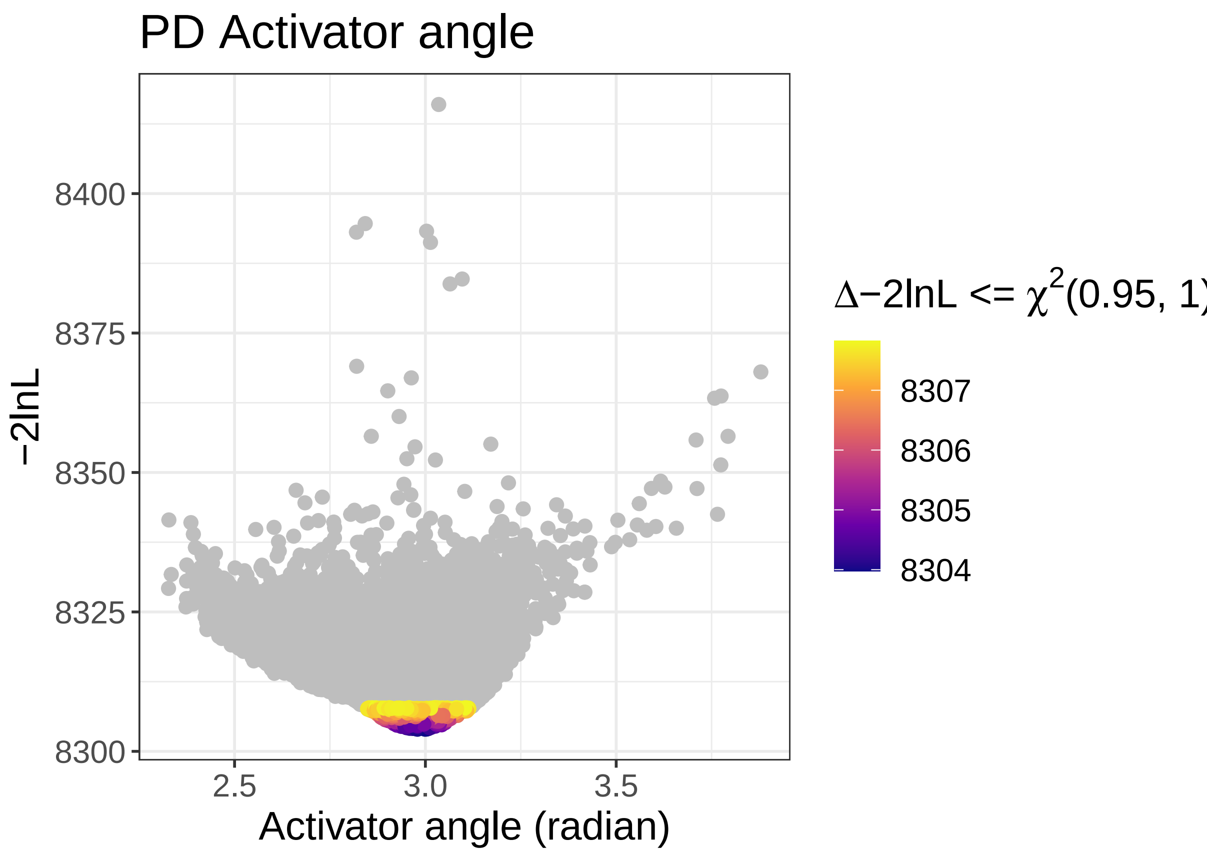

Activator angle

```
ggplot(PD_out_df) +
  geom_point(aes(x = p2, y = val),
             colour = "grey", size = 2) +
  geom_point(data = PD_out_df[PD_out_df$L <= qchisq(0.95, 1),],
             aes(x = p2, y = val, colour = val, group = iter),
             size = 2) +
  scale_colour_viridis_c(option = "C") +
  theme_bw() +
  theme(text = element_text(size = 15)) +
  labs(x = "North speed (km per day)",
       y = "-2lnL",
       title = "PD Activator speed",
       colour = expression(paste(Delta, "-2lnL <= ", chi^{2}, "(0.95, 1)")))
```

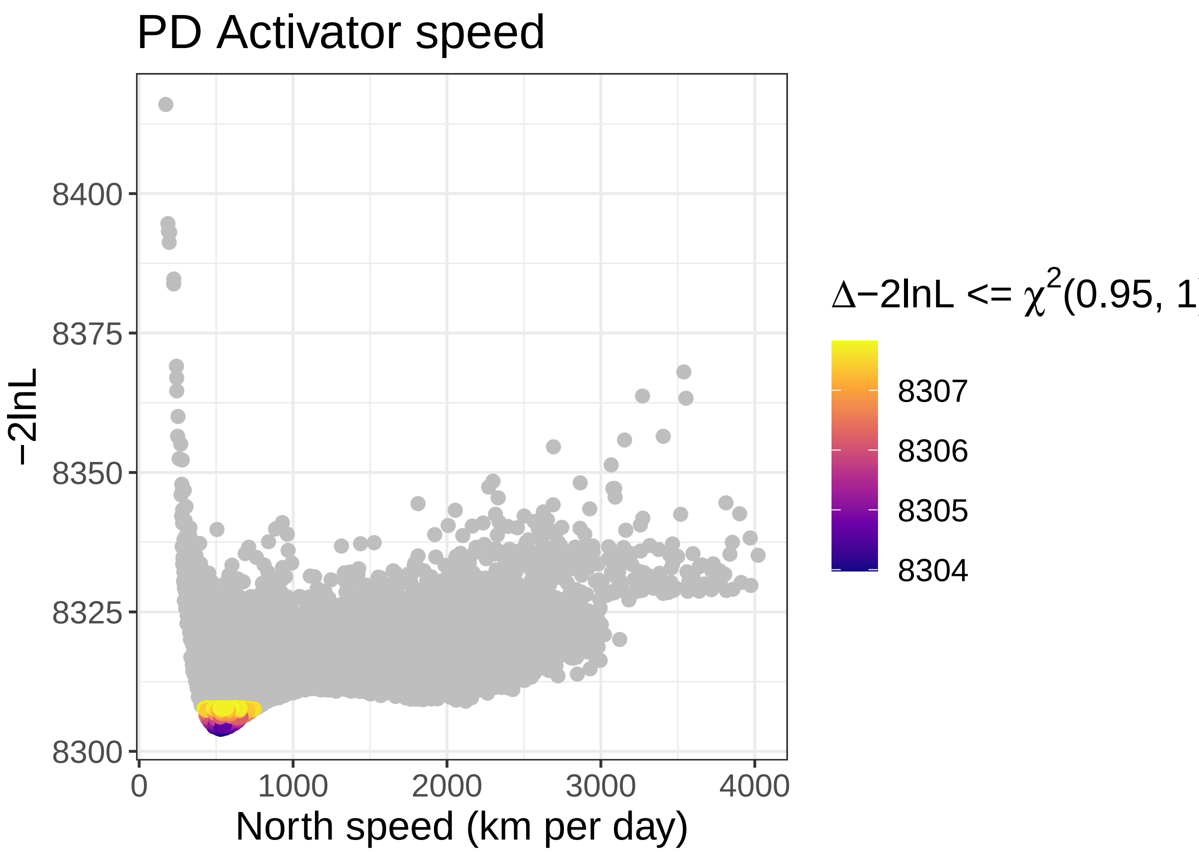

Activator speed

```
ggplot(PD_out_df) +
  geom_point(aes(x = p3, y = val, group = iter),
             size = 2, colour = "grey") +
  geom_point(data = PD_out_df[PD_out_df$L <= qchisq(0.95, 1),],
             aes(x = p3, y = val, colour = val, group = iter),
             size = 2) +
  scale_colour_viridis_c(option = "C") +
  theme_bw() +
  theme(text = element_text(size = 15)) +
  labs(x = "Inhibitor angle (radian)",
       y = "-2lnL",
       title = "PD Inhibitor angle",
       colour = expression(paste(Delta, "-2lnL <= ", chi^{2}, "(0.95, 1)")))
```

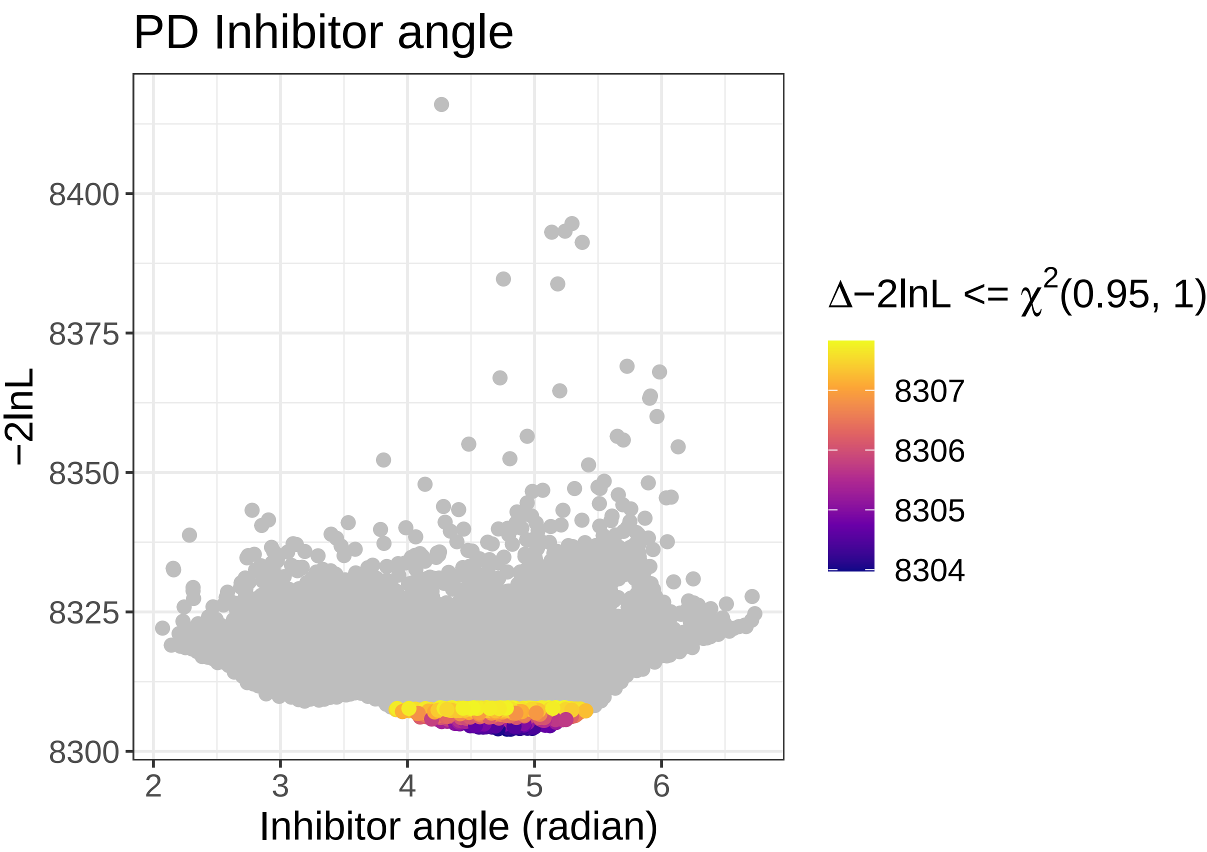

Inhibitor angle

```
ggplot(PD_out_df) +
  geom_point(aes(x = p4, y = val, group = iter),
             size = 2, colour = "grey") +
  geom_point(data = PD_out_df[PD_out_df$L <= qchisq(0.95, 1),],
             aes(x = p4, y = val, colour = val, group = iter),
             size = 2) +
  scale_colour_viridis_c(option = "C") +
  theme_bw() +
  theme(text = element_text(size = 15)) +
  labs(x = "Inhibitor speed (km per day)",
       y = "-2lnL",
       title = "PD Inhibitor speed",
       colour = expression(paste(Delta, "-2lnL <= ", chi^{2}, "(0.95, 1)")))
```

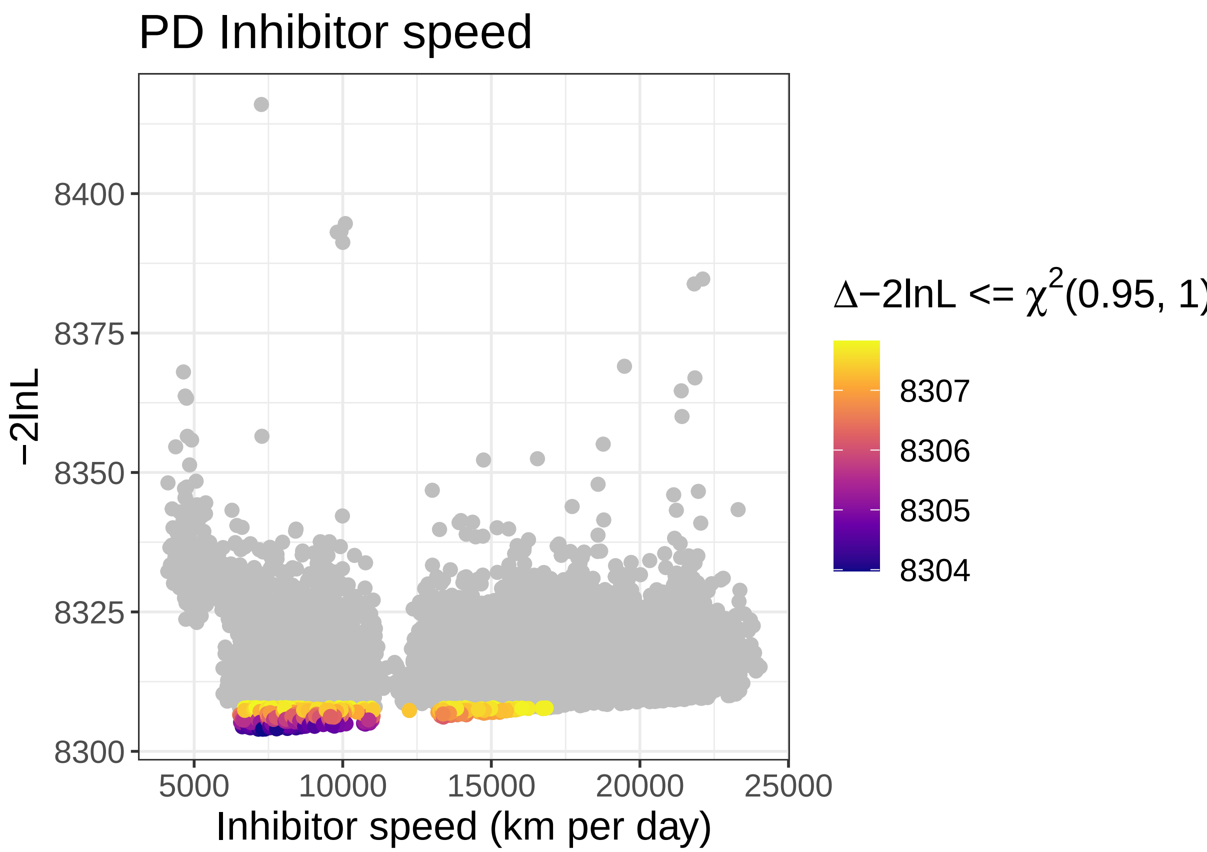

Inhibitor speed

Predictions

Resolution of figures:

```
reso <- c(50, 50, 9)
```

N2

```
plot(N2, pages = 1, shade = TRUE, shade.col = "lightgrey", seWithMean = TRUE, residuals = TRUE, rug = F
```

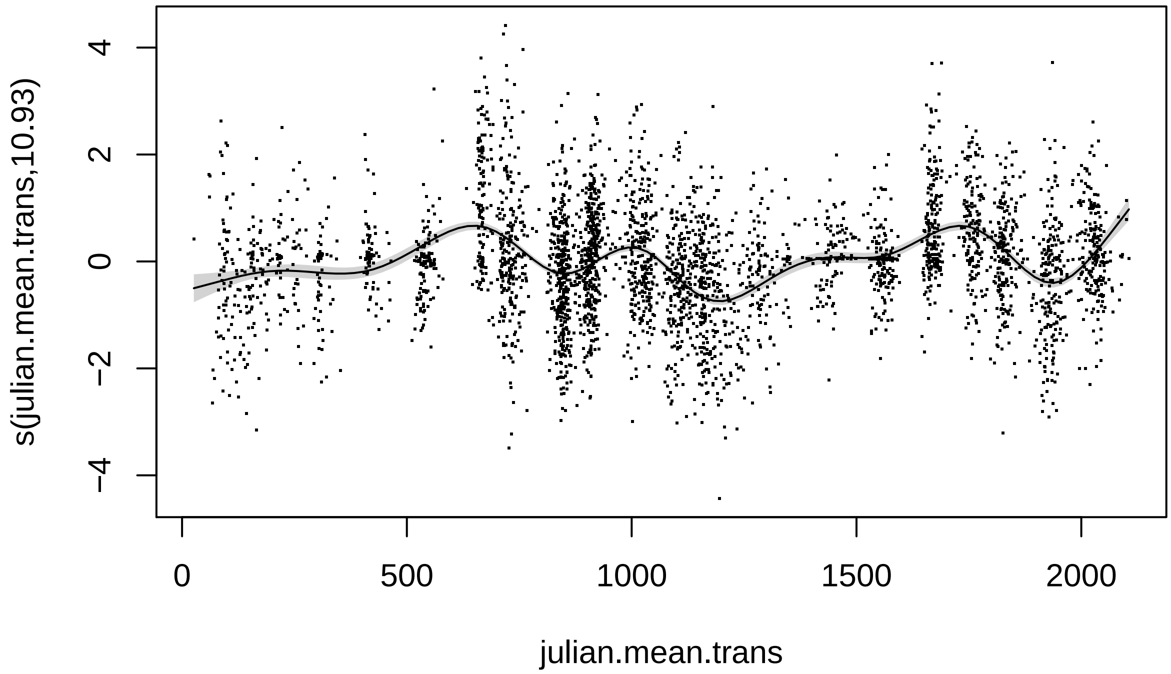

N3

```
p_spatial_N3 <- expand.grid(cen.x = seq(min(space$cen.x),
                                        max(space$cen.x),
                                        length = reso[1]),
                            cen.y = seq(min(space$cen.y),
                                        max(space$cen.y),
                                        length = reso[2]),
                            julian.mean.trans = seq(min(space$julian.mean.trans),
                                                    max(space$julian.mean.trans),
                                                    length = reso[3])
)

N3_fit <- data.frame(predict(N3, newdata = p_spatial_N3, se.fit = TRUE))

ind <- exclude.too.far(p_spatial_N3$cen.x, p_spatial_N3$cen.y,
                       space$cen.x, space$cen.y, dist = 0.1)

N3_fit$fit[ind] <- NA

N3_fit <- transform(N3_fit,
                    upper = fit + (2 * se.fit),
                    lower = fit - (2 * se.fit))
```

```r
pred_N3 <- cbind(p_spatial_N3, N3_fit)

ggplot(data = pred_N3) +
  geom_tile(aes(x = cen.x, y = cen.y, fill = fit)) +
  scale_fill_viridis_c(option = "C", na.value = "transparent") +
  scale_x_continuous(label = scales::comma) +
  scale_y_continuous(label = scales::comma) +
  labs(x = "X",
       y = "Y",
       fill= expression(r[t])) +
  theme_bw() +
  theme(text = element_text(size = 15))
```

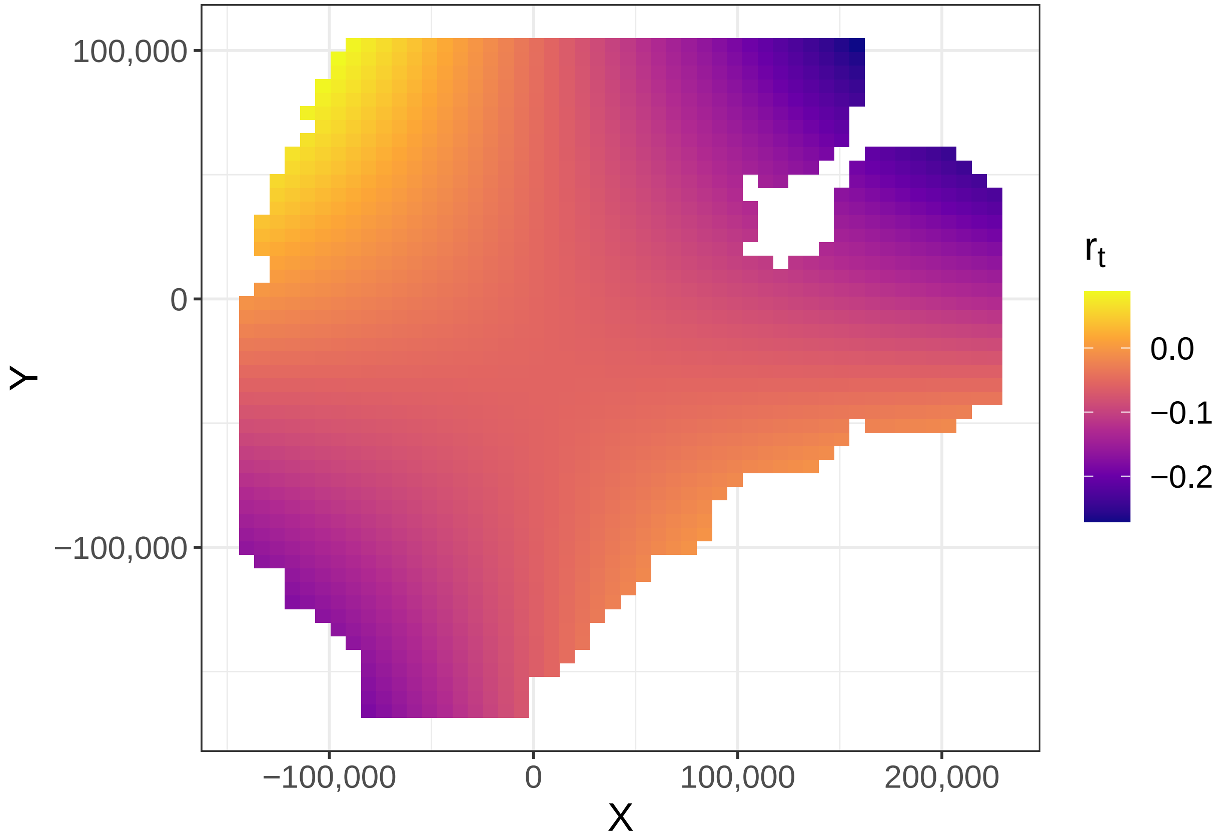

P

```r
space$D <- sin(P_out$estimate[1]) * space$cen.x + cos(P_out$estimate[1]) * space$cen.y
space$rho <- space$julian.mean.trans + (1 / P_out$estimate[2]) * space$D
P_TW <- gam(r.growth ~ s(rho, k = 12, bs = "tp"),
            weights = sqrt_diff_survey,
            method = "ML",
            family = "gaussian",
            data = space)
```

```
plot(P_TW, pages = 1, shade = TRUE, shade.col = "lightgrey", seWithMean = TRUE, residuals = TRUE, rug =
```

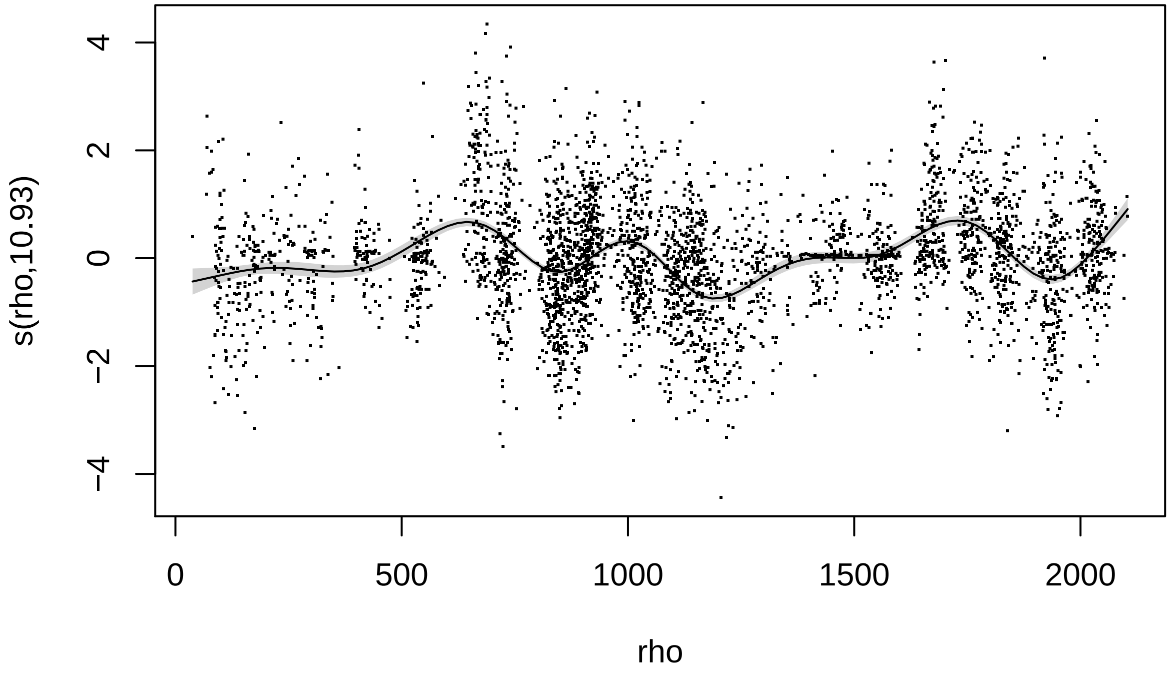

Temporal

```
p_spatial_P <- expand.grid(cen.x = seq(min(space$cen.x),
                                       max(space$cen.x),
                                       length = reso[1]),
                           cen.y = seq(min(space$cen.y),
                                       max(space$cen.y),
                                       length = reso[2]),
                           julian.mean.trans = seq(min(space$julian.mean.trans),
                                                   max(space$julian.mean.trans),
                                                   length = reso[3])
)

p_spatial_P$D <- sin(P_out$estimate[1]) * p_spatial_P$cen.x + cos(P_out$estimate[1]) * p_spatial_P$cen.y
p_spatial_P$rho = p_spatial_P$julian.mean.trans + (1 / P_out$estimate[2]) * p_spatial_P$D

P_fit <- data.frame(predict(P_TW, newdata = p_spatial_P, se.fit = TRUE))

ind <- exclude.too.far(p_spatial_P$cen.x, p_spatial_P$cen.y,
                       space$cen.x, space$cen.y, dist = 0.1)

P_fit$fit[ind] <- NA
```

```
P_fit <- transform(P_fit,
                   upper = fit + (2 * se.fit),
                   lower = fit - (2 * se.fit))

pred_P <- cbind(p_spatial_P, P_fit)

ggplot() +
  geom_tile(data = pred_P, aes(x = cen.x, y = cen.y, fill = fit)) +
  scale_fill_viridis_c(option = "C", na.value = "transparent") +
  scale_x_continuous(label = scales::comma) +
  scale_y_continuous(label = scales::comma) +
  labs(x = "X",
       y = "Y",
       fill= expression(r[t])) +
  theme_bw() +
  theme(text = element_text(size = 15)) +
  facet_wrap(~ round(julian.mean.trans))
```

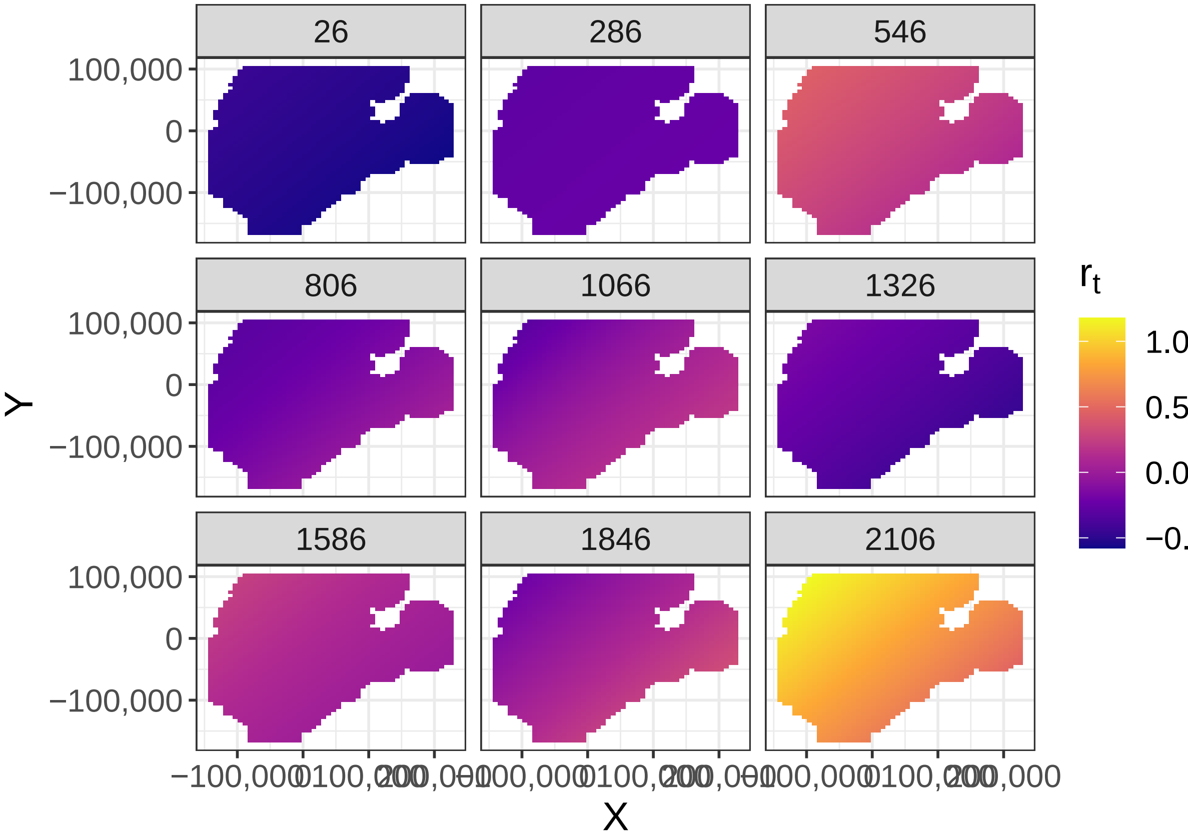

Spatio-temporal

RE

```
space$D <- -sqrt((RE_out$estimate[1] - space$cen.x)^2 + (RE_out$estimate[2] - space$cen.y)^2)
space$rho <- space$julian.mean.trans + (1 / RE_out$estimate[3]) * space$D
```

```
RE <- gam(r.growth ~ s(rho, k = 12, bs = "tp"),
          weights = sqrt_diff_survey,
          method = "ML",
          data = space,
          family = "gaussian")

plot(RE, pages = 1, shade = TRUE, shade.col = "lightgrey", seWithMean = TRUE, residuals = TRUE, rug = F
```

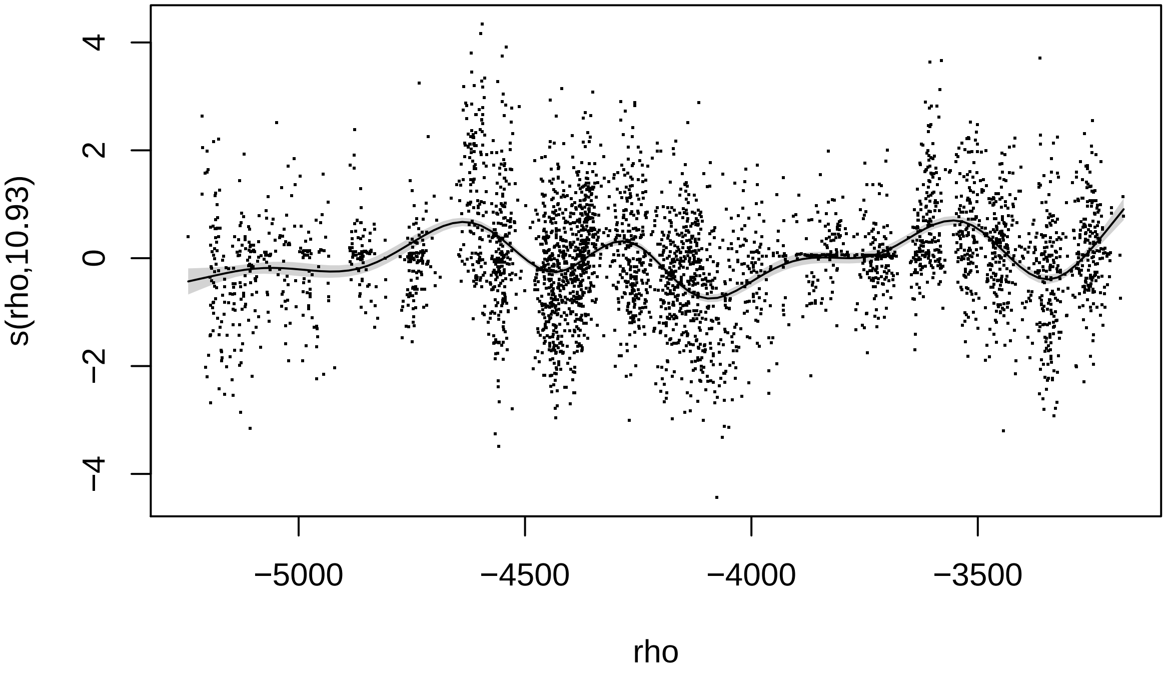

Temporal

```
p_spatial_RE <- expand.grid(cen.x = seq(min(space$cen.x),
                                        max(space$cen.x),
                                        length = reso[1]),
                            cen.y = seq(min(space$cen.y),
                                        max(space$cen.y),
                                        length = reso[2]),
                            julian.mean.trans = seq(min(space$julian.mean.trans),
                                                    max(space$julian.mean.trans),
                                                    length = reso[3])
)

p_spatial_RE$D <- -sqrt((RE_out$estimate[1] - p_spatial_RE$cen.x)^2 + (RE_out$estimate[2] - p_spatial_R
p_spatial_RE$rho <- p_spatial_RE$julian.mean.trans + (1 / RE_out$estimate[3]) * p_spatial_RE$D
```

```r
RE_fit <- data.frame(predict(RE, newdata = p_spatial_RE, se.fit = TRUE))

ind <- exclude.too.far(p_spatial_RE$cen.x, p_spatial_RE$cen.y,
                       space$cen.x, space$cen.y, dist = 0.1)

RE_fit$fit[ind] <- NA

RE_fit <- transform(RE_fit,
                    upper = fit + (2 * se.fit),
                    lower = fit - (2 * se.fit))

pred_RE <- cbind(p_spatial_RE, RE_fit)

ggplot() +
  geom_tile(data = pred_RE, aes(x = cen.x, y = cen.y, fill = fit)) +
  scale_fill_viridis_c(option = "C", na.value = "transparent") +
  scale_x_continuous(label = scales::comma) +
  scale_y_continuous(label = scales::comma) +
  labs(x = "X",
       y = "Y",
       fill= expression(r[t]),
       title = "RE spatial pattern over time") +
  theme_bw() +
  theme(text = element_text(size = 15)) +
  facet_wrap(~ round(julian.mean.trans))
```

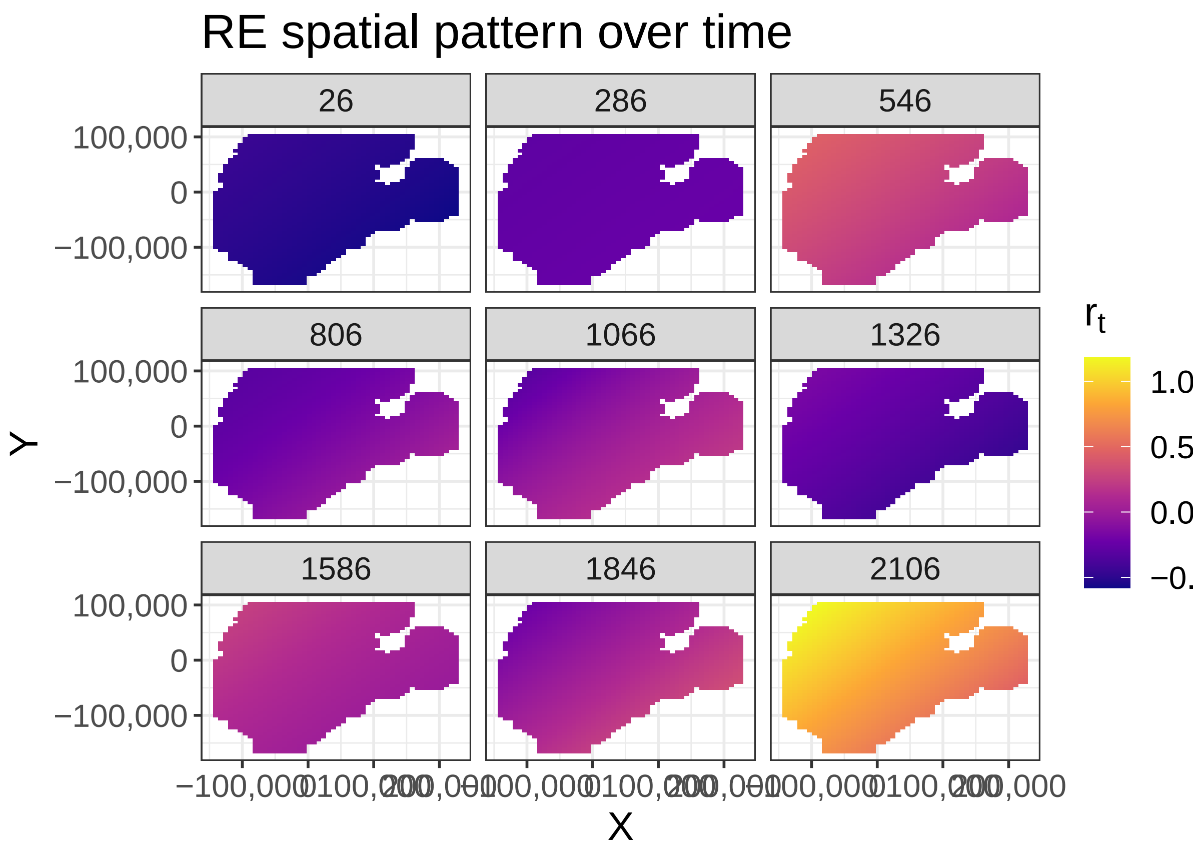

Spatio-temporal

RC

```
space$D <- sqrt((RC_out$estimate[1] - space$cen.x)^2 + (RC_out$estimate[2] - space$cen.y)^2)
space$rho <- space$julian.mean.trans + (1 / RC_out$estimate[3]) * space$D

RC <- gam(r.growth ~ s(rho, k = 12, bs = "tp"),
          weights = sqrt_diff_survey,
          method = "ML",
          data = space,
          family = "gaussian")

plot(RC, pages = 1, shade = TRUE, shade.col = "lightgrey", seWithMean = TRUE, residuals = TRUE, rug = F
```

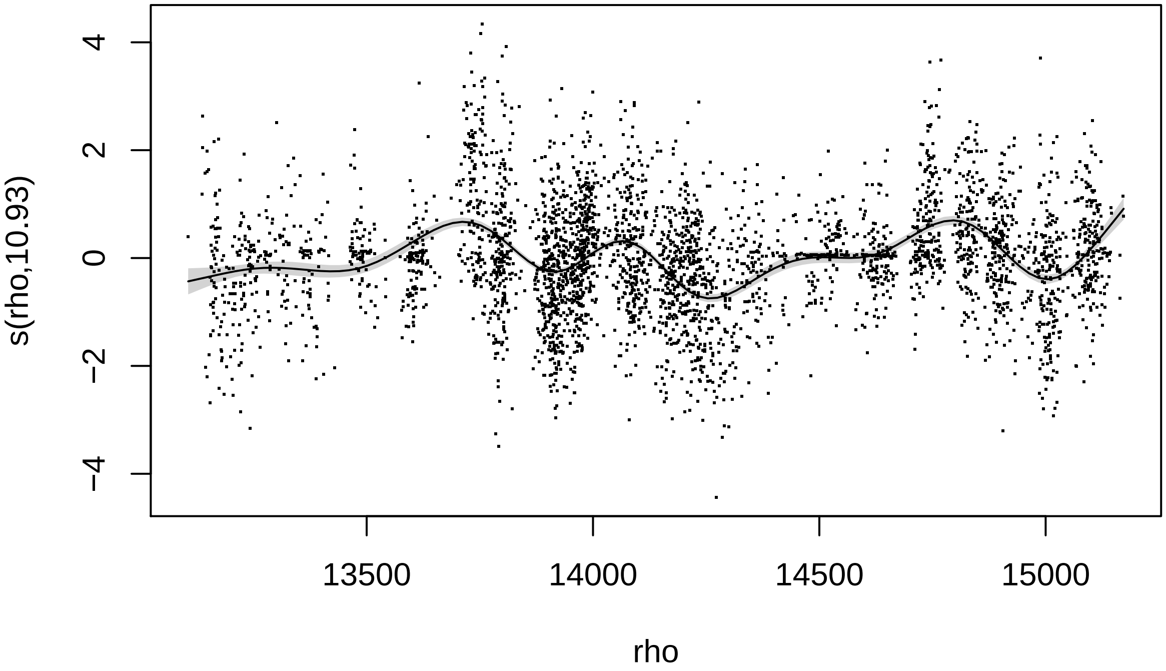

Temporal

```
p_spatial_RC <- expand.grid(cen.x = seq(min(space$cen.x),
                                        max(space$cen.x),
                                        length = reso[1]),
                            cen.y = seq(min(space$cen.y),
                                        max(space$cen.y),
                                        length = reso[2]),
                            julian.mean.trans = seq(min(space$julian.mean.trans),
```

```
                                                    max(space$julian.mean.trans),
                                                    length = reso[3])
)

p_spatial_RC$D <- sqrt((RC_out$estimate[1] - p_spatial_RC$cen.x)^2 + (RE_out$estimate[2] - p_spatial_RC$
p_spatial_RC$rho <- p_spatial_RC$julian.mean.trans + (1 / RE_out$estimate[3]) * p_spatial_RC$D

RC_fit <- data.frame(predict(RC, newdata = p_spatial_RC, se.fit = TRUE))

ind <- exclude.too.far(p_spatial_RC$cen.x, p_spatial_RC$cen.y,
                       space$cen.x, space$cen.y, dist = 0.1)

RC_fit$fit[ind] <- NA

RC_fit <- transform(RC_fit,
                    upper = fit + (2 * se.fit),
                    lower = fit - (2 * se.fit))

pred_RC <- cbind(p_spatial_RC, RC_fit)

ggplot() +
  geom_tile(data = pred_RC, aes(x = cen.x, y = cen.y, fill = fit)) +
  scale_fill_viridis_c(option = "C", na.value = "transparent") +
  scale_x_continuous(label = scales::comma) +
  scale_y_continuous(label = scales::comma) +
  labs(x = "X",
       y = "Y",
       title = "RC spatial pattern over time",
       fill= expression(r[t])) +
  theme_bw() +
  theme(text = element_text(size = 15)) +
  facet_wrap(~ round(julian.mean.trans))
```

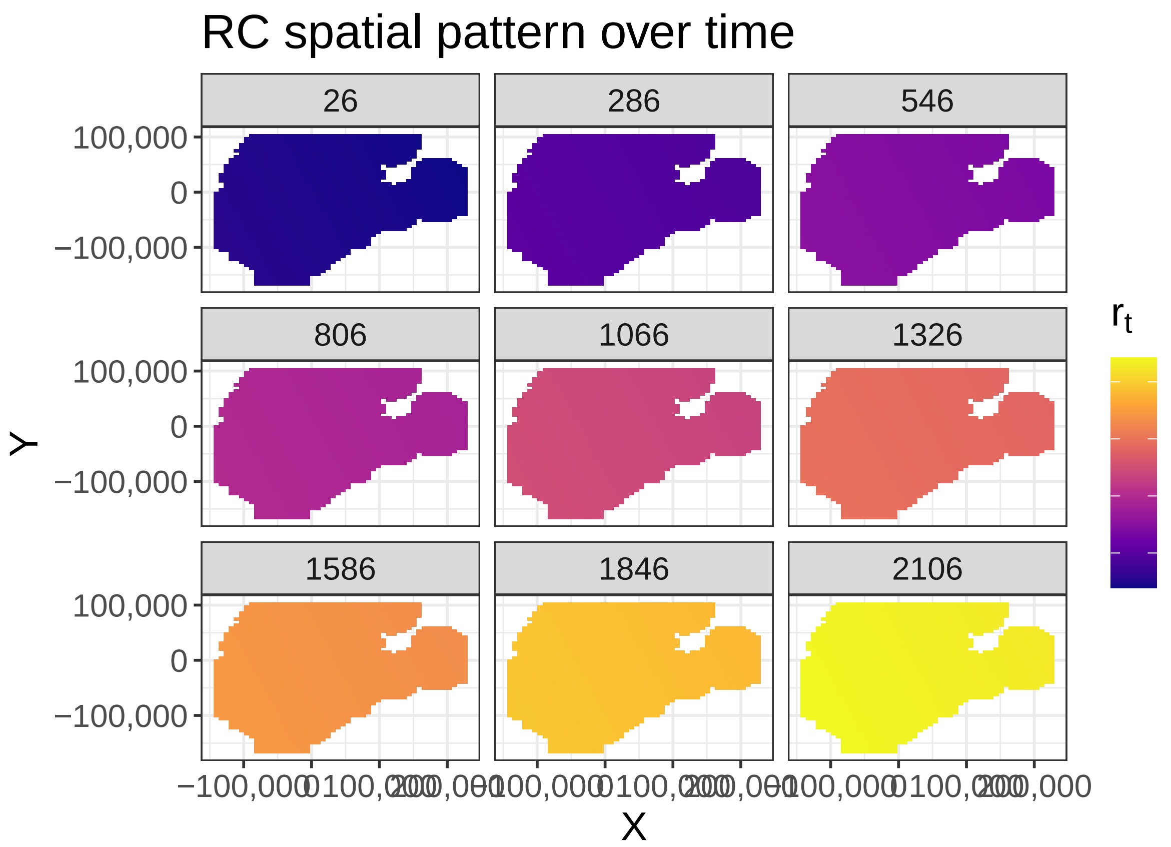

Spatio-temporal

RFE

```
space$D1 <- 1
space$rho1 <- 1
space$D2 <- 1
space$rho2 <- 1

for(i in 1:nrow(space)){
  if (space$north.south[i] == "north") {
    space$D1[i] <- -sqrt((RFE_out$estimate[1] - space$cen.x[i])^2 + (RFE_out$estimate[2] - space$cen.y[
    space$rho1[i] <- space$julian.mean.trans[i] + (1 / RFE_out$estimate[3]) * space$D1[i]
  } else {
    space$D2[i] <- -sqrt((RFE_out$estimate[4] - space$cen.x[i])^2 + (RFE_out$estimate[5] - space$cen.y[
    space$rho2[i] <- space$julian.mean.trans[i] + (1 / RFE_out$estimate[6]) * space$D2[i]
  }
}

RFE <- gam(r.growth ~ s(rho1, by = north, k = 12, bs = "tp") + s(rho2, by = south, k = 12, bs = "tp"),
           weights = sqrt_diff_survey,
           method = "ML",
           data = space,
           family = "gaussian")
```

```r
plot(RFE, pages = 1, shade = TRUE, shade.col = "lightgrey", seWithMean = TRUE, residuals = TRUE, rug = 
```

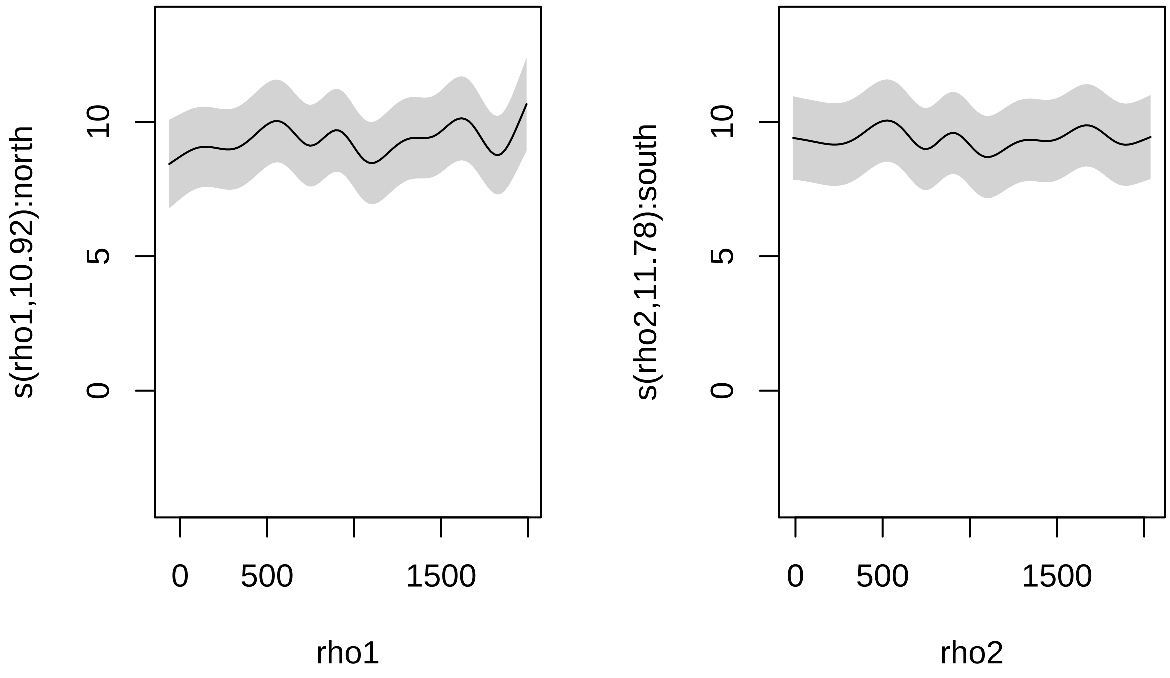

Temporal

```r
p_spatial_RFE <- expand.grid(cen.x = seq(min(space$cen.x),
                                         max(space$cen.x),
                                         length = reso[1]),
                             cen.y = seq(min(space$cen.y),
                                         max(space$cen.y),
                                         length = reso[2]),
                             julian.mean.trans = seq(min(space$julian.mean.trans),
                                                     max(space$julian.mean.trans),
                                                     length = reso[3])
)

p_spatial_RFE$north.south <- NA
p_spatial_RFE$north.south <- ifelse(p_spatial_RFE$cen.y > 0, "north", "south") # 41.8 latitude, 2590 tr
p_spatial_RFE$north.south <- as.factor(p_spatial_RFE$north.south)

p_spatial_RFE$D1 <- 1
p_spatial_RFE$rho1 <- 1
p_spatial_RFE$D2 <- 1
p_spatial_RFE$rho2 <- 1
```

```
for(i in 1:nrow(p_spatial_RFE)){
  if (p_spatial_RFE$north.south[i] == "north") {
    p_spatial_RFE$D1[i] <- -sqrt((RFE_out$estimate[1] - p_spatial_RFE$cen.x[i])^2 + (RFE_out$estimate[2
    p_spatial_RFE$rho1[i] <- p_spatial_RFE$julian.mean.trans[i] + (1 / RFE_out$estimate[3]) * p_spatial_
  } else {
    p_spatial_RFE$D2[i] <- -sqrt((RFE_out$estimate[4] - p_spatial_RFE$cen.x[i])^2 + (RFE_out$estimate[5
    p_spatial_RFE$rho2[i] <- p_spatial_RFE$julian.mean.trans[i] + (1 / RFE_out$estimate[6]) * p_spatial_
  }
}

p_spatial_RFE$north <- NA
p_spatial_RFE$south <- NA

p_spatial_RFE$north <- ifelse(p_spatial_RFE$north.south == "north", 1, 0)
p_spatial_RFE$south <- ifelse(p_spatial_RFE$north.south == "south", 1, 0)

RFE_fit <- data.frame(predict(RFE, newdata = p_spatial_RFE, se.fit = TRUE))

ind <- exclude.too.far(p_spatial_RFE$cen.x, p_spatial_RFE$cen.y,
                       space$cen.x, space$cen.y, dist = 0.1)

RFE_fit$fit[ind] <- NA

RFE_fit <- transform(RFE_fit,
                     upper = fit + (2 * se.fit),
                     lower = fit - (2 * se.fit))

pred_RFE <- cbind(p_spatial_RFE, RFE_fit)

ggplot() +
  geom_tile(data = pred_RFE, aes(x = cen.x, y = cen.y, fill = fit)) +
  scale_fill_viridis_c(option = "C", na.value = "transparent") +
  scale_x_continuous(label = scales::comma) +
  scale_y_continuous(label = scales::comma) +
  labs(x = "X",
       y = "Y",
       title = "RFE spatial pattern over time",
       fill= expression(r[t])) +
  theme_bw() +
  theme(text = element_text(size = 15)) +
  facet_wrap(~ round(julian.mean.trans))
```

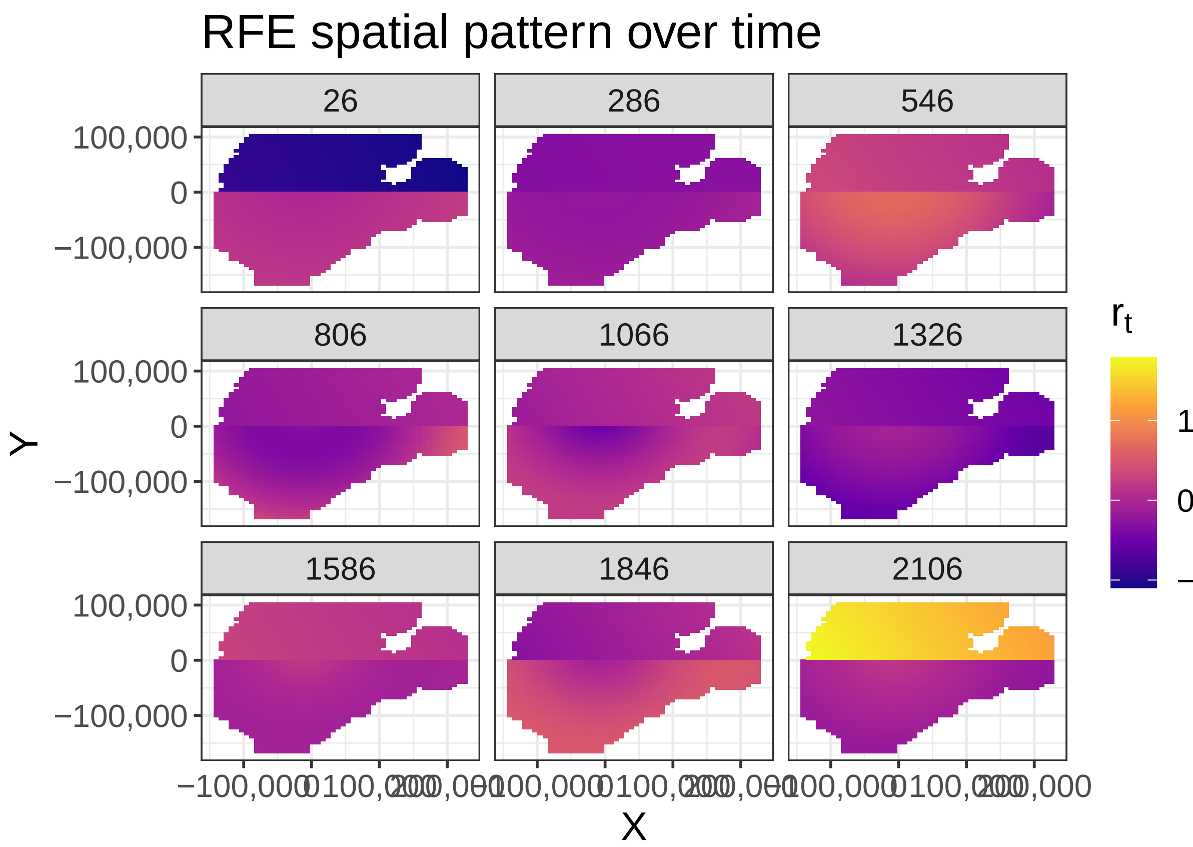

Spatio-temporal

RDE

```
space$D1 <- -sqrt((RDE_out$estimate[1] - space$cen.x)^2 + (RDE_out$estimate[2] - space$cen.y)^2)
space$rho1 <- space$julian.mean.trans + (1 / RDE_out$estimate[3]) * space$D1

space$D2 <- -sqrt((RDE_out$estimate[4] - space$cen.x)^2 + (RDE_out$estimate[5] - space$cen.y)^2)
space$rho2 <- space$julian.mean.trans + (1 / RDE_out$estimate[6]) * space$D2

RDE <- gam(r.growth ~ s(rho1, k = 12, bs = "tp") + s(rho2, k = 12, bs = "tp"),
           weights = sqrt_diff_survey,
           method = "ML",
           data = space,
           family = "gaussian")

plot(RDE, pages = 1, shade = TRUE, shade.col = "lightgrey", seWithMean = TRUE, residuals = TRUE, rug = 
```

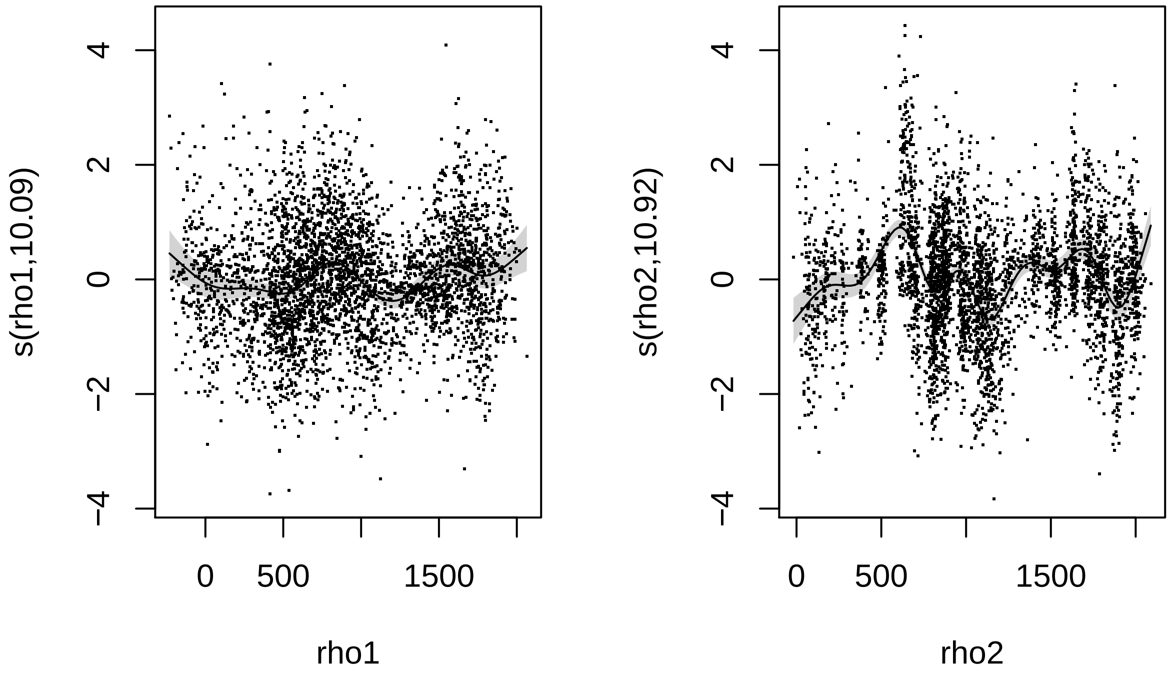

Temporal

```
p_spatial_RDE <- expand.grid(cen.x = seq(min(space$cen.x),
                                         max(space$cen.x),
                                         length = reso[1]),
                             cen.y = seq(min(space$cen.y),
                                         max(space$cen.y),
                                         length = reso[2]),
                             julian.mean.trans = seq(min(space$julian.mean.trans),
                                                     max(space$julian.mean.trans),
                                                     length = reso[3])
)

p_spatial_RDE$D1 <- -sqrt((RDE_out$estimate[1] - p_spatial_RDE$cen.x)^2 + (RDE_out$estimate[2] - p_spati
p_spatial_RDE$rho1 <- p_spatial_RDE$julian.mean.trans + (1 / RDE_out$estimate[3]) * p_spatial_RDE$D1

p_spatial_RDE$D2 <- -sqrt((RDE_out$estimate[4] - p_spatial_RDE$cen.x)^2 + (RDE_out$estimate[5] - p_spati
p_spatial_RDE$rho2 <- p_spatial_RDE$julian.mean.trans + (1 / RDE_out$estimate[6]) * p_spatial_RDE$D2

RDE_fit <- data.frame(predict(RDE, newdata = p_spatial_RDE, se.fit = TRUE))

ind <- exclude.too.far(p_spatial_RDE$cen.x, p_spatial_RDE$cen.y,
                       space$cen.x, space$cen.y, dist = 0.1)

RDE_fit$fit[ind] <- NA
```

```r
RDE_fit <- transform(RDE_fit,
                     upper = fit + (2 * se.fit),
                     lower = fit - (2 * se.fit))

pred_RDE <- cbind(p_spatial_RDE, RDE_fit)

ggplot() +
  geom_tile(data = pred_RDE, aes(x = cen.x, y = cen.y, fill = fit)) +
  scale_fill_viridis_c(option = "C", na.value = "transparent") +
  scale_x_continuous(label = scales::comma) +
  scale_y_continuous(label = scales::comma) +
  labs(x = "X",
       y = "Y",
       title = "RDE spatial pattern over time",
       fill= expression(r[t])) +
  theme_bw() +
  theme(text = element_text(size = 15)) +
  facet_wrap(~ round(julian.mean.trans))
```

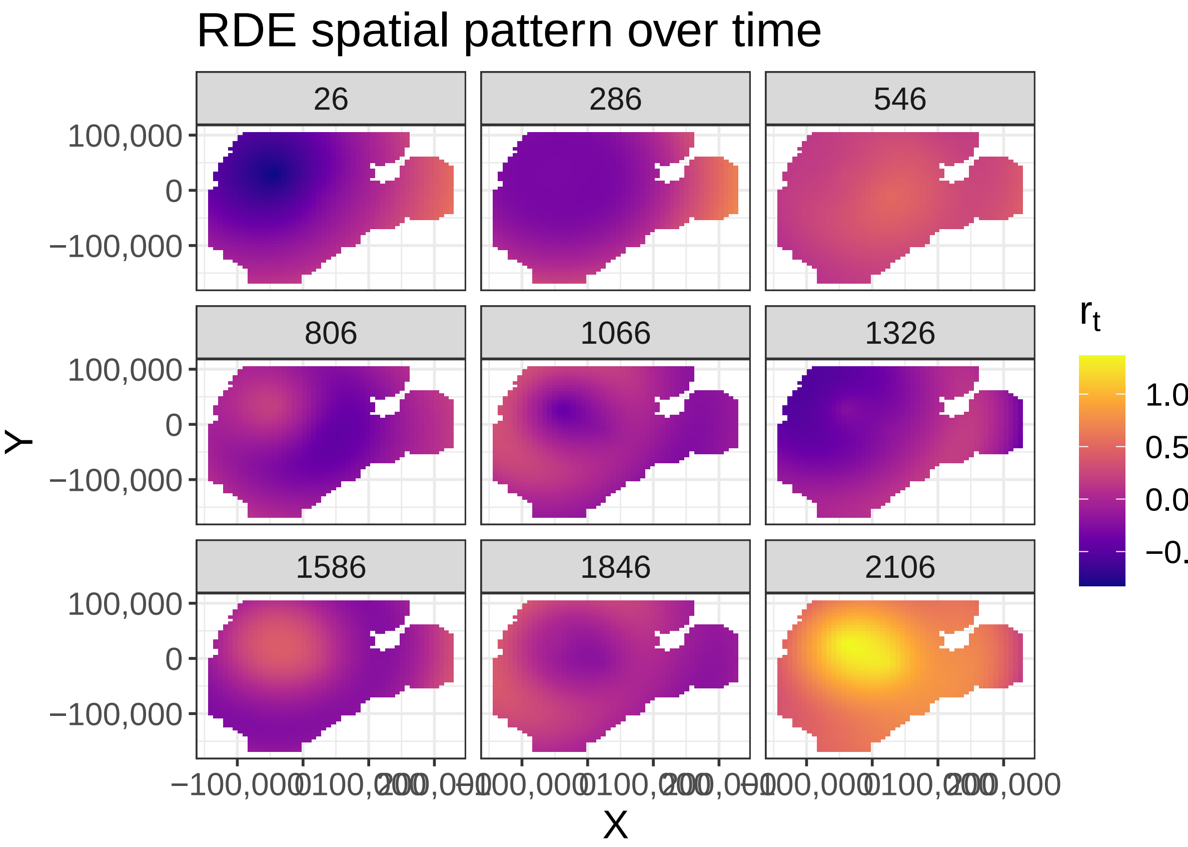

Spatio-temporal

RFC

```
space$D1 <- 1
space$rho1 <- 1
space$D2 <- 1
space$rho2 <- 1

for(i in 1:nrow(space)){
  if (space$north.south[i] == "north") {
    space$D1[i] <- sqrt((RFC_out$estimate[1] - space$cen.x[i])^2 + (RFC_out$estimate[2] - space$cen.y[i
    space$rho1[i] <- space$julian.mean.trans[i] + (1 / RFC_out$estimate[3]) * space$D1[i]
  } else {
    space$D2[i] <- sqrt((RFC_out$estimate[4] - space$cen.x[i])^2 + (RFC_out$estimate[5] - space$cen.y[i
    space$rho2[i] <- space$julian.mean.trans[i] + (1 / RFC_out$estimate[6]) * space$D2[i]
  }
}

RFC <- gam(r.growth ~ s(rho1, by = north, k = 12, bs = "tp") + s(rho2, by = south, k = 12, bs = "tp"),
           weights = sqrt_diff_survey,
           method = "ML",
           data = space,
           family = "gaussian")

plot(RFC, pages = 1, shade = TRUE, shade.col = "lightgrey", seWithMean = TRUE, residuals = TRUE, rug = 
```

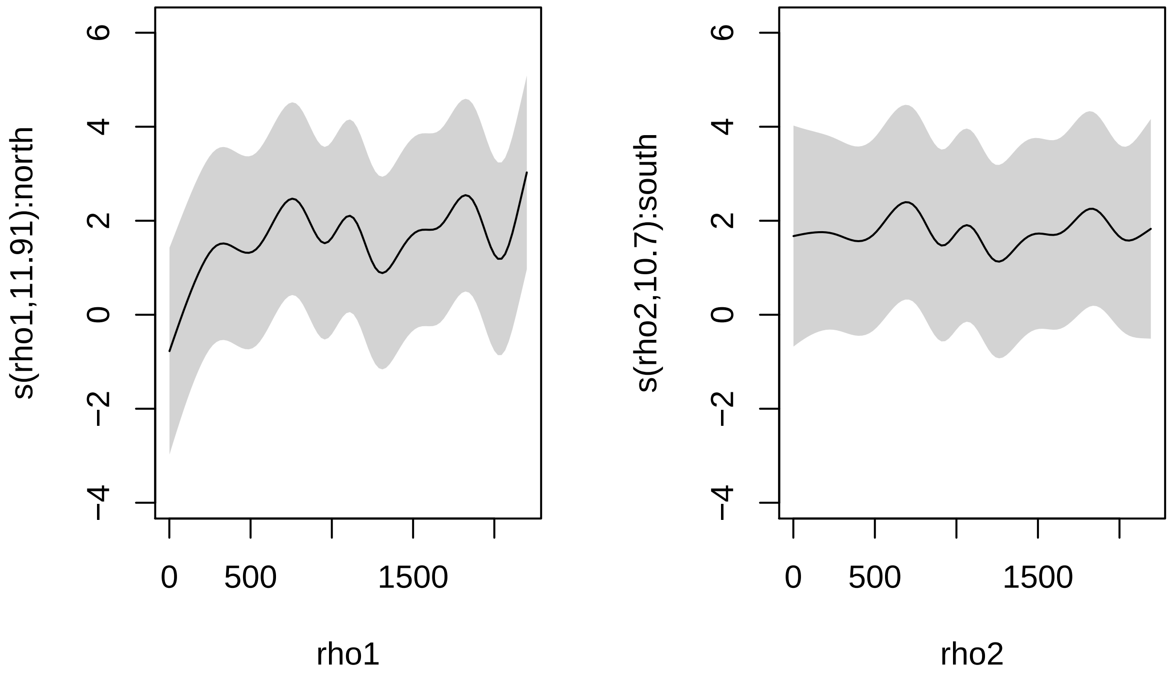

Temporal

```r
p_spatial_RFC <- expand.grid(cen.x = seq(min(space$cen.x),
                                         max(space$cen.x),
                                         length = reso[1]),
                             cen.y = seq(min(space$cen.y),
                                         max(space$cen.y),
                                         length = reso[2]),
                             julian.mean.trans = seq(min(space$julian.mean.trans),
                                                     max(space$julian.mean.trans),
                                                     length = reso[3])
)

p_spatial_RFC$north.south <- NA
p_spatial_RFC$north.south <- ifelse(p_spatial_RFC$cen.y > 0, "north", "south") # 41.8 latitude, 2590 tr
p_spatial_RFC$north.south <- as.factor(p_spatial_RFC$north.south)

p_spatial_RFC$D1 <- 1
p_spatial_RFC$rho1 <- 1
p_spatial_RFC$D2 <- 1
p_spatial_RFC$rho2 <- 1

for(i in 1:nrow(p_spatial_RFC)){
  if (p_spatial_RFC$north.south[i] == "north") {
    p_spatial_RFC$D1[i] <- -sqrt((RFC_out$estimate[1] - p_spatial_RFC$cen.x[i])^2 + (RFC_out$estimate[2
    p_spatial_RFC$rho1[i] <- p_spatial_RFC$julian.mean.trans[i] + (1 / RFC_out$estimate[3]) * p_spatial_
  } else {
    p_spatial_RFC$D2[i] <- -sqrt((RFC_out$estimate[4] - p_spatial_RFC$cen.x[i])^2 + (RFC_out$estimate[5
    p_spatial_RFC$rho2[i] <- p_spatial_RFC$julian.mean.trans[i] + (1 / RFC_out$estimate[6]) * p_spatial_
  }
}

p_spatial_RFC$north <- NA
p_spatial_RFC$south <- NA

p_spatial_RFC$north <- ifelse(p_spatial_RFC$north.south == "north", 1, 0)
p_spatial_RFC$south <- ifelse(p_spatial_RFC$north.south == "south", 1, 0)

RFC_fit <- data.frame(predict(RFC, newdata = p_spatial_RFC, se.fit = TRUE))

ind <- exclude.too.far(p_spatial_RFC$cen.x, p_spatial_RFC$cen.y,
                       space$cen.x, space$cen.y, dist = 0.1)

RFC_fit$fit[ind] <- NA

RFC_fit <- transform(RFC_fit,
                     upper = fit + (2 * se.fit),
                     lower = fit - (2 * se.fit))

pred_RFC <- cbind(p_spatial_RFC, RFC_fit)

ggplot() +
  geom_tile(data = pred_RFC, aes(x = cen.x, y = cen.y, fill = fit)) +
  scale_fill_viridis_c(option = "C", na.value = "transparent") +
```

```
  scale_x_continuous(label = scales::comma) +
  scale_y_continuous(label = scales::comma) +
  labs(x = "X",
       y = "Y",
       title = "RFC spatial pattern over time",
       fill= expression(r[t])) +
  theme_bw() +
  theme(text = element_text(size = 15)) +
  facet_wrap(~ round(julian.mean.trans))
```

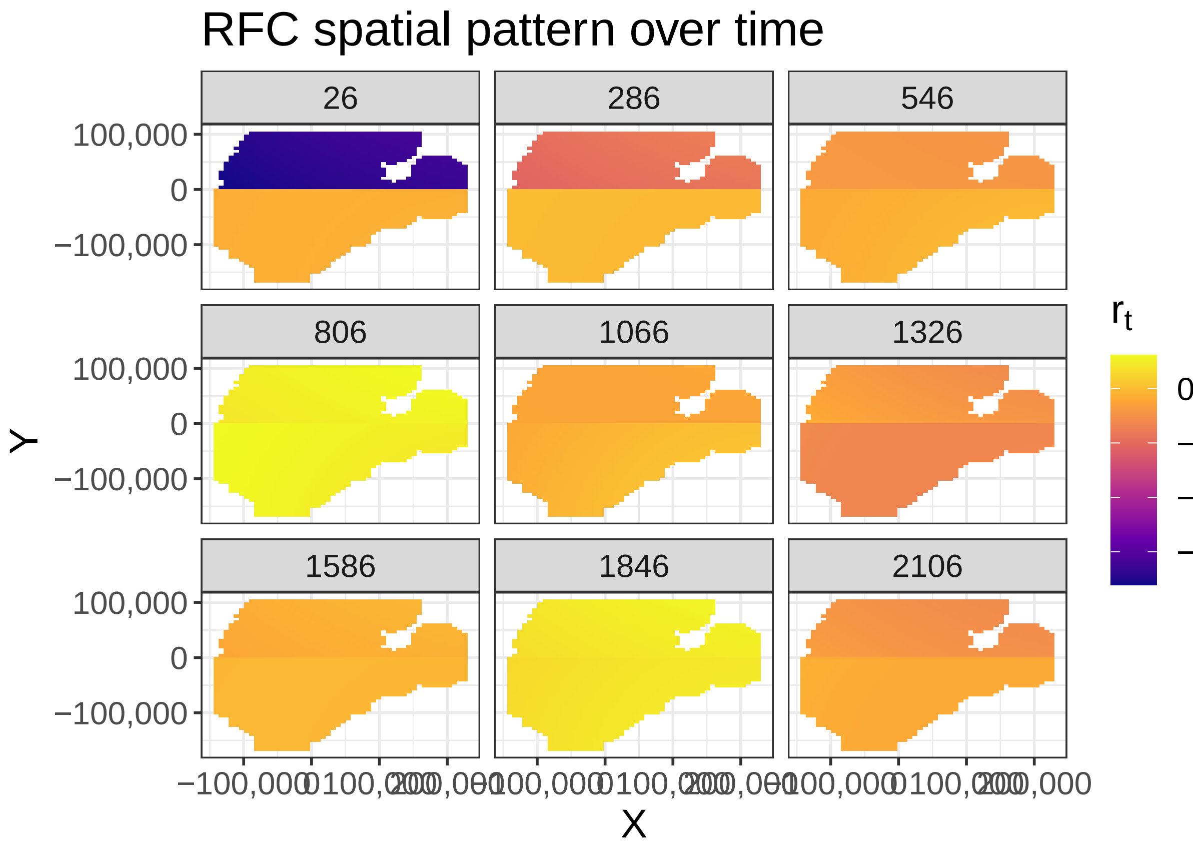

Spatio-temporal

RDC

```
space$D1 <- sqrt((RDC_out$estimate[1] - space$cen.x)^2 + (RDC_out$estimate[2] - space$cen.y)^2)
space$rho1 <- space$julian.mean.trans + (1 / RDC_out$estimate[3]) * space$D1

space$D2 <- sqrt((RDC_out$estimate[4] - space$cen.x)^2 + (RDC_out$estimate[5] - space$cen.y)^2)
space$rho2 <- space$julian.mean.trans + (1 / RDC_out$estimate[6]) * space$D2

RDC <- gam(r.growth ~ s(rho1, k = 12, bs = "tp") + s(rho2, k = 12, bs = "tp"),
           weights = sqrt_diff_survey,
           method = "ML",
           data = space,
```

```
            family = "gaussian")

plot(RDC, pages = 1, shade = TRUE, shade.col = "lightgrey", seWithMean = TRUE, residuals = TRUE, rug = F
```

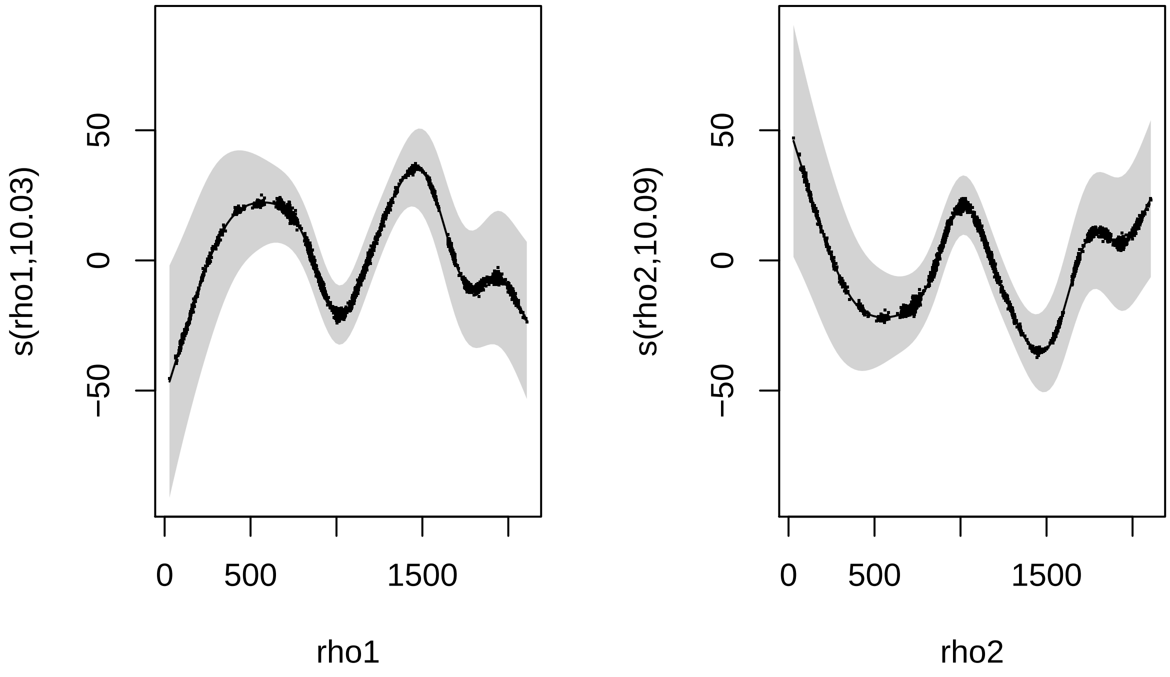

Temporal

```
p_spatial_RDC <- expand.grid(cen.x = seq(min(space$cen.x),
                                         max(space$cen.x),
                                         length = reso[1]),
                             cen.y = seq(min(space$cen.y),
                                         max(space$cen.y),
                                         length = reso[2]),
                             julian.mean.trans = seq(min(space$julian.mean.trans),
                                                     max(space$julian.mean.trans),
                                                     length = reso[3])
)

p_spatial_RDC$D1 <- sqrt((RDC_out$estimate[1] - p_spatial_RDC$cen.x)^2 + (RDC_out$estimate[2] - p_spatia
p_spatial_RDC$rho1 <- p_spatial_RDC$julian.mean.trans + (1 / RDC_out$estimate[3]) * p_spatial_RDC$D1

p_spatial_RDC$D2 <- sqrt((RDC_out$estimate[4] - p_spatial_RDC$cen.x)^2 + (RDC_out$estimate[5] - p_spatia
p_spatial_RDC$rho2 <- p_spatial_RDC$julian.mean.trans + (1 / RDC_out$estimate[6]) * p_spatial_RDC$D2

RDC_fit <- data.frame(predict(RDC, newdata = p_spatial_RDC, se.fit = TRUE))
```

```r
ind <- exclude.too.far(p_spatial_RDC$cen.x, p_spatial_RDC$cen.y,
                       space$cen.x, space$cen.y, dist = 0.1)

RDC_fit$fit[ind] <- NA

RDC_fit <- transform(RDC_fit,
                     upper = fit + (2 * se.fit),
                     lower = fit - (2 * se.fit))

pred_RDC <- cbind(p_spatial_RDC, RDC_fit)

ggplot() +
  geom_tile(data = pred_RDC, aes(x = cen.x, y = cen.y, fill = fit)) +
  scale_fill_viridis_c(option = "C", na.value = "transparent") +
  scale_x_continuous(label = scales::comma) +
  scale_y_continuous(label = scales::comma) +
  labs(x = "X",
       y = "Y",
       title = "RDC spatial pattern over time",
       fill= expression(r[t])) +
  theme_bw() +
  theme(text = element_text(size = 15)) +
  facet_wrap(~ round(julian.mean.trans))
```

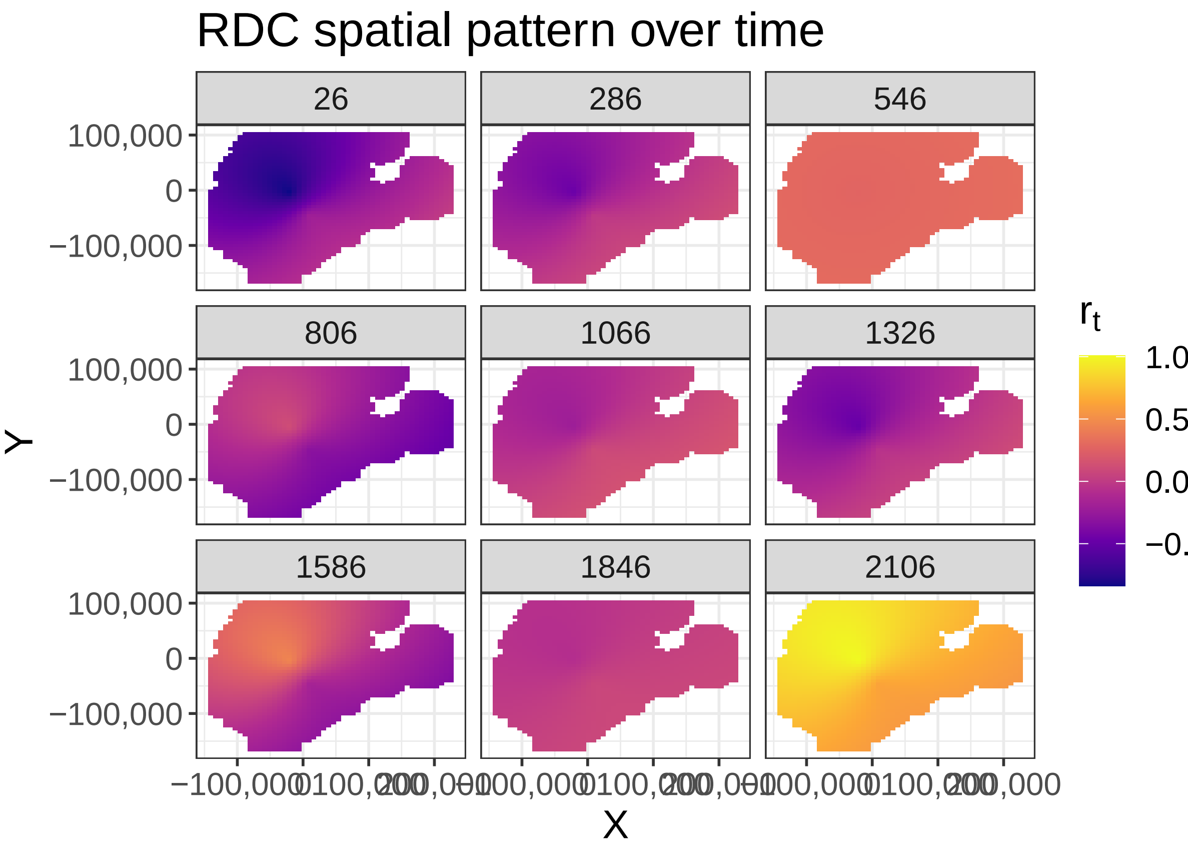

Spatio-temporal

PF

```
space$D1 <- 1
space$rho1 <- 1
space$D2 <- 1
space$rho2 <- 1

for(i in 1:nrow(space)){
  if (space$north.south[i] == "north") {
    space$D1[i] <-  sin(PF_out$estimate[1]) * space$cen.x[i] + cos(PF_out$estimate[1]) * space$cen.y[i]
    space$rho1[i] <- space$julian.mean.trans[i] + (1 / PF_out$estimate[2]) * space$D1[i]
  } else {
    space$D2[i] <- sin(PF_out$estimate[3]) * space$cen.x[i] + cos(PF_out$estimate[3]) * space$cen.y[i]
    space$rho2[i] <- space$julian.mean.trans[i] + (1 / PF_out$estimate[4]) * space$D2[i]
  }
}

PF <- gam(r.growth ~ s(rho1, by = north, k = 12, bs = "tp") + s(rho2, by = south, k = 12, bs = "tp"),
          weights = sqrt_diff_survey,
          method = "ML",
          data = space,
          family = "gaussian")

plot(PF, pages = 1, shade = TRUE, shade.col = "lightgrey", seWithMean = TRUE, residuals = TRUE, rug = FA
```

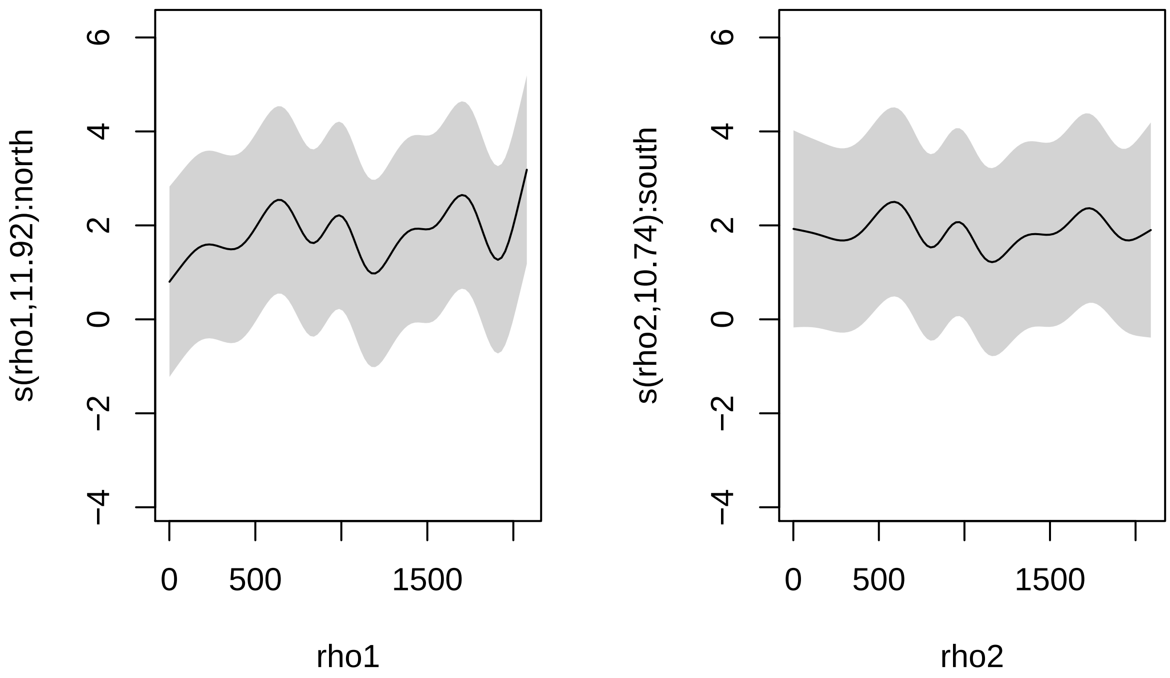

Temporal

```
p_spatial_PF <- expand.grid(cen.x = seq(min(space$cen.x),
                                        max(space$cen.x),
                                        length = reso[1]),
                            cen.y = seq(min(space$cen.y),
                                        max(space$cen.y),
                                        length = reso[2]),
                            julian.mean.trans = seq(min(space$julian.mean.trans),
                                                    max(space$julian.mean.trans),
                                                    length = reso[3])
)

p_spatial_PF$north.south <- NA
p_spatial_PF$north.south <- ifelse(p_spatial_PF$cen.y > 0, "north", "south") # 41.8 latitude, 2590 true
p_spatial_PF$north.south <- as.factor(p_spatial_PF$north.south)

p_spatial_PF$D1 <- 1
p_spatial_PF$rho1 <- 1
p_spatial_PF$D2 <- 1
p_spatial_PF$rho2 <- 1

for(i in 1:nrow(p_spatial_PF)){
  if (p_spatial_PF$north.south[i] == "north") {
    p_spatial_PF$D1[i] <- sin(PF_out$estimate[1]) * p_spatial_PF$cen.x[i] + cos(PF_out$estimate[1]) * p
    p_spatial_PF$rho1[i] <- p_spatial_PF$julian.mean.trans[i] + (1 / PF_out$estimate[2]) * p_spatial_PF
  } else {
    p_spatial_PF$D2[i] <- sin(PF_out$estimate[3]) * p_spatial_PF$cen.x[i] + cos(PF_out$estimate[3]) * p
    p_spatial_PF$rho2[i] <- p_spatial_PF$julian.mean.trans[i] + (1 / PF_out$estimate[4]) * p_spatial_PF
  }
}

p_spatial_PF$north <- NA
p_spatial_PF$south <- NA

p_spatial_PF$north <- ifelse(p_spatial_PF$north.south == "north", 1, 0)
p_spatial_PF$south <- ifelse(p_spatial_PF$north.south == "south", 1, 0)

PF_fit <- data.frame(predict(PF, newdata = p_spatial_PF, se.fit = TRUE))

ind <- exclude.too.far(p_spatial_PF$cen.x, p_spatial_PF$cen.y,
                       space$cen.x, space$cen.y, dist = 0.1)

PF_fit$fit[ind] <- NA

PF_fit <- transform(PF_fit,
                    upper = fit + (2 * se.fit),
                    lower = fit - (2 * se.fit))

pred_PF <- cbind(p_spatial_PF, PF_fit)

ggplot() +
  geom_tile(data = pred_PF, aes(x = cen.x, y = cen.y, fill = fit)) +
  scale_fill_viridis_c(option = "C", na.value = "transparent") +
```

```r
  scale_x_continuous(label = scales::comma) +
  scale_y_continuous(label = scales::comma) +
  labs(x = "X",
       y = "Y",
       title = "PF spatial pattern over time",
       fill= expression(r[t])) +
  theme_bw() +
  theme(text = element_text(size = 15)) +
  facet_wrap(~ round(julian.mean.trans))
```

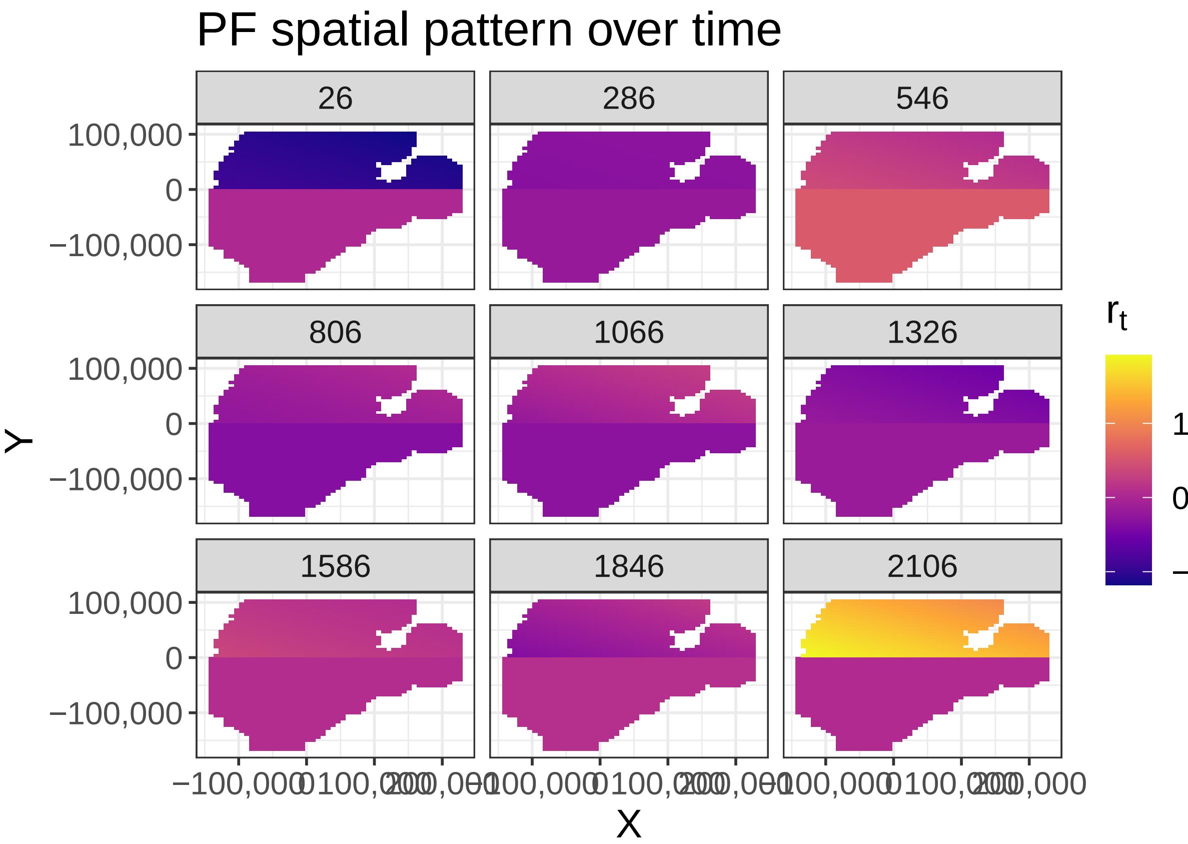

Spatio-temporal

PD

```r
space$D1 <-  sin(PD_out$estimate[1]) * space$cen.x + cos(PD_out$estimate[1]) * space$cen.y
space$rho1 <- space$julian.mean.trans + (1 / PD_out$estimate[2]) * space$D1
space$D2 <- sin(PD_out$estimate[3]) * space$cen.x + cos(PD_out$estimate[3]) * space$cen.y
space$rho2 <- space$julian.mean.trans + (1 / PD_out$estimate[4]) * space$D2

PD <- gam(r.growth ~ s(rho1, k = 12, bs = "tp") + s(rho2, k = 12, bs = "tp"),
          weights = sqrt_diff_survey,
          method = "ML",
          data = space,
          family = "gaussian")
```

```r
plot(PD, pages = 1, shade = TRUE, shade.col = "lightgrey", seWithMean = TRUE, residuals = TRUE, rug = FA
```

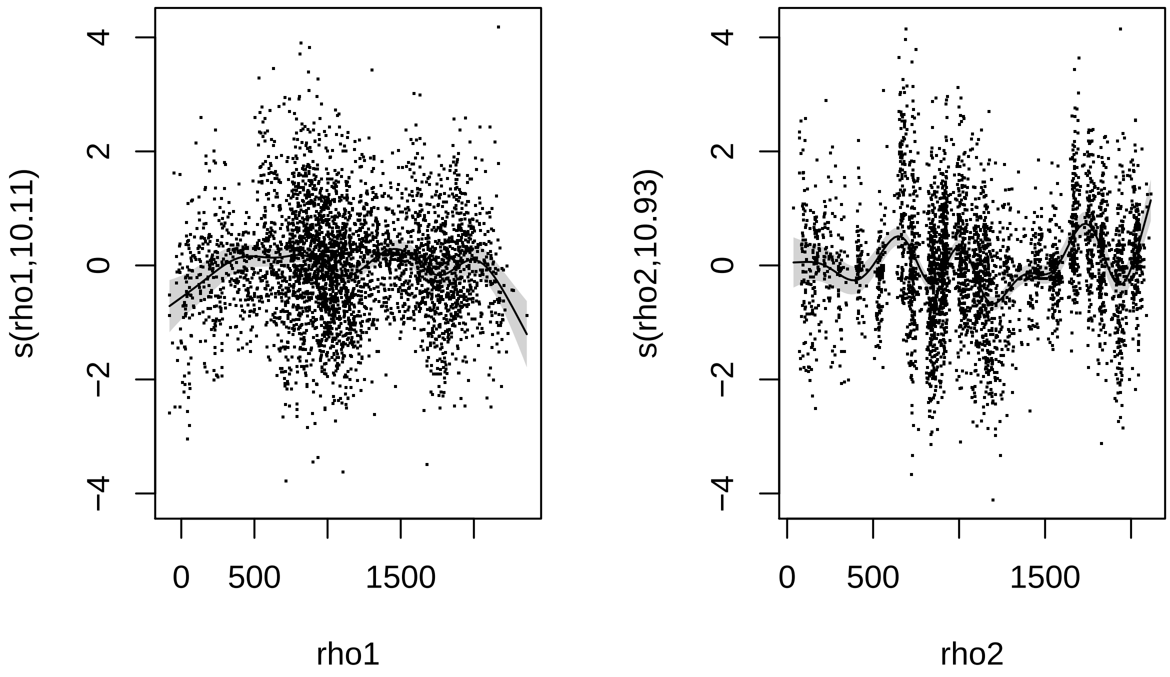

Temporal

```r
p_spatial_PD <- expand.grid(cen.x = seq(min(space$cen.x),
                                        max(space$cen.x),
                                        length = reso[1]),
                            cen.y = seq(min(space$cen.y),
                                        max(space$cen.y),
                                        length = reso[2]),
                            julian.mean.trans = seq(min(space$julian.mean.trans),
                                                    max(space$julian.mean.trans),
                                                    length = reso[3])
)

p_spatial_PD$D1 <- 1
p_spatial_PD$rho1 <- 1
p_spatial_PD$D2 <- 1
p_spatial_PD$rho2 <- 1

p_spatial_PD$D1 <-  sin(PD_out$estimate[1]) * p_spatial_PD$cen.x + cos(PD_out$estimate[1]) * p_spatial_I
p_spatial_PD$rho1 <- p_spatial_PD$julian.mean.trans + (1 / PD_out$estimate[2]) * p_spatial_PD$D1
p_spatial_PD$D2 <- sin(PD_out$estimate[3]) * p_spatial_PD$cen.x + cos(PD_out$estimate[3]) * p_spatial_PI
p_spatial_PD$rho2 <- p_spatial_PD$julian.mean.trans + (1 / PD_out$estimate[4]) * p_spatial_PD$D2
```

```r
PD_fit <- data.frame(predict(PD, newdata = p_spatial_PD, se.fit = TRUE))

ind <- exclude.too.far(p_spatial_PD$cen.x, p_spatial_PD$cen.y,
                       space$cen.x, space$cen.y, dist = 0.1)

PD_fit$fit[ind] <- NA

PD_fit <- transform(PD_fit,
                    upper = fit + (2 * se.fit),
                    lower = fit - (2 * se.fit))

pred_PD <- cbind(p_spatial_PD, PD_fit)

ggplot() +
  geom_tile(data = pred_PD, aes(x = cen.x, y = cen.y, fill = fit)) +
  scale_fill_viridis_c(option = "C", na.value = "transparent") +
  scale_x_continuous(label = scales::comma) +
  scale_y_continuous(label = scales::comma) +
  labs(x = "X",
       y = "Y",
       title = "PD spatial pattern over time",
       fill= expression(r[t])) +
  theme_bw() +
  theme(text = element_text(size = 15)) +
  facet_wrap(~ round(julian.mean.trans))
```

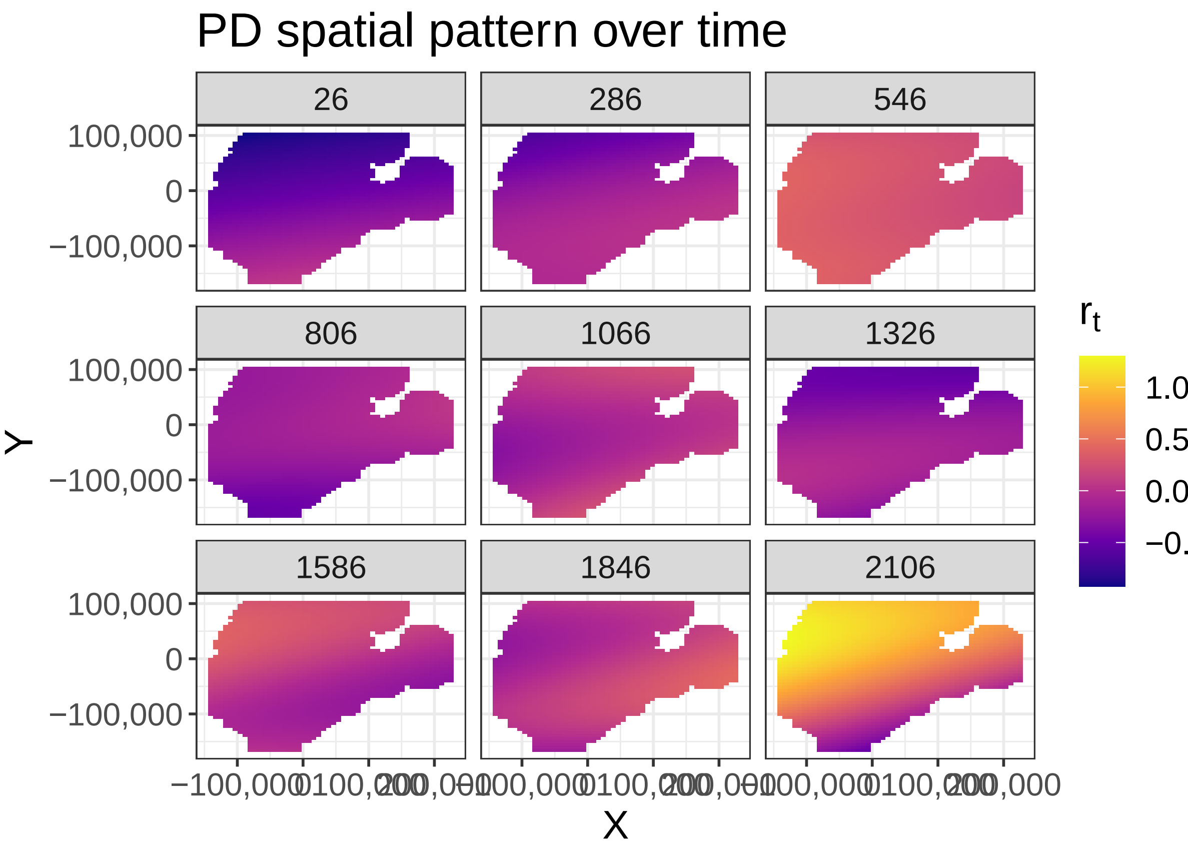

Spatio-temporal

AIC

Table showing corrected AIC values for each of the models tested here.

```
model_aic <- data.frame(
  Model = c("N1", "N2", "N3", "RE", "RC", "P", "RFE", "RDE", "RFC", "RDC", "PF", "PD"),
  Hypothesis = c(
    "Mean growth",
    "Phase-locked",
    "Spatial pattern",

    "Single radial expanding wave",
    "Single radial contracting wave",
    "Single planar wave",

    "Dual isolated expanding radial waves",
    "Activator-inhibitor expanding radial wave",

    "Dual isolated contracting radial waves",
    "Activator-inhibitor contracting radial wave",

    "Dual isolated planar waves",
    "Activator-inhibitor radial wave"
  ),
  AIC = c(
    AIC(N1),
    AIC(N2),
    AIC(N3),
    AIC(RE) + 2*length(par_list_RE),
    AIC(RC) + 2*length(par_list_RC),
    AIC(P_TW) + 2*length(par_list_P_TW),
    AIC(RFE) + 2*length(par_list_RFE),
    AIC(RDE) + 2*length(par_list_RDE),
    AIC(RFC) + 2*length(par_list_RFC),
    AIC(RDC) + 2*length(par_list_RDC),
    AIC(PF) + 2*length(par_list_PF),
    AIC(PD) + 2*length(par_list_PD)
  )
)

model_aic$deltaAIC <- model_aic$AIC - min(model_aic$AIC)

model_aic
```

Model	Hypothesis	AIC	deltaAIC
N1	Mean growth	9358.084	1089.97331
N2	Phase-locked	8475.602	207.49183
N3	Spatial pattern	9354.944	1086.83409
RE	Single radial expanding wave	8451.388	183.27795
RC	Single radial contracting wave	8451.473	183.36287
P	Single planar wave	8449.448	181.33756
RFE	Dual isolated expanding radial waves	8328.895	60.78436
RDE	Activator-inhibitor expanding radial wave	8268.110	0.00000

Model	Hypothesis	AIC	deltaAIC
RFC	Dual isolated contracting radial waves	8377.283	109.17223
RDC	Activator-inhibitor contracting radial wave	8321.274	53.16329
PF	Dual isolated planar waves	8350.728	82.61781
PD	Activator-inhibitor planar wave	8358.718	90.60730

Total run time

```
Sys.time() - analysis_start
```

```
## Time difference of 1.478722 days
```

Saving environment

```
save.image("C:\\Users\\r01dr16\\Desktop\\Travelling wave\\travelling_wave_model_runs.RData")
```

System info

```
Sys.info()
```

```
##        sysname        release        version       nodename        machine
##      "Windows"       "10 x64"  "build 18363"    "MD-056630"       "x86-64"
##          login           user effective_user
##      "r01dr16"      "r01dr16"      "r01dr16"
```
