# Supplementary material for: From pattern to process? Dual travelling waves, with contrasting propagation speeds, best describe a self‐organised spatio‐temporal pattern in population growth of a cyclic rodent
Source: Ecol Lett. 2022 Jul 31;25(9):1986–98. doi: 10.1111/ele.14074 (PMC9543711; doi:10.1111/ele.14074)
Supplement: Supplementary file 3 — Supinfo S2 [file ELE-25-1986-s005.pdf]

Travelling wave simulation

Deon Roos

Last run of 2022-03-28

Contents

Synopsis **2**

Packages used **3**

Simulation of single expanding radial travelling wave. **4**

True wave parameters 4
Creating realm 4
Simulating wave 4
Raw simulated trend 5
Figure 1 6
Figure 2 7
Parameter esimation 7
Table 1 10
Optimsation process 10
Figure 3 11
Figure 4 12
Predicted growth rate 12
Figure 5 14
Figure 6 16
Outcome of method with simulated data 16

Visual summary of analytical process **16**

Figure 7 17
Figure 8 20

Simulation with adaptive monitoring **20**
Select samples to analyse . . . 20
Raw simulated trend . . . 21
Figure 9 . . . 21
Figure 10 . . . 23
Parameter estimation . . . 23
Table 2 . . . 26
Optimsation process . . . 26
Figure 11 . . . 27
Figure 12 . . . 28
Predicted growth rate . . . 28
Figure 13 . . . 30
Figure 14 . . . 32
Outcome of method with simulated data with adapative monitoring . . . 33

Session information **33**

Synopsis

To ensure that the method used to fit the models is reliable, within this document we create two simulated traveling wave datasets and attempt to recover the known true parameter values using the statistical methods described in the main text.

In the first section of this document, a simple form of the traveling wave is used here, a single expanding radial traveling wave (Model RE, Table 1 in main text). This wave is simulated as:

$r_{i,t} = \mathcal{A}\sin(f\rho_{i,t}) + Normal(0, 0.5)$

$\rho_{i,t} = T_{i,t} + (\frac{1}{S}) \times D_i$

$D = -\sqrt{(\psi - X_i)^2 + (\gamma - Y_i)^2}$

where $r_{i,t}$ is the growth rate of centroid i at time t, and is generated according to a sin wave, with an amplitude of $\mathcal{A}$ and frequency of f. The values for amplitude and frequency were chosen to simulate growth that visually resembled the time series trend in the real data. Noise is included through the inclusion of values generated from a Normal distribution, with a mean of zero and an arbitrary standard deviation of 0.5. Space-modified time (ρ) is generated using the day of observation (T) added to the inverse of the speed of the wave ($\frac{1}{S}$), multiplied by distance from the epicentre (D). Distance is generated according to the negative (for an expanding radial wave) Pythagorean distance from each centroid coordinate pairing (X and Y) to the epicentre location (ψ and γ, set to 50,000 and -25,000 respectively), where,

$\mathcal{A} = 2$

$f = 0.005$

$S = 300$

$\psi = 50000$

$\gamma = -25000$

Time (T) is generated by creating a sequence of observation dates from the same time frame as in the real data, while centroid locations are generated using Uniform distributions with minimum and maximum values from the centroid locations in the real data.

In the second section of this document, the same traveling wave is simulated (using the same process and values as above), but the sampling process includes an "adaptive monitoring scheme" to better reflect the real data, whereby monitoring efforts focus on a problematic area (the equivalent to Tierra de Campos) and the probability that a location is surveyed increases with its growth rates. This simulation seeks to address concerns that the estimation of an epicenter's location is determined, in part, by the adaptive monitoring scheme. We decide to recreate the adaptive monitoring in this simulation as an "extreme" version of the real world (survey hyper-focused on one area), such that we are able to demonstrate adaptive monitoring is unlikely to have an influence on the estimation of the parameters.

To accommodate this, each centroid is assigned a probability to be surveyed, based on the centroids growth rate at that time ($r_{i,t}$), as well as whether or not the centroid falls within the core area ($Core_i$). Parameter values are chosen such that effort would be disproportionately focused in the core area, with non-core centroids most likely to be surveyed if they experience especially high growth (the maximum probability for a non-core centroid to be surveyed was $< 15\%$). In this way, this simulation is an exaggerated reflection of the real data.

Sampling was simulated according to,

$\xi_{i,t} \sim Binomial(p)$

$logit(p_{i,t}) = \beta_1 + \beta_2 \times r_{i,t} + \beta_3 \times Core_i$

with the values,

$\beta_1 = -5$

$\beta_2 = 0.8$

$\beta_3 = 7$

Designation to the core area was determined by,

$$Core_i = \begin{cases} 1, & \text{if } (X_i - Core_X)^2 + (Y_i - Core_Y)^2 < R^2 \\ 0, & \text{otherwise} \end{cases}$$

where X_i and Y_i are the coordinates of centroid i and $Core_X$ and $Core_Y$ (set to 100000 and 50000 respectively) is the center point of the core area, with a radius R of 25000.

The seed is set to `1234` in each relevant code chunk.

Packages used

Core packages used are `mgcv` (Wood, 2011), `emdbook` (Bolker, 2020), and `dplyr` (Wickham et al., 2021). `mgcv` is used to fit the generalised additive model, `emdbook` is used to fit the entire travelling wave model (SANN optimiser), and `dplyr` is used to sample a specified number of observations (and could be replaced with base `R`). Additional packages used are `ggplot2` (Wickham, 2016), `scales` (Wickham & Deidel, 2020), and `patchwork` (Pedersen, 2020). The additional packages are used exclusively for visualisations and are not required to run the analysis.

```
library(mgcv)       # For the gam component of the model
library(emdbook)    # Bolker's package for stochastic annealing optimiser method
library(ggplot2)    # For visualisation
library(scales)     # For comma scales
```

```
library(patchwork) # For plotting side-by-side
library(dplyr)     # For sample_n()
```

Simulation of single expanding radial travelling wave.

As previously outlined, the first simulation assumes the presence of a single expanding travelling wave. Importantly, this simulation assumes no adaptive monitoring (see second simulation for an adaptive monitoring dataset).

True wave parameters

The simulation uses the values:

- X coordinate of epicentre placed at 50,000 UTM, where UTM is mean centered (ψ).
- Y coordinate of epicentre placed at -25,000 UTM, where UTM is mean centered (γ).
- A single constant speed of 300 m per time interval (S).

Given these values are known, we are able to evaluate how well the analytical approach is able to recover these known values (see Outcome of method with simulated data section below for a comparison).

Creating realm

The spatial and temporal realms are generated using the ranges from the real dataset.

```
set.seed(1234)
x <- runif(n = 50, min = min(space$cen.x), max = max(space$cen.x))
y <- runif(n = 50, min = min(space$cen.y), max = max(space$cen.y))
time <- seq(length.out = 50, from = min(space$julian.mean.trans),
            to = max(space$julian.mean.trans))
map <- expand.grid(y, x, time)
df <- data.frame(
  x = map$Var2,
  y = map$Var1,
  t = map$Var3
)
```

Simulating wave

The sample size is reduced to 3751 observations (the same number as in the real dataset).

```
set.seed(1234)
# True wave parameters
# Given I specify the parameter values here, if the statistical method is
  # reliable, then these should be retrieved.
x_wave <- 50000   # the x coordinate of the wave epicenter
y_wave <- -25000  # the y coordinate of the wave epicenter
speed <- 300      # the speed of the wave
```

```r
# Sin wave features
# Values chosen to visually such that the simulated wave resembles the real data
freq <- 0.005 # frequency of wave
amp <- 2 # amplitude of wave

# noise
# Standard deviation used is arbitrary
n <- nrow(df)
mu <- 0
std <- 0.5

# Simulated variables

# Distance from epicenter
df$D_true <- -sqrt((x_wave - df$x)^2 + (y_wave - df$y)^2)

# Convert to space-modified time
df$rho_true <- df$t + (1/speed) * df$D_true

# Resulting growth rate generated using a sin wave with some noise
df$r.growth <- amp * sin(freq * df$rho_true) + rnorm(n, mu, std)

# Store in df_true to allow comparison later
df_true <- df

# df becomes the test dataset. 3751 samples retrieved to reflect genuine dataset.
df <- sample_n(df, 3751)
```

Raw simulated trend

```r
ggplot(df) +
  geom_point(aes(x = t, y = r.growth, fill = r.growth),
             alpha = 0.3, pch = 21, colour = "black") +
  geom_hline(yintercept = 0, linetype = 2) +
  scale_fill_viridis_c(option = "C") +
  labs(y = expression(r[t,i]),
       x = "Mean day of data collection in centroid",
       fill = expression(r[t][i]),
       size = "Number of transects\nper centroid") +
  theme_classic() +
  NULL
```

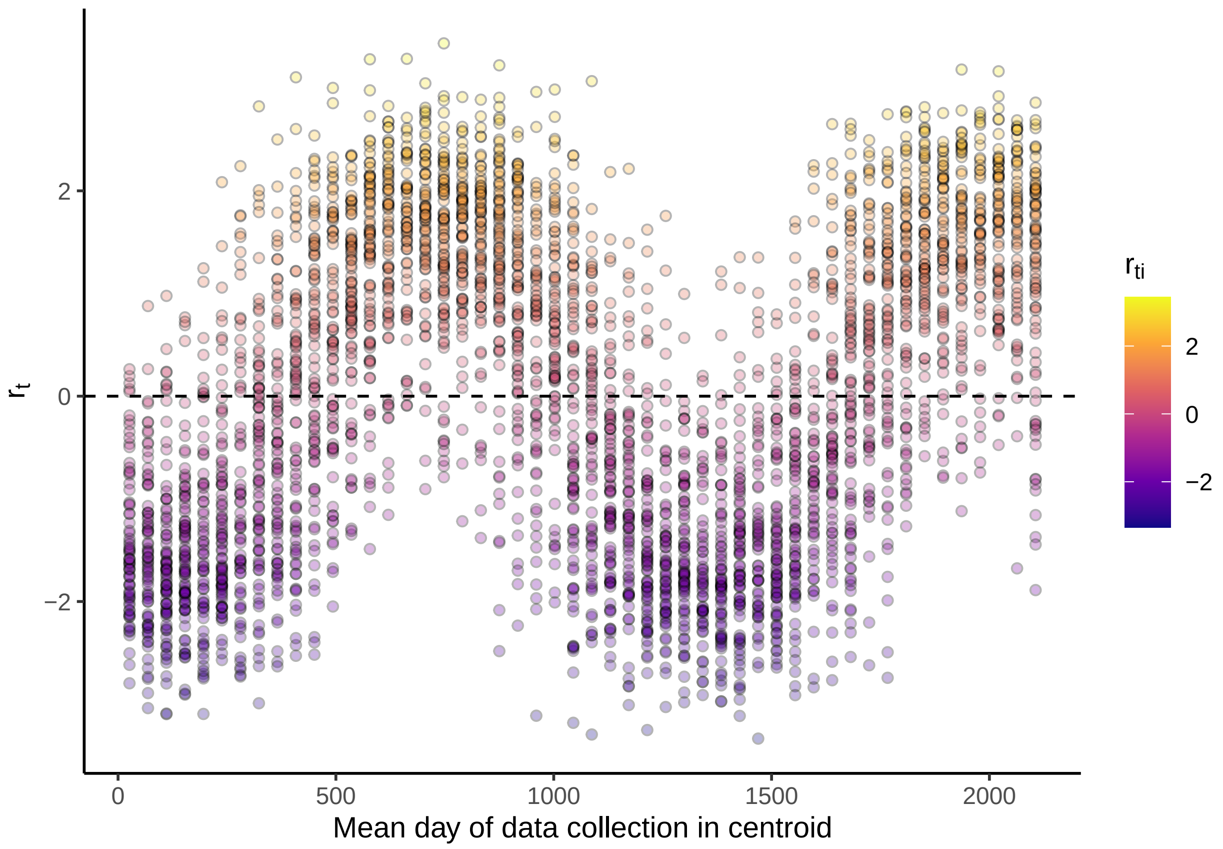

Figure 1

Simulated growth rate over time, as generated using the parameter values specified above. Frequency and amplitude of the sin wave were selected such that the simulated data would superficially resemble the real data.

```
# Yearly quarter calculated for plotting
  # Yearly quarter treated as 90 day period
df$qrt <- as.integer(df$t/90) + 1

ggplot(df) +
  geom_point(aes(x = x, y = y, colour = r.growth)) +
  scale_colour_viridis_c(option = "C") +
  scale_x_continuous(label= comma) +
  scale_y_continuous(label= comma) +
  facet_wrap(~qrt) +
  theme_classic() +
  theme(text = element_text(size = 8),
        axis.text.x = element_text(angle = 60, vjust = 1, hjust=1)) +
  labs(x = "X UTM",
       y = "Y UTM",
       colour = expression(r[t]))
```

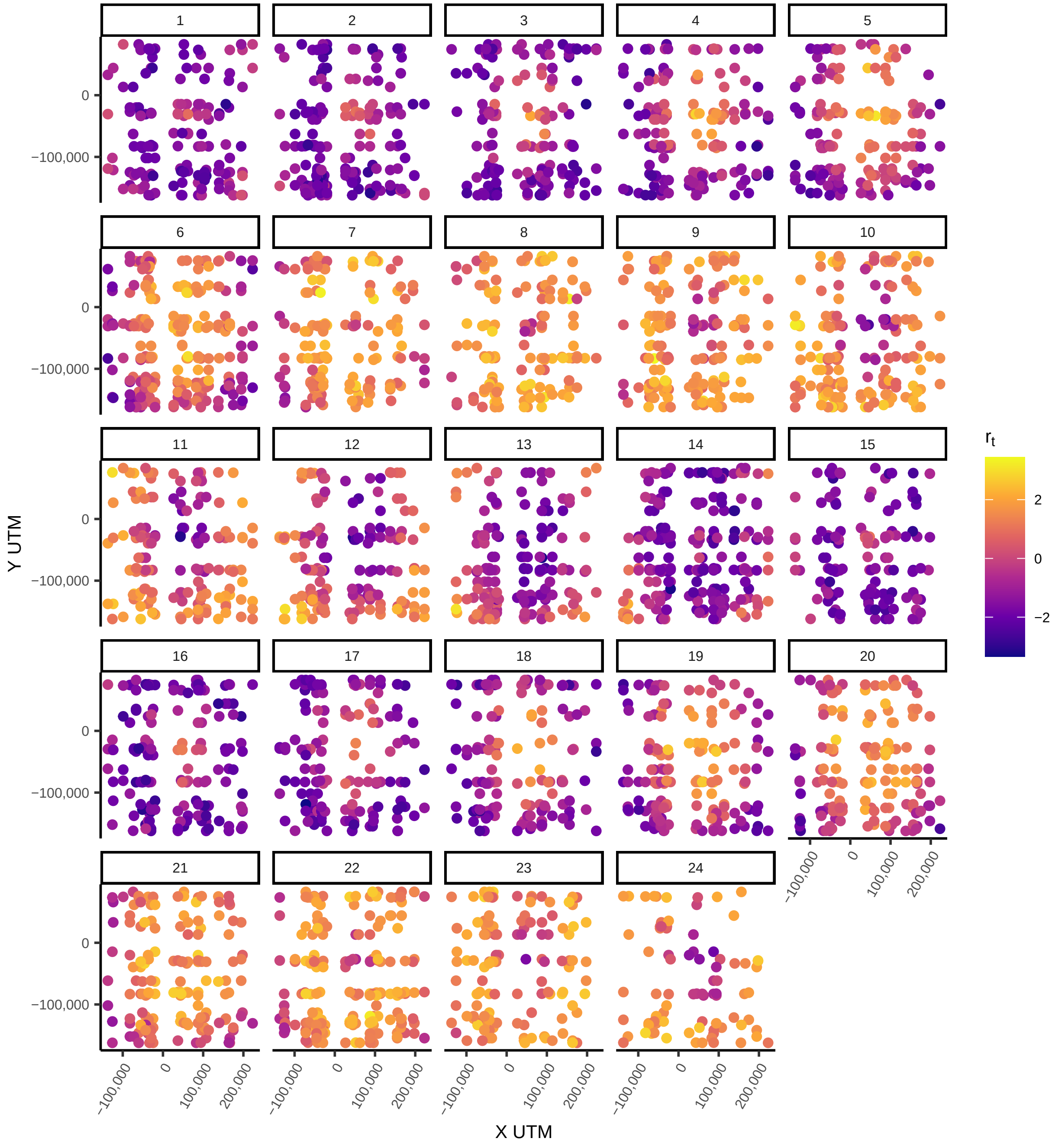

Figure 2

Growth rate across space in yearly quarters.

Parameter esimation

The data is fed into the appropriate model (model RE). If the statistical method is reliable, then this should retrieve parameter estimates close to the known, true values. Details of the analysis are described in the main text (see Table 1 in main text).

The SANN optimiser is run for 10,000 iterations (as opposed to 15,000 used with real analysis to reduce run time).

```
niter <- 10000
```

The initial parameter values used in the optimiser are arbitrarily specified to: 10000 for the X coordinate; -5000 for the Y coordinate; and a speed of 1000 m per day.

```
set.seed(1234)
# Function to run the model
RE_fun_sim <- function(par, data = df) {
  df$D_est <- -sqrt((par[1] - df$x)^2 + (par[2] - df$y)^2)
  df$rho_est <- df$t + (1 / par[3]) * df$D_est

  -logLik.gam(gam(r.growth ~ s(rho_est, k = 12, bs = "tp"),
                 method = "ML",
                 data = df,
                 family = "gaussian")
  )[1]
}

# Specifying the initial values
par_list_RE_sim <- c(10000, -5000, 1000)

# Recording start time of model run
start_time <- Sys.time()

# Running the model as in formal analysis
RE_sim_out <- metropSB(fn = RE_fun_sim,
                   start = par_list_RE_sim,
                   nmax = niter,
                   retvals = TRUE,
                   retfreq = 1,
                   verbose = FALSE,
                   rptfreq = -1)

# Report total run time
Sys.time() - start_time
```

```
## Time difference of 1.003862 hours
```

The parameter values are then extracted along with the 95% confidence intervals, using the methods described in Bolker (2020).

```
set.seed(1234)
# Extract parameter space from SANN
RE_sim_out_df <- data.frame(RE_sim_out$retvals[,c(1:3, 10)])

# Convert negative log likelihood to -2lnL
RE_sim_out_df$val <- 2*RE_sim_out_df$val

# Create empty dataframe
RE_sim_profile <- data.frame(
```

```r
  p1 = NA,
  p2 = NA,
  p3 = NA,
  val = NA
)

# For each unique speed considered by SANN
for(i in 1:length(unique(RE_sim_out_df$p3))) {

  # Store all unique values of speed
  match <- unique(RE_sim_out_df$p3)

  # Store all parameter combinations for a given speed considered
  temp <- subset(RE_sim_out_df, p3 == match[i])

  # Extract all parameter combinations where L is maximised
  temp1 <- temp[temp$val == max(temp$val),]

  # combine with previously created RE_sim_profile
  RE_sim_profile <- rbind(RE_sim_profile, temp1)
}

# Remove first NA entry when creating dataframe
RE_sim_profile <- RE_sim_profile[-1,]

# Find where -2lnL is best
lnLmax <- RE_sim_profile$val[RE_sim_profile$val == min(RE_sim_profile$val)]

# Calculate difference for each lnL from best
RE_sim_profile$lnL_diff <- RE_sim_profile$val - lnLmax

# For all parameter combinations, extract those that fall within chisq 95% CI
p3ci_profile_df <- RE_sim_profile[RE_sim_profile$lnL_diff < qchisq(p = 0.95, df = 1),]
p3min <- min(p3ci_profile_df$p3)
p3max <- max(p3ci_profile_df$p3)


# Repeat above, but for centroid coordinates
RE_sim_out_df$centroid <- paste(RE_sim_out_df$p1, RE_sim_out_df$p2, sep = " ")
RE_sim_profile <- data.frame(
  p1= NA,
  p2 = NA,
  p3 = NA,
  val = NA,
  centroid = NA
)
for(i in 1:length(unique(RE_sim_out_df$centroid))) {
  match <- unique(RE_sim_out_df$centroid)
  temp <- subset(RE_sim_out_df, centroid == match[i])
  temp1 <- temp[temp$val == max(temp$val),]
  RE_sim_profile <- rbind(RE_sim_profile, temp1)
}
RE_sim_profile <- RE_sim_profile[-1,]
```

```
lnLmax <- RE_sim_profile$val[RE_sim_profile$val == min(RE_sim_profile$val)]
RE_sim_profile$lnL_diff <- RE_sim_profile$val - lnLmax

# Note that CI is based on 2 degrees of freedom from chisq
ci_profile_df <- RE_sim_profile[RE_sim_profile$lnL_diff < qchisq(p = 0.95, df = 2),]
p1min <- min(ci_profile_df$p1)
p1max <- max(ci_profile_df$p1)
p2min <- min(ci_profile_df$p2)
p2max <- max(ci_profile_df$p2)
RE_sim_est <- data.frame(
  Parameter = c("Epicentre X", "Epicentre Y", "Speed"),
  Units = c("mean centred UTM", "mean centred UTM", "m per day"),
  True = comma(c(x_wave, y_wave, speed)),
  Estimate = comma(RE_sim_out$estimate),
  Lower95CI = comma(c(p1min, p2min, p3min)),
  Upper95CI = comma(c(p1max, p2max, p3max)),
  Initial = comma(par_list_RE_sim)
)
RE_sim_est
```

Parameter	Units	True	Estimate	Lower95CI	Upper95CI	Initial
Epicentre X	mean centred UTM	50,000	49,749	49,229	51,133	10,000
Epicentre Y	mean centred UTM	-25,000	-24,048	-24,148	-23,016	-5,000
Speed	m per day	300	305	300	310	1,000

Table 1

The above table shows the true parameter values, the estimated parameter values and their 95% CI, as well as the value which the SANN optimiser was initiated at.

Optimsation process

```
RE_sim_out_df$iter <- as.numeric(row.names(RE_sim_out_df))
RE_sim_out_df$L <- RE_sim_out_df$val - min(RE_sim_out_df$val)

ggplot(RE_sim_out_df) +
  geom_path(aes(x = p1, y = p2),
            linetype = 2,
            colour = "grey") +
  geom_point(aes(x = p1, y = p2, group = iter),
             size = 2, colour = "grey") +
  geom_point(data = RE_sim_out_df[RE_sim_out_df$L <= qchisq(0.95, 2),],
             aes(x = p1, y = p2, colour = val, group = iter),
             size = 1.5) +
  scale_colour_viridis_c(option = "C") +
  theme_bw() +
  theme(text = element_text(size = 15)) +
  labs(x = "X UTM (centered)",
       y = "Y UTM (centered)",
       colour = expression(paste(Delta, "-2lnL <= ", chi^{2}, "(0.95, 2)")))
```

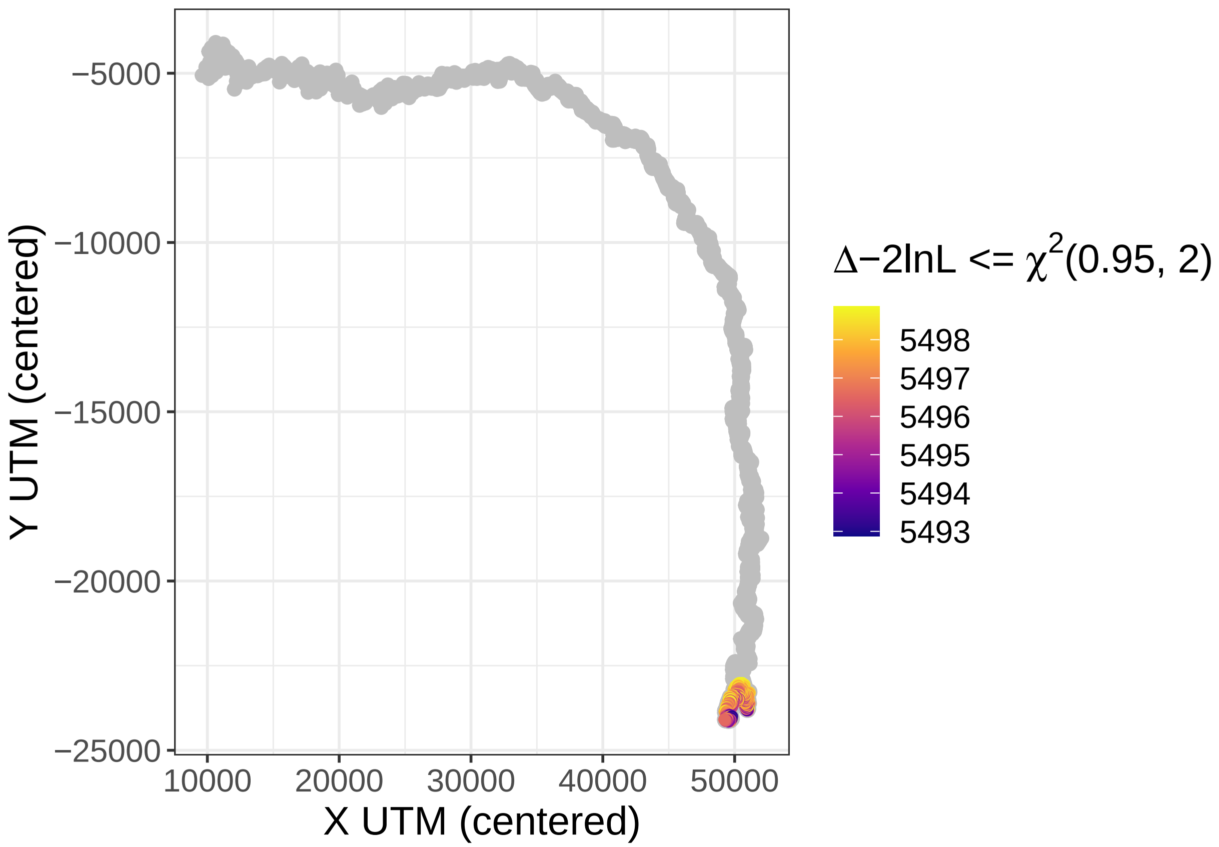

Figure 3

Visual summary of how SANN explored the parameter space for wave centroid location using 10,000 iterations. Each iteration is represented with a grey dot, the likelihood of all locations where $\Delta - 2lnL < \chi2(0.95, 2)$ are coloured according to their $-2lnL$.

```
ggplot(RE_sim_out_df) +
  geom_point(aes(x = p3, y = val, group = iter),
             size = 2, colour = "grey") +
  geom_point(data = RE_sim_out_df[RE_sim_out_df$L <= qchisq(0.95, 1),],
             aes(x = p3, y = val, colour = val, group = iter),
             size = 1.5) +
  scale_colour_viridis_c(option = "C") +
  theme_bw() +
  theme(text = element_text(size = 15)) +
  labs(x = "Speed (m per day)",
       y = "-2lnL",
       title = "RE Speed",
       colour = expression(paste(Delta, "-2lnL <= ", chi^{2}, "(0.95, 1)")))
```

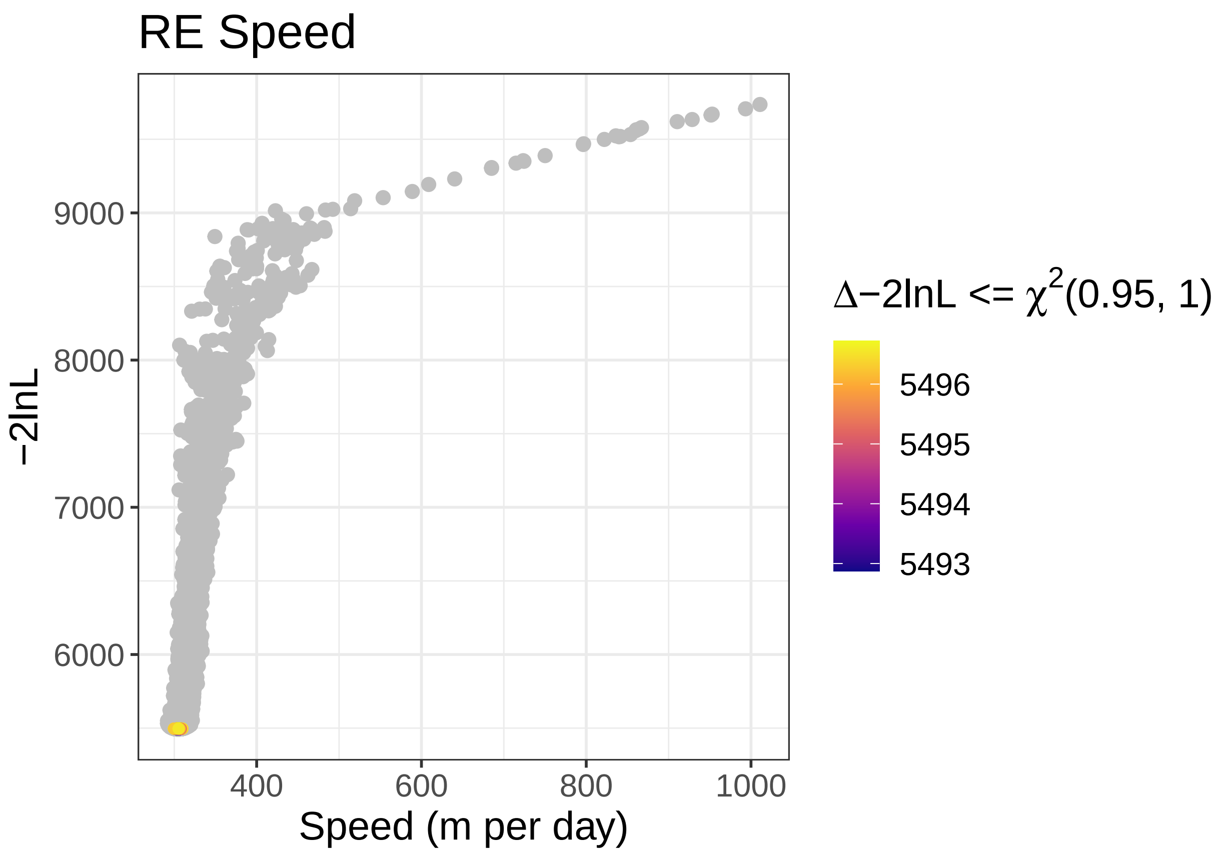

Figure 4

Shows the same as Figure 3, except for speed.

Predicted growth rate

Using the estimated parameter values, growth rates are predicted across space and time and compared with the true values.

```
df$D <- -sqrt((RE_sim_out$estimate[1] - df$x)^2 + (RE_sim_out$estimate[2] - df$y)^2)
df$rho <- df$t + (1 / RE_sim_out$estimate[3]) * df$D

RE_sim <- gam(r.growth ~ s(rho, k = 12, bs = "tp"),
          method = "ML",
          data = df,
          family = "gaussian")

p_spatial_RE_sim <- expand.grid(x = seq(min(df$x),
                                    max(df$x),
                                    length = 50),
                            y = seq(min(df$y),
                                    max(df$y),
                                    length = 50),
                            t = seq(min(df$t),
```

```
                                    max(df$t),
                                    length = 15)
)

p_spatial_RE_sim$D <- -sqrt((RE_sim_out$estimate[1] - p_spatial_RE_sim$x)^2 +
                              (RE_sim_out$estimate[2] - p_spatial_RE_sim$y)^2)
p_spatial_RE_sim$rho <- p_spatial_RE_sim$t +
  (1 / RE_sim_out$estimate[3]) * p_spatial_RE_sim$D

RE_fit <- data.frame(predict(RE_sim, newdata = p_spatial_RE_sim, se.fit = TRUE))

ind <- exclude.too.far(p_spatial_RE_sim$x, p_spatial_RE_sim$y,
                       df$x, df$y, dist = 0.1)

RE_fit$fit[ind] <- NA

RE_fit <- transform(RE_fit,
                    upper = fit + (2 * se.fit),
                    lower = fit - (2 * se.fit))

pred_RE <- cbind(p_spatial_RE_sim, RE_fit)

ggplot() +
  geom_tile(data = pred_RE, aes(x = x, y = y, fill = fit)) +
  scale_fill_viridis_c(option = "C", na.value = "transparent") +
  scale_x_continuous(label = scales::comma) +
  scale_y_continuous(label = scales::comma) +
  labs(x = "X",
       y = "Y",
       fill= expression(r[t]),
       subtitle = "Predicted spatial pattern over time") +
  theme_bw() +
  theme(text = element_text(size = 8),
             axis.text.x = element_text(angle = 60, vjust = 1, hjust=1)) +
  facet_wrap(~ round(t))
```

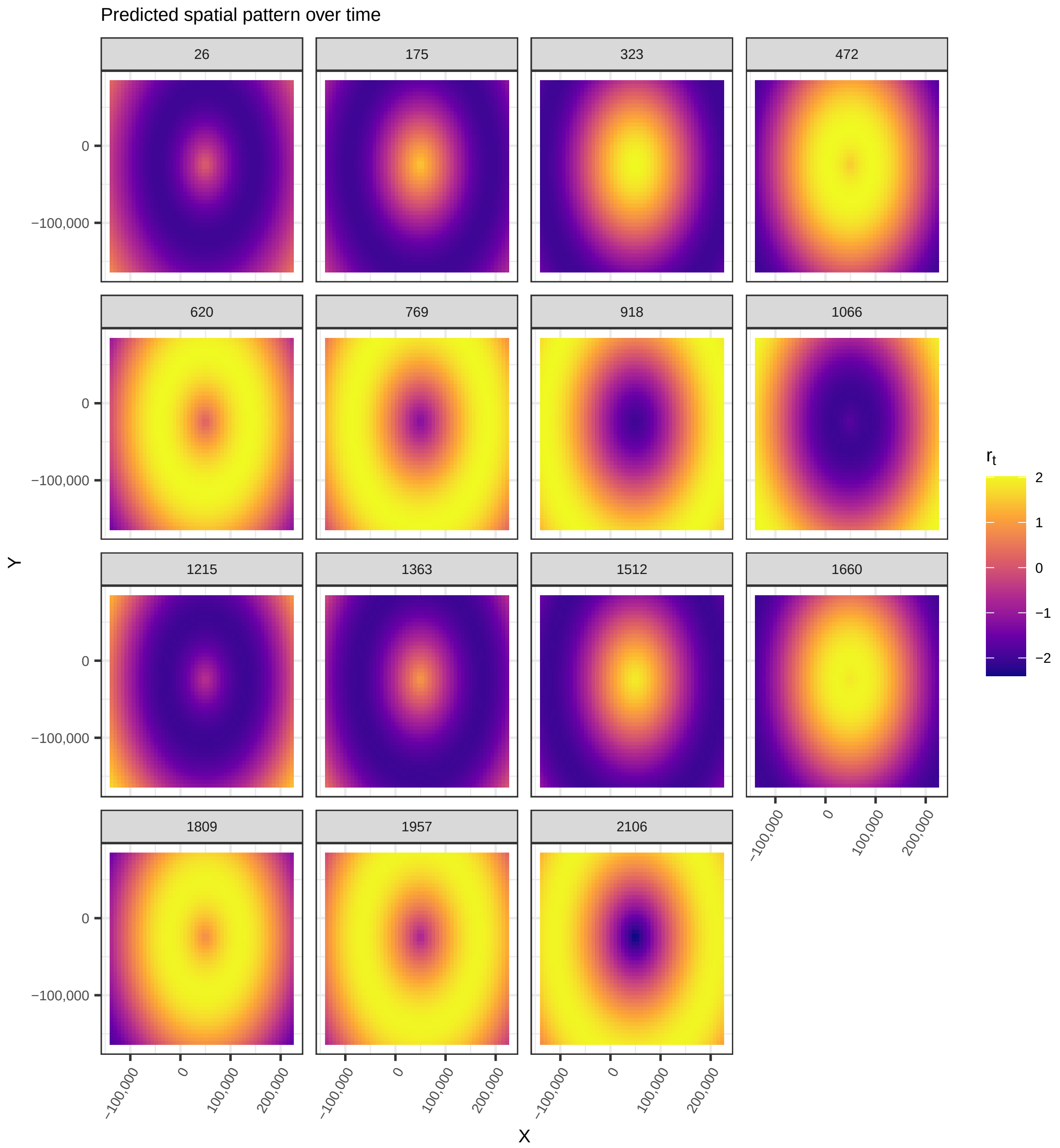

Figure 5

Spatio-temporal prediction based on the model fit. Each facet shows a given day, with X and Y axes showing mean centered UTM coordinates.

```
df_true$qrt <- as.integer(df_true$t/90) + 1

ggplot(df_true) +
  geom_point(aes(x = x, y = y, colour = r.growth)) +
  scale_colour_viridis_c(option = "C") +
```

```
scale_x_continuous(label= comma) +
scale_y_continuous(label= comma) +
facet_wrap(~qrt) +
theme_bw() +
theme(text = element_text(size = 8),
      axis.text.x = element_text(angle = 60, vjust = 1, hjust=1)) +
labs(x = "X UTM",
     y = "Y UTM",
     subtitle = "True pattern over time and space",
     colour = expression(r[t]))
```

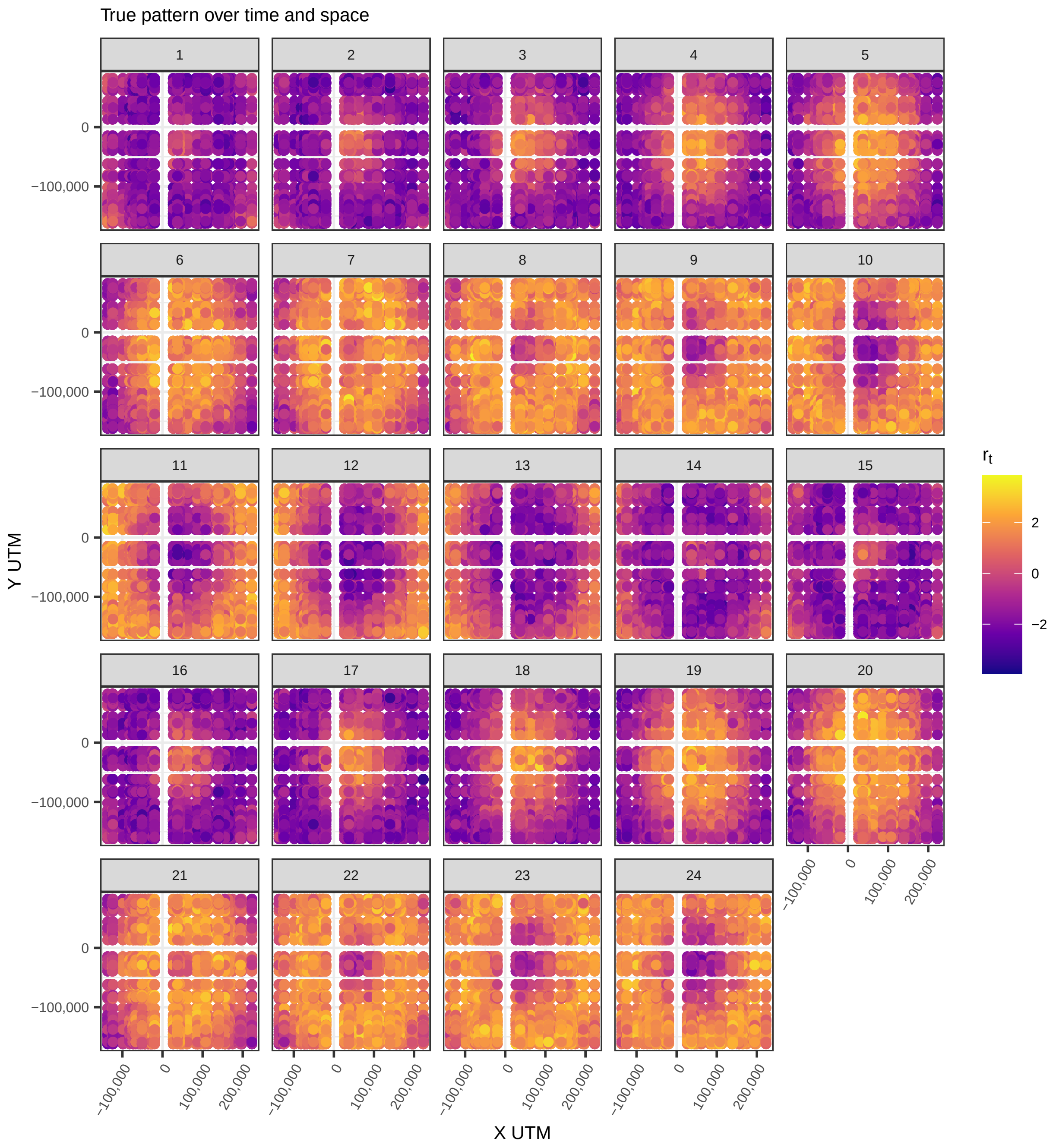

Figure 6

True spatio-temporal pattern in raw data showing all data points (not limited to the sampled data). Note that the time intervals (shown in each facet) here are yearly quarter, not day as in figure 5 As such, the spatial pattern in any given facet may not directly correspond to figure 5, however, across the different facets in each of the two figures the the overall pattern to be near identical.

Outcome of method with simulated data

Comparing the True values, simulated according to a single expanding radial wave without an adaptive monitoring scheme, with the Estimated parameter values (see the table below) we find that the method is able to reliably recover known parameter values.

```
RE_sim_est[,-7]
```

Parameter	Units	True	Estimate	Lower95CI	Upper95CI
Epicentre X	mean centred UTM	50,000	49,749	49,229	51,133
Epicentre Y	mean centred UTM	-25,000	-24,048	-24,148	-23,016
Speed	m per day	300	305	300	310

Visual summary of analytical process

Below, we outline a visual summary of the model fitting process. Initially, distance is calculated from the considered epicentre location for all centroids.

```
df$D <- -sqrt((RE_sim_out$estimate[1] - df$x)^2 + (RE_sim_out$estimate[2] - df$y)^2)

ggplot(df) +
  geom_point(aes(x = x, y = y, colour = D)) +
  geom_point(aes(x = RE_sim_out$estimate[1], y = RE_sim_out$estimate[2]),
             pch = 21, size = 3, colour = "black", fill = "grey") +
  scale_colour_viridis_c(option = "A", label = comma) +
  scale_x_continuous(label= comma) +
  scale_y_continuous(label= comma) +
  theme_bw() +
  theme(text = element_text(size = 15),
        axis.text.x = element_text(angle = 60, vjust = 1, hjust=1)) +
  labs(x = "X UTM (centered)",
       y = "Y UTM (centered)",
       colour = "Distance from\nepicentre")
```

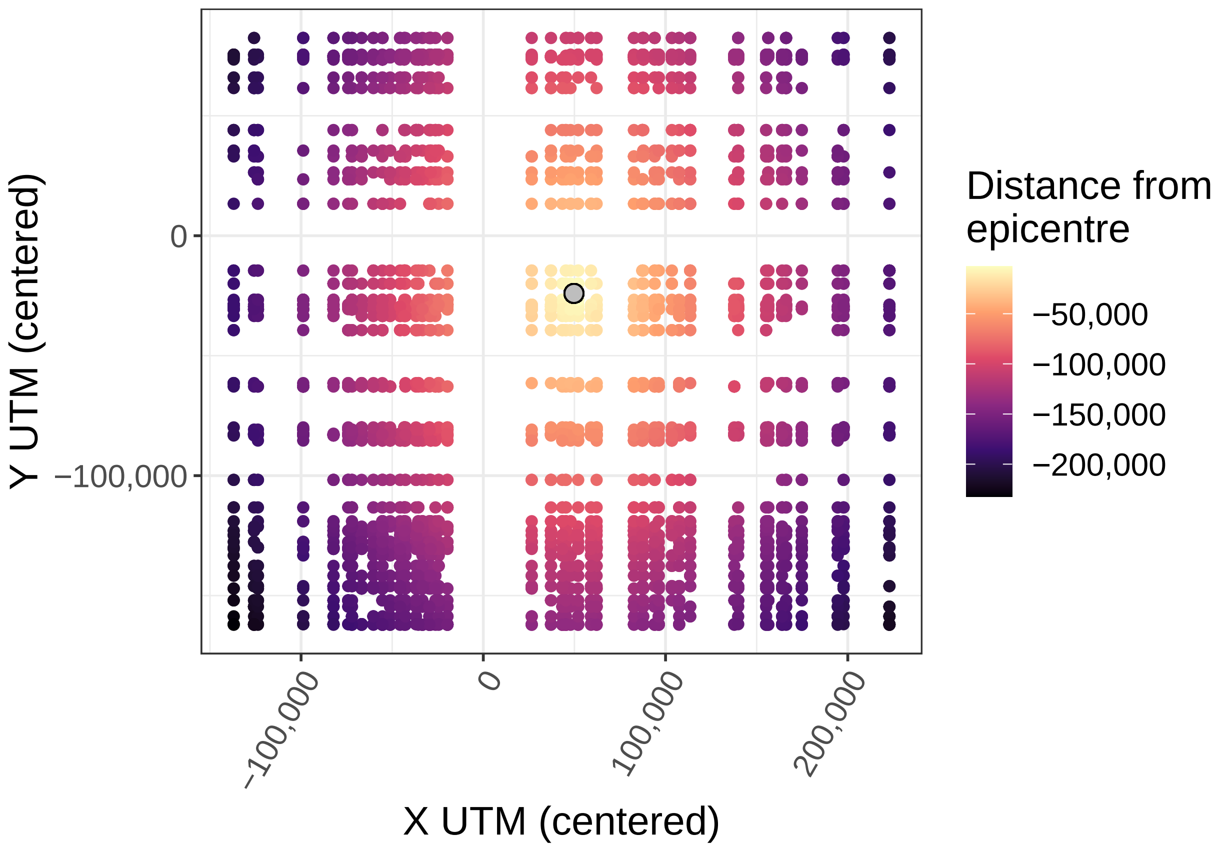

Figure 7

The first part of the modeling process (Distance equation, Table 1 in the main text) is to calculate distance from a proposed epicentre location, here the estimated epicentre location is shown with a grey dot. Populations in the landscape which are equally distant to the epicentre (i.e. the same distance) experience the same phase of a cycle. For instance, all populations 1 km away from the epicentre may experience high growth, while populations 100 km distant may still be experiencing low growth. By calculating distance from a proposed epicentre we account for such spatio-temporal lags.

While calculating distance from an epicentre allows us to identify populations which should be acting near synchronously, those populations will not be monitored continuously. Instead, population A (10 km distant) may be surveyed on day 1 while population B (also 10 km distant) may be surveyed only on day 100. As such, even though both population A and B are the same distance from the epicentre, the measurements taken from these two populations are unlikely different, as 99 days have passed since population A was surveyed. Therefore, we must take time into account.

```
df$rho <- df$t + (1 / RE_sim_out$estimate[3]) * df$D
df$rho_n <- cut_number(df$rho, 16)
df$D_n <- cut_number(df$D, 5)
a <- ggplot(df) +
  geom_point(aes(x = rho, y = r.growth),
             alpha = 0.1) +
  geom_smooth(aes(x = rho, y = r.growth, colour = D_n),
              method = "gam", show.legend = FALSE,
              formula = y ~ s(x, bs = "tp", k = 10), se = FALSE) +
```

```
  scale_x_continuous(label= comma) +
  scale_y_continuous(label= comma) +
  theme_bw() +
  theme(text = element_text(size = 15),
        axis.text.x = element_text(angle = 60, vjust = 1, hjust=1)) +
  labs(x = "Space-modified time",
       y = " ",
       subtitle = "GAM fit using space-modified time")

b <- ggplot(df) +
  geom_point(aes(x = t, y = r.growth),
             alpha = 0.1) +
  geom_smooth(aes(x = t, y = r.growth, colour = D_n),
              method = "gam", show.legend = FALSE,
              formula = y ~ s(x, bs = "tp", k = 10), se = FALSE) +
  scale_x_continuous(label= comma) +
  scale_y_continuous(label= comma) +
  theme_bw() +
  theme(text = element_text(size = 15),
        axis.text.x = element_text(angle = 60, vjust = 1, hjust=1)) +
  labs(x = "True time",
       y = "",
       subtitle = "GAM fit using true time,\nbut fit to binned distance")

c <- ggplot(df) +
  geom_point(aes(x = t, y = r.growth),
             alpha = 0.1) +
  scale_x_continuous(label= comma) +
  scale_y_continuous(label= comma) +
  theme_bw() +
  theme(text = element_text(size = 15),
        axis.text.x = element_text(angle = 60, vjust = 1, hjust=1)) +
  labs(x = "True time",
       y = expression(r[t]),
       subtitle = "Raw simulated data")

c / b / a + plot_annotation(
  caption = "Each coloured line is a spline fit to time according to binned distance from the estimated
  tag_levels = "A",
  tag_suffix = ")")
```

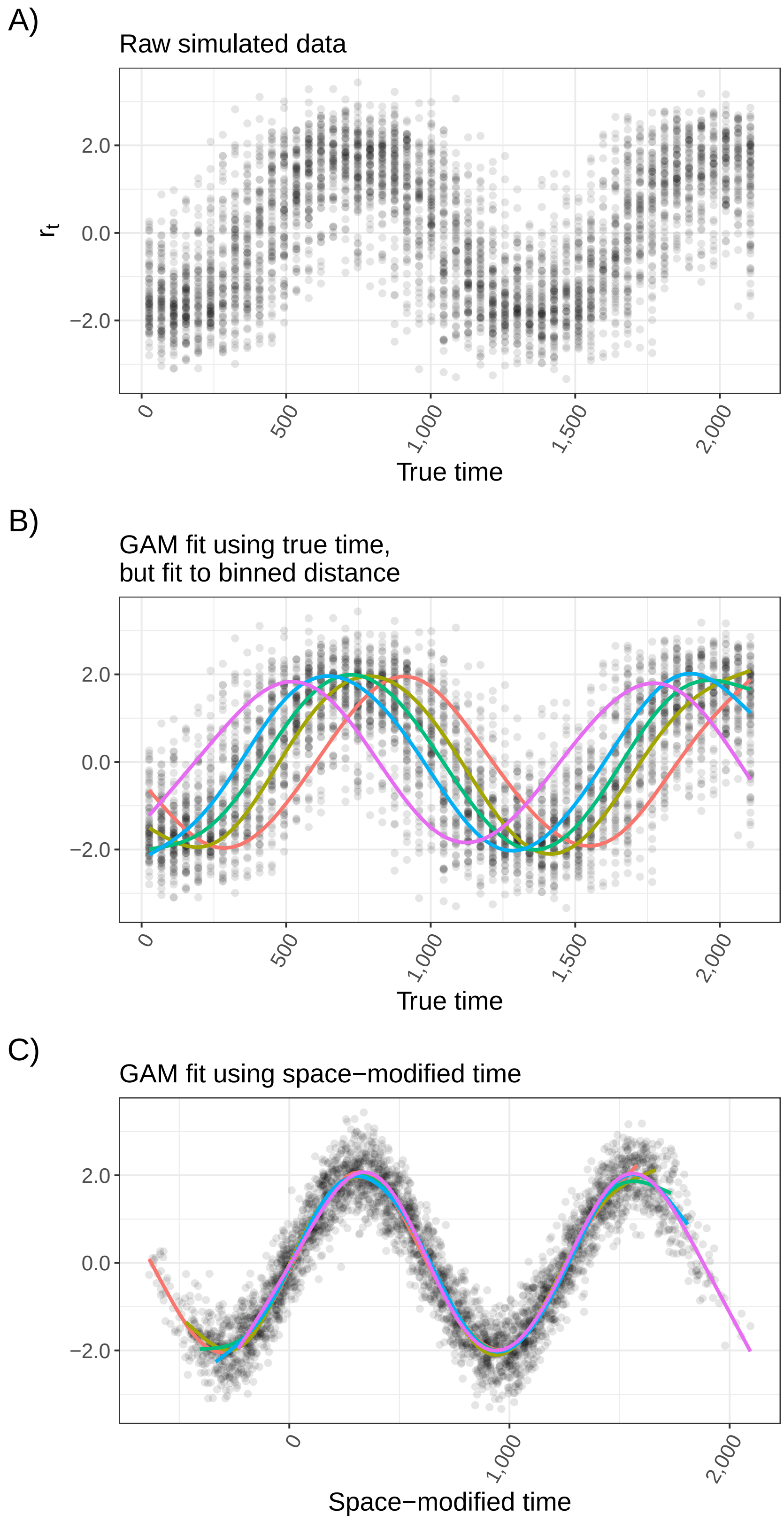

Each coloured line is a spline fit to time according to binned distance from the estimated epicentre location
A shows raw data,
s various splines fit to true time, but according to binned distance from epicentre (equivalent to Fig 1B in main text),
plines fit to space−modified time, but according to binned distance from epicentre (equivalent to Fig 1I in main text).

Figure 8

Each facet shows the progression in the analytical process from the initial partially synchronous data to recreated synchrony (see Fig. 1 in main text). A shows the raw simulated data as a time series. B shows the same raw data but with GAMs fit where growth is explained by time ($r_{i,t} = f(T_{i,t})$), but with separate splines fit according to binned distances from the epicentre (cut into 16 bins) (the equivalent to Figure 1B in main text). Note that B is shown to visually resemble Fig. 1 in the main text. C shows the same as in B, except that the GAMs are now fit using space modified time ($r_{i,t} = f(\rho_{i,t}), \rho_{it,} = T_{i,t} + \frac{1}{S} \times D_i$), and finally the resulting same but on space-modified time. Each line in C shows the splines for each binned distance, but these now overlap as a result of recreating synchrony.

Simulation with adaptive monitoring

The following simulation uses the same parameter values as in the above simulation. The only change here is that the sampling is carried out according to a simulated adapative monitoring scheme.

Select samples to analyse

Sampling is simulated using a Binomial distribution, as described above (Synopsis).

```
set.seed(1234)
# Using a binomial distribution to select samples
# Based on Rt, where higher growth increases probability to be sampled
# And based on an area where monitoring is arbitrarily focussed

b1 <- -5
b2 <- 0.8
b3 <- 7

# central monitoring area is in exact middle of arena
central_x <- 100000
central_y <- 50000
# central monitoring area has a radius of 30 km
  # Gives 3450 samples, close to real sample size
central_radius <- 25000

df_true$central <- ifelse((df_true$x - central_x)^2 +
                            (df_true$y - central_y)^2 < central_radius^2, 1, 0)

df_true$sampled_prob <- plogis(b1 + b2 * df_true$r.growth + b3 * df_true$central)

df_true$sampled <- rbinom(n = nrow(df_true), size = 1, prob = df_true$sampled_prob)
#
# ggplot(df_true) +
#   geom_jitter(aes(x = r.growth, y = sampled),
#               width = 0, height = 0.3, alpha = 0.1)

df_true$qrt <- as.integer(df_true$t/90) + 1

df <- df_true[df_true$sampled == 1,]
```

Raw simulated trend

```
ggplot(df) +
  geom_point(aes(x = t, y = r.growth, fill = r.growth),
             alpha = 0.3, pch = 21, colour = "black") +
  geom_hline(yintercept = 0, linetype = 2) +
  scale_fill_viridis_c(option = "C") +
  labs(y = expression(r[t,i]),
       x = "Mean day of data collection in centroid",
       fill = expression(r[t][i]),
       size = "Number of transects\nper centroid") +
  theme_classic() +
  NULL
```

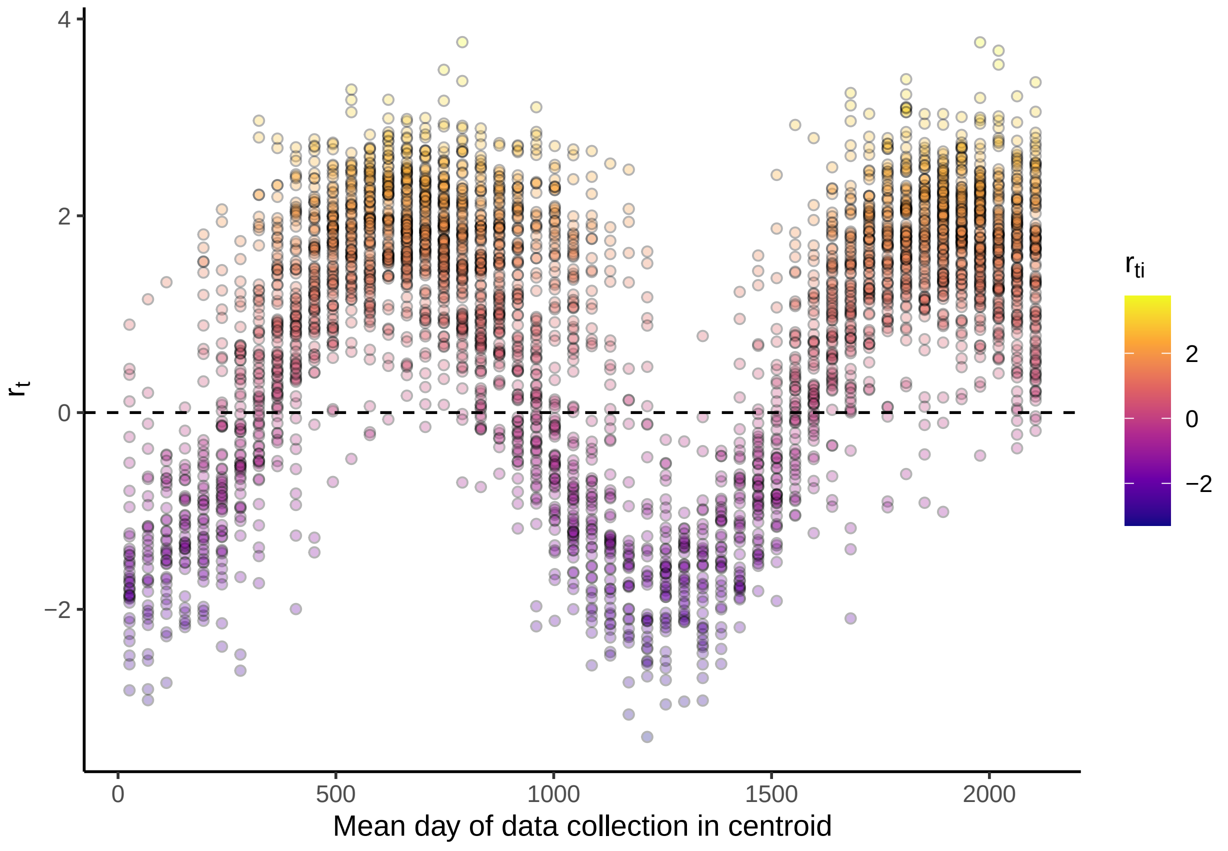

Figure 9

Simulated growth rate over time, as generated using the parameter values specified above. Frequency and amplitude of the sin wave were selected such that the simulated data would superficially resemble the real data. Data is more sparse during periods of low growth owing to the reactive nature of the monitoring.

```
# Yearly quarter calculated for plotting
  # Yearly quarter treated as 90 day period
df$qrt <- as.integer(df$t/90) + 1
```

```
ggplot(df) +
  geom_point(aes(x = x, y = y, colour = r.growth)) +
  scale_colour_viridis_c(option = "C") +
  scale_x_continuous(label= comma) +
  scale_y_continuous(label= comma) +
  facet_wrap(~qrt) +
  theme_bw() +
  theme(text = element_text(size = 8),
        axis.text.x = element_text(angle = 60, vjust = 1, hjust=1)) +
  labs(x = "X UTM",
       y = "Y UTM",
       colour = expression(r[t]))
```

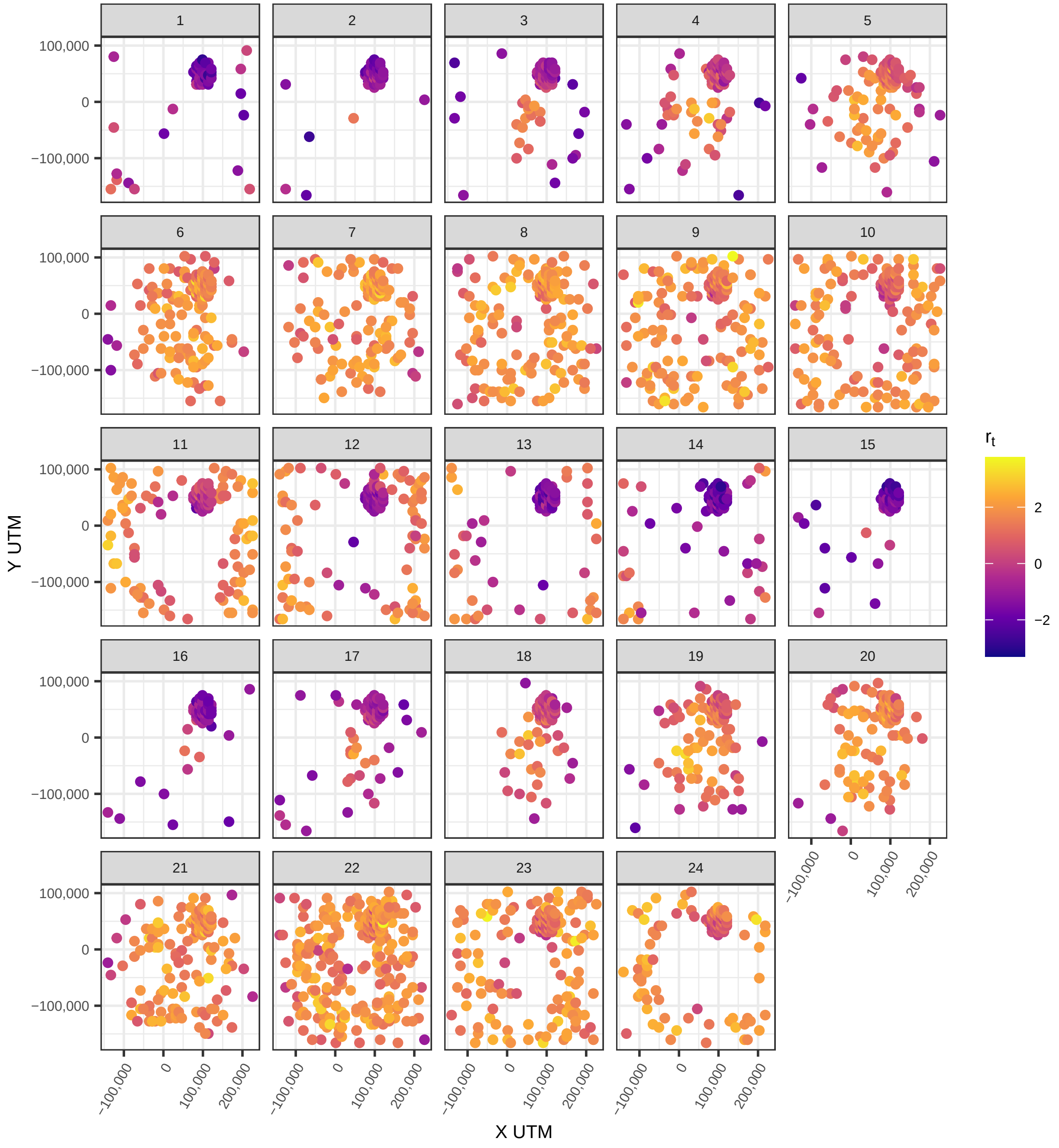

Figure 10

Growth rate across space in yearly quarters.

Parameter estimation

The data is fed into the appropriate model (model RE). If the statistical method is reliable, then this should retrieve parameter estimates close to the known, true values.

The SANN optimiser is run using 10,000 iterations (as opposed to 15,000 used with real analysis to reduce run time).

```
set.seed(1234)
# Function to run the model
RE_fun_sim <- function(par, data = df) {
  df$D_est <- -sqrt((par[1] - df$x)^2 + (par[2] - df$y)^2)
  df$rho_est <- df$t + (1 / par[3]) * df$D_est

  -logLik.gam(gam(r.growth ~ s(rho_est, k = 12, bs = "tp"),
                  method = "ML",
                  data = df,
                  family = "gaussian")
  )[1]
}

# Specifying the initial values
par_list_RE_sim <- c(10000, -5000, 1000)

# Recording start time of model run
start_time <- Sys.time()

# Running the model as in formal analysis
RE_sim_out <- metropSB(fn = RE_fun_sim,
                   start = par_list_RE_sim,
                   nmax = niter,
                   retvals = TRUE,
                   retfreq = 1,
                   verbose = FALSE,
                   rptfreq = -1)

# Report total run time
Sys.time() - start_time
```

```
## Time difference of 57.53653 mins
```

The parameter values are then extracted along with the 95% confidence intervals.

```
set.seed(1234)
# Extract parameter space from SANN
RE_sim_out_df <- data.frame(RE_sim_out$retvals[,c(1:3, 10)])

# Convert negative log likelihood to -2lnL
RE_sim_out_df$val <- 2*RE_sim_out_df$val

# Create empty dataframe
RE_sim_profile <- data.frame(
  p1 = NA,
  p2 = NA,
  p3 = NA,
  val = NA
)
```

```
# For each unique speed considered by SANN
for(i in 1:length(unique(RE_sim_out_df$p3))) {

  # Store all unique values of speed
  match <- unique(RE_sim_out_df$p3)

  # Store all parameter combinations for a given speed considered
  temp <- subset(RE_sim_out_df, p3 == match[i])

  # Extract all parameter combinations where L is maximised
  temp1 <- temp[temp$val == max(temp$val),]

  # combine with previously created RE_sim_profile
  RE_sim_profile <- rbind(RE_sim_profile, temp1)
}

# Remove first NA entry when creating dataframe
RE_sim_profile <- RE_sim_profile[-1,]

# Find where -2lnL is best
lnLmax <- RE_sim_profile$val[RE_sim_profile$val == min(RE_sim_profile$val)]

# Calculate difference for each lnL from best
RE_sim_profile$lnL_diff <- RE_sim_profile$val - lnLmax

# For all parameter combinations, extract those that fall within chisq 95% CI
p3ci_profile_df <- RE_sim_profile[RE_sim_profile$lnL_diff < qchisq(p = 0.95, df = 1),]
p3min <- min(p3ci_profile_df$p3)
p3max <- max(p3ci_profile_df$p3)


# Repeat above, but for centroid coordinates
RE_sim_out_df$centroid <- paste(RE_sim_out_df$p1, RE_sim_out_df$p2, sep = " ")
RE_sim_profile <- data.frame(
  p1= NA,
  p2 = NA,
  p3 = NA,
  val = NA,
  centroid = NA
)
for(i in 1:length(unique(RE_sim_out_df$centroid))) {
  match <- unique(RE_sim_out_df$centroid)
  temp <- subset(RE_sim_out_df, centroid == match[i])
  temp1 <- temp[temp$val == max(temp$val),]
  RE_sim_profile <- rbind(RE_sim_profile, temp1)
}
RE_sim_profile <- RE_sim_profile[-1,]
lnLmax <- RE_sim_profile$val[RE_sim_profile$val == min(RE_sim_profile$val)]
RE_sim_profile$lnL_diff <- RE_sim_profile$val - lnLmax

# Note that CI is based on 2 degrees of freedom from chisq
ci_profile_df <- RE_sim_profile[RE_sim_profile$lnL_diff < qchisq(p = 0.95, df = 2),]
p1min <- min(ci_profile_df$p1)
```

```r
p1max <- max(ci_profile_df$p1)
p2min <- min(ci_profile_df$p2)
p2max <- max(ci_profile_df$p2)
RE_sim_est <- data.frame(
  Parameter = c("Epicentre X", "Epicentre Y", "Speed"),
  Units = c("mean centred UTM", "mean centred UTM", "m per day"),
  True = comma(c(x_wave, y_wave, speed)),
  Estimate = comma(RE_sim_out$estimate),
  Lower95CI = comma(c(p1min, p2min, p3min)),
  Upper95CI = comma(c(p1max, p2max, p3max)),
  Initial = comma(par_list_RE_sim)
)
RE_sim_est
```

Parameter	Units	True	Estimate	Lower95CI	Upper95CI	Initial
Epicentre X	mean centred UTM	50,000	49,631	48,437	50,162	10,000
Epicentre Y	mean centred UTM	-25,000	-23,885	-23,930	-22,922	-5,000
Speed	m per day	300	310	305	317	1,000

Table 2

The above table shows the true parameter values, the estimated parameter values and their 95% CI, as well as the value which the SANN optimiser was initiated at.

Optimsation process

```r
RE_sim_out_df$iter <- as.numeric(row.names(RE_sim_out_df))
RE_sim_out_df$L <- RE_sim_out_df$val - min(RE_sim_out_df$val)

ggplot(RE_sim_out_df) +
  geom_path(aes(x = p1, y = p2),
            linetype = 2,
            colour = "grey") +
  geom_point(aes(x = p1, y = p2, group = iter),
             size = 2, colour = "grey") +
  geom_point(data = RE_sim_out_df[RE_sim_out_df$L <= qchisq(0.95, 2),],
             aes(x = p1, y = p2, colour = val, group = iter),
             size = 1.5) +
  scale_colour_viridis_c(option = "C") +
  theme_bw() +
  theme(text = element_text(size = 15)) +
  labs(x = "X UTM (centered)",
       y = "Y UTM (centered)",
       colour = expression(paste(Delta, "-2lnL <= ", chi^{2}, "(0.95, 2)")))
```

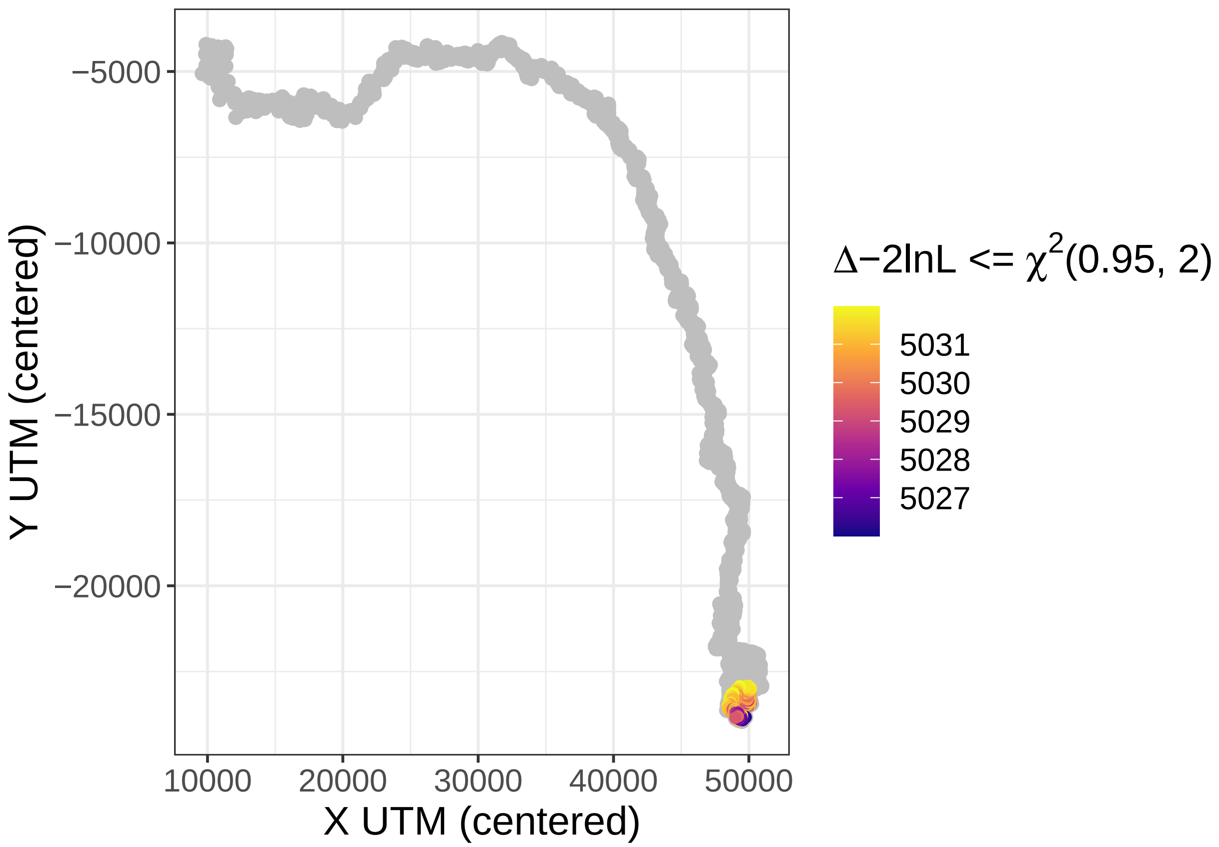

Figure 11

Visual summary of how SANN explored the parameter space for wave centroid location using 10,000 iterations. Each iteration is represented with a grey dot, the likelihood of all locations where $\Delta - 2lnL < \chi2(0.95, 2)$ are coloured according to their $-2lnL$.

```
ggplot(RE_sim_out_df) +
  geom_point(aes(x = p3, y = val, group = iter),
             size = 2, colour = "grey") +
  geom_point(data = RE_sim_out_df[RE_sim_out_df$L <= qchisq(0.95, 1),],
             aes(x = p3, y = val, colour = val, group = iter),
             size = 1.5) +
  scale_colour_viridis_c(option = "C") +
  theme_bw() +
  theme(text = element_text(size = 15)) +
  labs(x = "Speed (m per day)",
       y = "-2lnL",
       title = "RE Speed",
       colour = expression(paste(Delta, "-2lnL <= ", chi^{2}, "(0.95, 1)")))
```

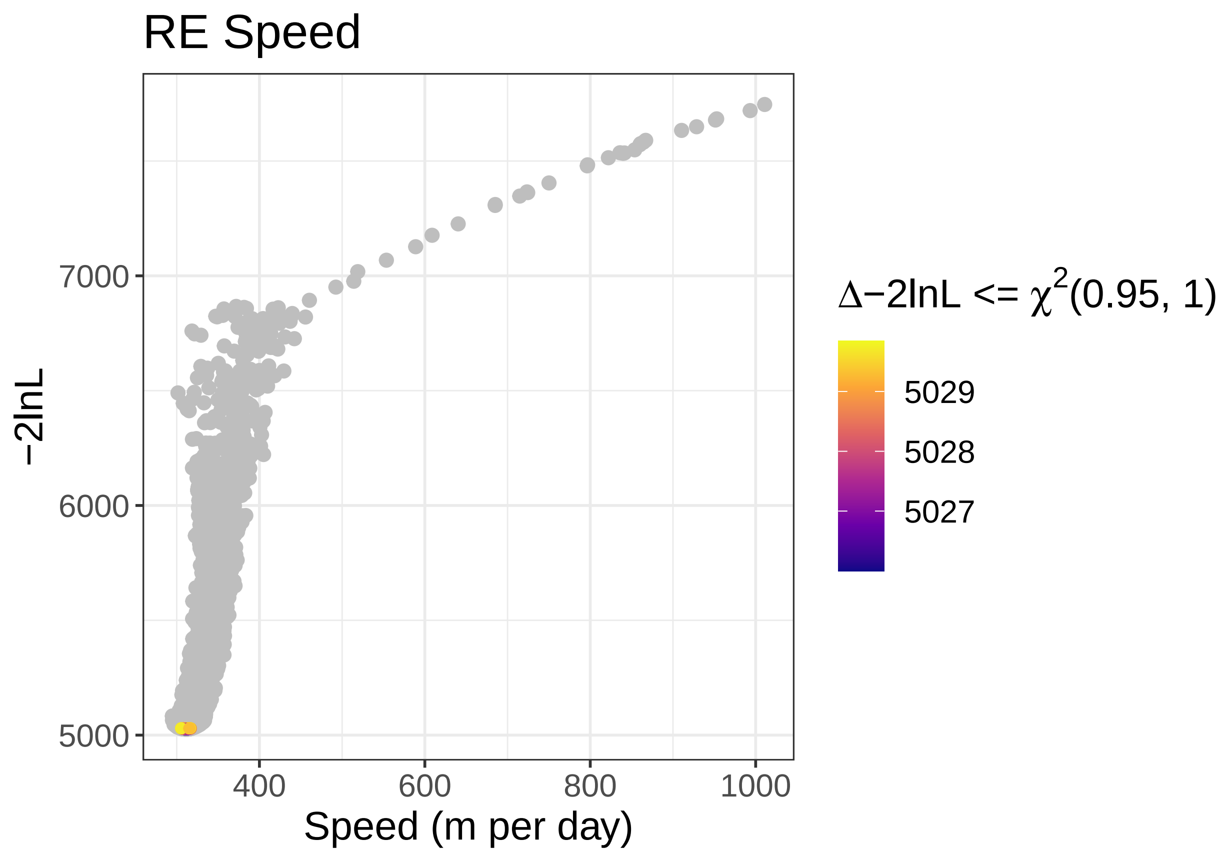

Figure 12

Shows the same as Figure 11, except for speed.

Predicted growth rate

Using the estimated parameter values, growth rates are predicted across space and time and compared with the true values.

```
df$D <- -sqrt((RE_sim_out$estimate[1] - df$x)^2 + (RE_sim_out$estimate[2] - df$y)^2)
df$rho <- df$t + (1 / RE_sim_out$estimate[3]) * df$D

RE_sim <- gam(r.growth ~ s(rho, k = 12, bs = "tp"),
          method = "ML",
          data = df,
          family = "gaussian")

p_spatial_RE_sim <- expand.grid(x = seq(min(df$x),
                                        max(df$x),
                                        length = 50),
                                y = seq(min(df$y),
                                        max(df$y),
                                        length = 50),
                                t = seq(min(df$t),
```

```
                                    max(df$t),
                                    length = 15)
)

p_spatial_RE_sim$D <- -sqrt((RE_sim_out$estimate[1] - p_spatial_RE_sim$x)^2 +
                              (RE_sim_out$estimate[2] - p_spatial_RE_sim$y)^2)
p_spatial_RE_sim$rho <- p_spatial_RE_sim$t +
  (1 / RE_sim_out$estimate[3]) * p_spatial_RE_sim$D

RE_fit <- data.frame(predict(RE_sim, newdata = p_spatial_RE_sim, se.fit = TRUE))

ind <- exclude.too.far(p_spatial_RE_sim$x, p_spatial_RE_sim$y,
                       df$x, df$y, dist = 0.1)

RE_fit$fit[ind] <- NA

RE_fit <- transform(RE_fit,
                    upper = fit + (2 * se.fit),
                    lower = fit - (2 * se.fit))

pred_RE <- cbind(p_spatial_RE_sim, RE_fit)

ggplot() +
  geom_tile(data = pred_RE, aes(x = x, y = y, fill = fit)) +
  scale_fill_viridis_c(option = "C", na.value = "transparent") +
  scale_x_continuous(label = scales::comma) +
  scale_y_continuous(label = scales::comma) +
  labs(x = "X",
       y = "Y",
       fill= expression(r[t]),
       title = "RE spatial pattern over time") +
  theme_bw() +
  theme(text = element_text(size = 15),
        axis.text.x = element_text(angle = 60, vjust = 1, hjust=1)) +
  facet_wrap(~ round(t))
```

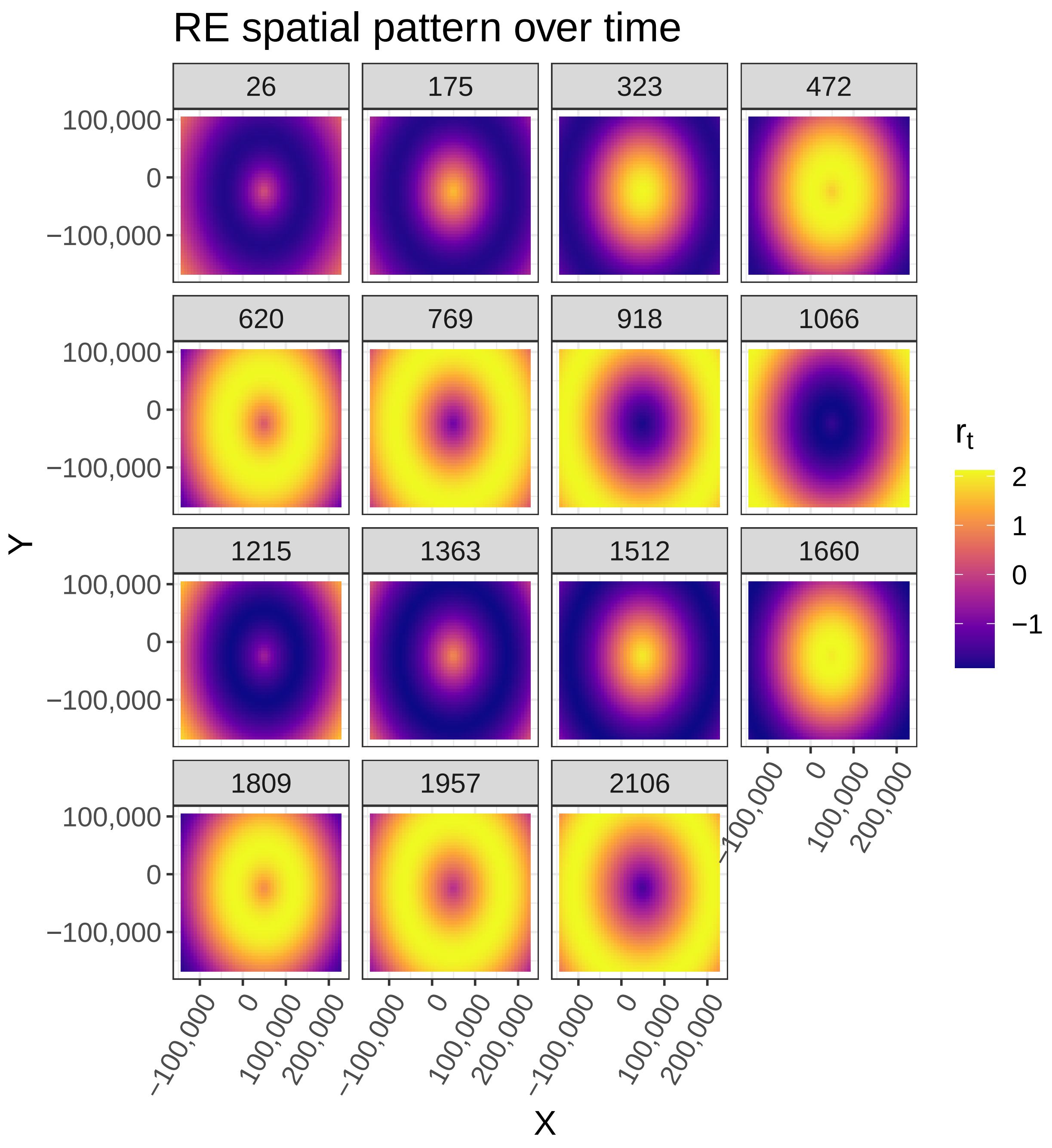

Figure 13

Spatio-temporal prediction based on the model fit. Each facet shows a given day, with X and Y axes showing mean centered UTM coordinates.

Using the estimated parameter values, growth rates are predicted across space and time.

```
df_true$qrt <- as.integer(df_true$t/90) + 1

ggplot(df_true) +
```

```
  geom_point(aes(x = x, y = y, colour = r.growth)) +
  scale_colour_viridis_c(option = "C") +
  scale_x_continuous(label= comma) +
  scale_y_continuous(label= comma) +
  facet_wrap(~qrt) +
  theme_bw() +
  theme(text = element_text(size = 15),
        axis.text.x = element_text(angle = 60, vjust = 1, hjust=1)) +
  labs(x = "X UTM",
       y = "Y UTM",
       colour = expression(r[t]))
```

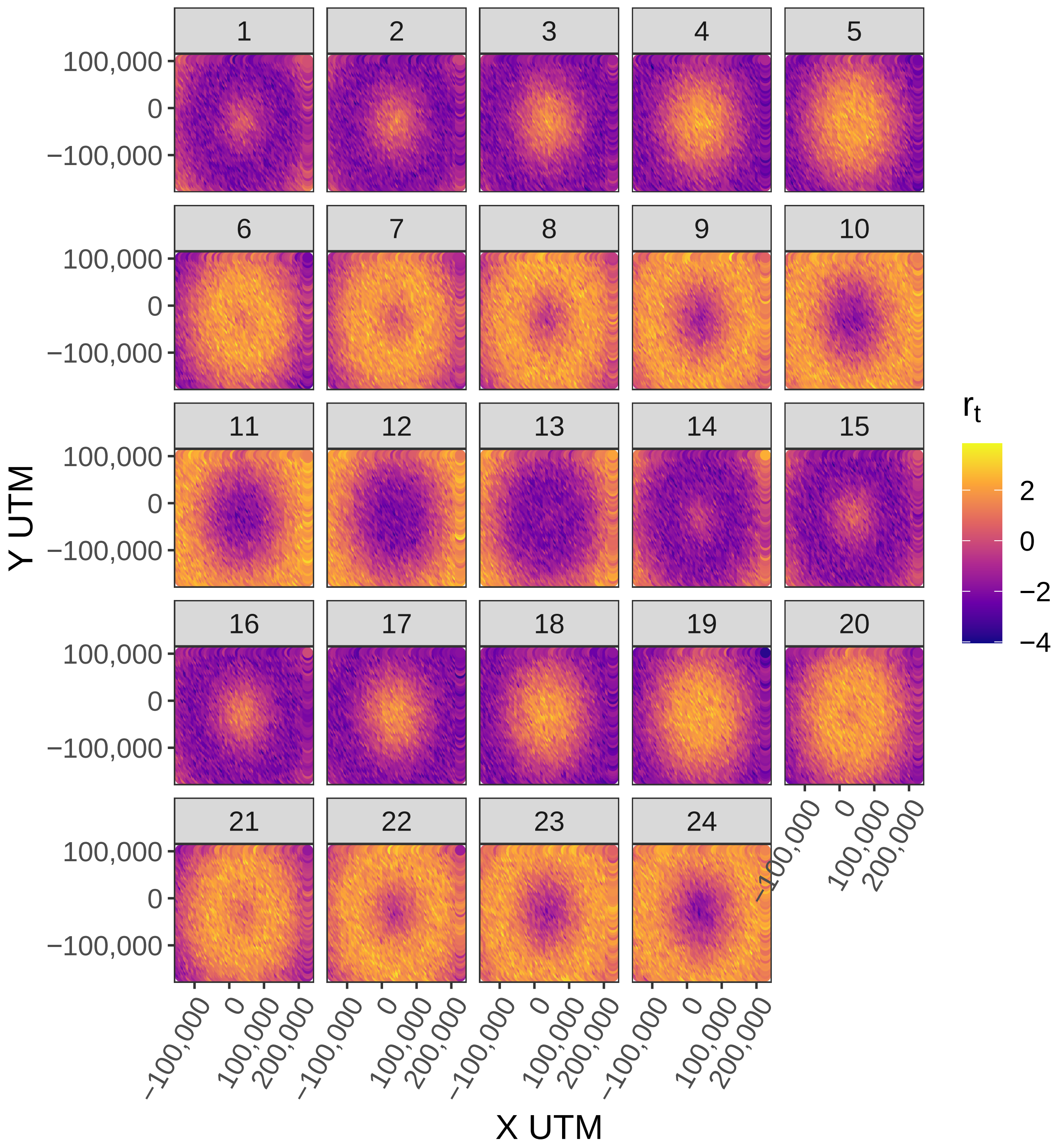

Figure 14

True spatio-temporal pattern in raw data showing all data points (not limited to the sampled data). Note that the time intervals (shown in each facet) here are yearly quarter, not day as in figure 13. As such, the spatial pattern in any given facet may not directly correspond to figure 5, however, across the different facets in each of the two figures the the overall pattern to be near identical.

Outcome of method with simulated data with adapative monitoring

Comparing the True values, simulated according to a single expanding radial wave with an adaptive monitoring scheme, with the Estimated parameter values (see the table below) we find that the method is able to reliably recover known parameter values, irrespective of an adaptive monitoring scheme. The reason for this, is that although there may be locations and periods with very few samples, in truth all samples contribute to the estimation of parameters, regardless of where they are in space (so long as spatial resolution and scale is suitably fine).

```
RE_sim_est[,-7]
```

Parameter	Units	True	Estimate	Lower95CI	Upper95CI
Epicentre X	mean centred UTM	50,000	49,631	48,437	50,162
Epicentre Y	mean centred UTM	-25,000	-23,885	-23,930	-22,922
Speed	m per day	300	310	305	317

Session information

```
sessionInfo()
```

```
## R version 4.1.0 (2021-05-18)
## Platform: x86_64-w64-mingw32/x64 (64-bit)
## Running under: Windows 10 x64 (build 19043)
##
## Matrix products: default
##
## locale:
## [1] LC_COLLATE=English_United Kingdom.1252
## [2] LC_CTYPE=English_United Kingdom.1252
## [3] LC_MONETARY=English_United Kingdom.1252
## [4] LC_NUMERIC=C
## [5] LC_TIME=English_United Kingdom.1252
##
## attached base packages:
## [1] stats     graphics  grDevices utils     datasets  methods   base
##
## other attached packages:
## [1] dplyr_1.0.7     patchwork_1.1.1 scales_1.1.1    ggplot2_3.3.5
## [5] emdbook_1.3.12  mgcv_1.8-35     nlme_3.1-152
##
## loaded via a namespace (and not attached):
##  [1] tidyselect_1.1.2    xfun_0.30           purrr_0.3.4
##  [4] splines_4.1.0       lattice_0.20-44     colorspace_2.0-1
##  [7] vctrs_0.3.8         generics_0.1.2      viridisLite_0.4.0
## [10] htmltools_0.5.2     stats4_4.1.0        yaml_2.2.1
## [13] utf8_1.2.1          rlang_1.0.2         pillar_1.7.0
## [16] withr_2.5.0         DBI_1.1.2           glue_1.4.2
## [19] lifecycle_1.0.1     plyr_1.8.6          stringr_1.4.0
## [22] munsell_0.5.0       gtable_0.3.0        bdsmatrix_1.3-4
```

```
## [25] mvtnorm_1.1-3       coda_0.19-4         evaluate_0.15
## [28] labeling_0.4.2      knitr_1.37          fastmap_1.1.0
## [31] fansi_0.4.2         highr_0.9           Rcpp_1.0.7
## [34] farver_2.1.0        digest_0.6.27       stringi_1.6.1
## [37] numDeriv_2016.8-1.1 grid_4.1.0          cli_3.2.0
## [40] tools_4.1.0         bbmle_1.0.24        magrittr_2.0.1
## [43] tibble_3.1.2        crayon_1.5.0        pkgconfig_2.0.3
## [46] MASS_7.3-54         ellipsis_0.3.2      Matrix_1.3-3
## [49] assertthat_0.2.1    rmarkdown_2.13      rstudioapi_0.13
## [52] R6_2.5.1            compiler_4.1.0
```
