# Supplementary material for: From pattern to process? Dual travelling waves, with contrasting propagation speeds, best describe a self‐organised spatio‐temporal pattern in population growth of a cyclic rodent
Source: Ecol Lett. 2022 Jul 31;25(9):1986–98. doi: 10.1111/ele.14074 (PMC9543711; doi:10.1111/ele.14074)
Supplement: Supplementary file 4 — Supinfo S3 [file ELE-25-1986-s004.pdf]

Roos et al 2021_Model checks

Deon Roos et al.

Last run of 2021-05-31

Contents

Model checks **2**

Null models . . . 2

N1 . . . 2

N2 . . . 3

N3 . . . 4

Travelling wave models . . . 4

P . . . 5

RE . . . 5

RC . . . 6

RFE . . . 7

RDE . . . 8

RFC . . . 9

RDC . . . 10

PF . . . 11

PD . . . 12

Model summaries . . . 13

N1 . . . 13

N2 . . . 14

N3 . . . 14

P . . . 15

RE . . . 15

RC . . . 16

RFE . . . 16

RDE . . . 17

RFC . . . 18

RDC . . . 18

PF 19
PD 19
Deviance explained 20
RDE residual plots 21

Packages used:

```
library(mgcv)       # for gams
library(beepr)      # beeps when optimisation is done
library(emdbook)    # Bolker's package for stochastic annealing optimiser method
library(ggplot2)    # For visualisation
library(gganimate) # For animations
library(scales)     # For comma scales
library(patchwork) # For plotting side-by-side
library(dplyr)      # For sample_n
```

Model checks

Null models

N1

```
gam.check(N1)
```

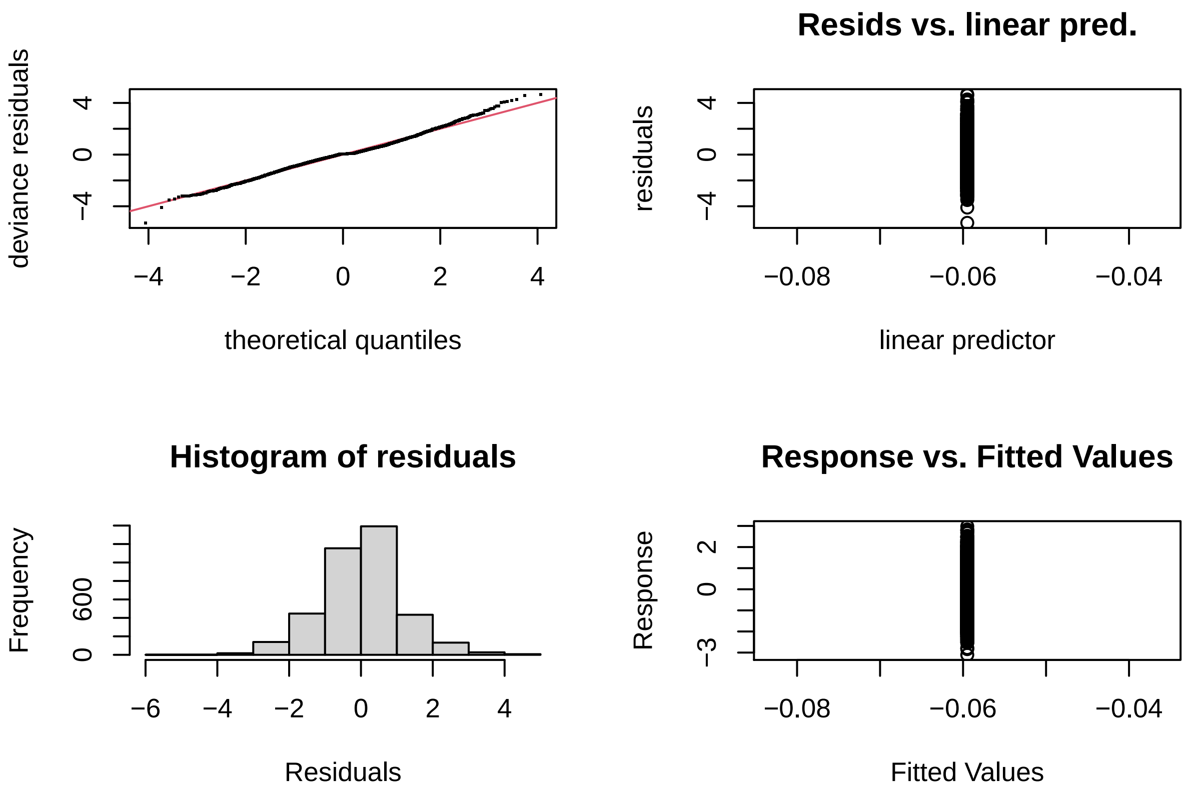

```
##
## Method: ML   Optimizer: outer newton
## full convergence after 6 iterations.
## Gradient range [-8.60777e-06,-8.60777e-06]
## (score 4677.042 & scale 1.240568).
## Hessian positive definite, eigenvalue range [1875.5,1875.5].
## Model rank =  1 / 1
```

N2

```r
gam.check(N2)
```

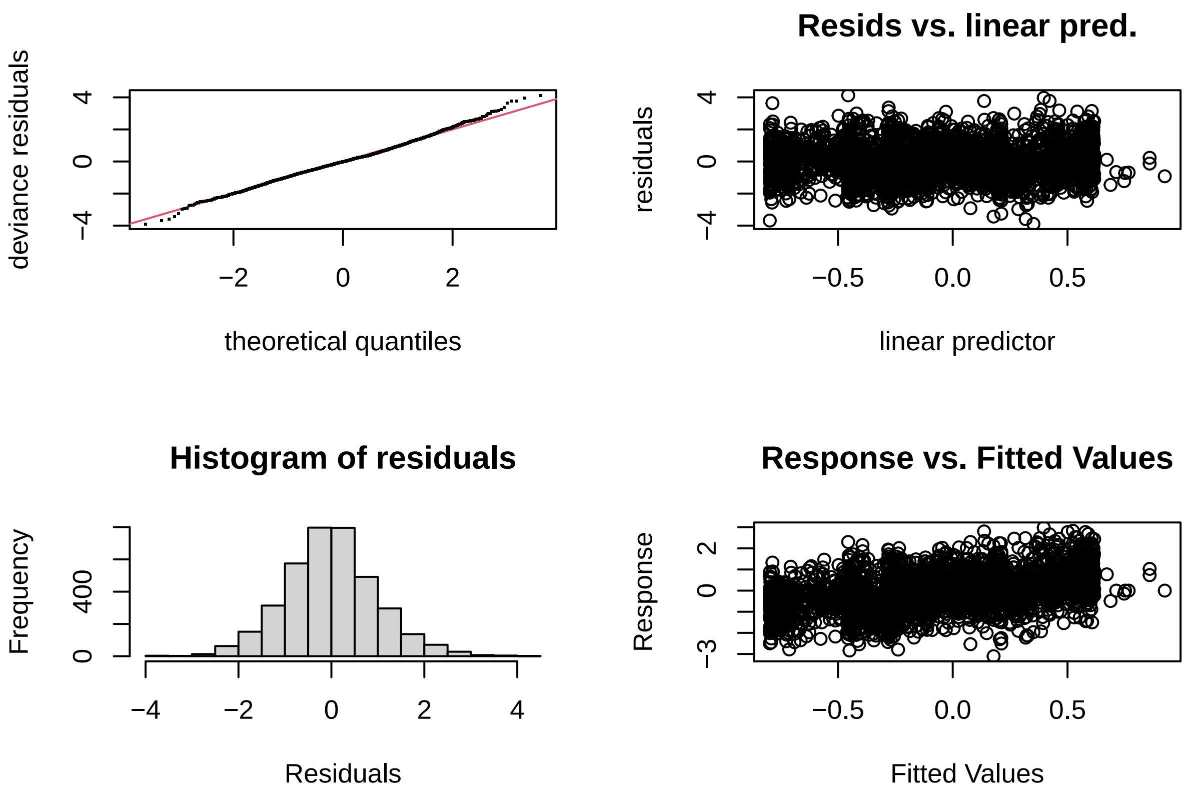


```
##
## Method: ML   Optimizer: outer newton
## full convergence after 7 iterations.
## Gradient range [-0.0003388366,0.0003081304]
## (score 4265.177 & scale 0.9776244).
## Hessian positive definite, eigenvalue range [4.750503,1875.514].
## Model rank =  12 / 12
##
## Basis dimension (k) checking results. Low p-value (k-index<1) may
## indicate that k is too low, especially if edf is close to k'.
##
##                          k'   edf k-index p-value
```

```
## s(julian.mean.trans) 11.0 10.9    0.94  <2e-16 ***
## ---
## Signif. codes:  0 '***' 0.001 '**' 0.01 '*' 0.05 '.' 0.1 ' ' 1
```

N3

```
gam.check(N3)
```

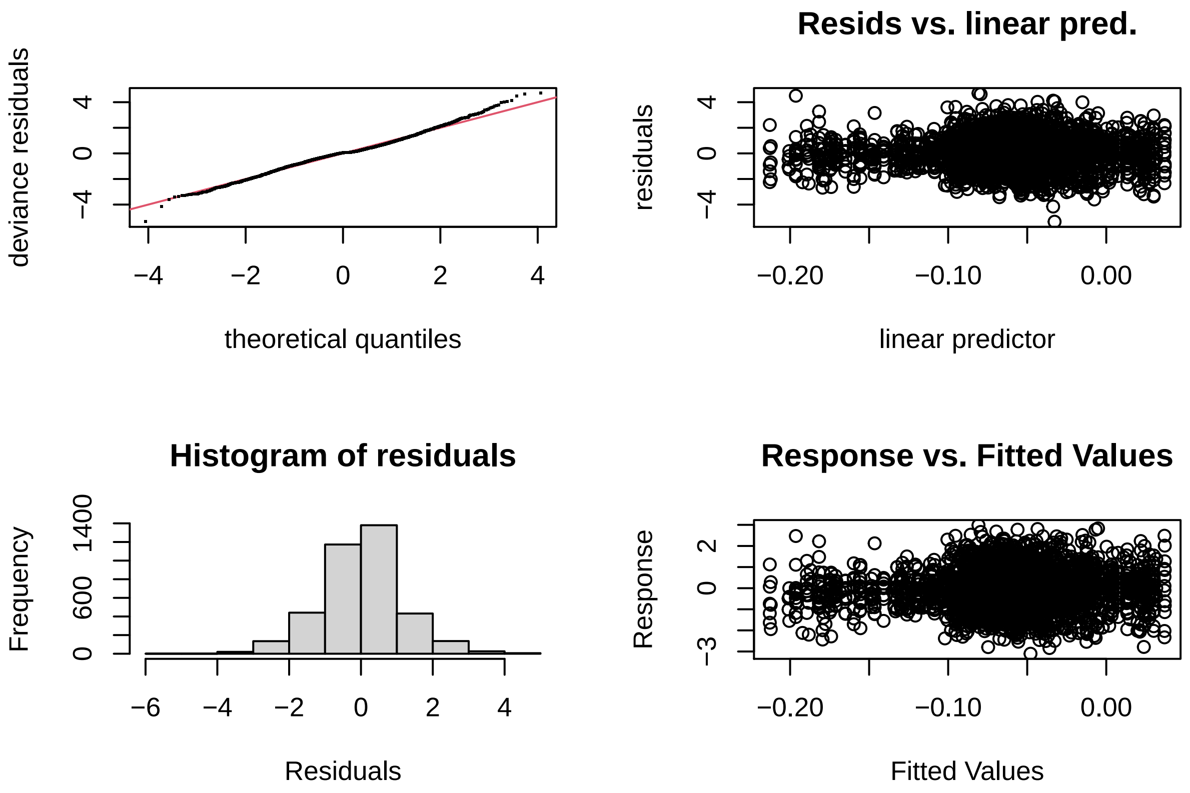


```
##
## Method: ML   Optimizer: outer newton
## full convergence after 8 iterations.
## Gradient range [-0.0009046154,0.002779714]
## (score 4672.468 & scale 1.238535).
## Hessian positive definite, eigenvalue range [0.0006902296,1875.497].
## Model rank =  100 / 100
##
## Basis dimension (k) checking results. Low p-value (k-index<1) may
## indicate that k is too low, especially if edf is close to k'.
##
##                    k'   edf k-index p-value
## te(cen.x,cen.y) 99.00  3.01    1.06       1
```

Travelling wave models

P

```r
gam.check(P_TW)
```

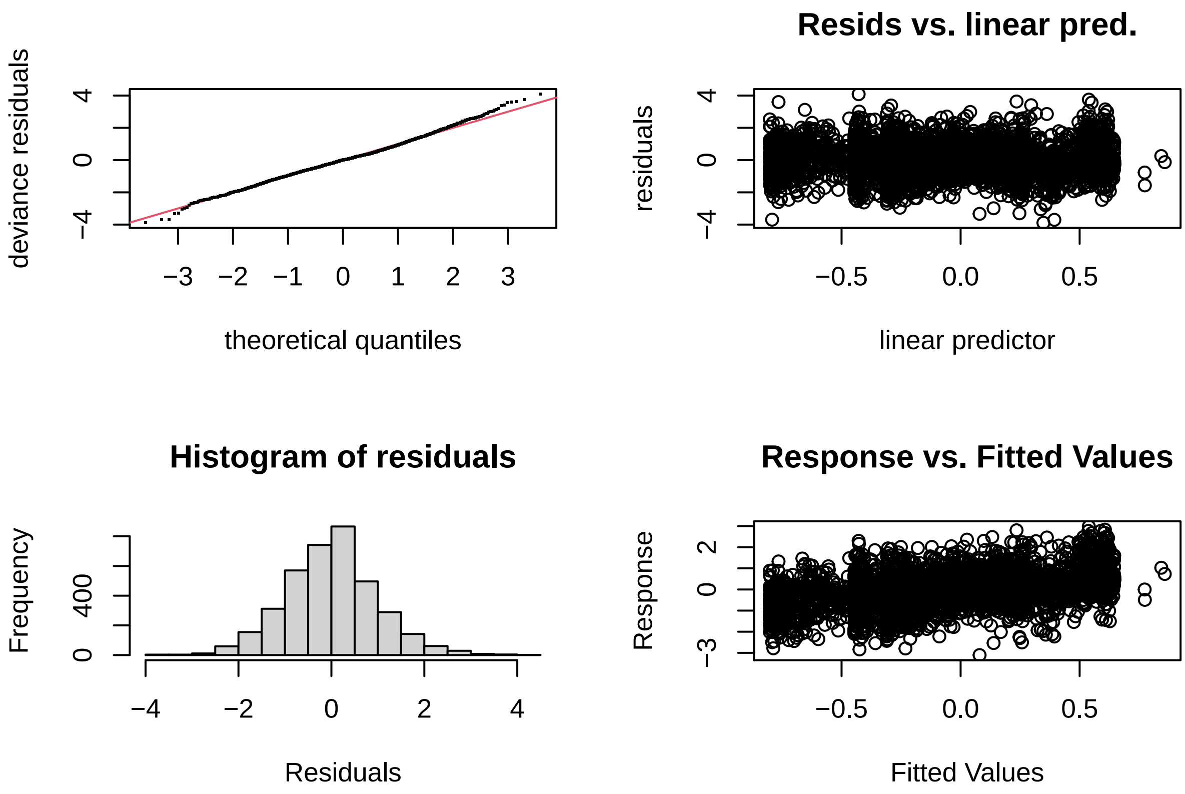


```
##
## Method: ML   Optimizer: outer newton
## full convergence after 7 iterations.
## Gradient range [-0.0006847697,0.0006281789]
## (score 4250.48 & scale 0.9697976).
## Hessian positive definite, eigenvalue range [4.77194,1875.514].
## Model rank =  12 / 12
##
## Basis dimension (k) checking results. Low p-value (k-index<1) may
## indicate that k is too low, especially if edf is close to k'.
##
##          k'  edf k-index p-value
## s(rho) 11.0 10.9     0.9  <2e-16 ***
## ---
## Signif. codes:  0 '***' 0.001 '**' 0.01 '*' 0.05 '.' 0.1 ' ' 1
```

RE

```r
gam.check(RE)
```

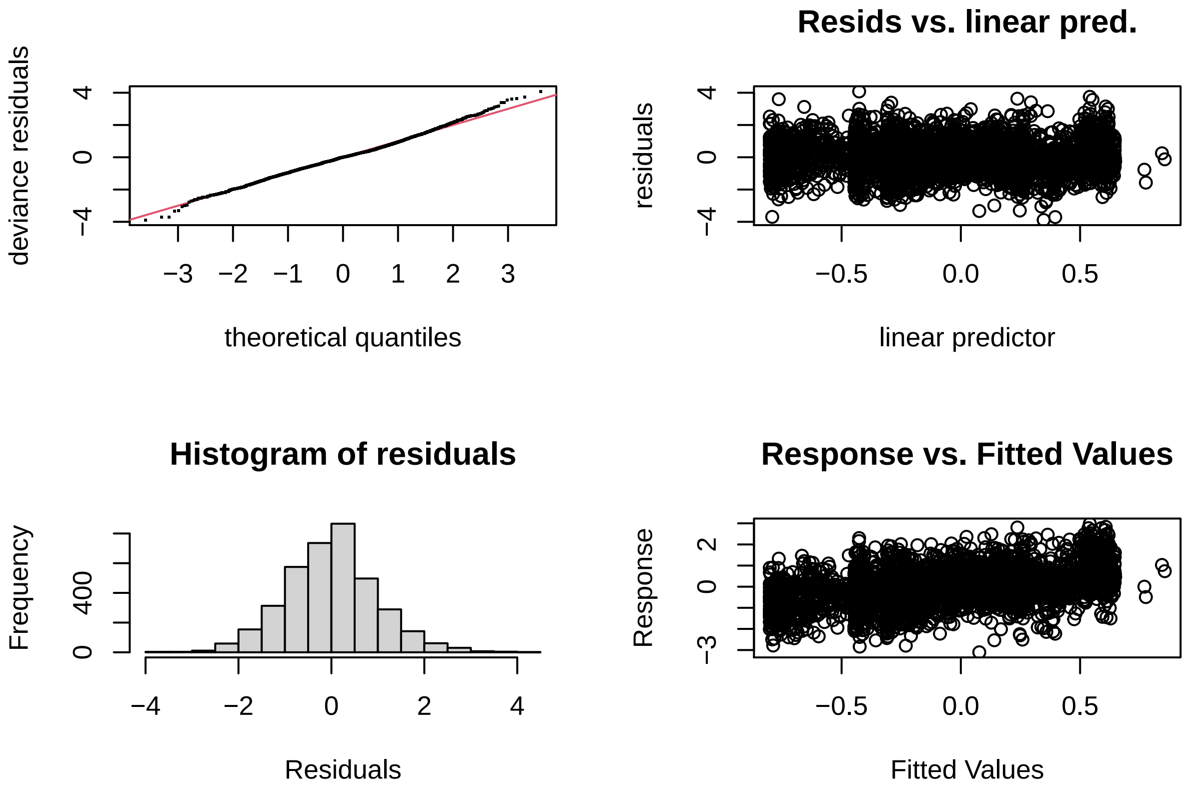


```
##
## Method: ML   Optimizer: outer newton
## full convergence after 7 iterations.
## Gradient range [-0.000718503,0.0006591574]
## (score 4250.452 & scale 0.9697821).
## Hessian positive definite, eigenvalue range [4.772145,1875.514].
## Model rank =  12 / 12
##
## Basis dimension (k) checking results. Low p-value (k-index<1) may
## indicate that k is too low, especially if edf is close to k'.
##
##          k'  edf k-index p-value
## s(rho) 11.0 10.9    0.92  <2e-16 ***
## ---
## Signif. codes:  0 '***' 0.001 '**' 0.01 '*' 0.05 '.' 0.1 ' ' 1
```

RC

```r
gam.check(RC)
```

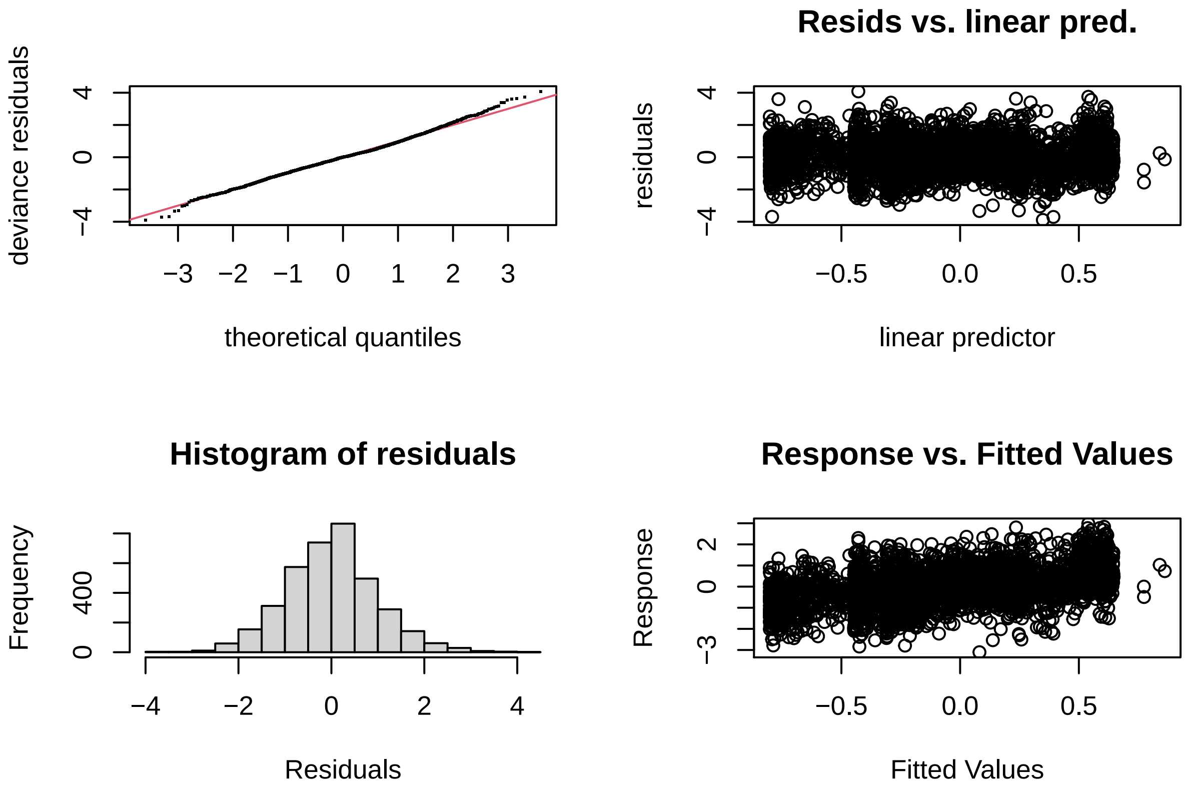


```
##
## Method: ML   Optimizer: outer newton
## full convergence after 7 iterations.
## Gradient range [-0.0006961626,0.0006386157]
## (score 4250.492 & scale 0.9698041).
## Hessian positive definite, eigenvalue range [4.771916,1875.514].
## Model rank =  12 / 12
##
## Basis dimension (k) checking results. Low p-value (k-index<1) may
## indicate that k is too low, especially if edf is close to k'.
##
##          k'  edf k-index p-value
## s(rho) 11.0 10.9    0.91  <2e-16 ***
## ---
## Signif. codes:  0 '***' 0.001 '**' 0.01 '*' 0.05 '.' 0.1 ' ' 1
```

RFE

```
gam.check(RFE)
```

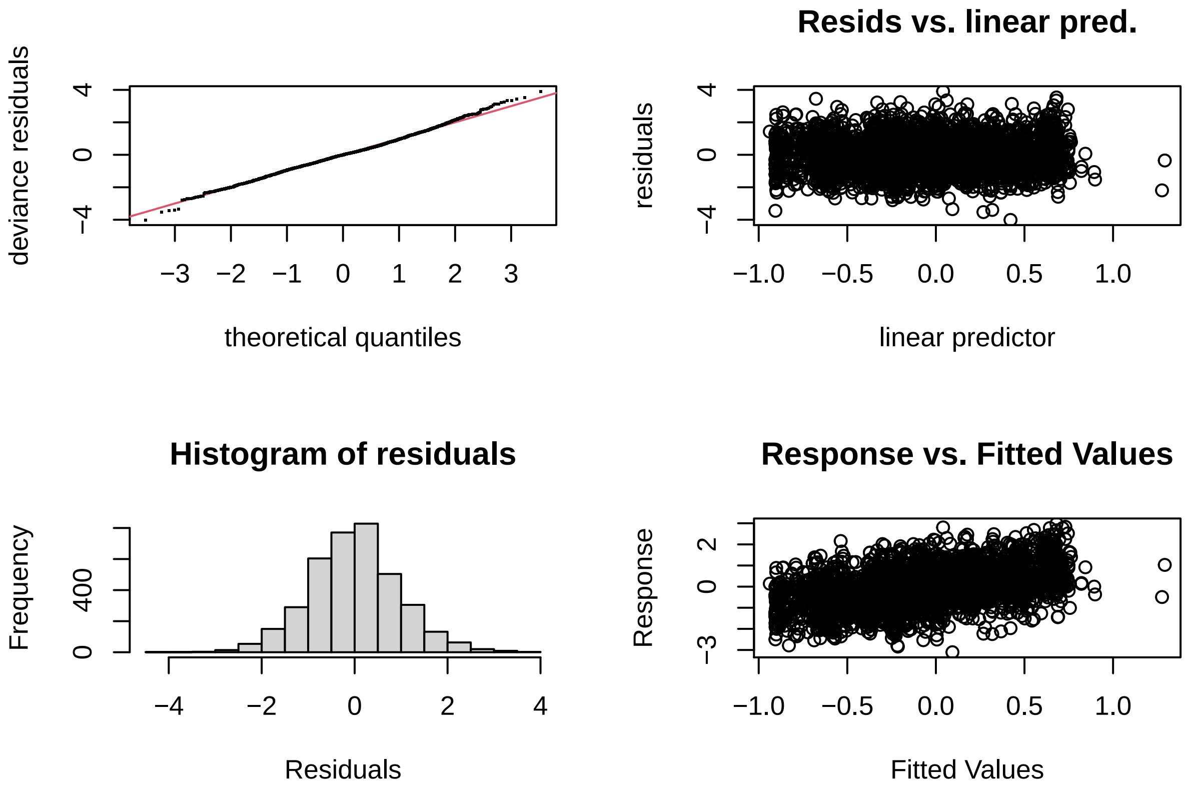


```
##
## Method: ML   Optimizer: outer newton
## full convergence after 9 iterations.
## Gradient range [-0.000226454,0.0002025754]
## (score 4215.753 & scale 0.934137).
## Hessian positive definite, eigenvalue range [4.402697,1875.526].
## Model rank =  24 / 25
##
## Basis dimension (k) checking results. Low p-value (k-index<1) may
## indicate that k is too low, especially if edf is close to k'.
##
##                   k'  edf k-index p-value
## s(rho1):north 12.0 10.9    0.81  <2e-16 ***
## s(rho2):south 12.0 11.8    0.86  <2e-16 ***
## ---
## Signif. codes:  0 '***' 0.001 '**' 0.01 '*' 0.05 '.' 0.1 ' ' 1
```

RDE

```
gam.check(RDE)
```

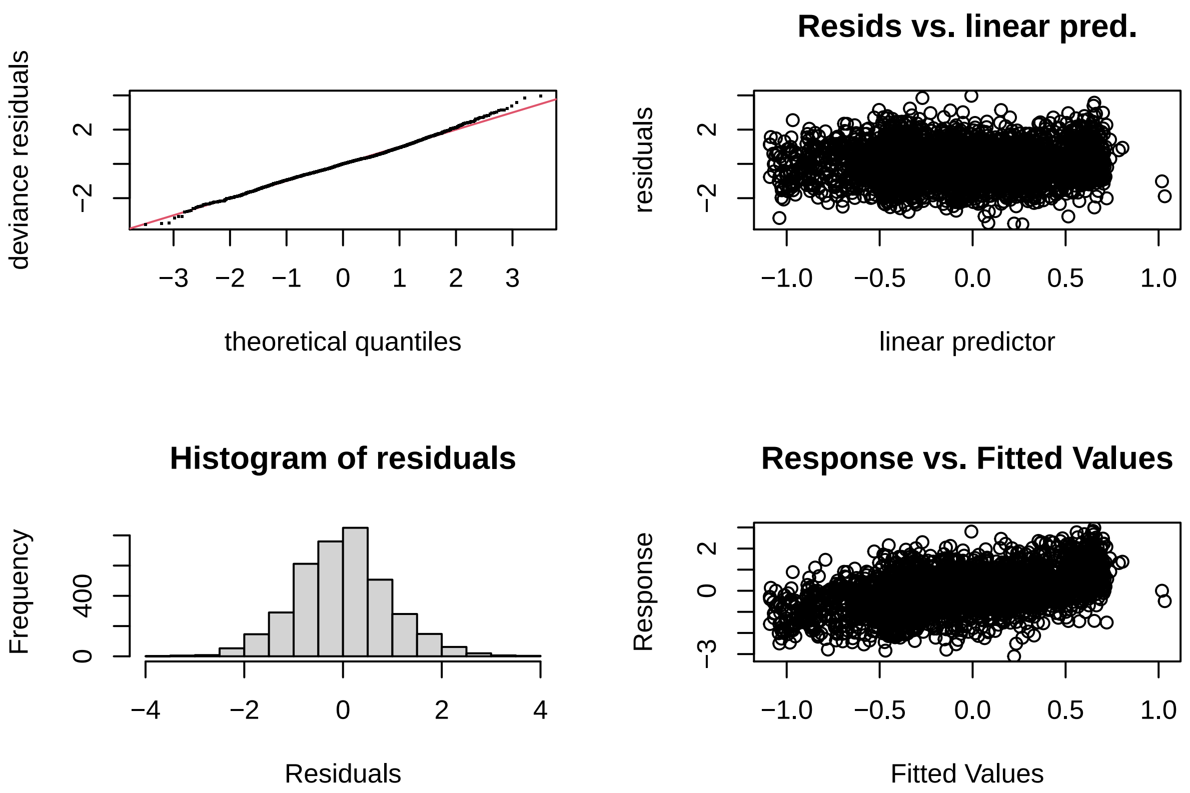


```
##
## Method: ML   Optimizer: outer newton
## full convergence after 8 iterations.
## Gradient range [-0.0002188441,0.0001232898]
## (score 4168.334 & scale 0.9194978).
## Hessian positive definite, eigenvalue range [4.043394,1875.525].
## Model rank =  23 / 23
##
## Basis dimension (k) checking results. Low p-value (k-index<1) may
## indicate that k is too low, especially if edf is close to k'.
##
##           k'  edf k-index p-value
## s(rho1) 11.0 10.1    0.98    0.13
## s(rho2) 11.0 10.9    0.93  <2e-16 ***
## ---
## Signif. codes:  0 '***' 0.001 '**' 0.01 '*' 0.05 '.' 0.1 ' ' 1
```

RFC

```
gam.check(RFC)
```

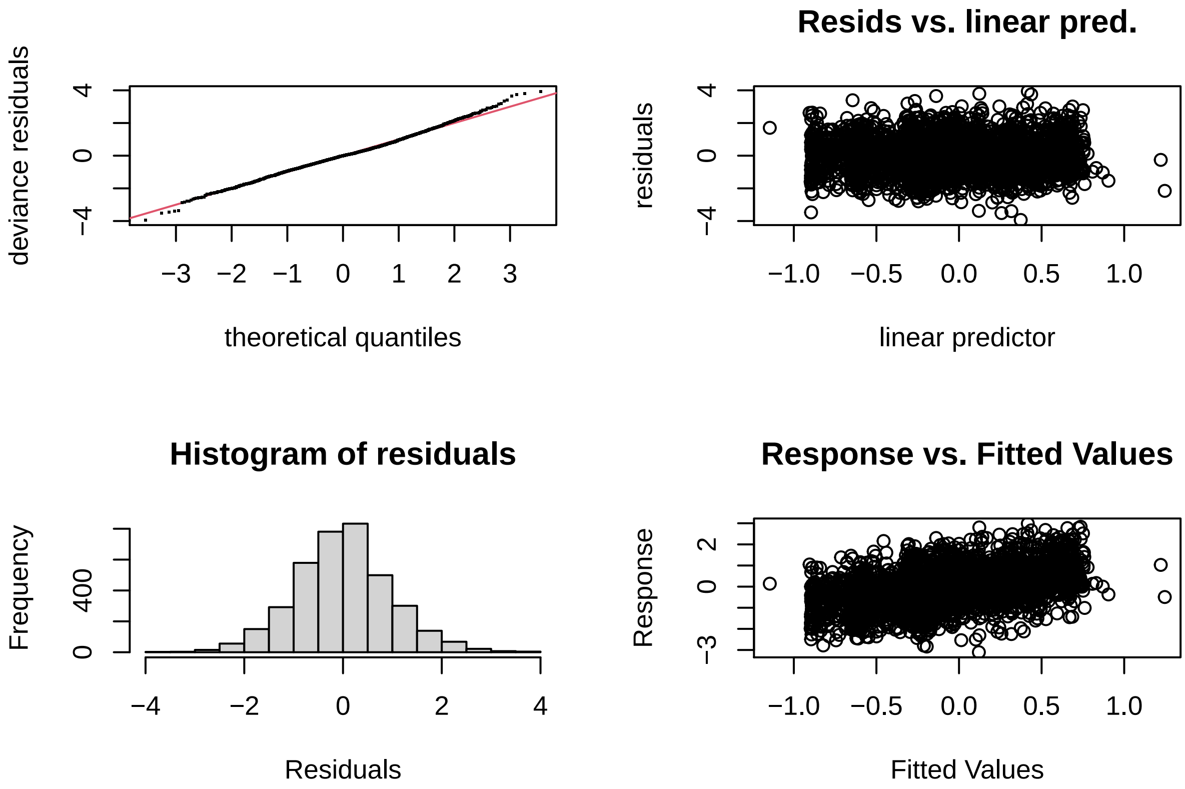


```
##
## Method: ML   Optimizer: outer newton
## full convergence after 9 iterations.
## Gradient range [-1.112319e-05,9.964846e-06]
## (score 4239.138 & scale 0.9462598).
## Hessian positive definite, eigenvalue range [4.185329,1875.526].
## Model rank =  24 / 25
##
## Basis dimension (k) checking results. Low p-value (k-index<1) may
## indicate that k is too low, especially if edf is close to k'.
##
##                  k'  edf k-index p-value
## s(rho1):north 12.0 11.9    0.82  <2e-16 ***
## s(rho2):south 12.0 10.7    0.86  <2e-16 ***
## ---
## Signif. codes:  0 '***' 0.001 '**' 0.01 '*' 0.05 '.' 0.1 ' ' 1
```

RDC

```
gam.check(RDC)
```

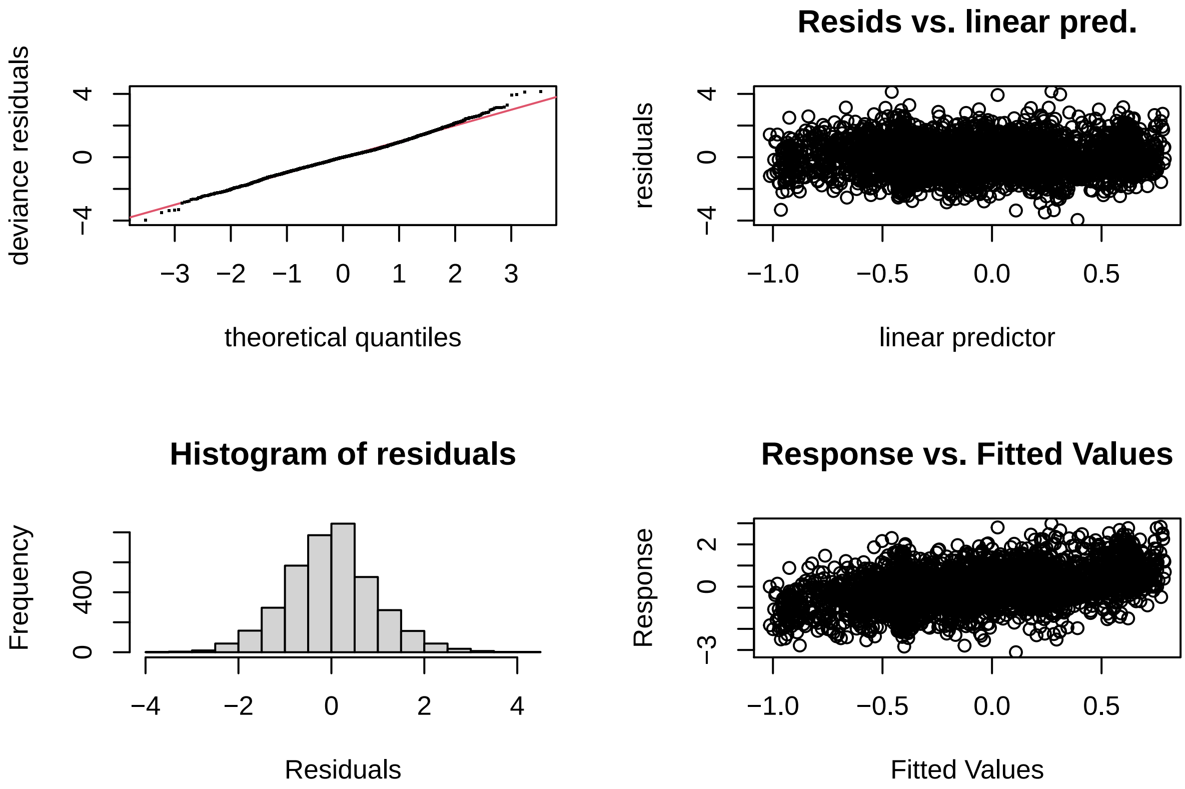


```
##
## Method: ML   Optimizer: outer newton
## full convergence after 13 iterations.
## Gradient range [-0.0013011,0.0005090112]
## (score 4220.628 & scale 0.9327492).
## Hessian positive definite, eigenvalue range [3.075838,1875.524].
## Model rank =  23 / 23
##
## Basis dimension (k) checking results. Low p-value (k-index<1) may
## indicate that k is too low, especially if edf is close to k'.
##
##           k'  edf k-index p-value
## s(rho1) 11.0 10.0    0.92  <2e-16 ***
## s(rho2) 11.0 10.1    0.95   0.005 **
## ---
## Signif. codes:  0 '***' 0.001 '**' 0.01 '*' 0.05 '.' 0.1 ' ' 1
```

PF

```
gam.check(PF)
```

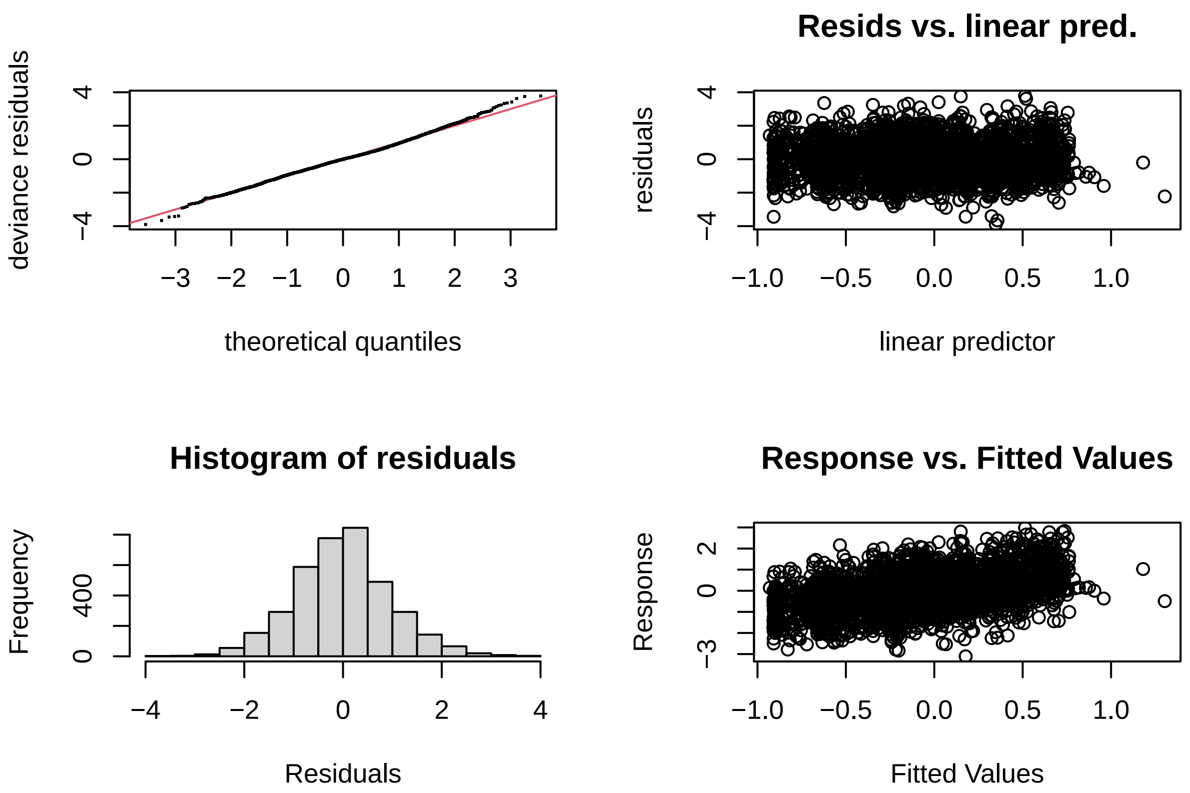


```
##
## Method: ML   Optimizer: outer newton
## full convergence after 9 iterations.
## Gradient range [-0.0008598054,0.000771823]
## (score 4227.921 & scale 0.9405916).
## Hessian positive definite, eigenvalue range [4.303538,1875.527].
## Model rank =  24 / 25
##
## Basis dimension (k) checking results. Low p-value (k-index<1) may
## indicate that k is too low, especially if edf is close to k'.
##
##                  k'  edf k-index p-value
## s(rho1):north 12.0 11.9    0.83  <2e-16 ***
## s(rho2):south 12.0 10.7    0.85  <2e-16 ***
## ---
## Signif. codes:  0 '***' 0.001 '**' 0.01 '*' 0.05 '.' 0.1 ' ' 1
```

PD

```
gam.check(PD)
```

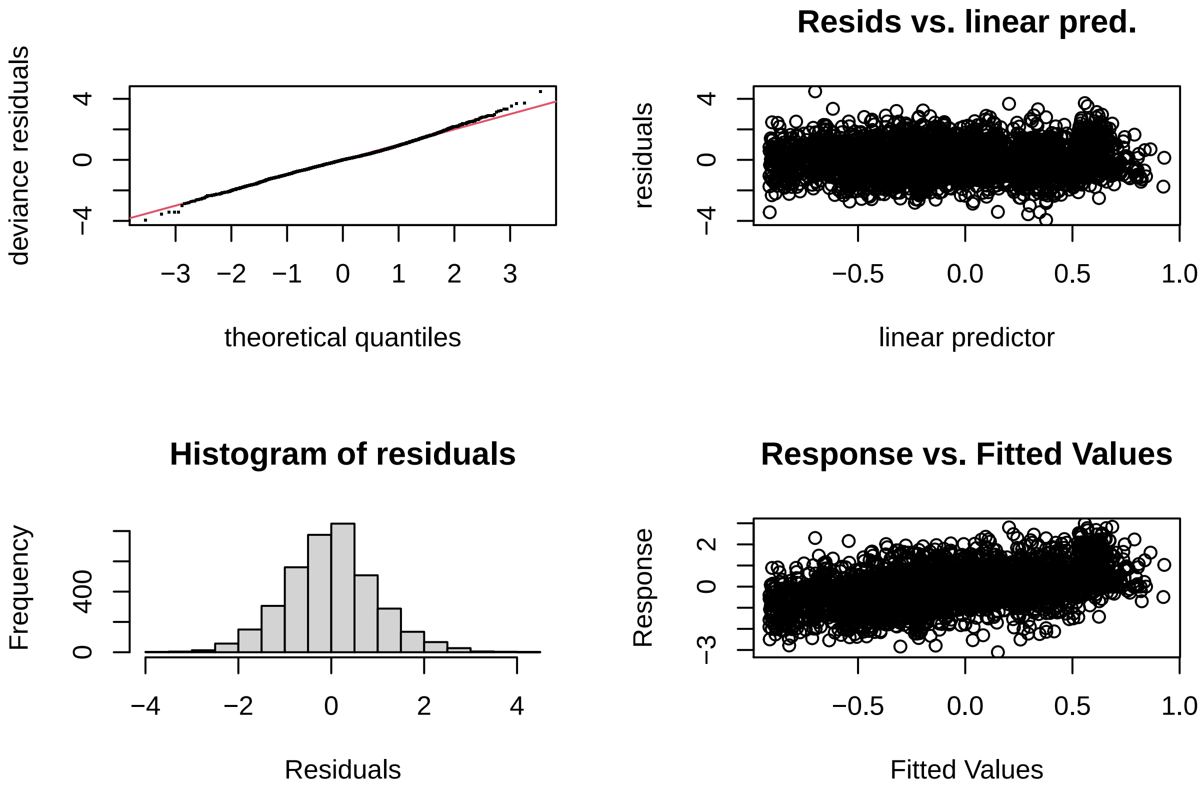


```
##
## Method: ML   Optimizer: outer newton
## full convergence after 7 iterations.
## Gradient range [-0.0003869345,0.0002625676]
## (score 4215.657 & scale 0.9429339).
## Hessian positive definite, eigenvalue range [3.690696,1875.525].
## Model rank =  23 / 23
##
## Basis dimension (k) checking results. Low p-value (k-index<1) may
## indicate that k is too low, especially if edf is close to k'.
##
##           k'  edf k-index p-value
## s(rho1) 11.0 10.1    0.97   0.055 .
## s(rho2) 11.0 10.9    0.96   0.005 **
## ---
## Signif. codes:  0 '***' 0.001 '**' 0.01 '*' 0.05 '.' 0.1 ' ' 1
```

Model summaries

N1

```
summary(N1)
```

```
##
```

```
## Family: gaussian
## Link function: identity
##
## Formula:
## r.growth ~ 1
##
## Parametric coefficients:
##             Estimate Std. Error t value Pr(>|t|)
## (Intercept) -0.05949    0.01326  -4.487 7.43e-06 ***
## ---
## Signif. codes:  0 '***' 0.001 '**' 0.01 '*' 0.05 '.' 0.1 ' ' 1
##
##
## R-sq.(adj) =      0   Deviance explained =    0%
## -ML =   4677  Scale est. = 1.2406    n = 3751
```

N2

```r
summary(N2)
```

```
##
## Family: gaussian
## Link function: identity
##
## Formula:
## r.growth ~ s(julian.mean.trans, k = 12, bs = "tp")
##
## Parametric coefficients:
##             Estimate Std. Error t value Pr(>|t|)
## (Intercept) -0.05196    0.01184  -4.388 1.18e-05 ***
## ---
## Signif. codes:  0 '***' 0.001 '**' 0.01 '*' 0.05 '.' 0.1 ' ' 1
##
## Approximate significance of smooth terms:
##                        edf Ref.df     F p-value
## s(julian.mean.trans) 10.93     11 91.67  <2e-16 ***
## ---
## Signif. codes:  0 '***' 0.001 '**' 0.01 '*' 0.05 '.' 0.1 ' ' 1
##
## R-sq.(adj) =  0.212   Deviance explained = 21.4%
## -ML = 4265.2  Scale est. = 0.97762   n = 3751
```

N3

```r
summary(N3)
```

```
##
## Family: gaussian
## Link function: identity
```

```
##
## Formula:
## r.growth ~ te(cen.x, cen.y, k = c(10, 10), bs = "tp")
##
## Parametric coefficients:
##             Estimate Std. Error t value Pr(>|t|)
## (Intercept) -0.06037    0.01325  -4.556 5.38e-06 ***
## ---
## Signif. codes:  0 '***' 0.001 '**' 0.01 '*' 0.05 '.' 0.1 ' ' 1
##
## Approximate significance of smooth terms:
##                   edf Ref.df     F p-value
## te(cen.x,cen.y) 3.006  3.011 3.039  0.0277 *
## ---
## Signif. codes:  0 '***' 0.001 '**' 0.01 '*' 0.05 '.' 0.1 ' ' 1
##
## R-sq.(adj) =  0.00164   Deviance explained = 0.244%
## -ML = 4672.5  Scale est. = 1.2385    n = 3751
```

P

```r
summary(P_TW)
```

```
##
## Family: gaussian
## Link function: identity
##
## Formula:
## r.growth ~ s(rho, k = 12, bs = "tp")
##
## Parametric coefficients:
##             Estimate Std. Error t value Pr(>|t|)
## (Intercept) -0.05223    0.01179  -4.428 9.77e-06 ***
## ---
## Signif. codes:  0 '***' 0.001 '**' 0.01 '*' 0.05 '.' 0.1 ' ' 1
##
## Approximate significance of smooth terms:
##          edf Ref.df     F p-value
## s(rho) 10.93     11 95.23  <2e-16 ***
## ---
## Signif. codes:  0 '***' 0.001 '**' 0.01 '*' 0.05 '.' 0.1 ' ' 1
##
## R-sq.(adj) =  0.218   Deviance explained = 22.1%
## -ML = 4250.5  Scale est. = 0.9698    n = 3751
```

RE

```r
summary(RE)
```

```
##
```

```
## Family: gaussian
## Link function: identity
##
## Formula:
## r.growth ~ s(rho, k = 12, bs = "tp")
##
## Parametric coefficients:
##             Estimate Std. Error t value Pr(>|t|)
## (Intercept) -0.05223    0.01179  -4.429 9.76e-06 ***
## ---
## Signif. codes:  0 '***' 0.001 '**' 0.01 '*' 0.05 '.' 0.1 ' ' 1
##
## Approximate significance of smooth terms:
##          edf Ref.df     F p-value
## s(rho) 10.93     11 95.24  <2e-16 ***
## ---
## Signif. codes:  0 '***' 0.001 '**' 0.01 '*' 0.05 '.' 0.1 ' ' 1
##
## R-sq.(adj) =  0.218   Deviance explained = 22.1%
## -ML = 4250.5  Scale est. = 0.96978   n = 3751
```

RC

```r
summary(RC)
```

```
##
## Family: gaussian
## Link function: identity
##
## Formula:
## r.growth ~ s(rho, k = 12, bs = "tp")
##
## Parametric coefficients:
##             Estimate Std. Error t value Pr(>|t|)
## (Intercept) -0.05222    0.01179  -4.428 9.79e-06 ***
## ---
## Signif. codes:  0 '***' 0.001 '**' 0.01 '*' 0.05 '.' 0.1 ' ' 1
##
## Approximate significance of smooth terms:
##          edf Ref.df     F p-value
## s(rho) 10.93     11 95.23  <2e-16 ***
## ---
## Signif. codes:  0 '***' 0.001 '**' 0.01 '*' 0.05 '.' 0.1 ' ' 1
##
## R-sq.(adj) =  0.218   Deviance explained = 22.1%
## -ML = 4250.5  Scale est. = 0.9698    n = 3751
```

RFE

```r
summary(RFE)
```

```
##
## Family: gaussian
## Link function: identity
##
## Formula:
## r.growth ~ s(rho1, by = north, k = 12, bs = "tp") + s(rho2, by = south,
##     k = 12, bs = "tp")
##
## Parametric coefficients:
##             Estimate Std. Error t value Pr(>|t|)
## (Intercept)  -9.3703     0.7629  -12.28   <2e-16 ***
## ---
## Signif. codes:  0 '***' 0.001 '**' 0.01 '*' 0.05 '.' 0.1 ' ' 1
##
## Approximate significance of smooth terms:
##                   edf Ref.df     F p-value
## s(rho1):north 10.92  11.00 77.56  <2e-16 ***
## s(rho2):south 11.78  11.99 43.78  <2e-16 ***
## ---
## Signif. codes:  0 '***' 0.001 '**' 0.01 '*' 0.05 '.' 0.1 ' ' 1
##
## Rank: 24/25
## R-sq.(adj) =  0.247   Deviance explained = 25.2%
## -ML = 4215.8  Scale est. = 0.93414   n = 3751
```

RDE

```r
summary(RDE)
```

```
##
## Family: gaussian
## Link function: identity
##
## Formula:
## r.growth ~ s(rho1, k = 12, bs = "tp") + s(rho2, k = 12, bs = "tp")
##
## Parametric coefficients:
##             Estimate Std. Error t value Pr(>|t|)
## (Intercept) -0.05303    0.01150   -4.61 4.16e-06 ***
## ---
## Signif. codes:  0 '***' 0.001 '**' 0.01 '*' 0.05 '.' 0.1 ' ' 1
##
## Approximate significance of smooth terms:
##           edf Ref.df     F p-value
## s(rho1) 10.09  10.83 17.43  <2e-16 ***
## s(rho2) 10.92  11.00 70.72  <2e-16 ***
## ---
## Signif. codes:  0 '***' 0.001 '**' 0.01 '*' 0.05 '.' 0.1 ' ' 1
```

```
##
## R-sq.(adj) =  0.259   Deviance explained = 26.3%
## -ML = 4168.3  Scale est. = 0.9195    n = 3751
```

RFC

```r
summary(RFC)
```

```
##
## Family: gaussian
## Link function: identity
##
## Formula:
## r.growth ~ s(rho1, by = north, k = 12, bs = "tp") + s(rho2, by = south,
##     k = 12, bs = "tp")
##
## Parametric coefficients:
##             Estimate Std. Error t value Pr(>|t|)
## (Intercept)   -1.782      1.024  -1.741   0.0817 .
## ---
## Signif. codes:  0 '***' 0.001 '**' 0.01 '*' 0.05 '.' 0.1 ' ' 1
##
## Approximate significance of smooth terms:
##                   edf Ref.df     F p-value
## s(rho1):north 11.91  12.00 70.63  <2e-16 ***
## s(rho2):south 10.70  10.98 30.03  <2e-16 ***
## ---
## Signif. codes:  0 '***' 0.001 '**' 0.01 '*' 0.05 '.' 0.1 ' ' 1
##
## Rank: 24/25
## R-sq.(adj) =  0.237   Deviance explained = 24.2%
## -ML = 4239.1  Scale est. = 0.94626   n = 3751
```

RDC

```r
summary(RDC)
```

```
##
## Family: gaussian
## Link function: identity
##
## Formula:
## r.growth ~ s(rho1, k = 12, bs = "tp") + s(rho2, k = 12, bs = "tp")
##
## Parametric coefficients:
##             Estimate Std. Error t value Pr(>|t|)
## (Intercept) -0.05260    0.01158  -4.541 5.77e-06 ***
## ---
## Signif. codes:  0 '***' 0.001 '**' 0.01 '*' 0.05 '.' 0.1 ' ' 1
```

```
##
## Approximate significance of smooth terms:
##           edf Ref.df     F p-value
## s(rho1) 10.03  10.71 14.09  <2e-16 ***
## s(rho2) 10.09  10.73 14.47  <2e-16 ***
## ---
## Signif. codes:  0 '***' 0.001 '**' 0.01 '*' 0.05 '.' 0.1 ' ' 1
##
## R-sq.(adj) =  0.248   Deviance explained = 25.2%
## -ML = 4220.6  Scale est. = 0.93275   n = 3751
```

PF

```r
summary(PF)
```

```
##
## Family: gaussian
## Link function: identity
##
## Formula:
## r.growth ~ s(rho1, by = north, k = 12, bs = "tp") + s(rho2, by = south,
##     k = 12, bs = "tp")
##
## Parametric coefficients:
##             Estimate Std. Error t value Pr(>|t|)
## (Intercept)  -1.8811     0.9956  -1.889   0.0589 .
## ---
## Signif. codes:  0 '***' 0.001 '**' 0.01 '*' 0.05 '.' 0.1 ' ' 1
##
## Approximate significance of smooth terms:
##                 edf Ref.df    F p-value
## s(rho1):north 11.92  12.00 71.5  <2e-16 ***
## s(rho2):south 10.74  10.98 31.8  <2e-16 ***
## ---
## Signif. codes:  0 '***' 0.001 '**' 0.01 '*' 0.05 '.' 0.1 ' ' 1
##
## Rank: 24/25
## R-sq.(adj) =  0.242   Deviance explained = 24.6%
## -ML = 4227.9  Scale est. = 0.94059   n = 3751
```

PD

```r
summary(PD)
```

```
##
## Family: gaussian
## Link function: identity
##
## Formula:
```

```
## r.growth ~ s(rho1, k = 12, bs = "tp") + s(rho2, k = 12, bs = "tp")
##
## Parametric coefficients:
##             Estimate Std. Error t value Pr(>|t|)
## (Intercept) -0.05250    0.01164   -4.51 6.68e-06 ***
## ---
## Signif. codes:  0 '***' 0.001 '**' 0.01 '*' 0.05 '.' 0.1 ' ' 1
##
## Approximate significance of smooth terms:
##           edf Ref.df     F p-value
## s(rho1) 10.11  10.84 10.96  <2e-16 ***
## s(rho2) 10.93  11.00 58.55  <2e-16 ***
## ---
## Signif. codes:  0 '***' 0.001 '**' 0.01 '*' 0.05 '.' 0.1 ' ' 1
##
## R-sq.(adj) =   0.24   Deviance explained = 24.4%
## -ML = 4215.7  Scale est. = 0.94293   n = 3751
```

Deviance explained

```
df <- data.frame(
  Model = factor(c("N1", "N2", "N3",
                   "P", "RE", "RC",
                   "RFE", "RDE",
                   "RFC", "RDC",
                   "PF", "PD")),
  Deviance = c(as.numeric(summary(N1)$dev.expl),
               as.numeric(summary(N2)$dev.expl),
               as.numeric(summary(N3)$dev.expl),
               as.numeric(summary(P_TW)$dev.expl),
               as.numeric(summary(RE)$dev.expl),
               as.numeric(summary(RC)$dev.expl),
               as.numeric(summary(RFE)$dev.expl),
               as.numeric(summary(RDE)$dev.expl),
               as.numeric(summary(RFC)$dev.expl),
               as.numeric(summary(RDC)$dev.expl),
               as.numeric(summary(PF)$dev.expl),
               as.numeric(summary(PD)$dev.expl)
  ),
  Form = c("Null", "Null", "Null",
           "Single wave", "Single wave", "Single wave",
           "Dual radial expanding", "Dual radial expanding",
           "Dual radial contracting", "Dual radial contracting",
           "Dual planar", "Dual planar")
)

df$Model <- factor(df$Model, levels = c("N1", "N2", "N3",
                                        "P", "RE", "RC",
                                        "RFE", "RDE",
                                        "RFC", "RDC",
                                        "PF", "PD"))
```

```r
ggplot(df) +
  geom_point(aes(x = Model, y = Deviance, colour = Form),
             size = 2) +
  scale_colour_brewer(palette = "Set1") +
  scale_y_continuous(label = scales::percent) +
  theme_bw() +
  labs(x = "Model hypothesis label",
       y = "Deviance explained",
       colour = "Model grouping")
```

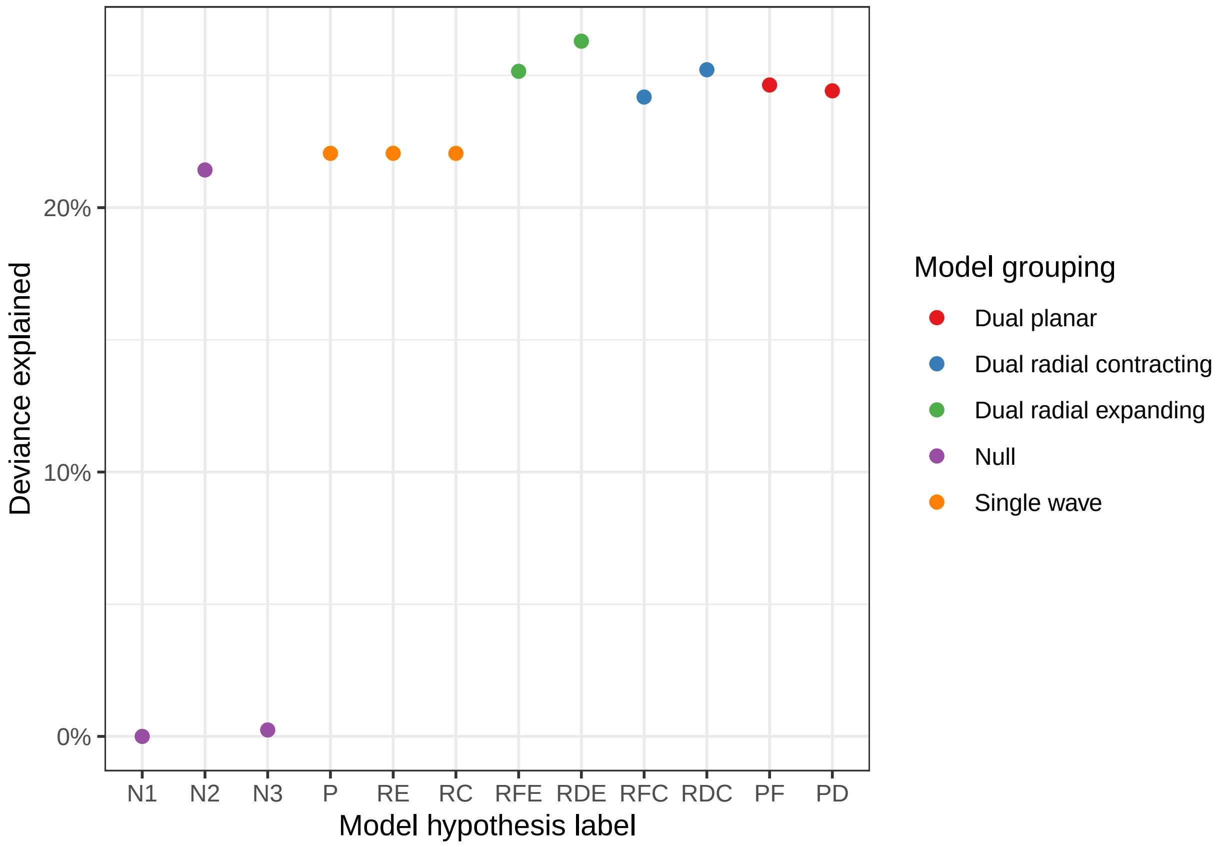

RDE residual plots

```r
space <- read.csv("C:\\Users\\r01dr16\\Desktop\\Travelling wave\\vole_TW_publication_data_roos_et_al.cs
space$resid <- residuals(RDE, type = "scaled.pearson")
space$day_bin <- cut(space$julian.mean.trans, breaks = seq(from = min(space$julian.mean.trans) - 100,
                                                           to = max(space$julian.mean.trans) + 100,
                                                           length.out = 13))

ggplot(space) +
  geom_point(aes(x = cen.x, y = cen.y, colour = resid)) +
  facet_wrap(.~ day_bin) +
  scale_colour_viridis_c(option = "C") +
  theme_bw()
```

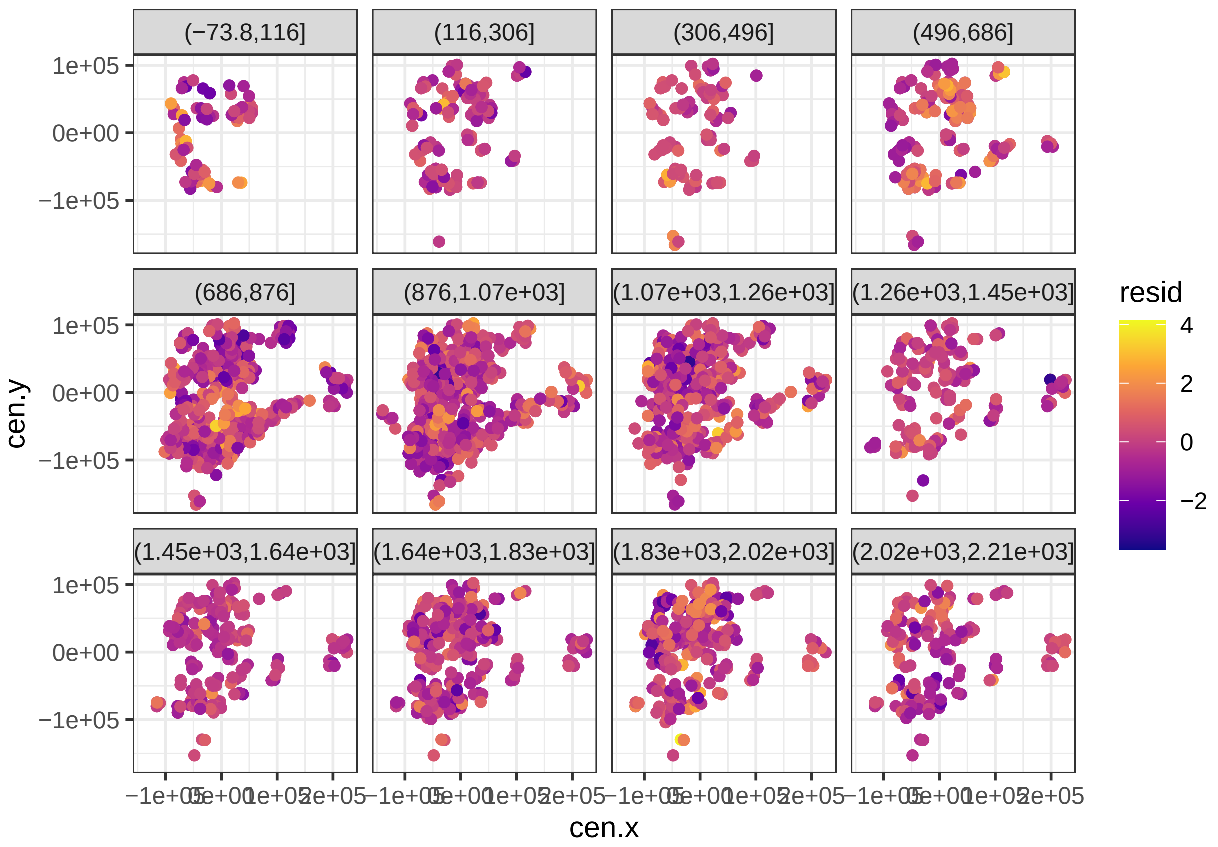
(−73.8,116]
(116,306]
(306,496]
(496,686]
(686,876]
(876,1.07e+03]
(1.07e+03,1.26e+03]
(1.26e+03,1.45e+03]
(1.45e+03,1.64e+03]
(1.64e+03,1.83e+03]
(1.83e+03,2.02e+03]
(2.02e+03,2.21e+03]
1e+05
0e+00
−1e+05
cen.y
cen.x
resid
4
2
0
−2
