# Supplementary material for: From pattern to process? Dual travelling waves, with contrasting propagation speeds, best describe a self‐organised spatio‐temporal pattern in population growth of a cyclic rodent
Source: Ecol Lett. 2022 Jul 31;25(9):1986–98. doi: 10.1111/ele.14074 (PMC9543711; doi:10.1111/ele.14074)
Supplement: Supplementary file 5 — Video S1 [file ELE-25-1986-s001.zip › ele14074-sup-0006-VideoLegends.docx]

Legends:-

Video 1: Predicted underlying spatio-temporal pattern of $\bar{r_{t,i}}$ as a result of both travelling waves (model RDE), animated over true time (i.e., days since the start of the study). Satellite imagery was retrieved from Google via ggmaps (Kahle & Wickham, 2013) and animated using gganimate (Pedersen & Robinson, 2020).

References:

Khale, D. & Wickham, H. (2013) Ggmap: spatial visualization with ggplot2. *The R Journal*, 5(1), 144–161.

Pedersen T.L., & Robinson, D. gganimate: *A Grammar of Animated Graphics*. R package version 1.0.7. https://CRAN.R‐project.org/package=gganimate
